# A practical and catalyst-free trifluoroethylation reaction of amines using trifluoroacetic acid

**DOI:** 10.1038/ncomms15913

**Published:** 2017-06-26

**Authors:** Keith G. Andrews, Radmila Faizova, Ross M. Denton

**Affiliations:** 1 School of Chemistry, University Park, University of Nottingham, Nottingham NG7 2RD, UK

## Abstract

Amines are a fundamentally important class of biologically active compounds and the ability to manipulate their physicochemical properties through the introduction of fluorine is of paramount importance in medicinal chemistry. Current synthesis methods for the construction of fluorinated amines rely on air and moisture sensitive reagents that require special handling or harsh reductants that limit functionality. Here we report practical, catalyst-free, reductive trifluoroethylation reactions of free amines exhibiting remarkable functional group tolerance. The reactions proceed in conventional glassware without rigorous exclusion of either moisture or oxygen, and use trifluoroacetic acid as a stable and inexpensive fluorine source. The new methods provide access to a wide range of medicinally relevant functionalized tertiary *β*-fluoroalkylamine cores, either through direct trifluoroethylation of secondary amines or via a three-component coupling of primary amines, aldehydes and trifluoroacetic acid. A reduction of *in situ*-generated silyl ester species is proposed to account for the reductive selectivity observed.

## Abstract (web summary)

Amino and trifluoromethyl groups are ubiquitous within medicinal chemistry, but synthesis of fluoroalkylamines normally requires harsh conditions or sensitive reagents. Here the authors report an operationally simple, catalyst-free procedure for the trifluoroethylation of primary and secondary amines.

The incorporation of fluorine into potential medicines can be used to modify conformation, basicity, intrinsic potency, membrane permeability and pharmacokinetic properties^1^,^2^. For these reasons fluorinated entities are particularly important within drug discovery and development^3^,^4^. However, the use of fluorine in this field is currently limited to a small number of chemotypes with aryl fluorides being dominant^5^,^6^,^7^,^8^,^9^. Given the beneficial properties of fluorine, practical new methods that allow access to a more diverse range of medicinally relevant fluorinated building blocks are of particular importance^10^,^11^,^12^,^13^,^14^,^15^,^16^,^17^,^18^,^19^,^20^,^21^,^22^,^23^,^24^,^25^,^26^. For example, *β*-fluoroalkylamines are less basic than their hydrocarbon counterparts (p*K*_a_H 10.7 versus 5.7 for ethylamine and trifluoroethylamine respectively)^27^ and often exhibit decreased acute toxicity and increased metabolic stability rendering them very attractive in pharmaceutical contexts (for examples, see Fig. 1b)^28^,^29^,^30^,^31^,^32^. Unfortunately, while many C–H trifluoroethylation methods have been reported^33^,^34^,^35^,^36^,^37^,^38^, there are fewer reagents for the trifluoroethylation of amines (Fig. 1a, left)^39^,^40^. The most widely used method involves LiAlH_4_ or borane reduction of trifluoromethylamides generated using trifluoroacetic anhydride (Fig. 1a)^41^,^42^. The use of pyrophoric reductants requires special precautions, limits applicability on-scale and precludes substrates that contain other reducible functional groups. Alternatively, highly reactive trifluoroacetaldehyde (b.p. −18 °C) or derivatives can be used under milder reductive conditions^43^. Recent work by Hartwig and co-workers describes palladium-catalysed *N*-arylation reactions of fluoroalkylamines^44^. While this opens up an important new synthesis route the approach is inherently limited to fluoroalkylanilines.

Herein we report a practical and catalyst-free method to access structurally diverse *β*-fluoroalkylamines using trifluoroacetic acid (TFA), which occurs through reduction of *in situ*-generated silyl ester intermediates.

## Results

### Reaction design

Seeking to develop a practical method for the fluoroalkylation of free amines, the direct use of TFA was attractive (Fig. 2), owing to its availability, low cost and stability. To the best of our knowledge reductive fluoroalkylation reactions using TFA have only been carried out in the presence of either platinum^45^ or borane^46^ catalysts under Schlenk conditions. Therefore, the development of a general and practical method with wide functional group tolerance would constitute a powerful new approach to the synthesis of fluorinated amines.

### Trifluoroethylation reactions of secondary amines

**Figure 1:**
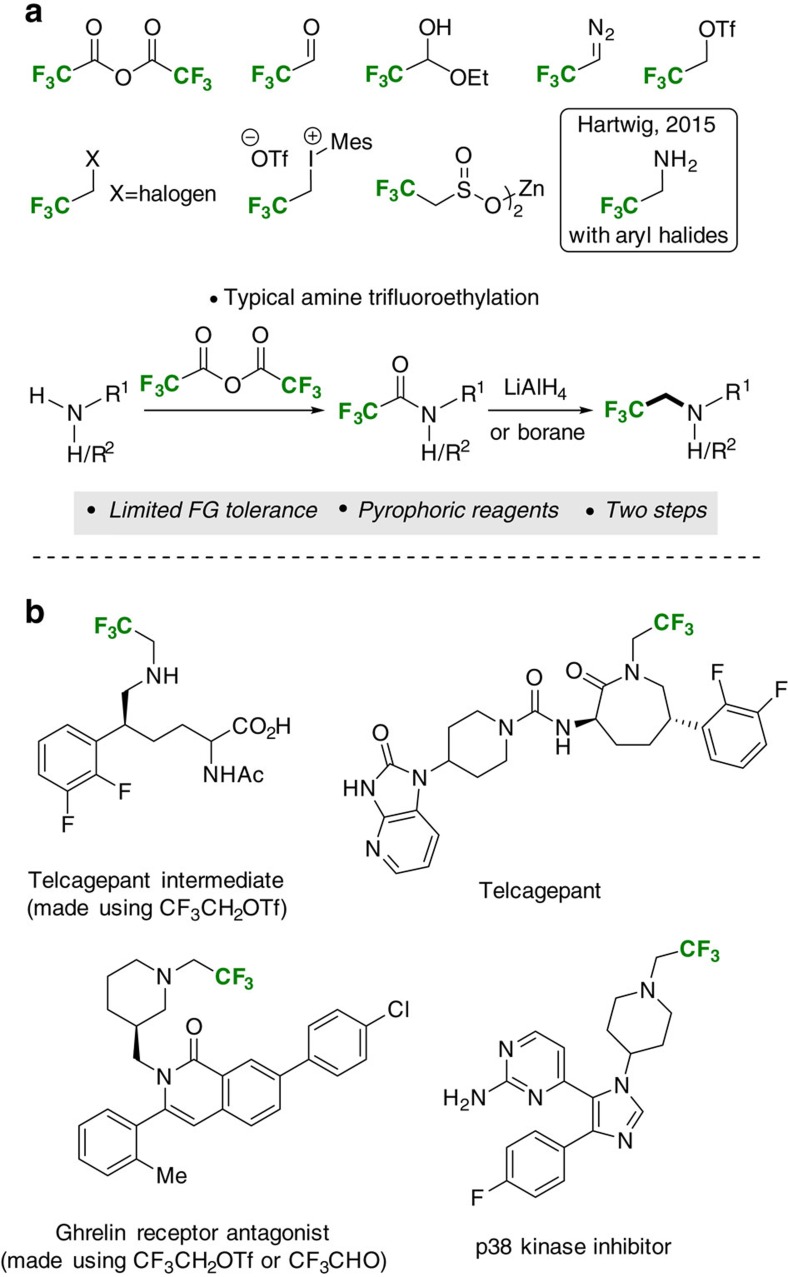
Methods for the trifluoroethylation of amines. (**a**) Existing reagents for the trifluoroethylation of amines. (**b**) Examples of *N*-trifluoroethylamines in medicinal chemistry.

**Figure 2:**
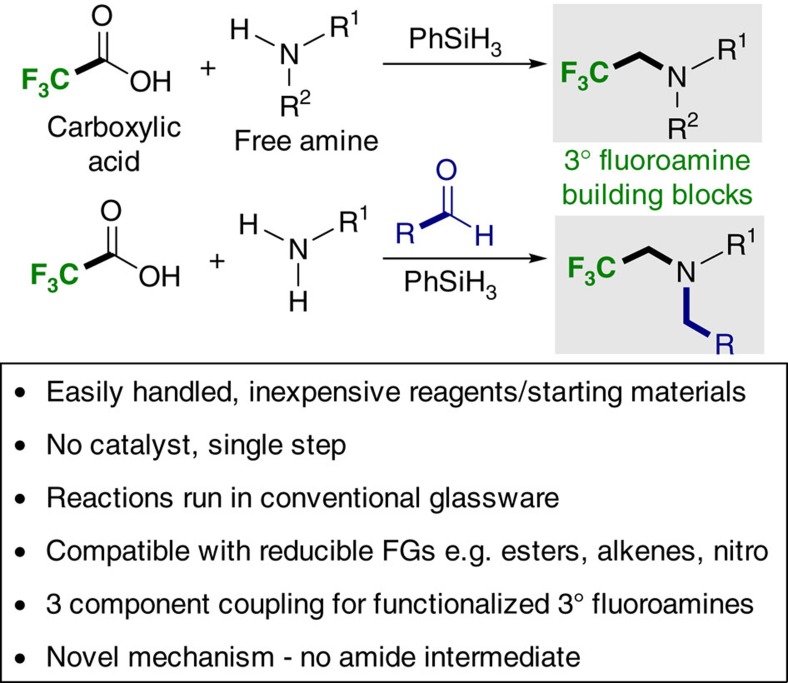
This work. A practical and catalyst-free trifluoroethylation reaction using trifluoroacetic acid.

**Figure 3:**
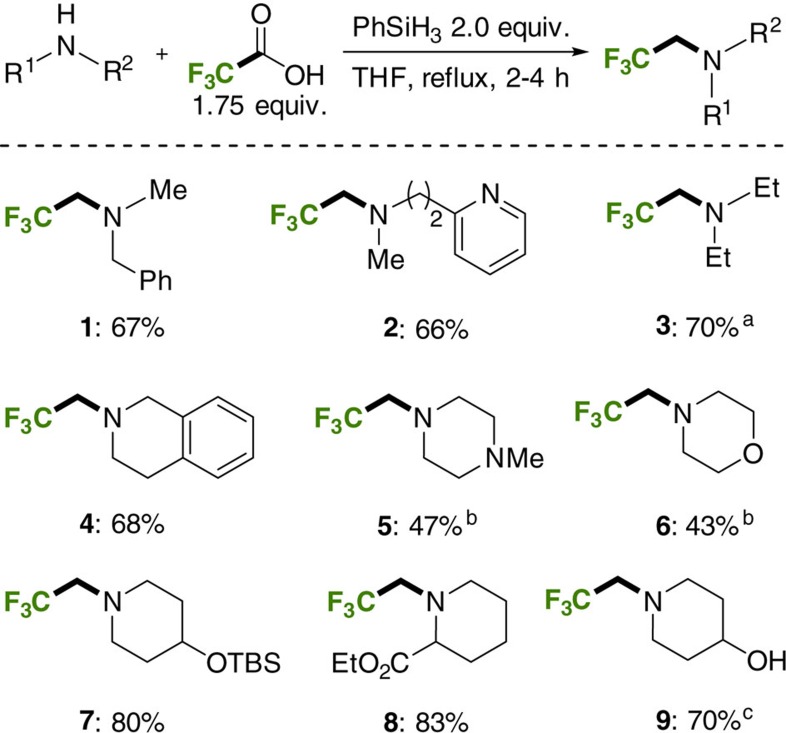
Trifluoroethylation reactions of secondary amines. Isolated yields following chromatography. ^a^Isolated as its HCl salt following aqueous work up. ^b^Product is volatile. ^c^The reaction was quenched with 1 M NaOH to desilylate the hydroxyl group (THF, tetrahydrofuran; TBS, *tert*-butyldimethylsilyl).

Trifluoroethylation reactions were performed in tetrahydrofuran, with 1.75 equivalents of TFA providing optimal results (Fig. 3; for optimization, see Supplementary Table 1). Importantly, the reactions were carried out in conventional laboratory glassware. Performing the reaction open to air in non-anhydrous solvent was not detrimental (Fig. 3; **1**) highlighting the practicality of the method. The phenylsilane and TFA were used as supplied without purification. Acyclic and cyclic secondary amines could be trifluoroethylated in good to moderate isolated yields following flash column chromatography (conversions of 70–90% are typical). Additional moderately basic nitrogen atoms (**2**, **5**) are tolerated as are silyl-protected alcohols (**7**) and free alcohols (**9**). In the case of the latter, the hydroxyl group undergoes silylation under the reaction conditions and the silyl group is cleaved upon workup with aqueous base. Most significantly, esters are not reduced under the reaction conditions (**8**). This transformation would not be possible using the conventional amidation/reduction protocol using LiAlH_4_ (ref. 43); nor could it be achieved using the Pt^45^ or borane-catalyzed reactions^46^. Anilines were relatively poor substrates, with *N*-methyl anilines typically giving less than 40% conversion to the desired amine (not shown).

### Three component trifluoroethylation reactions

**Figure 4:**
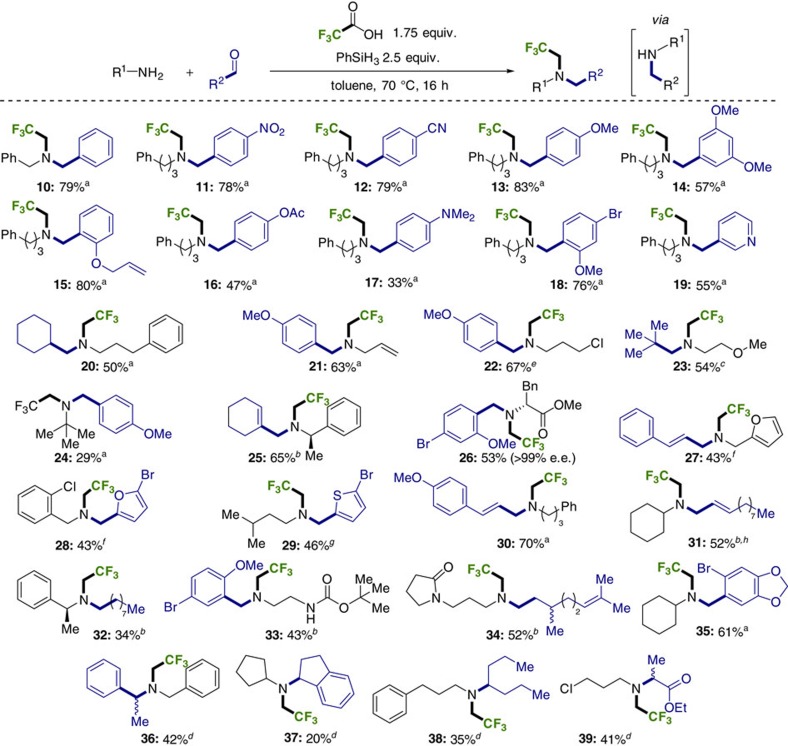
Three-component trifluoroethylation reactions. ^a^Typically, amine (0.50 mmol) and aldehyde (0.50 mmol) were mixed neat, before adding toluene (0.5 ml) and PhSiH_3_ (0.25 mmol) and stirring for 10 min at 70 °C. Trifluoroacetic acid (0.875 mmol) and further PhSiH_3_ (2.0 mmol) were added and the reaction heated at 70 °C for 16 h. The products were purified by concentration, washing an ether solution of the crude material with sodium bicarbonate and chromatography. Slight timing/temperature modifications are described in Supplementary Methods for: ^b^Method B (alkyl aldehydes), ^c^Method C/D (hindered aldehydes), ^d^Method E (ketones). ^e^5.00 mmol scale from HCl salt of amine, ^f^trifluoroacetic acid (1.50 mmol) used. ^g^trifluoroacetic acid (1.00 mmol) used, ^h^as mixture of 93:7 *trans*:*cis* alkenes.

We anticipated that we might be able to exploit the acidic, reductive conditions to perform a reductive amination of an aldehyde and a primary amine to form a secondary amine (Fig. 4; brackets), prior to the trifluoroethylation reaction. In this case, a complex, bespoke, *β*-fluorinated amine core could be generated. As before, these three-component couplings could be carried out in conventional laboratory glassware with moisture exclusion providing small yield gains. The functional group tolerance of this catalyst-free process allows the inclusion of reductively labile, but synthetically valuable, functional groups including alkenes (Fig. 4; **15**, **21**, **25**, **27**, **30**, **31**, **34**), esters (**16**, **26**, **39**), nitro (**11**), nitrile (**12**), Boc-protected amines (**33**), amides (**34**), azides (see Supplementary Discussion) and aryl bromides (**18**, **26**, **28**, **29**, **33**, **35**). Acid-sensitive functional groups, for example, furans are also tolerated (Fig. 4; **27**, **28**). The stereo-integrity of amino acid derivative **26** was preserved. Alkyl chloride **22** was prepared at gram-scale (5.00 mmol) from the HCl salt of the amine. The procedure was also amenable to ketones (**36**–**39**).

Typically, an initial imine-forming period was followed by the standard reaction, with minor timing/temperature adjustments beneficial for difficult substrates (full details in Supplementary Methods). Alkyl aldehydes (**20**, **23**, **32**, **34**) gave poorer conversions relative to stabilized analogues. Slightly increased TFA excesses were beneficial in some cases (**27–29)**. Analysis of the reaction mixtures in these cases revealed competing over-alkylation (double aldehyde addition to the primary amine), with the resulting basic tertiary amines acting to neutralize the required excess acidity. Other competing pathways include amidation of both the initial and alkylated amine.

### Mechanistic investigation

The results depicted in Figs 3 and 4 demonstrate that a general trifluoroethylation reaction with wide functional group tolerance is viable in the absence of a catalyst. To understand the process further and account for the remarkable functional group tolerance, we conducted mechanistic experiments to ascertain the provenance of the amine product.

Intuitively, the product might be expected to arise from silane-mediated amidation^47^, followed by amide reduction by an activated silane; however, an activated reductant is not consistent with the high functional group tolerance observed.

**Figure 5:**
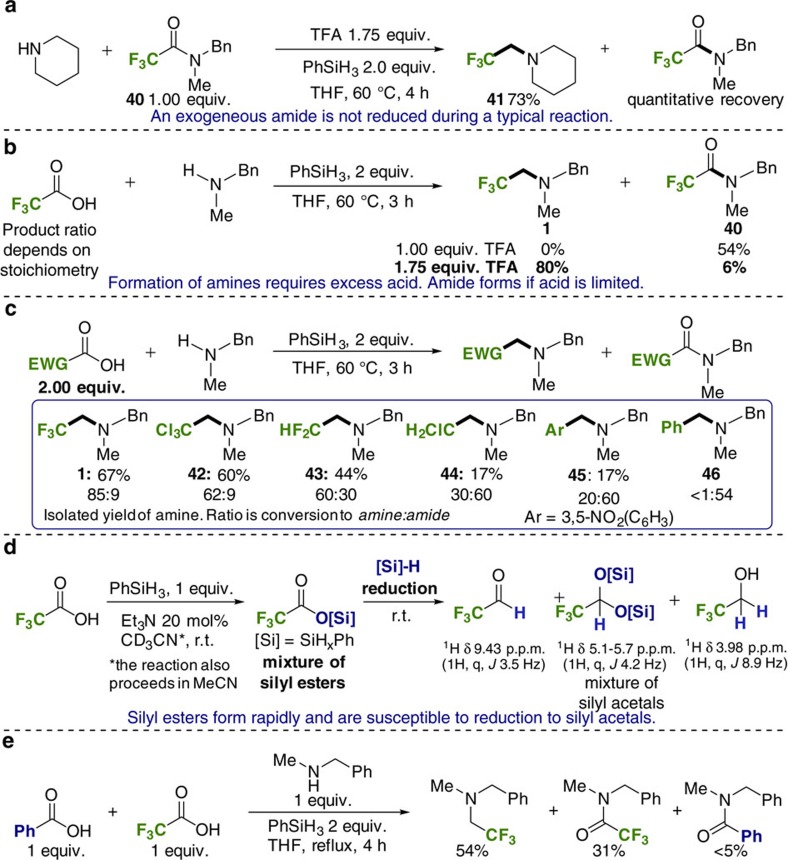
Mechanistic investigation. (**a**) Trifluoroethylation does not occur via an amide intermediate. (**b**) Acid stoichiometry controls product distribution. (**c**) Product distribution varies as a function of acid p*K*_a_/electrophilicity. (**d**) ^1^H-nuclear magnetic resonance (^1^H-NMR) experiments demonstrate the rapid generation of silyl esters followed by partial reduction to silyl acetal species. (**e**) Competition experiment with benzoic acid.

Indeed, trifluoroethylation of piperidine in the presence of trifluoroacetamide **40** resulted in the formation of trifluoroethylamine **41** and complete recovery of amide **40** (Fig. 5a). This result undermines the intervention of an activated reductant/trifluoroacetamide intermediate and implies a separate trifluoroethylation pathway. Further evidence for a discrete amination pathway was obtained when the stoichiometry of TFA to amine was examined (Fig. 5b). When the amine and TFA are equimolar, amide product **40** was obtained in moderate yield and none of the trifluoroethylated amine **1** was formed. In contrast, increasing the amount of TFA to 1.75 equivalents resulted in 80% of the fluorinated amine **1** and only 6% of amide **40**. We next examined a series of carboxylic acids and observed that the product ratio varied as a function of p*K*_a_ (Fig. 5c), with higher acidities leading to more amine. Synthetically useful amounts of alkylated amines, for which there are few convenient preparative procedures, are produced when other chlorine and fluorine-containing acids are employed (Fig. 5c, **42–44**).

Having ruled out amide reduction we next considered the possible *in situ* reduction of TFA (or a derivative) to trifluoroacetaldehyde. Given the very high reactivity of this aldehyde it is reasonable to assume that it would react rapidly under the reaction conditions and, in practice, we were unable to observe the aldehyde or derivatives during trifluoroethylation reactions. However, we could identify plausible intermediates via ^1^H NMR when a typical amine was replaced with substoichiometric (20 mol%) triethylamine (Fig. 5d), which cannot participate in either the amination or amidation reaction. Under these conditions, we observed that phenylsilane reacts with TFA to afford a mixture of silyl ester intermediates (Fig. 5d). No aldehyde was detectable at this point. Over time, the mixture became increasingly complex, due to the ability of each silicon centre to form up to three different Si–O bonds, and for polymeric hydridosiloxanes to form. After standing at room temperature for 24 h, several species derived from trifluoroacetaldehyde were visible including silyl acetals (^1^H δ 5.10–5.70 ^3^*J*_FH_=4.2 Hz) and trifluoroethanol (^1^H δ 3.98 ^3^*J*_FH_=8.9 Hz). Trifluoroacetaldehyde (^1^H δ 9.43 ^3^*J*_FH_=3.5 Hz) was itself also present in smaller amounts. These observations are consistent with silane-mediated reduction of the highly electrophilic TFA-derived silyl ester intermediates. The privileged reactivity of these trifluoroacetoxy silyl esters towards reduction is further apparent from the competition reaction depicted in Fig. 5e. In this case both phenyl and trifluoromethyl-containing silyl esters would be produced leading to amide and amine products respectively.

**Figure 6:**
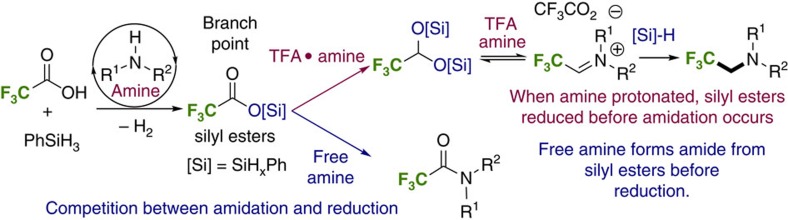
Proposed pathway for trifluoroethylation. Reduction of silyl esters to silyl acetals occurs before carbon-nitrogen bond formation.

Based upon these data we depict in Fig. 6 a plausible pathway for trifluoroethylation that is congruous with the foregoing experiments. Specifically, we propose that amine-catalysed dehydrogenative silyl ester formation occurs to generate a mixture of silyl esters, from which point it is possible to generate either amide or amine products depending on the stoichiometry of TFA. In the presence of excess acid (and, therefore, low amine concentration) silane-mediated reduction of the silyl ester intermediates occurs to afford silyl acetals and hemiacetals. Related species at this oxidation level are known to participate in reductive amination reactions with amines in the presence of acetic acid and borohydride or borane reductants^43^. Under the acidic conditions, an equilibrium^43^ of the silyl acetals with the amine to form highly reactive iminium ions is proposed. Silane-mediated reduction of the iminium species generates the observed trifluoroethylated products. Conversely, when one equivalent of TFA is used, the concentration of free amine is higher and the electrophilic silyl ester intermediates function as activated acids leading to amide products.

## Discussion

In summary, we have demonstrated the first catalyst-free trifluoroethylation reactions of amines using TFA as a bench stable and inexpensive fluorine source. The reactions proceed in conventional glassware without rigorous exclusion of either moisture or air. A wide range of functionalized tertiary *β*-fluoroalkylamines has been prepared either through direct trifluoroethylation of secondary amines or via a three-component coupling of primary amines, aldehydes and TFA. Importantly, the reaction manifold is applicable to other carboxylic acids, opening the door to an extended range of useful alkylated amines. This novel catalyst-free reaction is expected to be a powerful tool for the construction of functionalized tertiary *β*-fluoroalkylamines, thereby expanding the range of fluorinated organic compounds for applications in drug discovery, agrochemistry, catalysis and materials science.

## Methods

### General methods

See Supplementary Methods for further details, and Figs 1–136 for additional data, supporting experiments and spectra.

### General procedure for trifluoroethylation of secondary amines

To an oven-dried 10 ml round-bottomed flask fitted with a water condenser under an argon atmosphere (balloon) was added tetrahydrofuran (0.5 ml) and the amine (0.50 mmol) as the free base. The reaction flask was heated in an oil bath at 70 °C and added immediately by microsyringe was phenylsilane (123 μl, 1.00 mmol) followed by TFA (67.0 μl, 0.875 mmol). The reaction was stirred at reflux for 2–4 h, allowed to cool and was concentrated. After dilution with diethyl ether, the material was washed with saturated aqueous sodium bicarbonate, the organics dried over magnesium sulfate and the material purified by flash column chromatography to give the trifluoroethylated amine product.

### General procedure for three-component trifluoroethylation reactions

To an oven-dried 10 ml round-bottomed flask fitted with a water condenser under an argon atmosphere (balloon) was added the amine (0.50 mmol) and aldehyde (0.50 mmol). Toluene was added (0.5 ml), followed by phenylsilane (31 μl, 0.25 mmol). The reaction was stirred at 70 °C for 10 min. Then TFA (67.0 μl, 0.875 mmol) and further PhSiH_3_ (123 μl, 1.00 mmol) were added and the reaction heated at 70 °C for 16 h, and processed as above to yield the trifluoroethylated amine.

### Data availability

All data supporting the findings of this study are available within the article and its Supplementary Information file or from the authors on reasonable request. For NMR spectra of the compounds in this article, see Supplementary Figs 11–136.

## Additional information

**How to cite this article:** Andrews, K. G. *et al*. A practical and catalyst-free trifluoroethylation reaction of amines using trifluoroacetic acid. *Nat. Commun.* **8,** 15913 doi: 10.1038/ncomms15913 (2017).

**Publisher’s note**: Springer Nature remains neutral with regard to jurisdictional claims in published maps and institutional affiliations.

## Supplementary Material

Supplementary Information Supplementary figures, supplementary table, supplementary discussion, supplementary methods and supplementary references.
