# Supplementary material for: A practical and catalyst-free trifluoroethylation reaction of amines using trifluoroacetic acid
Source: Nat Commun. 2017 Jun 26;8:15913. doi: 10.1038/ncomms15913 (PMC5490195; doi:10.1038/ncomms15913)
Supplement: Supplementary Information — Supplementary figures, supplementary table, supplementary discussion, supplementary methods and supplementary references. [file ncomms15913-s1.pdf]

Supplementary Methods

General Information

Unless stated otherwise, the following procedural techniques were used: Glassware was dried in an oven (140 °C) overnight before use. All reactions were carried out under an argon balloon atmosphere. All reagents were used as supplied unless otherwise stated. Toluene and THF were obtained from typical solvent stills and stored over sodium wire under argon before use. Thin layer chromatography was carried out on Polgram SIL G/UV254 silica-aluminium plates and plates were visualized using ultra-violet light (254 nm) or a $KMnO_4$ stain. For flash column chromatography, fluorochem silica gel 60, 35–70 mesh was used. NMR data was collected at 400 or 270 MHz for ^{1}H; 101 or 68 MHz for ^{13}C, and 376 MHz for ^{19}F. Data was manipulated directly from the spectrometer or via a networked PC with appropriate software (Mestrenova or ACD labs). Reference values for residual solvent were taken as δ=7.26 ($CDCl_3$) and δ=4.79 (D_2O) for ^{1}H NMR; δ=77.00 ($CDCl_3$) for ^{13}C NMR; ^{19}F-NMR shifts were referenced to $CFCl_3$ at 0.0 ppm. NMR-yields were calculated relative to one or half an equivalent of 1,1,2,2-tetrachloroethane as an internal standard; ^{1}H NMR (400 MHz, $CDCl_3$) δ 5.94 (s, 2H). Multiplicities for coupled signals are designated using the following abbreviations: s=singlet, d=doublet, t=triplet, q=quartet, p=pentet, sex=sextet, h=heptet, br.=broad signal. The coupling constants are reported in Hertz (Hz). ^{13}C multiplicities were assigned using a DEPT sequence. Carbon shifts reported *per* environment: (2C) = two overlapped distinct environments. Where appropriate, COSY, HMQC and HMBC experiments were performed to aid assignment. High-resolution mass spectrometry data are quoted to four decimal places (0.1 mDa). Mass spectra were acquired on a VG micromass 70E, VG autospec or micromass LCTOF. Infrared spectra were recorded on a Perkin–Elmer 1600 FTIR instrument as dilute chloroform solutions. Melting points were recorded on a Stuart manual melting point apparatus. GCMS and HPLC data was obtained as described below:

GCMS – machine specifications: Instrument Parameters: Electron Ionisation --- Acquisition mass range 50-500Da --- MS Ion Source conditions: Temperature: 150 °C Ionisation energy: 70eV --- Mass Calibration by PFTBA --- Chromatography conditions: GC column: J&W DB-5MS 30m x 0.25mm x 25um --- Column temperature programme: 40 °C to 260 °C @ 30 °C min^{-1} --- Carrier gas: Helium --- Inlet temperature: 180 °C --- Transfer line temperature: 150 °C --- Instrument: Mass Spectrometer: JEOL AccuTOF GCX (JEOL Ltd., Tokyo, Japan) --- Gas Chromatograph: Agilent 7890B (Agilent Technologies Inc, Wilmington, USA). (data given includes: rt = retention time.)

Enantiopurities were determined by Reach Separations (BioCity Nottingham, Pennyfoot Street, Nottingham NG1 1GF – http://www.reachseparations.com/).

General Procedure for the trifluoroethylation of secondary amines with trifluoroacetic acid

See also additional notes below

To an oven-dried 10 mL round-bottomed flask(see note a) fitted with a water condenser under an argon atmosphere (balloon) was added THF (0.5 mL) and the amine (0.50 mmol) as the free base.(note b for HCl salt variation) The reaction flask was submerged up to the solvent level in an oil bath at 70 °C and added immediately by microsyringe *via* partial, *brief*, removal of the condenser was phenylsilane (123 µL, 1.00 mmol) followed by trifluoroacetic acid (67.0 µL, 0.875 mmol). ***(NOTE: rapid and copious H_2 gas evolution).***[c] The reaction was then stirred at reflux for 2-4 h. The reaction was allowed to cool and was concentrated.[d] The crude reaction was diluted with ether and either:

Purification A) washed with a saturated aqueous sodium bicarbonate solution,[e] the organics dried over magnesium sulfate, and the solution concentrated to about 2 mL volume.[f] This material was then purified by flash column chromatography, dry loading the oil and eluting with ethyl acetate/pentane (ratios specified below).[g]

OR

Purification B) extracted x3 into aqueous HCl (3 M). The combined aqueous layers were basified to ≈pH 10 with an aqueous NaOH solution (6 M) and extracted with dichloromethane x3. The combined organics were dried over magnesium sulfate and concentrated to dryness to give the amine as the free base directly. The material could optionally be precipitated as the HCl salt by the addition of HCl in ether (1 M) and filtration.

Notes on General Procedures

a) The reaction retains a high level of performance in non-dried glassware, Winchester grade THF and open to air (see Supplementary Table 1).

b) A limited range of amine HCl salts have been trialled. For instance, the HCl salt of methylbenzylamine was used in the presence of triethylamine (0.50 mmol, 1 equiv) and resulted in an 83% conversion to the desired amine. A white precipitate was present through-out the reaction, which did not seem to interfere. See also **compound 22**.

c) **Scale-Up:** Reactions should be conducted in a flask at least 20 times the solvent volume, particularly on scale, due to the vigorous hydrogen gas evolution. For reactions above 2.0 mmol, the phenylsilane should be added last, at room temperature, and the flask gradually warmed to temperature. These reactions should NOT be performed in a sealed vessel. The largest reaction performed here was at 5.0 mmol scale.

d) At this point, the conversion was optionally determined by analysing the ^{1}H-NMR spectrum in the presence of an internal standard (1,1,2,2-tetrachloroethane: ($CDCl_3$) s, 2H, δ = 5.94).

e) If the product is not base-sensitive, the majority of the silane waste can be removed in the work-up stage by washing the ether layer thoroughly with a 1 M sodium hydroxide solution.

f) At this point, leaving the crude material in its fully concentrated form often leads to the precipitation of silicon-based solid material, which can trap product material and reduce recovery of the product, so it is beneficial to leave the crude material dissolved in dichloromethane or to column the crude material immediately. (Low molecular weight amines often become relatively volatile as the trifluoroethylated product.)

g) Silicon-waste precipitation sometimes hinders using the eluent dissolution loading technique for chromatography. Note that, unless a second more basic amine was present in the molecule, it was never necessary to add additional base (e.g. Et_3N) to the column eluent. Elution of product was typically achieved using a manually regulated gradient (x-y%) of the more polar solvent, increasing in even intervals of the lowest significant figure (e.g. 3-6% = 3,4,5,6%: 10-40% = 10,20,30,40%).

Synthesis and characterization data for compounds 1-9

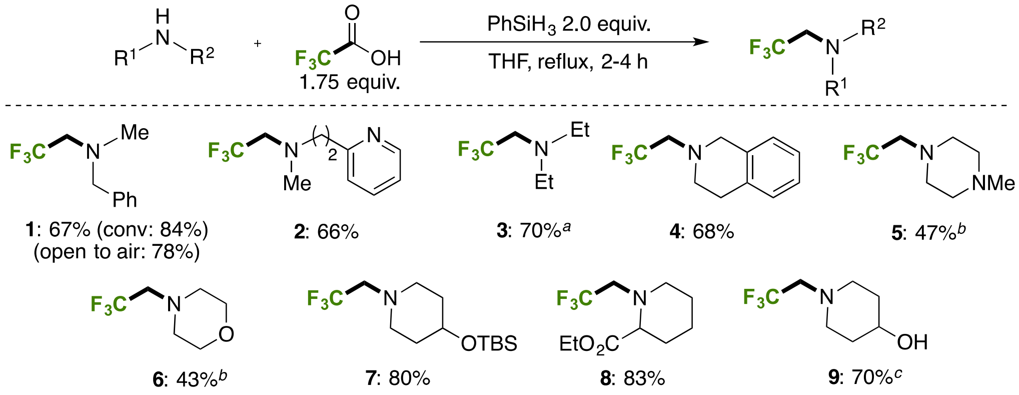

N-benzyl-2,2,2-trifluoro-*N*-methylethan-1-amine (**1**)[1]

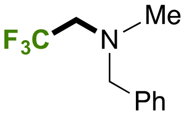

1: 67%

Title compound prepared using the general trifluoroethylation method. Purification using method B (acid/base work-up and salt precipitation) to give a clear oil (68 mg, 67%).

^{1}**H NMR** (400 MHz, $CDCl_3$) δ 7.45 – 7.27 (m, 5H), 3.74 (s, 2H), 3.06 (q, *J* = 9.6 Hz, 2H), 2.46 (s, 3H); ^{13}**C NMR** (101 MHz, $CDCl_3$) δ 138.1, 128.8, 128.4, 127.4, 125.8 (q, *J* = 281.4 Hz), 62.1, 56.8 (q, *J* = 30.3 Hz), 42.8; ^{19}**F NMR** (376 MHz, $CDCl_3$) δ – 68.8 (t, *J* = 9.6 Hz); **IR** (cm^{-1}) ($CDCl_3$) 2964, 2797, 1455, 1318, 1272, 1148, 1095; **HRMS** (ESI+): Exact mass calcd for $C_{10}H_{12}NF_3$ [M+H], 204.095. Found 204.0996, σ = 0.0035.

2,2,2-trifluoro-*N*-methyl-*N*-(2-(pyridin-2-yl)ethyl)ethan-1-amine (**2**)

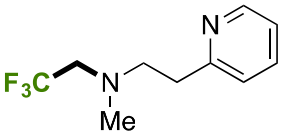

2: 66%

Title compound prepared using the general trifluoroethylation method. Purification A (chromatography, 25-65% EtOAc in pentane with 0.5% Et_3N) to give a pungent, pale yellow oil (72 mg, 66%).

^{1}**H NMR** (400 MHz, $CDCl_3$) δ 8.52 (ddd, *J* = 4.9, 1.8, 1.1 Hz, 1H), 7.58 (ddd, *J* = 7.8, 7.6, 1.8 Hz, 1H), 7.17 (d, *J* = 7.8 Hz, 1H), 7.11 (ddd, *J* = 7.6, 4.9, 1.1 Hz, 1H), 3.05 (q, *J* = 9.6 Hz, 2H), 3.00 – 2.92 (m, 4H), 2.50 (s, 3H); ^{13}**C NMR** (101 MHz, $CDCl_3$) δ 159.7, 149.3, 136.3, 125.7 (q, *J* = 281.0 Hz), 123.3, 121.3, 57.8, 57.5 (q, *J* = 30.4 Hz), 43.0, 36.3; ^{19}**F NMR** (376 MHz, $CDCl_3$) δ -69.60 (t, *J* = 9.6 Hz); **IR** (cm^{-1}) ($CDCl_3$) 2961, 2256, 1593, 1476, 1320, 1147, 1101; **HRMS** (ESI+): Exact mass calcd for $C_{10}H_{13}F_3N_2Na$ [M+Na], 241.0923. Found 241.0936, σ= 0.0063.

N,N-diethyl-2,2,2-trifluoroethan-1-amine hydrochloride (**3**)[2]

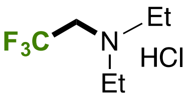

3: 70%

Title compound prepared on 5.0 mmol scale using the general trifluoroethylation method. The product was distilled with the solvent to give a solution in THF, and the amine was then precipitated as the HCl salt by the addition of HCl in ether, 1 M to give a white solid (677 mg, 70%).

^{1}H NMR (270 MHz, D_2O) δ 4.12 (q, *J* = 8.9 Hz, 2H), 3.38 (q, *J* = 7.1 Hz, 4H), 1.31 (t, *J* = 7.1 Hz, 6H); **^{13}C NMR** (126 MHz, D_2O) δ 119.5 (q, *J* = 284.5 Hz), 51.3 (q, *J* = 34.3 Hz), 49.8, 7.8; **^{19}F NMR** (376 MHz, D_2O) -66.1 (t, *J* = 8.9 Hz); **IR** (cm^{-1}) ($CDCl_3$) 2979, 1264, 1191, 1144, 1027; **HRMS** (ESI+): Exact mass calcd for $C_6H_{12}NF_3$ [M+H], 156.0922. Found 156.0955 σ < 0.05.

2-(2,2,2-trifluoroethyl)-1,2,3,4-tetrahydroisoquinoline (**4**)[1]

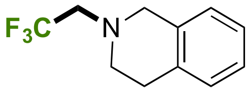

4: 68%

Title compound prepared using the general trifluoroethylation method. Purification B (acid/base work-up as described above) to give a pale yellow oil (73 mg, 68%).

^{1}H NMR (270 MHz, $CDCl_3$) δ 7.23 - 7.06 (m, 3H) 7.02 (d, *J* = 5.1 Hz, 1H) 3.89 (s, 2H) 3.16 (q, *J* = 9.6 Hz, 2H) 3.08 - 2.81 (m, 4H); **^{13}C NMR** (68 MHz, $CDCl_3$) δ 133.9, 133.7, 128.8, 126.5, 126.3, 125.8, 125.5 (q, *J* = 280.1 Hz), 57.9 (q, *J* = 30.6 Hz), 55.9, 51.4, 28.6; **^{19}F NMR** (376 MHz, $CDCl_3$) δ -69.2 (t, *J* = 9.6 Hz); **IR** (cm^{-1}) ($CDCl_3$) 3009, 2928, 2809, 1603, 1319, 1272, 1147, 1100; **HRMS** (ESI+): Exact mass calcd for $C_{11}H_{13}F_3N$ [M+H], 216.095. Found 216.105, σ= 0.0420.

1-methyl-4-(2,2,2-trifluoroethyl)piperazine (**5**)

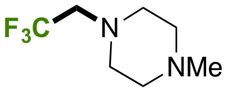

5: 47%

Title compound prepared using the general trifluoroethylation method. Purification B (acid/base work-up as described above) to give a pale yellow oil (43 mg, 47%).

^{1}H NMR (270 MHz, $CDCl_3$) δ 2.96 (q, *J* = 9.6 Hz, 2H), 2.72 (t, *J* = 4.8 Hz, 4H), 2.55-2.44 (m, 4H), 2.31 (s, 3H); **^{13}C NMR** (68 MHz, $CDCl_3$) δ 125.3 (q, *J* = 279.3 Hz), 58.4 (q, *J* = 30.3 Hz), 54.9, 53.4, 45.9; **^{19}F NMR** (376 MHz, $CDCl_3$) -68.9 (t, *J* = 9.6 Hz); **IR** (cm^{-1}) ($CDCl_3$) 2927, 2855, 1239, 1137, 1026; **HRMS** (ESI+): Exact mass calcd for $C_7H_{13}F_3N_2$ [M+H], 183.1064. Found 183.1111, σ = 0.0055.

4-(2,2,2-trifluoroethyl)morpholine (**6**)[3]

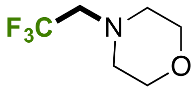

6: 43%

Title compound prepared using the general trifluoroethylation method. Purification B (acid/base work-up as described above) to give a pale yellow oil (40 mg, 47%).

^{1}H NMR (400 MHz, $CDCl_3$) δ 3.76 – 3.68 (m, 4H), 2.95 (q, *J* = 9.6 Hz, 2H), 2.69 – 2.63 (m, 4H); **^{13}C NMR** (101 MHz, $CDCl_3$) δ 125.5 (q, *J* = 283.9 Hz) 66.9, 59.2 (q, *J* = 30.1 Hz), 54.0; **^{19}F NMR** (376 MHz, $CDCl_3$) δ -69.1 (t, *J* = 9.6 Hz); **IR** (cm^{-1}) ($CDCl_3$) 3689, 3619, 3006, 2976, 1601, 1391, 1240, 1046, 909; **HRMS** (ESI+): Exact mass calcd for $C_6H_{11}F_3NO$ [M+H], 170.0787. Found 170.0791, σ= 0.0128.

4-((tert-butyldimethylsilyl)oxy)-1-(2,2,2-trifluoroethyl)piperidine (**7**)

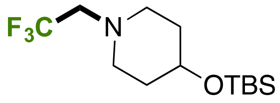

7: 80%

Title compound prepared using general trifluoroethylation method A. Purification using method A (chromatography, 3-6% EtOAc in pentane) to give a clear oil (119 mg, 80%).

^{1}H NMR (400 MHz, $CDCl_3$) δ 3.78 – 3.67 (m, 1H), 2.94 (q, *J* = 9.7 Hz, 2H), 2.88 – 2.75 (m, 2H), 2.57 – 2.41 (m, 2H), 1.85 – 1.69 (m, 2H), 1.68 – 1.50 (m, 2H), 0.88 (s, 9H), 0.04 (s, 6H); **^{13}C NMR** (101 MHz, $CDCl_3$) δ 125.6 (q, *J* = 280.3 Hz), 66.7, 58.5 (q, *J* = 30.2 Hz), 51.0, 34.5, 25.8, 18.1, -4.7; **^{19}F NMR** (376 MHz, $CDCl_3$) δ -69.13 (t, *J* = 9.7 Hz); **IR** (cm^{-1}) (neat) 2952, 2930, 2888, 2857, 1697, 1471, 1315, 1272, 1254, 1141, 1095, 1075, 1056; **HRMS** (ESI+): Exact mass calcd for $C_{13}H_{27}F_3NOSi$ [M+H], 298.1809. Found 298.1822, σ = 0.0060.

ethyl 1-(2,2,2-trifluoroethyl)piperidine-2-carboxylate (**8**)

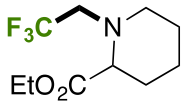

8: 83%

Title compound prepared using general trifluoroethylation method. Purification A: (chromatography, 3-6% EtOAc in pentane) to give a clear oil (100 mg, 83%).

^{1}H NMR (400 MHz, $CDCl_3$) δ 4.28 – 4.09 (m, 2H), 3.53 (app. t, *J* = 4.8 Hz, 1H), 3.25 – 3.04 (m, 3H), 2.71 (app. dt, *J* = 11.2, 4.5 Hz, 1H), 2.04 – 1.93 (m, 1H), 1.90 – 1.77 (m, 1H), 1.66 – 1.47 (m, 3H), 1.36 – 1.22 (m, 4H); **^{13}C NMR** (101 MHz, $CDCl_3$) δ 173.0, 125.7 (q, *J* = 280.0 Hz), 62.8, 60.5, 57.1 (q, *J* = 30.8 Hz), 50.4, 28.9, 25.5, 20.9, 14.3; **^{19}F NMR** (376 MHz, $CDCl_3$) δ -71.11 (t, *J* = 9.7 Hz); **IR** (cm^{-1}) (neat) 2940, 2859, 1929, 1272, 1197, 1176, 1135, 1112, 1094, 1065, 1024; **HRMS** (ESI+): Exact mass calcd for $C_{10}H_{16}F_3NNaO_2$ [M+Na], 262.1025. Found 262.1041, σ = 0.0014.

1-(2,2,2-trifluoroethyl)piperidin-4-ol (**9**)[4]

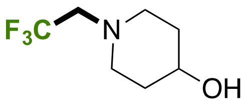

9: 70%

Title compound prepared using the general trifluoroethylation method A but with 2.5 equivalents of phenylsilane (1.25 mmol). A modified work-up procedure was used: after 4 h, the reaction was cautiously quenched whilst still hot with 1 M aqueous NaOH solution (gas released!) and allowed to stir, cooling to ambient temperature, over 1 h (to cleave any *O*-silylated species). The reaction was diluted with ether and the organic layer separated and dried with magnesium sulfate. (The majority of the desired alcohol was extracted from the hydroxide solution using this method, leaving most of the silane waste in the aqueous layer. Attempts to neutralise/acidify the aqueous layer and extract further product led to the extraction of undesired silane waste material.) The resulting oil was purified further by chromatography using a gradient of 20-70% EtOAc in pentane (10 % step rate) to give a clear oil (64 mg, 70%) that formed a low-melting waxy solid on standing.

^{1}H NMR (400 MHz, $CDCl_3$) δ 3.70 (tt, *J* = 8.6, 4.1 Hz, 1H), 2.96 (q, *J* = 9.7 Hz, 2H), 2.92 – 2.85 (m, 2H), 2.48 (ddd, *J* = 12.1, 9.7, 3.1 Hz, 2H), 1.92 – 1.84 (m, 2H), 1.75 (br. s, 1H), 1.59 (dddd, *J* = 12.9, 9.7, 8.6, 3.8 Hz, 2H); **^{13}C NMR** (126 MHz, $CDCl_3$) δ 125.5 (q, *J* = 280.4 Hz), 67.1, 58.2 (q, *J* = 30.6 Hz), 51.4, 34.3; **^{19}F NMR** (376 MHz, $CDCl_3$) δ -69.07 (t, *J* = 9.7 Hz); **IR** (cm^{-1}) (neat) 3330, 2926, 1595, 1430, 1270, 1130, 1091, 1066, 1024, 997; **HRMS** (ESI+): Exact mass calcd for $C_7H_{13}F_3NO$ [M+H], 184.0944. Found 184.0946, σ = 0.0007.

Synthesis methods for the alkylation of secondary amines using other acidic acids (compounds 43-46)

The general trifluoroethylation method used above is applicable to other acidic acids by substituting trifluoroacetic acid for another acid; trichloroacetic acid, difluoroacetic acid, dinitrobenzoic acid and chloroacetic acid have all been used successfully, with yields depending on acidity. Lower acidity acids tend to give more amide product; higher acidities give more amine. Other *alpha*-fluorinated carboxylic acids should be amenable to this protocol. For lower acidities, increasing excesses of acid (typically to two equivalents) may improve the yield slightly.

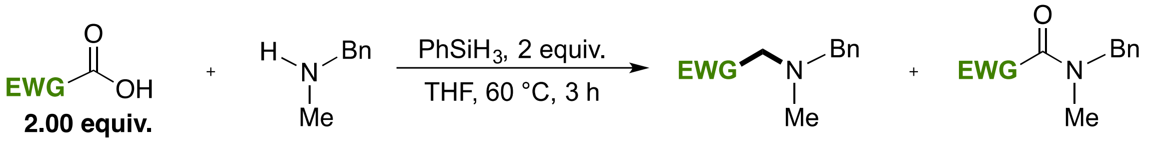

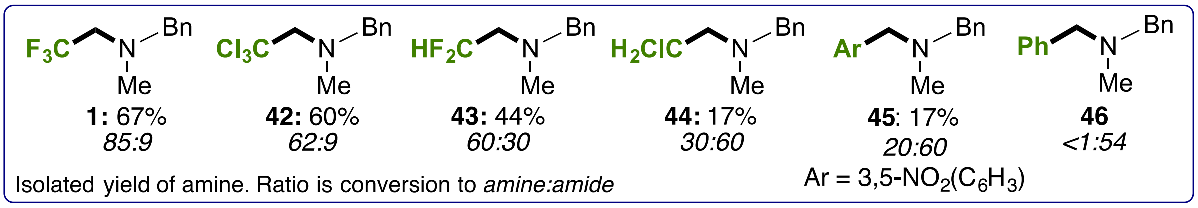

(ratios of amine:amide conversion quoted below yield for the following substrates)

N-benzyl-2,2,2-trichloro-*N*-methylethan-1-amine (**42**)[5]

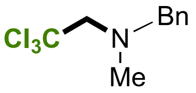

42: 60%
62:9

Title compound prepared using general trifluoroethylation method A, but with 2 equivalents of trichloroacetic acid instead of trifluoroacetic acid. Purified by flash column chromatography (3-6% EtOAc/pentane); isolated as a clear oil (72 mg, 60%).

^{1}H NMR (400 MHz, $CDCl_3$) δ 7.42 (d, *J* = 7.0 Hz, 2H), 7.39 – 7.33 (m, 2H), 7.32 – 7.26 (m, 1H), 4.00 (s, 2H), 3.55 (s, 2H), 2.55 (s, 3H); **^{13}C NMR** (101 MHz, $CDCl_3$) δ 138.8, 128.6, 128.3, 127.2, 101.3, 75.4, 63.1, 43.2; **IR** (cm^{-1}) (neat) 2904, 2847, 2797, 1495, 1454, 1348, 1021, 982, 781, 735, 696; **HRMS** (ESI+): Exact mass calcd for $C_{10}H_{13}Cl_3N$ [M+H], 252.0108. Found 252.0095, σ = 0.0252.

N-benzyl-2,2-difluoro-*N*-methylethan-1-amine (**43**)[6]

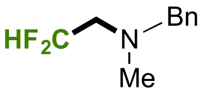

43: 44%
60:30

Title compound prepared using general trifluoroethylation method A but using two equivalents of difluoroacetic acid in place of the trifluoroacetic acid. Purification using standard work-up and chromatography (8-15% EtOAc in pentane) to give a clear oil (41 mg, 44%).

^{1}H NMR (400 MHz, $CDCl_3$) δ 7.40 – 7.24 (m, 5H), 5.84 (tt, *J* = 56.1, 4.4 Hz, 1H), 3.64 (s, 2H), 2.79 (td, *J* = 14.9, 4.4 Hz, 2H), 2.38 (s, 3H); **^{13}C NMR** (101 MHz, $CDCl_3$) δ 138.2, 128.9, 128.4, 127.3, 116.1 (t, *J* = 241.1 Hz), 62.8, 58.6 (t, *J* = 24.9 Hz), 43.3; **^{19}F NMR** (282 MHz, $CDCl_3$) δ -118.73 (dt, *J* = 56.1, 14.9 Hz);

IR (cm^{-1}) (neat) 3064, 3029, 2853, 1495, 1408, 1122, 1043, 1025; **HRMS** (ESI+): Exact mass calcd for $C_{10}H_{14}F_2N$ [M+H], 186.108. Found 186.1086, σ = 0.0015.

N-benzyl-2-chloro-*N*-methylethan-1-amine (**44**)[7]

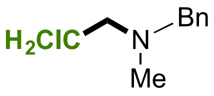

44: 17%
30:60

<u>CAUTION! Compound shares motif with extremely cytotoxic nitrogen mustard compounds.</u> Title compound prepared using general trifluoroethylation method A, but with 2 equivalents of chloroacetic acid instead of trifluoroacetic acid. Purification using standard work-up and chromatography (10-20% EtOAc in pentane) to give a clear oil (16 mg, 17%).

^{1}H NMR (400 MHz, $CDCl_3$) δ 7.44 – 7.30 (m, 5H), 3.65 – 3.57 (m, 4H), 2.80 (t, *J* = 7.0 Hz, 2H), 2.32 (s, 3H); **^{13}C NMR** (101 MHz, $CDCl_3$) δ 138.43, 128.94, 128.31, 127.18, 62.25, 58.56, 42.31, 41.61; **IR** (cm^{-1}) (neat) 2794, 1454, 1123, 1043, 1025, 739, 698; **HRMS** (ESI+): Exact mass calcd for $C_{10}H_{14}NCl$ [M+H], 184.0815. Found 184.0913, σ = 0.0078.

N-benzyl-1-(3,5-dinitrophenyl)-*N*-methylmethanamine (**45**)

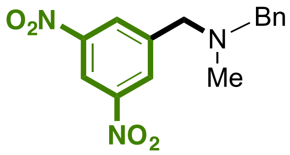

45: 17%
20:60

Title compound prepared using general trifluoroethylation method A, but with 2 equivalents of 3,5-dinitrobenzoic acid instead of trifluoroacetic acid. Purified by flash column chromatography (30-70% CH_2Cl_2/pentane); isolated as a clear oil (25 mg, 17%).

^{1}H NMR (400 MHz, $CDCl_3$) δ 8.92 (t, *J* = 2.2 Hz, 1H), 8.57 (d, *J* = 2.2 Hz, 2H), 7.43 – 7.31 (m, 4H), 7.30 – 7.27 (m, 1H), 3.70 (s, 2H), 3.64 (s, 2H), 2.27 (s, 3H); **^{13}C NMR** (68 MHz, $CDCl_3$) δ 148.5, 144.8, 138.1, 128.9, 128.9, 127.5, 126.9, 117.5, 62.2, 60.0, 42.6; **IR** (cm^{-1}) ($CDCl_3$) 3011, 2932, 1543, 1345, 909; **HRMS** (ESI+): Exact mass calcd for $C_{15}H_{16}N_3O_4$ [M+H], 302.1135. Found 302.1143, σ = 0.0006.

General procedure for the alkylative-trifluoroethylation of primary amines with aldehydes/ketones and trifluoroacetic acid

*****the above notes on general procedures also apply to these methods*****

(It is possible to run this reaction in THF if solubility is an issue. Toluene was used for dryness and its higher boiling point.)

Alkylative-trifluoroethylation Method A (typical, unhindered, aryl/vinyl aldehydes):
To an oven-dried 10 mL round-bottomed flask fitted with a water condenser under an argon atmosphere (balloon) was added the amine (0.50 mmol) and aldehyde (0.50 mmol) (which sometimes precipitated immediately as the imine). Toluene was added (0.5 mL), followed by phenylsilane (31 µL, 0.25 mmol). The reaction was stirred at **70 °C for 10 min**. Then trifluoroacetic acid (67.0 µL, 0.875 mmol) and further $PhSiH_3$ (123 µL, 1.00 mmol) were added and the reaction heated at **70 °C for 16 h**.

Alkylative-trifluoroethylation Method B (typical, unhindered, alkyl aldehydes):
To an oven-dried 10 mL round-bottomed flask fitted with a water condenser under an argon atmosphere (balloon) was added the amine (0.50 mmol) and aldehyde (0.50 mmol) (which sometimes precipitated immediately as the imine). Toluene was added (0.5 mL), followed by phenylsilane (31 µL, 0.25 mmol). The reaction was stirred at **25 °C for 30-45 min**. Then trifluoroacetic acid (67.0 µL, 0.875 mmol) and further $PhSiH_3$ (123 µL, 1.00 mmol) were added and the reaction heated at **110 °C for 4 h.**

Alkylative-trifluoroethylation Method C (hindered aldehydes):
To an oven-dried 10 mL round-bottomed flask fitted with a water condenser under an argon atmosphere (balloon) was added the amine (0.50 mmol) and aldehyde (0.50 mmol). Toluene was added (0.5 mL), followed by phenylsilane (31 µL, 0.25 mmol). The reaction was stirred at **70 °C for 30 min**. Then trifluoroacetic acid (67.0 µL, 0.875 mmol) and further $PhSiH_3$ (123 µL, 1.00 mmol) were added and the reaction heated at **70 °C for 16 h**.

Alkylative-trifluoroethylation Method D (hindered aldehydes/less reactive amines):
To an oven-dried 10 mL round-bottomed flask fitted with a water condenser under an argon atmosphere (balloon) was added the amine (0.50 mmol) and aldehyde (0.50 mmol) (which sometimes precipitated immediately as the imine). Toluene was added (0.5 mL), followed by phenylsilane (31 µL, 0.25 mmol). The reaction was stirred at **70 °C for 30 min**. Then trifluoroacetic acid (67.0 µL, 0.875 mmol) and further $PhSiH_3$ (123 µL, 1.00 mmol) were added and the reaction heated at **110 °C for 16 h**.

Alkylative-trifluoroethylation Method E (ketones):
To an oven-dried 10 mL round-bottomed flask fitted with a water condenser under an argon atmosphere (balloon) was added the amine (0.50 mmol), ketone (0.50 mmol), toluene (0.5 mL) **and trifluoroacetic acid (7 µL, 0.05 mmol)** to encourage imine formation**. After 30 min at 70 °C,** phenylsilane (31 µL, 0.25 mmol) was added and the reaction stirred at **25 °C for 10 min**. Then trifluoroacetic acid (**63.2 µL, 0.825 mmol**) and further $PhSiH_3$ (123 µL, 1.00 mmol) were added and the reaction heated at **110 °C for 4 h**.

Purification of alkylated-trifluoroethylated primary amines: For all methods, the reaction was allowed to cool, concentrated under reduced pressure, and the conversion determined using ^{1}H-NMR spectroscopy (relative to internal standard, 1,1,2,2-tetrachloroethane). The crude reaction was diluted with ether and washed with a saturated aqueous sodium bicarbonate solution, the organics dried over magnesium sulfate, and the solution concentrated to about 2 mL volume. The crude material was typically purified by flash column chromatography by dry loading the oil onto a minimum amount of silica, loading onto a silica column, and eluting from the column with ethyl acetate/pentane (ratios specified below).

Synthesis and characterization data for compounds 10-35

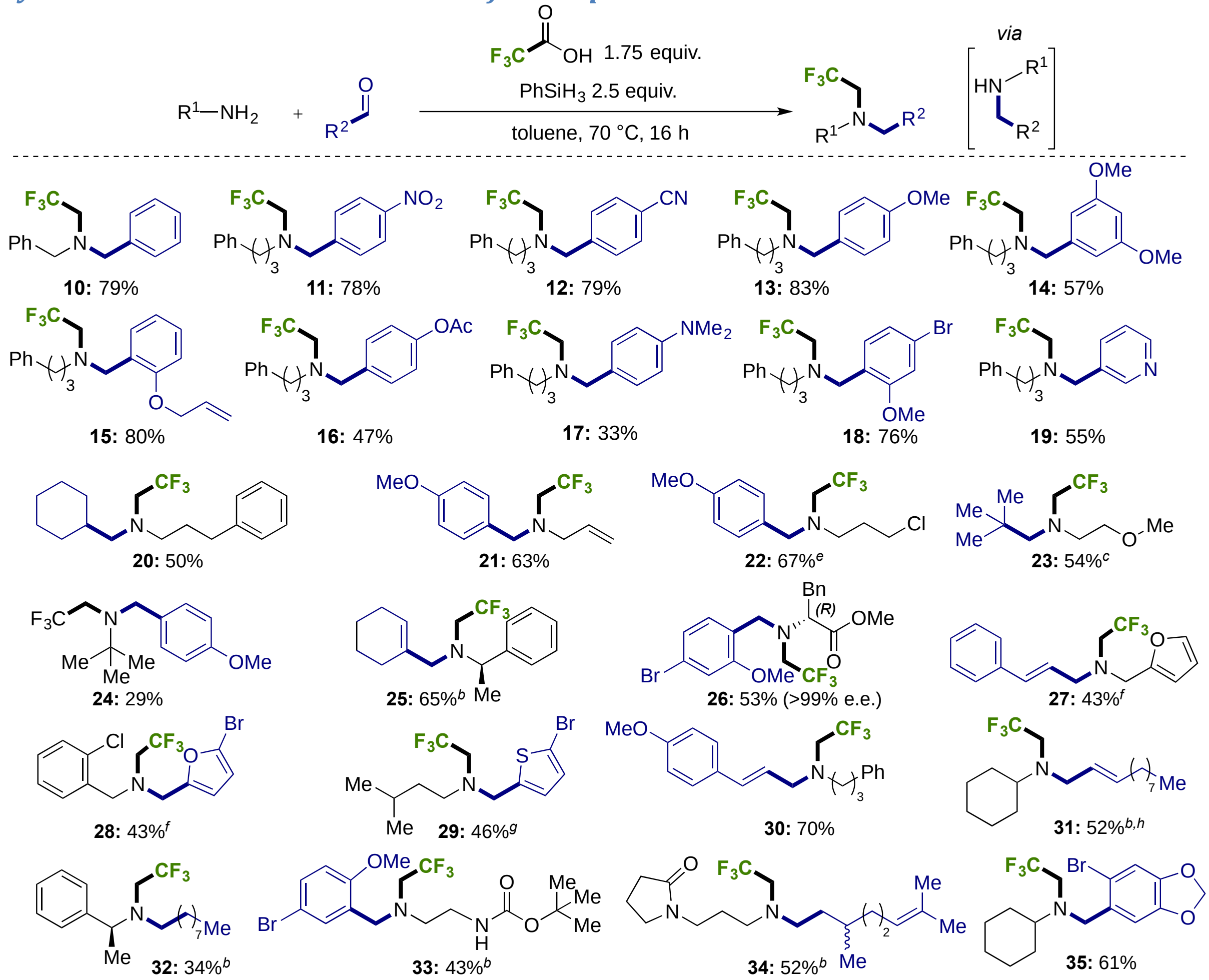

as depicted, the blue fragment with emboldened N-C bond is the aldehyde fragment and the non-bold black bond is the original amine moiety.

N,*N*-dibenzyl-2,2,2-trifluoroethan-1-amine (**10**)[8]

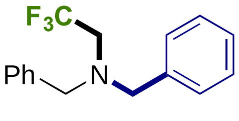

10: 79%

Title compound prepared using general alkylative-trifluoroethylation method A. Purified using standard work-up and flash column chromatography (0-10% Et_2O in pentane); isolated as a clear oil (110 mg, 79%).

^{1}H NMR (400 MHz, $CDCl_3$) δ 7.43 – 7.26 (m, 10H), 3.83 (s, 4H), 3.13 (q, *J* = 9.5 Hz, 1H); **^{13}C NMR** (101 MHz, $CDCl_3$) δ 138.3, 128.8, 128.4, 127.3, 126.1 (q, *J* = 282.7 Hz), 58.2, 52.9 (q, *J* = 30.1 Hz). **^{19}F NMR** (376 MHz, $CDCl_3$) δ -67.85 (t, *J* = 9.6 Hz); **IR** (cm^{-1}) (neat) 3064, 3030, 2840, 1602, 1496, 1307, 1178, 1134, 1084. **HRMS** (ESI+): Exact mass calcd for $C_{16}H_{17}F_3N$ [M+H], 280.1308. Found 280.1315, σ = 0.0023.

N-(4-nitrobenzyl)-3-phenyl-*N*-(2,2,2-trifluoroethyl)propan-1-amine (**11**)

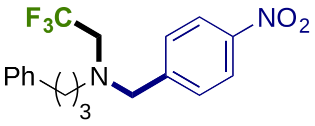

11: 78%

Title compound prepared using general alkylative-trifluoroethylation method A. Purified using standard work-up and flash column chromatography (2-8% EtOAc in pentane); isolated as a clear oil (137 mg, 78%).

^{1}H NMR (400 MHz, $CDCl_3$) 8.21 – 8.15 (m, 2H), 7.54 – 7.47 (m, 2H), 7.29 – 7.22 (m, 2H), 7.21 – 7.14 (m, 1H), 7.14 – 7.07 (m, 2H), 3.89 (s, 2H), 3.13 (q, *J* = 9.4 Hz, 2H), 2.66 (t, *J* = 7.3 Hz, 2H), 2.63 – 2.55 (m, 2H), 1.81 (m, 2H); **^{13}C NMR** (101 MHz, $CDCl_3$) δ 147.3, 146.6, 141.6, 129.1, 128.4, 128.2, 125.9, 125.7 (q, *J* = 281.5 Hz), 123.6, 58.8, 54.5 (q, *J* = 30.6 Hz), 54.0, 33.0, 29.1; **^{19}F NMR** (376 MHz, $CDCl_3$) δ -69.3 (t, *J* = 9.4 Hz); **IR** (cm^{-1}) (neat) 3027, 2940, 2855, 1603, 1519, 1344, 1269, 1136, 1090; **HR-GC-EIMS**: rt = 16.44 min. Exact mass calcd for $C_{18}H_{19}N_2O_2F_3$, 352.13931. Found 352.13767, (mass difference: -4.67 ppm).

4-(((3-phenylpropyl)(2,2,2-trifluoroethyl)amino)methyl)benzonitrile (**12**)

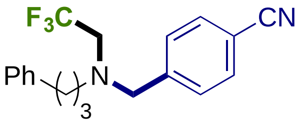

12: 79%

Title compound prepared using general alkylative-trifluoroethylation method A. Purified using standard work-up and flash column chromatography (2-10% EtOAc in pentane); isolated as a clear oil (131 mg, 79%).

^{1}H NMR (400 MHz, $CDCl_3$) 7.64 – 7.59 (m, 2H), 7.48 – 7.42 (m, 2H), 7.30 – 7.24 (m, 2H), 7.22 – 7.16 (m, 1H), 7.14 – 7.09 (m, 2H), 3.84 (s, 2H), 3.12 (q, *J* = 9.4 Hz, 2H), 2.65 (t, *J* = 7.2 Hz, 2H), 2.62 – 2.56 (m, 2H), 1.88 – 1.73 (m, 2H); **^{13}C NMR** (101 MHz, $CDCl_3$) δ 144.5, 141.6, 132.2, 129.1, 128.3, 128.2, 125.9, 125.7 (q, *J* = 281.4 Hz), 118.8, 111.1, 59.0, 54.4 (q, *J* = 30.3 Hz), 53.9, 33.0, 29.1; **^{19}F NMR** (376 MHz, $CDCl_3$) δ -69.28 (t, *J* = 9.4 Hz); **IR** (cm^{-1}) (neat) 2935, 2849, 2228 (CN), 1608, 1269, 1135, 1090; **HR-GC-EIMS**: rt = 15.05 min. Exact mass calcd for $C_{19}H_{19}N_2F_3$, 332.14948. Found 332.14814, (mass difference: -4.04 ppm).

N-(4-methoxybenzyl)-3-phenyl-*N*-(2,2,2-trifluoroethyl)propan-1-amine (**13**)

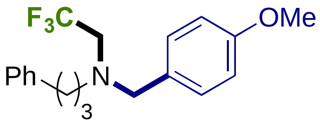

13: 83%

Title compound prepared using general alkylative-trifluoroethylation method A. Purified using standard work-up and flash column chromatography (5% EtOAc in pentane); isolated as a clear oil (141 mg, 83%).

^{1}H NMR (400 MHz, $CDCl_3$) δ 7.26 (m, 4H), 7.21 – 7.11 (m, 3H), 6.90 – 6.84 (m, 2H), 3.82 (s, 3H), 3.73 (s, 2H), 3.07 (q, *J* = 9.6 Hz, 2H), 2.66 (t, *J* = 7.0 Hz, 2H), 2.63 – 2.57 (m, 2H), 1.80 (m, 2H); **^{13}C NMR** (101 MHz, $CDCl_3$) δ 158.8, 142.2, 130.5, 130.0, 128.4, 128.3, 126.0 (q, *J* = 281.8 Hz), 125.7, 113.7, 58.4, 55.2, 53.8 (q, *J* = 30.3 Hz), 53.6, 33.1, 29.3; **^{19}F NMR** (376 MHz, $CDCl_3$) δ 69.0 (t, *J* = 9.6 Hz); **IR**

(cm^{-1}) (neat) 3062, 2937, 2836, 1612, 1511, 1269, 1245, 1132, 1090, 1076. **HRMS** (ESI+): Exact mass calcd for $C_{19}H_{23}F_3NO$ [M+H], 338.1726. Found 338.1726, σ = 0.0025.

N-(3,5-dimethoxybenzyl)-3-phenyl-*N*-(2,2,2-trifluoroethyl)propan-1-amine (**14**)

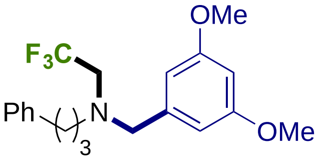

14: 57%

Title compound prepared using general alkylative-trifluoroethylation method A. Purified using standard work-up and flash column chromatography (6-11% EtOAc in pentane); isolated as a clear oil (105 mg, 57%).

^{1}**H NMR** (400 MHz, $CDCl_3$) δ 7.31 – 7.25 (m, 2H), 7.22 – 7.13 (m, 3H), 6.54 (d, *J* = 2.3 Hz, 2H), 6.40 (t, *J* = 2.3 Hz, 1H), 3.80 (s, 6H), 3.75 (s, 2H), 3.11 (q, *J* = 9.5 Hz, 2H), 2.70 (t, *J* = 7.1 Hz, 2H), 2.64 (t, *J* = 7.3 Hz, 2H), 1.82 (tt, *J* = 7.3, 7.1 Hz, 2H); ^{13}**C NMR** (101 MHz, $CDCl_3$) δ 160.8, 142.1, 141.2, 128.3 (2C), 126.0 (q, *J* = 281.6 Hz), 125.7, 106.4, 99.2, 59.3, 55.2, 54.1 (q, *J* = 31.4 Hz), 53.9, 33.2, 29.4; ^{19}**F NMR** (376 MHz, $CDCl_3$) δ-68.97 (t, *J* = 9.5 Hz); **IR** (cm^{-1}) (neat) 2939, 2839, 1596, 1456, 1269, 1204, 1135, 1091, 1056; **HRMS** (ESI+): Exact mass calcd for $C_{20}H_{25}F_3NO_2$ [M+H], 368.1832. Found 368.1830, σ = 0.0006.

N-(2-(allyloxy)benzyl)-3-phenyl-*N*-(2,2,2-trifluoroethyl)propan-1-amine (**15**)

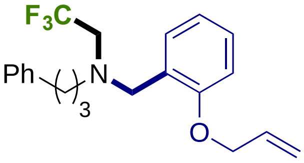

15: 80%

Title compound prepared using general alkylative-trifluoroethylation method A. Purified using standard work-up and flash column chromatography (3-5% EtOAc in pentane); isolated as a clear oil (145 mg, 80%).

^{1}**H NMR** (400 MHz, $CDCl_3$) δ 7.41 (dd, *J* = 7.5, 1.7 Hz, 1H), 7.32 – 7.12 (m, 6H), 6.97 (t, *J* = 7.5 Hz, 1H), 6.88 (d, *J* = 8.2 Hz, 1H), 6.07 (ddt, *J* = 17.3, 10.5, 5.2 Hz, 1H), 5.42 (ddt, *J* = 17.3, 1.7, 1.7 Hz, 1H), 5.29 (ddt, *J* = 10.5, 1.7, 1.3 Hz, 1H), 4.56 (ddd, *J* = 5.2, 1.7, 1.3 Hz, 2H), 3.91 (s, 2H), 3.16 (q, *J* = 9.6 Hz, 2H), 2.71 (t, *J* = 7.1 Hz, 2H), 2.63 (t, *J* = 7.6 Hz, 2H), 1.83 (tt, *J* = 7.6, 7.1 Hz, 2H); ^{13}**C NMR** (101 MHz, $CDCl_3$) δ 156.7, 142.3, 133.4, 130.4, 128.4, 128.3, 128.1, 127.0, 126.0 (q, *J* = 282.2, 281.2 Hz), 125.7, 120.6, 117.3, 111.7, 68.9, 54.6 (q, *J* = 30.2 Hz), 54.2, 52.8, 33.2, 29.5; ^{19}**F NMR** (376 MHz, $CDCl_3$) δ -69.47 (t, *J* = 9.6 Hz); **IR** (cm^{-1}) (neat) 3027, 2933, 2861, 1601, 1588, 1492, 1453, 1270, 1133, 1090; **HRMS** (ESI+): Exact mass calcd for $C_{21}H_{24}F_3NNaO$ [M+Na], 386.1702. Found 386.1702, σ = 0.0050.

4-(((3-phenylpropyl)(2,2,2-trifluoroethyl)amino)methyl)phenyl acetate (**16**)

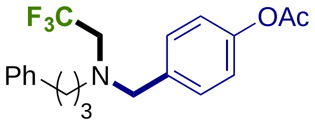

16: 47%

Title compound prepared using general alkylative-trifluoroethylation method A. Purified using standard work-up and flash column chromatography (6-12% EtOAc in pentane); isolated as a clear oil (85 mg, 47%).

^{1}H NMR (400 MHz, $CDCl_3$) δ 7.37 – 7.31 (m, 2H), 7.30 – 7.23 (m, 2H), 7.21 – 7.10 (m, 3H), 7.08 – 7.01 (m, 2H), 3.78 (s, 2H), 3.09 (q, *J* = 9.5 Hz, 2H), 2.67 (t, *J* = 7.1 Hz, 2H), 2.60 (t, *J* = 7.6 Hz, 2H), 2.31 (s, 3H), 1.80 (tt, *J* = 7.6, 7.1 Hz, 2H); **^{13}C NMR** (101 MHz, $CDCl_3$) δ 169.5, 149.8, 142.0, 136.2, 129.6, 128.3, 125.9 (q, *J* = 281.9 Hz), 125.8, 121.4, 58.5, 54.0 (q, *J* = 30.2 Hz), 53.7, 33.1, 29.3, 21.1; **^{19}F NMR** (376 MHz, $CDCl_3$) δ -69.05 (t, *J* = 9.5 Hz); **IR** (cm^{-1}) (neat) 2936, 2850, 1761, 1506, 1369, 1269, 1213, 1191, 1134, 1089, 1077; **HRMS** (ESI+): Exact mass calcd for $C_{20}H_{22}F_3NNaO_2$ [M+Na], 388.1495. Found 388.1503, σ = 0.0003.

N,*N*-dimethyl-4-(((3-phenylpropyl)(2,2,2-trifluoroethyl)amino)methyl)aniline (**17**)

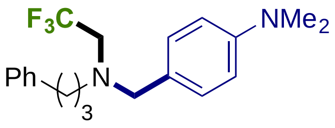

17: 33%

Title compound prepared using general alkylative-trifluoroethylation method A with the following modifications: Initially, the amine and aldehyde were stirred in toluene for 10 min at 110 °C. The trifluoroacetic acid and phenylsilane were then added at 25 °C and the reaction heated for 4 h at reflux. Purified using standard work-up and flash column chromatography (5-9% EtOAc in pentane); isolated as a clear oil (57 mg, 33%).

^{1}H NMR (400 MHz, $CDCl_3$) δ 7.30 – 7.22 (m, 2H), 7.21 – 7.11 (m, 5H), 6.77 – 6.66 (m, 2H), 3.69 (s, 2H), 3.06 (q, *J* = 9.6 Hz, 2H), 2.95 (s, 6H), 2.66 (t, *J* = 7.0 Hz, 2H), 2.61 (t, *J* = 7.7 Hz, 2H), 1.80 (tt, *J* = 7.7, 7.0 Hz, 2H); **^{13}C NMR** (101 MHz, $CDCl_3$) δ 150.0, 142.3, 129.8, 128.4, 128.3, 126.1 (q, *J* = 283.2 Hz), 126.1, 125.7, 112.5, 58.4, 53.6 (q, *J* = 30.0 Hz), 53.6, 40.7, 33.2, 29.4; **^{19}F NMR** (376 MHz, $CDCl_3$) δ -68.92 (t, *J* = 9.6 Hz); **IR** (cm^{-1}) (neat) 2935, 2852,2805, 1614, 1521, 1269, 1131, 1089, 1075; **HRMS** (ESI+): Exact mass calcd for $C_{20}H_{26}F_3N_2$ [M+H], 351.2043. Found 351.2047, σ = 0.0008.

N-(5-bromo-2-methoxybenzyl)-3-phenyl-*N*-(2,2,2-trifluoroethyl)propan-1-amine (**18**)

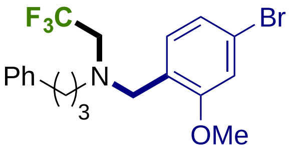

18: 76%

Title compound prepared using general alkylative-trifluoroethylation method A. Purified using standard work-up and flash column chromatography (3-7% EtOAc in pentane); isolated as a clear oil (159 mg, 76%).

^{1}H NMR (400 MHz, $CDCl_3$) δ 7.52 (d, *J* = 2.6 Hz, 1H), 7.34 (dd, *J* = 8.7, 2.6 Hz, 1H), 7.31 – 7.24 (m, 2H), 7.22 – 7.12 (m, 3H), 6.74 (d, *J* = 8.7 Hz, 1H), 3.81 (s, 2H), 3.79 (s, 3H), 3.14 (q, *J* = 9.5 Hz, 2H), 2.68 (t, *J* = 7.2 Hz, 2H), 2.65 – 2.59 (m, 2H), 1.88 – 1.74 (m, 2H); **^{13}C NMR** (101 MHz, $CDCl_3$) δ 156.7, 142.1, 132.5, 130.7, 128.3 (2C), 125.9 (q, *J* = 281.5 Hz), 125.7, 112.9, 112.0, 55.5, 54.6 (q, *J* = 30.5 Hz), 54.1, 52.5, 33.1, 29.3; **^{19}F NMR** (376 MHz, $CDCl_3$) δ -69.41 (t, *J* = 9.5 Hz); **IR** (cm^{-1}) (neat) 2939, 1486, 1269, 1249, 1134, 1090, 1030; **HRMS** (ESI+): Exact mass calcd for $C_{19}H_{22}BrF_3NO$ [M+H], 416.0831. Found 416.0831, σ = 0.0086.

3-phenyl-*N*-(pyridin-3-ylmethyl)-*N*-(2,2,2-trifluoroethyl)propan-1-amine (**19**)

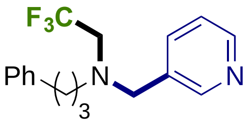

19: 55%

Title compound prepared using general alkylative-trifluoroethylation method A but with 2.75 equiv. TFA. Purified using standard work-up and flash column chromatography (20-30% EtOAc in pentane with 1% Et_3N throughout); isolated as a clear oil (85 mg, 55%).

^{1}H NMR (400 MHz, $CDCl_3$) δ 8.59 – 8.46 (m, 2H), 7.74 – 7.61 (m, 1H), 7.29 – 7.21 (m, 3H), 7.19 – 7.13 (m, 1H), 7.13 – 7.06 (m, 2H), 3.79 (s, 2H), 3.09 (q, *J* = 9.4 Hz, 2H), 2.65 (t, *J* = 7.1 Hz, 2H), 2.57 (t, *J* = 7.8 Hz, 2H), 1.79 (tt, *J* = 7.8, 7.1 Hz, 2H); **^{13}C NMR** (101 MHz, $CDCl_3$) δ 150.0, 148.9, 141.8, 136.4, 134.1, 128.3, 128.3, 125.8, 125.8 (q, *J* = 281.7, 280.8 Hz), 123.4, 56.6, 54.2 (q, *J* = 30.4 Hz), 53.8, 33.0, 29.2; **^{19}F NMR** (376 MHz, $CDCl_3$) δ -69.28 (t, *J* = 9.4 Hz); **IR** (cm^{-1}) (neat) 3028, 2940, 2858, 1577, 1425, 1269, 1136, 1077; **HRMS** (ESI+): Exact mass calcd for $C_{17}H_{19}F_3N_2Na$ [M+Na], 331.1393. Found 331.1407, σ = 0.0766.

N-(cyclohexylmethyl)-3-phenyl-*N*-(2,2,2-trifluoroethyl)propan-1-amine (**20**)

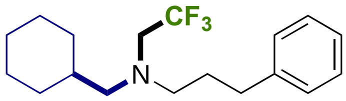

20: 50%

Title compound prepared using general alkylative-trifluoroethylation method A. Purified using standard work-up and flash column chromatography (3-6% EtOAc in pentane); isolated as a clear oil (79 mg, 50%).

^{1}H NMR (400 MHz, $CDCl_3$) δ 7.33 – 7.25 (m, 2H), 7.23 – 7.16 (m, 3H), 3.01 (q, *J* = 9.6 Hz, 2H), 2.68 – 2.57 (m, 4H), 2.37 (d, *J* = 7.1 Hz, 2H), 1.86 – 1.62 (m, 7H), 1.38 (ttt, *J* = 10.8, 7.1, 3.4 Hz, 1H), 1.30 – 1.09 (m, 3H), 0.93 – 0.76 (m, 2H); **^{13}C NMR** (101 MHz, $CDCl_3$) δ 142.3, 128.4, 128.3, 126.0 (q, *J* = 281.0 Hz), 125.7, 62.5, 55.8 (q, *J* = 30.1 Hz), 55.1, 36.4, 33.2, 31.4, 29.5, 26.8, 26.1; **^{19}F NMR** (376 MHz, $CDCl_3$) δ -69.79 (t, *J* = 9.6 Hz); **IR** (cm^{-1}) (neat) 2922, 2851, 1451, 1270, 1135, 1093; **HRMS** (ESI+): Exact mass calcd for $C_{18}H_{27}F_3N$ [M+H], 314.2090. Found 314.2094, σ = 0.0028.

N-(4-methoxybenzyl)-*N*-(2,2,2-trifluoroethyl)prop-2-en-1-amine (**21**)

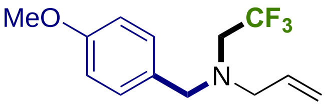

21: 63%

Title compound prepared using general alkylative-trifluoroethylation method A. Purified using standard work-up and flash column chromatography (1-2% EtOAc in pentane); isolated as a clear oil (82 mg, 63%).

^{1}H NMR (400 MHz, $CDCl_3$) δ 7.31 – 7.21 (m, 2H), 6.93 – 6.82 (m, 2H), 5.84 (ddt, *J* = 16.8, 10.2, 6.5 Hz, 1H), 5.25 – 5.15 (m, 2H), 3.81 (s, 3H), 3.74 (s, 2H), 3.24 (d, *J* = 6.5 Hz, 2H), 3.08 (q, *J* = 9.6 Hz, 2H); **^{13}C NMR** (101 MHz, $CDCl_3$) δ 158.9, 134.9, 130.3, 130.0, 126.0 (q, *J* = 281.4 Hz), 118.3, 113.7, 57.8, 56.8, 55.2, 52.8 (q, *J* = 30.2 Hz); **^{19}F NMR** (376 MHz, $CDCl_3$) δ -68.95 (t, *J* = 9.6 Hz); **IR** (cm^{-1}) (neat) 3005, 2937, 2906, 2837, 1612, 1511, 1244, 1136, 1074; **HR-GC-EIMS**: rt = 11.25 min. Exact mass calcd for $C_{13}H_{16}NOF_3$, 259.11785. Found 259.11854, (mass difference: 2.65 ppm).

3-chloro-*N*-(4-methoxybenzyl)-*N*-(2,2,2-trifluoroethyl)propan-1-amine (**22**)

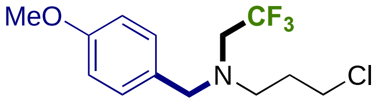

22: 67%

Title compound prepared using general alkylative-trifluoroethylation method A using the HCl salt of the amine. (No additional base was added in this case, but should be added for highly crystalline/poorly soluble salts (e.g. triethylamine, 1 equiv.)). Purified using standard work-up and flash column chromatography (1.0-2.5% EtOAc in pentane); isolated as a clear oil (70 mg, 47%). The reaction was also performed on a 5.00 mmol scale (see scale up safety notes in Supplementary Methods; notes on general procedures), providing the title compound as a clear oil (970 mg, 67%).

^{1}H NMR (400 MHz, $CDCl_3$) 7.24 – 7.19 (m, 2H), 6.90 – 6.83 (m, 2H), 3.81 (s, 3H), 3.73 (s, 2H), 3.56 (t, *J* = 6.6 Hz, 2H), 3.07 (q, *J* = 9.5 Hz, 2H), 2.79 (t, *J* = 6.6 Hz, 2H), 1.91 (tt, *J* = 6.6, 6.6 Hz, 2H); **^{13}C NMR** (101 MHz, $CDCl_3$) δ 158.9, 130.1, 129.9, 125.9 (q, *J* = 281.8 Hz), 113.8, 58.5, 55.2, 54.0 (q, *J* = 30.3 Hz), 51.3, 42.5, 30.8; **^{19}F NMR** (376 MHz, $CDCl_3$) δ -69.1 (t, *J* = 9.5 Hz); **IR** (cm^{-1}) (neat) 2960, 2838, 1612, 1586, 1511, 1268, 1244, 1136, 1084, 1034, 974, 812; **HR-GC-EIMS**: rt = 12.24 min. Exact mass calcd for $C_{13}H_{17}NOF_3Cl$, 295.09453. Found 295.09467, (mass difference: 0.50 ppm).

N-(2-methoxyethyl)-2,2-dimethyl-*N*-(2,2,2-trifluoroethyl)propan-1-amine (**23**)

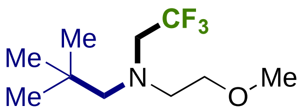

23: 54%

Title compound prepared using general alkylative-trifluoroethylation method C. Purified using standard work-up and flash column chromatography (0-1% EtOAc in pentane); isolated as a clear oil (61 mg, 54%). Compound is volatile.

^{1}H NMR (400 MHz, $CDCl_3$) δ 3.49 (t, *J* = 6.2 Hz, 2H), 3.34 (s, 3H), 3.20 (q, *J* = 9.5 Hz, 2H), 2.87 (t, *J* = 6.2 Hz, 2H), 2.44 (s, 2H), 0.88 (s, 9H); **^{13}C NMR** (101 MHz, $CDCl_3$) δ 126.0 (q, *J* = 282.9 Hz), 71.5, 68.8, 58.8, 57.8 (q, *J* = 29.7 Hz), 56.1, 33.3, 27.8; **^{19}F NMR** (282 MHz, $CDCl_3$) δ -74.1 (t, *J* = 9.5 Hz); **IR** (cm^{-1}) (neat) 2954, 2870, 1272, 1135, 1093; **HR-GC-EIMS**: rt = 9.34 min. Exact mass calcd for $C_{10}H_{20}NOF_3$, 227.1492. Found 227.1489, (mass difference: -0.94 ppm).

N-(4-methoxybenzyl)-2-methyl-*N*-(2,2,2-trifluoroethyl)propan-2-amine (**24**)

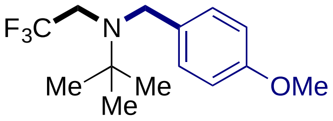

24: 29%

Title compound prepared using general alkylative-trifluoroethylation method A. Purified using standard work-up and flash column chromatography (3-7% EtOAc in pentane); isolated as a clear oil (40 mg, 29%).

^{1}H NMR (400 MHz, $CDCl_3$) δ 7.33 – 7.23 (m, 2H), 6.88 – 6.78 (m, 2H), 3.86 (s, 2H), 3.80 (s, 3H), 3.18 (q, *J* = 9.5 Hz, 2H), 1.12 (s, 9H); **^{13}C NMR** (101 MHz, $CDCl_3$) δ 158.2, 134.0, 128.4, 126.0 (q, *J* = 280.5 Hz), 113.4, 55.6, 55.2, 54.9, 51.9 (q, *J* = 31.1 Hz), 27.5; **^{19}F NMR** (376 MHz, $CDCl_3$) δ -69.90 (t, *J* = 9.5 Hz); **IR** (cm^{-1}) (neat) 2971, 1511, 1242, 1129, 1047; **HRMS** (ESI+): Exact mass calcd for $C_{14}H_{21}F_3NO$ [M+H], 276.1570. Found 276.1580, σ = 0.0084.

(*R*)-*N*-(cyclohex-1-en-1-ylmethyl)-2,2,2-trifluoro-*N*-(1-phenylethyl)ethan-1-amine (**25**)

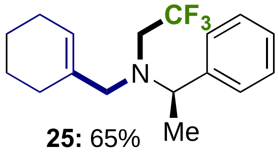

25: 65%

Title compound prepared using general alkylative-trifluoroethylation method B. Purified using standard work-up and flash column chromatography (2-10% EtOAc in pentane); isolated as a clear oil (97 mg, 65%).

^{1}H NMR (400 MHz, $CDCl_3$) 7.35 – 7.27 (m, 4H), 7.24 – 7.19 (m, 1H), 5.58 – 5.50 (m, 1H), 4.03 (q, *J* = 6.9 Hz, 1H), 3.12 – 2.84 (m, 4H), 2.08 – 1.92 (m, 3H), 1.90 – 1.78 (m, 1H), 1.61 – 1.47 (m, 4H), 1.35 (d, *J* = 6.9 Hz, 3H); **^{13}C NMR** (101 MHz, $CDCl_3$) δ 142.4, 135.8, 128.1, 127.9, 126.9, 126.1 (q, *J* = 280.3 Hz), 125.4, 58.1, 58.0, 50.2 (q, *J* = 30.8 Hz), 26.3, 25.3, 22.7, 22.6, 14.6; **^{19}F NMR** (282 MHz, $CDCl_3$) δ -69.56 (t, *J* = 9.5 Hz); **IR** (cm^{-1}) (neat) 2929, 2837, 1494, 1449, 1269, 1135, 1097 **HRMS** (ESI+): Exact mass calcd for $C_{17}H_{23}F_3N$ [M+H], 298.1777. Found 298.1763, σ = 0.0581; $[\alpha]_D^{26}$ +22.2°(*c* 1.0, EtOH).

methyl *N*-(4-bromo-2-methoxybenzyl)-*N*-(2,2,2-trifluoroethyl)-*D*-phenylalaninate (**26**)

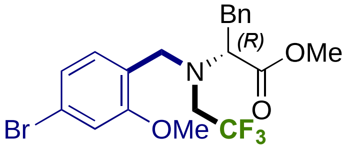

26: 53% (>99% e.e.)

Title compound prepared using general alkylative-trifluoroethylation method A using enantiopure *D*-phenylalanine methyl ester. Purified using standard work-up and flash column chromatography (10-15% EtOAc in pentane); isolated as a clear oil (121 mg, 53%). The ee of the material was determined by HPLC to be >99% by comparison to the (*S*-) enantiomer (see HPLC data at Supplementary Figure 9).

^{1}H NMR (400 MHz, $CDCl_3$) δ 7.36 – 7.17 (m, 5H), 7.13 – 7.06 (m, 2H), 6.69 (d, *J* = 8.5 Hz, 1H), 4.01 (d, *J* = 15.3 Hz, 1H), 3.86 (d, *J* = 15.3 Hz, 1H), 3.75 (s, 3H), 3.69 (dd, *J* = 7.8, 7.0 Hz, 1H), 3.65 (s, 3H), 3.53 – 3.31 (m, 2H), 3.09 (dd, *J* = 13.9, 7.8 Hz, 1H), 2.94 (dd, *J* = 13.9, 7.0 Hz, 1H); **^{13}C NMR** (101 MHz, $CDCl_3$) δ 173.0, 156.6, 137.4, 132.4, 130.9, 128.9, 128.5, 128.4, 126.6, 125.4 (q, *J* = 279.8 Hz), 112.9, 111.8,

65.0, 55.4, 52.1 (q, J = 31.6 Hz), 51.5, 51.2, 36.6; **^{19}F NMR** (376 MHz, $CDCl_3$) δ -70.72 (t, J = 9.3 Hz); **IR** (cm^{-1}) (neat) 2952, 1732, 1487, 1271, 1244, 1138, 1091, 1029; **HRMS** (ESI+): Exact mass calcd for $C_{20}H_{21}BrF_3NNaO_3$ [M+Na], 482.0549. Found 482.0550, σ = 0.0059; $[\alpha]_D^{26}$ +5.2° (c 1.0, EtOH).

methyl *N*-(4-bromo-2-methoxybenzyl)-*N*-(2,2,2-trifluoroethyl)-*L*-phenylalaninate (***ent*-26**)

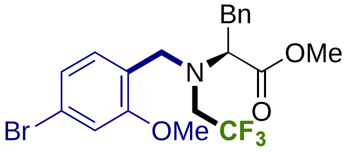

***ent*-26**

Title compound prepared using general alkylative-trifluoroethylation method A. Purified using standard work-up and flash column chromatography (10-15% EtOAc in pentane); isolated as a clear oil (104 mg, 45%). The ee of the material was determined by HPLC to be >99% by comparison to the (*R*-) enantiomer (see HPLC data at Supplementary Figure 9). Identical data to the other enantiomer, except: $[\alpha]_D^{26}$ -5.2°(c 1.0, EtOH).

(*E*)-*N*-(furan-2-ylmethyl)-3-phenyl-*N*-(2,2,2-trifluoroethyl)prop-2-en-1-amine (**27**)

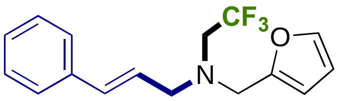

27: 43%

Title compound prepared using general alkylative-trifluoroethylation method A, but using 3 equivalents of trifluoroacetic acid. Purified using standard work-up and flash column chromatography (1-2% EtOAc in pentane); isolated as a clear oil (64 mg, 43%).

^{1}H NMR (400 MHz, $CDCl_3$) δ 7.45 – 7.15 (m, 6H), 6.56 (d, J = 15.8 Hz, 1H), 6.36 (dd, J = 3.2, 1.9 Hz, 1H), 6.23 (dt, J = 15.8, 6.7 Hz, 1H), 6.26 – 6.24 (m, 1H) 3.90 (s, 2H), 3.46 (d, J = 6.7, Hz, 2H), 3.17 (q, J = 9.5 Hz, 2H); **^{13}C NMR** (101 MHz, $CDCl_3$) δ 151.5, 142.5, 136.7, 133.5, 128.6, 127.7, 126.4 (2C), 125.8 (q, J = 280.3 Hz), 110.1, 109.3, 57.0, 52.7 (q, J = 30.8 Hz), 50.3; **^{19}F NMR** (376 MHz, $CDCl_3$) δ -69.63 (t, J = 9.5 Hz); **IR** (cm^{-1}) (neat) 3028, 2932, 2839, 1599, 1497, 1269, 1138, 1068; **HR-GC-EIMS**: rt = 12.49 min. Exact mass calcd for $C_{16}H_{16}NOF_3$, 295.11785. Found 295.11764, (mass difference: -0.70 ppm).

N-((5-bromofuran-2-yl)methyl)-*N*-(2-chlorobenzyl)-2,2,2-trifluoroethan-1-amine (**28**)

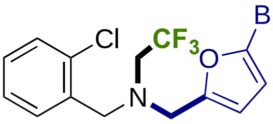

28: 43%

Title compound prepared using general alkylative-trifluoroethylation method A, but using 3 equivalents of trifluoroacetic acid. Purified using standard work-up and flash column chromatography (3-6% CH_2Cl_2 in pentane); isolated as a clear oil (83 mg, 43%).

^{1}H NMR (400 MHz, $CDCl_3$) 7.57 (dd, J = 7.7, 1.8 Hz, 1H), 7.35 (dd, J = 7.7, 1.4 Hz, 1H), 7.32 – 7.23 (m, 1H), 7.21 (td, J = 7.7, 1.8 Hz, 1H), 6.25 (d, J = 3.3 Hz, 1H), 6.21 (d, J = 3.3 Hz, 1H), 3.97 (s, 2H), 3.81 (s, 2H), 3.22 (q, J = 9.4 Hz, 2H); **^{13}C NMR** (101 MHz, $CDCl_3$) δ 153.4, 135.6, 133.9, 130.2, 129.5, 128.5, 126.9, 124.3 (q, J = 281.0 Hz), 112.1, 111.8, 55.3, 53.7 (q, J = 31.0 Hz), 50.0; **^{19}F NMR** (376 MHz,

CDCl$_3$) δ -69.4 (t, *J* = 9.4 Hz); **IR** (cm^{-1}) (neat) 3067, 2923, 1501, 1443, 1269, 1139, 1077; **HR-GC-EIMS**: rt = 12.95 min. Exact mass calcd for $C_{14}H_{12}NOF_3ClBr$, 380.97374. Found 380.97266, (mass difference: 2.65 ppm).

N-((5-bromothiophen-2-yl)methyl)-3-methyl-*N*-(2,2,2-trifluoroethyl)butan-1-amine (**29**)

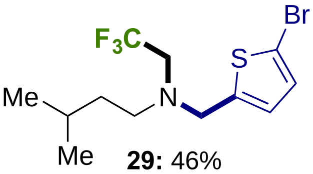

29: 46%

Title compound prepared using general alkylative-trifluoroethylation method A, but using 2 equivalents of trifluoroacetic acid. Purified using standard work-up and flash column chromatography (0-4% CH_2Cl_2 in pentane); isolated as a clear oil (79 mg, 46%).

^{1}H NMR (400 MHz, CDCl$_3$) δ 6.89 (d, *J* = 3.7 Hz, 1H), 6.68 (d, *J* = 3.7 Hz, 1H), 3.92 (s, 2H), 3.09 (q, *J* = 9.5 Hz, 2H), 2.71 – 2.62 (m, 2H), 1.61 (dh, *J* = 6.9, 6.6 Hz, 1H), 1.43 – 1.33 (m, 2H), 0.88 (d, *J* = 6.6 Hz, 6H); **^{13}C NMR** (126 MHz, CDCl$_3$) δ 143.8, 129.2, 126.2, 125.8 (q, *J* = 281.8 Hz), 111.6, 53.6, 53.4 (q, *J* = 30.5 Hz), 52.2, 36.3, 25.8, 22.6; **^{19}F NMR** (376 MHz, CDCl$_3$) δ -69.06 (t, *J* = 9.5 Hz); **IR** (cm^{-1}) (neat) 2957, 2929, 2870, 1467, 1268, 1139, 1076; **HR-GC-EIMS**: rt = 11.85 min. Exact mass calcd for $C_{12}H_{17}NF_3SBr$, 343.0212. Found 343.0203, (mass difference: -2.42 ppm).

(*E*)-3-(4-methoxyphenyl)-*N*-(3-phenylpropyl)-*N*-(2,2,2-trifluoroethyl)prop-2-en-1-amine (**30**)

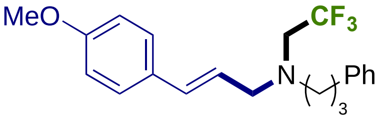

30: 70%

Title compound prepared using general alkylative-trifluoroethylation method A. Purified using standard work-up and flash column chromatography (3-6% EtOAc in pentane); isolated as a clear oil (127 mg, 70%).

^{1}H NMR (400 MHz, CDCl$_3$) δ 7.35 – 7.24 (m, 4H), 7.20 (m, 3H), 6.91 – 6.85 (m, 2H), 6.47 (d, *J* = 15.9 Hz, 1H), 6.08 (dt, *J* = 15.9, 6.8 Hz, 1H), 3.83 (s, 3H), 3.41 (d, *J* = 6.8 Hz, 2H), 3.10 (q, *J* = 9.6 Hz, 2H), 2.72 (t, *J* = 7.2 Hz, 2H), 2.67 (t, *J* = 7.7 Hz, 2H), 1.83 (tt, *J* = 7.7, 7.2 Hz, 2H); **^{13}C NMR** (101 MHz, CDCl$_3$) δ 159.2, 142.1, 132.7, 129.6, 128.4, 128.3, 127.5, 125.9 (q, *J* = 280.7 Hz), 125.8, 124.0, 114.0, 57.4, 55.3, 54.2, 53.9 (q, *J* = 30.4 Hz), 33.1, 29.3; **^{19}F NMR** (376 MHz, CDCl$_3$) δ -69.69 (t, *J* = 9.6 Hz); **IR** (cm^{-1}) (neat) 2936, 2837, 1607, 1510, 1269, 1300, 1133, 1091, 1033; **HRMS** (ESI+): Exact mass calcd for $C_{21}H_{24}F_3NNaO$ [M+Na], 386.1702. Found 386.1702, σ = 0.0185.

(E)-*N*-(2,2,2-trifluoroethyl)-*N*-(undec-2-en-1-yl)cyclohexanamine (**31**)

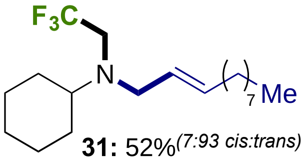

31: 52%*(7:93 cis:trans)*

Title compound prepared using general alkylative-trifluoroethylation method B. Purified using standard work-up and flash column chromatography (1-2% EtOAc in pentane); isolated as a clear oil (87 mg, 52%) as a 7:93 ratio of cis:trans isomers, as determined from integration of the pair of

allylamine methylene doublets. Attempts to reduce isomerisation by altering the times and temperatures of each part of the protocol proved unsuccessful.

^{1}H NMR (400 MHz, $CDCl_3$) δ 5.60 – 5.48 (dt, *J* = 15.3, 6.8 Hz, 1H), 5.46 – 5.36 (dt, *J* = 15.3, 6.4 Hz, 1H), 3.30 (d, *J* = 6.7 Hz, 0.14H, NCH_2CH=CH - cis), 3.21 (d, *J* = 6.4 Hz, 1.86H, NCH_2CH=CH - trans), 3.00 (q, *J* = 9.5 Hz, 2H), 2.56 (tt, *J* = 11.3, 3.2 Hz, 1H), 2.02 (dt, *J* = 7.0, 6.8 Hz, 2H), 1.84 – 1.73 (m, 4H), 1.66 – 1.58 (m, 1H), 1.42 – 0.98 (m, 17H), 0.88 (t, *J* = 6.8 Hz, 3H); **^{13}C NMR** (101 MHz, $CDCl_3$) *(trans only)* δ 133.9, 127.8, 126.0 (q, *J* = 279.7 Hz), 60.4, 54.2, 50.6 (q, *J* = 30.8 Hz), 32.3, 31.9, 29.5, 29.5, 29.3, 29.3, 29.1, 26.1, 26.0 (d, *J* = 1.3 Hz), 22.7, 14.1; **^{19}F NMR** (376 MHz, $CDCl_3$) δ -71.23 (t, *J* = 9.5 Hz); **IR** (cm^{-1}) (neat) 2925, 2854, 1452, 1272, 1135, 1094, 1067; **HR-GC-EIMS**: rt = 13.09 min. Exact mass calcd for $C_{19}H_{34}NF_3$, 333.2638. Found 333.2640, (mass difference: 0.69 ppm).

(*S*)-*N*-(1-phenylethyl)-*N*-(2,2,2-trifluoroethyl)nonan-1-amine (**32**)

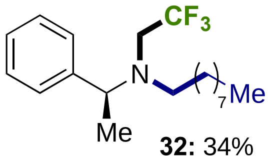

32: 34%

Title compound prepared using general alkylative-trifluoroethylation method B. Purified using standard work-up and flash column chromatography (0.5-2% EtOAc in pentane); isolated as a clear oil (55 mg, 34%).

^{1}H NMR (400 MHz, $CDCl_3$) δ 7.43 – 7.32 (m, 4H), 7.32 – 7.25 (m, 1H), 4.04 (q, *J* = 6.8 Hz, 1H), 3.16 (dq, *J* = 15.5, 9.5 Hz, 1H), 3.02 (dq, *J* = 15.5, 9.5 Hz, 1H), 2.61 (dt, *J* = 13.2, 7.2 Hz, 1H), 2.51 (dt, *J* = 13.2, 7.4 Hz, 1H), 1.52 – 1.43 (m, 2H), 1.41 (d, *J* = 6.8 Hz, 3H), 1.37 – 1.16 (m, 12H), 0.92 (t, *J* = 6.9 Hz, 3H); **^{13}C NMR** (101 MHz, $CDCl_3$) δ 143.0, 128.1, 127.7, 127.0, 126.0 (q, *J* = 279.9 Hz), 59.7, 51.7 (q, *J* = 31.2 Hz), 51.0, 31.9, 29.6, 29.4, 29.3, 28.0, 26.8, 22.7, 15.9, 14.1; **^{19}F NMR** (376 MHz, $CDCl_3$) δ -70.2 (t, *J* = 9.5 Hz); **IR** (cm^{-1}) (neat) 2925, 2855, 1270, 1137, 1095; **HR-GC-EIMS**: rt = 12.78. Exact mass calcd for $C_{19}H_{30}NF_3$ [M+H], 329.2325. Found 329.2315, (mass difference: -2.88 ppm); $[\alpha]_D^{26}$ +15.6° (*c* 1.0, EtOH).

tert-butyl (6-((5-bromo-2-methoxybenzyl)(2,2,2-trifluoroethyl)amino)hexyl)carbamate (**33**)

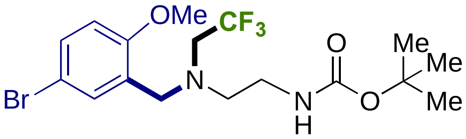

33: 43%

Title compound prepared using general alkylative-trifluoroethylation method B. Purified using standard work-up and flash column chromatography (2-10% EtOAc in pentane); isolated as a clear oil (107 mg, 43%).

^{1}H NMR (400 MHz, $CDCl_3$) δ 7.50 (d, *J* = 2.6 Hz, 1H), 7.31 (dd, *J* = 8.7, 2.6 Hz, 1H), 6.72 (d, *J* = 8.7 Hz, 1H), 4.50 (br. s, 1H, NH), 3.78 (s, 3H), 3.76 (s, 2H), 3.10 (q, *J* = 9.6 Hz, 2H), 3.12 – 3.01 (m, 2H), 2.59 (t, *J* = 7.2 Hz, 2H), 1.45 (d, *J* = 12.6 Hz, 13H), 1.28 (h, *J* = 4.8, 3.8 Hz, 4H); **^{13}C NMR** (101 MHz, $CDCl_3$) δ 156.6, 156.0, 132.3, 130.6, 129.4, 125.9 (q, *J* = 281.0 Hz), 112.9, 112.0, 79.0, 55.5, 54.6 (q, *J* = 30.3 Hz), 54.4, 52.5, 40.5, 30.0, 28.4, 27.3, 26.7–26.4 (m, 2C); **^{19}F NMR** (376 MHz, $CDCl_3$) δ -69.43 (t, *J* = 9.6 Hz); **IR** (cm^{-1}) (neat) 2934, 2860, 1696, 1487, 1366, 1269, 1248, 1169, 1135, 1082; **HRMS** (ESI+): Exact mass calcd for $C_{21}H_{32}BrF_3N_2NaO_3$ [M+Na], 519.1441. Found 519.1434, σ = 0.0017.

1-(3-((3,7-dimethyloct-6-en-1-yl)(2,2,2-trifluoroethyl)amino)propyl)pyrrolidin-2-one (**34**)

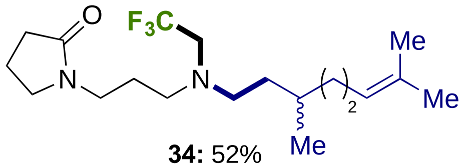

34: 52%

Title compound prepared using general alkylative-trifluoroethylation method B. Purified using standard work-up and flash column chromatography (20-50% EtOAc in pentane); isolated as a clear oil (94 mg, 52%).

^{1}H NMR (400 MHz, $CDCl_3$) 5.15 – 5.02 (m, 1H), 3.37 (t, *J* = 7.0 Hz, 2H), 3.30 (t, *J* = 7.4 Hz, 2H), 3.00 (q, *J* = 9.6 Hz, 2H), 2.69 – 2.52 (m, 4H), 2.37 (t, *J* = 8.1 Hz, 2H), 2.09 – 1.86 (m, 4H), 1.78 – 1.55 (m, 8H), 1.54 – 1.38 (m, 2H), 1.37 – 1.08 (m, 3H), 0.87 (d, *J* = 6.3 Hz, 3H); **^{13}C NMR** (101 MHz, $CDCl_3$) δ 174.9, 131.2 (C_{quat}), 125.8 (q, *J* = 280.9 Hz), 124.7 (=CH), 55.0 (q, *J* = 30.4 Hz), 53.2 (CH_2), 52.6 (CH_2), 47.2 (CH_2), 40.5 (CH_2), 37.1 (CH_2), 34.1 (CH_2), 31.0 (CH_2), 30.3 (CH), 25.7 (CH_2), 25.4 (CH_2), 19.6 (2 x CH_3), 17.9 (CH_2), 17.6 (CH_3); **^{19}F NMR** (376 MHz, $CDCl_3$) δ -70.13 (t, *J* = 9.5 Hz); **IR** (cm^{-1}) (neat) 2958, 2926, 2857, 1671, 1464, 1427, 1269, 1137, 1086; **HRMS** (ESI+): Exact mass calcd for $C_{19}H_{34}F_3N_2O$ [M+H], 363.2617. Found 363.2628, σ = 0.0026.

N-((6-bromobenzo[*d*][1,3]dioxol-5-yl)methyl)-*N*-(2,2,2-trifluoroethyl)cyclohexanamine (**35**)

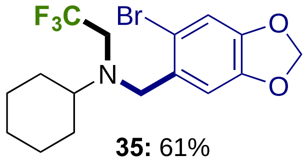

35: 61%

Title compound prepared using general alkylative-trifluoroethylation method A. Purified using standard work-up and flash column chromatography (2-5% EtOAc in pentane); isolated as a pale yellow oil (121 mg, 61%).

^{1}H NMR (400 MHz, $CDCl_3$) δ 7.15 (s, 1H), 6.95 (s, 1H), 5.97 (s, 2H), 3.83 (s, 2H), 3.12 (q, *J* = 9.3 Hz, 2H), 2.40 (tt, *J* = 11.2, 3.6 Hz, 1H), 1.92 – 1.74 (m, 4H), 1.67 – 1.57 (m, 1H), 1.30 – 0.99 (m, 5H); **^{13}C NMR** (101 MHz, $CDCl_3$) δ 147.5, 147.1, 132.3, 125.8 (q, *J* = 279.0 Hz), 113.3, 112.2, 109.7, 101.5, 60.1, 56.0, 51.5 (q, *J* = 31.3 Hz), 29.2, 26.0, 25.9; **^{19}F NMR** (376 MHz, $CDCl_3$) δ -71.29 (t, *J* = 9.3 Hz); **IR** (cm^{-1}) (neat) 2929, 2855, 1503, 1475, 1230, 1137, 1101, 1073, 1037; **HRMS** (ESI+): Exact mass calcd for $C_{16}H_{20}BrF_3NO_2$ [M+H], 394.0624. Found 394.0619, σ = 0.0152.

Synthesis and characterization data for compounds 36-39 (ketones)

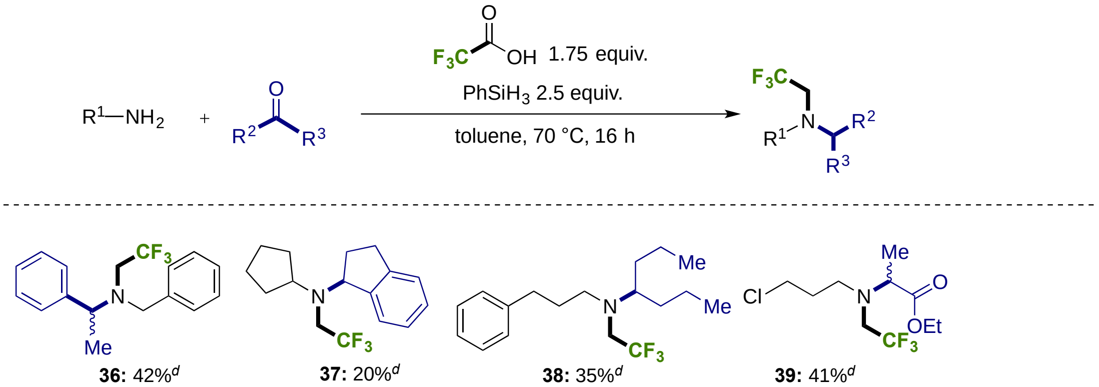

36: 42%[d] **37:** 20%[d] **38:** 35%[d] **39:** 41%[d]

as depicted, the blue fragment with emboldened N-C bond is the ketone fragment and the non-bold black bond is the original amine moiety.

N-benzyl-2,2,2-trifluoro-*N*-(1-phenylethyl)ethan-1-amine (**36**)

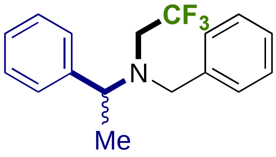

36: 42%

Title compound prepared using general alkylative-trifluoroethylation method E. Purified using standard work-up and flash column chromatography (1-6% dichloromethane in pentane); isolated as a clear oil (61 mg, 42%).

^{1}H NMR (400 MHz, $CDCl_3$) δ 7.40 – 7.32 (m, 8H), 7.31 – 7.25 (m, 2H), 4.05 (q, *J* = 6.9 Hz, 1H), 3.80 (d, *J* = 14.0 Hz, 1H), 3.75 (d, *J* = 14.0 Hz, 1H), 3.24 (dq, *J* = 15.4, 9.4 Hz, 1H), 2.98 (dq, *J* = 15.4, 9.4 Hz, 1H), 1.44 (d, *J* = 6.9 Hz, 3H); **^{13}C NMR** (101 MHz, $CDCl_3$) δ 142.1, 139.0, 128.7, 128.3, 128.2, 127.8, 127.2, 127.2, 126.0 (q, *J* = 281.1, Hz), 58.1, 55.3, 50.2 (q, *J* = 30.9 Hz), 15.7; **^{19}F NMR** (376 MHz, $CDCl_3$) δ -69.15 (t, *J* = 9.4 Hz); **IR** (cm^{-1}) (neat) 3063, 3030, 2975, 1494, 1453, 1432, 1268, 1134, 1097, 1070; **HRMS** (ESI+): Exact mass calcd for $C_{17}H_{18}F_3NNa$ [M+Na], 316.1284. Found 316.1293, σ = 0.0160.

N-(3-phenylpropyl)-*N*-(2,2,2-trifluoroethyl)-2,3-dihydro-1H-inden-1-amine (**37**)

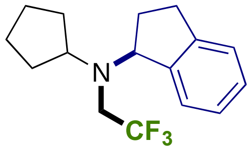

37: 20%

Title compound prepared using general alkylative-trifluoroethylation method E. Purified using standard work-up and flash column chromatography (1-4% EtOAc in pentane); isolated as a clear oil (28 mg, 20%).

^{1}H NMR (400 MHz, $CDCl_3$) 7.38 – 7.31 (m, 1H), 7.24 – 7.17 (m, 3H), 4.56 (dd, *J* = 9.1, 8.1 Hz, 1H), 3.25 – 3.18 (m, 1H), 3.14 (q, *J* = 9.3 Hz, 2H), 2.92 (ddd, *J* = 16.1, 9.1, 2.9 Hz, 1H), 2.76 (ddd, *J* = 16.1, 8.9 Hz, 8.1, 1H), 2.28 (dddd, *J* = 12.8, 8.9, 8.1, 2.9 Hz, 1H), 1.87 (dddd, *J* = 12.8, 9.1, 9.1, 8.9 Hz, 1H), 1.81 – 1.67 (m, 2H), 1.67 – 1.31 (m, 6H); **^{13}C NMR** (101 MHz, $CDCl_3$) δ 144.1, 143.2, 127.3, 126.5 (q, *J* = 279.7 Hz), 126.3, 124.8, 124.6, 67.0, 61.2, 49.9 (q, *J* = 31.6 Hz), 31.6, 30.0, 29.5, 29.4, 24.1, 23.3; **^{19}F NMR** (376 MHz, $CDCl_3$) δ -70.8 (t, *J* = 9.3 Hz); **IR** (cm^{-1}) (neat) 2953, 2870, 1477, 1458, 1267, 1131, 1097,

1023; **HR-GC-EIMS**: rt = 12.30 min. Exact mass calcd for $C_{16}H_{20}NF_3$, 283.15424. Found 283.15300, (mass difference: -4.35 ppm).

N-(3-phenylpropyl)-*N*-(2,2,2-trifluoroethyl)heptan-4-amine (**38**)

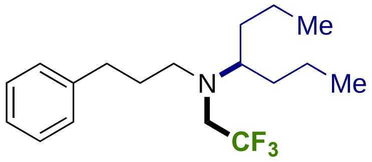

38: 35%

Title compound prepared using general alkylative-trifluoroethylation method E. Purified using standard work-up and flash column chromatography (1-3% EtOAc in pentane); isolated as a clear oil (55 mg, 35%).

^{1}H NMR (400 MHz, $CDCl_3$) δ 7.33 – 7.25 (m, 2H), 7.22 – 7.15 (m, 3H), 3.00 (q, *J* = 9.5 Hz, 2H), 2.68 – 2.61 (m, 2H), 2.59 (t, *J* = 7.1 Hz, 2H), 2.50 (h, *J* = 6.6 Hz, 1H), 1.80 – 1.68 (m, 2H), 1.50 – 1.16 (m, 8H), 0.90 (t, *J* = 6.9 Hz, 6H); **^{13}C NMR** (101 MHz, $CDCl_3$) δ 142.5, 128.3, 128.3, 126.0 (q, *J* = 279.2 Hz), 125.6, 61.7, 52.9 (q, *J* = 31.0 Hz), 51.1, 33.6, 33.3, 31.1, 20.2, 14.2; **^{19}F NMR** (376 MHz, $CDCl_3$) δ -71.21 (t, *J* = 9.5 Hz); **IR** (cm^{-1}) (neat) 3027, 2958, 2931, 2871, 1455, 1270, 1134, 1088; **HRMS** (ESI+): Exact mass calcd for $C_{18}H_{29}F_3N$ [M+H], 316.2247. Found 316.2255, σ = 0.0066.

ethyl *N*-(3-chloropropyl)-*N*-(2,2,2-trifluoroethyl)alaninate (**39**)

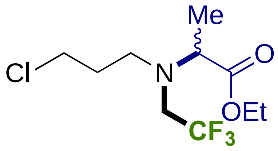

39: 41%

Title compound prepared using general alkylative-trifluoroethylation method E. Purified using standard work-up and flash column chromatography (5-10% EtOAc in pentane); isolated as a clear oil (57 mg, 41%).

^{1}H NMR (400 MHz, $CDCl_3$) δ 4.17 (q, *J* = 7.1 Hz, 2H), 3.60 (t, *J* = 6.4 Hz, 2H), 3.56 (q, *J* = 7.3 Hz, 1H), 3.36 – 3.17 (m, 2H), 2.86 (t, *J* = 6.6 Hz, 2H), 1.94 – 1.85 (m, 2H), 1.34 (d, *J* = 7.3 Hz, 3H), 1.28 (t, *J* = 7.1 Hz, 3H); **^{13}C NMR** (101 MHz, $CDCl_3$) δ 173.6, 126.0 (q, *J* = 279.4 Hz), 60.6, 59.8, 53.3 (q, *J* = 31.8 Hz), 50.7, 42.3, 31.7, 16.6, 14.3; **^{19}F NMR** (376 MHz, $CDCl_3$) δ -71.99 (t, *J* = 9.5 Hz); **IR** (cm^{-1}) (neat) 2985, 2941, 1730, 1271, 1138, 1109, 833; **HRMS** (ESI+): Exact mass calcd for $C_{10}H_{18}ClF_3NO_2$ [M+H], 276.0973. Found 276.0983, σ = 0.0764.

Supplementary Discussion

Supplementary Table 1. Optimization of the trifluoroethylation reaction

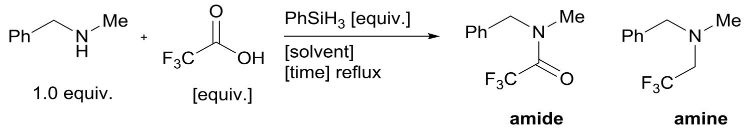

entry	Solvent (reflux)	time /h	TFA equiv	$PhSiH_3$ equiv.	additives/conditions	amide %	amine %
1	toluene	16	1.00	1.00	-	18	42
2	toluene	16	1.00	2.00	-	43	39
3	toluene	4	2.00	2.00	-	9	85
4	toluene	4	1.00	2.00	amine.HCl	17	52
5	toluene	4	1.00	2.00	amine.HCl + 1 drop Et_3N	31	50
6	THF	4	1.50	2.00	-	22	71
**7**	**THF**	**4**	**1.75**	**2.00**	**-**	**8**	**84**
**8**	**THF**	**4**	**1.75**	**2.00**	open to air/undried solvent	**8**	**78**
9	THF	3	1.75	2.00	Fig 4B in manuscript	6	80
10	THF	3	1.00	2.00	Fig 4B in manuscript	54	0

[a]0.5 mL solvent containing phenylsilane and trifluoroacetic acid. Heat to temperature, remove from heat to add amine (0.5 mmol), then return to heat for time required. Cooled and concentrated and conversion determined by ^{1}H-NMR relative to 1,1,2,2-tetrachloroethane as an internal standard.

Supplementary Figure 1. Azide tolerance experiment

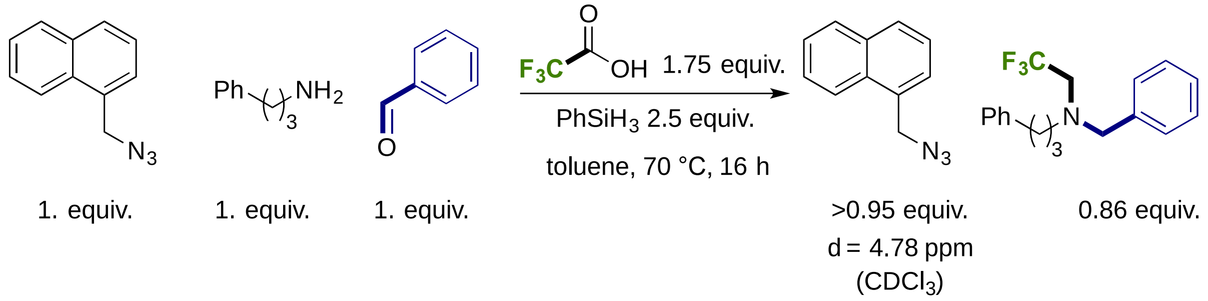

The standard alkylation-trifluoroethylation reaction was performed in the presence of one equivalent of 1-(azidomethyl)naphthalene to test the stability of azides to the reductive conditions. After 16 h at 70 °C, the reaction was concentrated and the conversions to products deduced by inspection of the ^{1}H-NMR spectrum and comparison to 1,1,2,2-tetrachloroethane as an internal standard, in chloroform. Analysis suggested full tolerance of the azide to the conditions, which did not interfere with the desired transformation, which still ran to 86% conversion to the alkylated-trifluoroethylated amine.

Supplementary Figure 2. Amine competition experiments

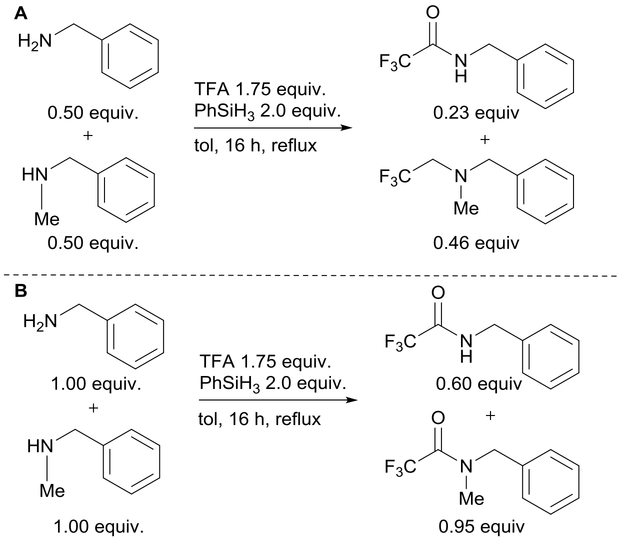

Experiment A:

Primary amines give amide; secondary amines give amine, even in the same pot.

Benzylamine (0.25 mmol) and *N*-methyl-1-phenylmethanamine (0.25 mmol) were combined in a flask and toluene (0.5 mL) and phenylsilane (1.00 mmol) added. The reaction was heated to reflux and trifluoroacetic acid (0.875 mmol) was added and the reaction stirred for 16 h. After this time, the reaction was concentrated and the conversion to each product determined by integration of convenient ^{1}H-NMR signals and comparison to 0.25 mmol of 1,1,2,2-tetrachloroethane as an internal standard. The analysis indicated that there was a 46% conversion of the available primary amine to amide, and no detectable trifluoroethylated amine. A 92% conversion of the available secondary amine to the trifluoroethylated amine was observed, and no appreciable level of amide.

Experiment B:

In the absence of excess acidity, only amide is observed. Secondary amines outcompete primary amines.

Benzylamine (0.50 mmol) and *N*-methyl-1-phenylmethanamine (0.50 mmol) were combined in a flask and toluene (0.5 mL) and phenylsilane (1.00 mmol) added. The reaction was heated to reflux and trifluoroacetic acid (0.875 mmol) was added and the reaction stirred for 16 h. After this time, the reaction was concentrated and the conversion to each product determined by integration of convenient ^{1}H-NMR signals and comparison to 0.25 mmol of 1,1,2,2-tetrachloroethane as an internal standard. The analysis indicated that there was a 60% conversion of the available primary amine to amide, and no detectable trifluoroethylated amine. A 95% conversion of the available secondary amine to the trifluoroacetamide was observed, and no appreciable level of amine.

Supplementary Figure 3. Resubjection of amide to the reaction conditions

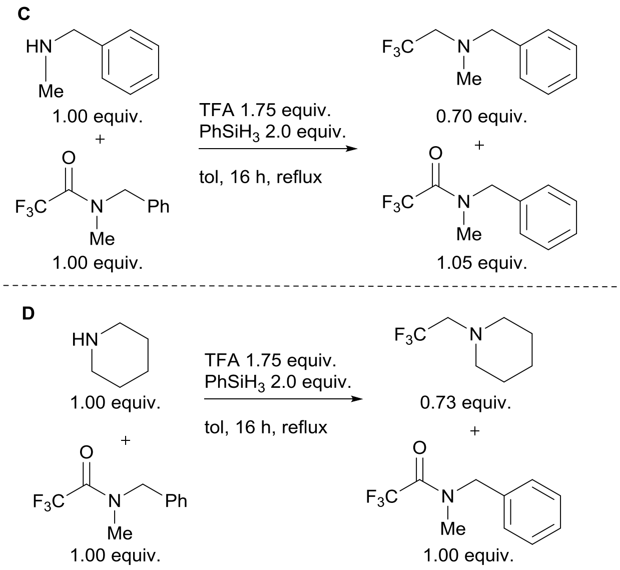

Experiment C:

Amide is not reduced to amine under the reaction conditions. Typical background amide formation of ~5% observed.

N-benzyl-2,2,2-trifluoro-*N*-methylacetamide (0.50 mmol) and *N*-methyl-1-phenylmethanamine (0.50 mmol) were combined in a flask and toluene (0.5 mL) and phenylsilane (1.00 mmol) added. The reaction was heated to reflux and trifluoroacetic acid (0.875 mmol) was added and the reaction stirred for 16 h. After this time, the reaction was concentrated and the conversion to each product determined by integration of convenient ^{1}H-NMR signals and comparison to 0.25 mmol of 1,1,2,2-tetrachloroethane as an internal standard. The analysis indicated that there was a 70% conversion of secondary amine to the trifluoroethylated amine. 1.05 equivalents of amide were observed, which is consistent with no reduction of amide + 5% background reaction (see Supplementary Table 1).

To rule out a coincidental result and show that amide is not reduced, experiment D utilised a different secondary amine.

Experiment D (Fig S4, Manuscript Fig 4A)

Amide is not reduced to amine under the reaction conditions.

N-benzyl-2,2,2-trifluoro-*N*-methylacetamide (0.50 mmol) and piperidine (0.50 mmol) were combined in a flask and toluene (0.5 mL) and phenylsilane (1.00 mmol) added. The reaction was heated to reflux and trifluoroacetic acid (0.875 mmol) was added and the reaction stirred for 16 h. After this time, the reaction was concentrated and the conversion to each product determined by integration of convenient ^{1}H-NMR signals and comparison to 0.25 mmol of 1,1,2,2-tetrachloroethane as an internal standard. The analysis indicated that there was a 73% conversion of secondary amine to the trifluoroethylated piperidine. 1.00 equivalent of amide was observed, ruling out the reduction of free amide by an external reductant as the mechanism of reduction.

Supplementary Figure 4. Attempts to observe trifluoroacetaldehyde

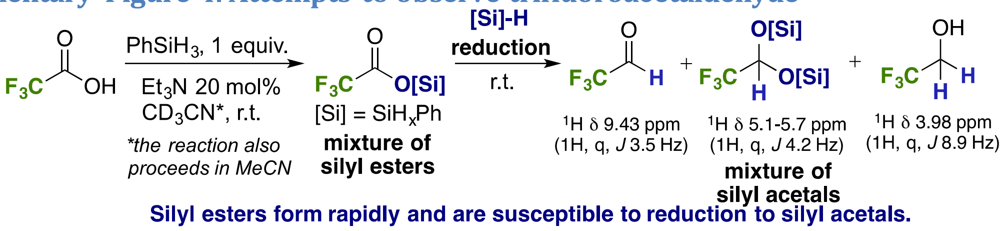

Silyl esters form rapidly and are susceptible to reduction to silyl acetals.

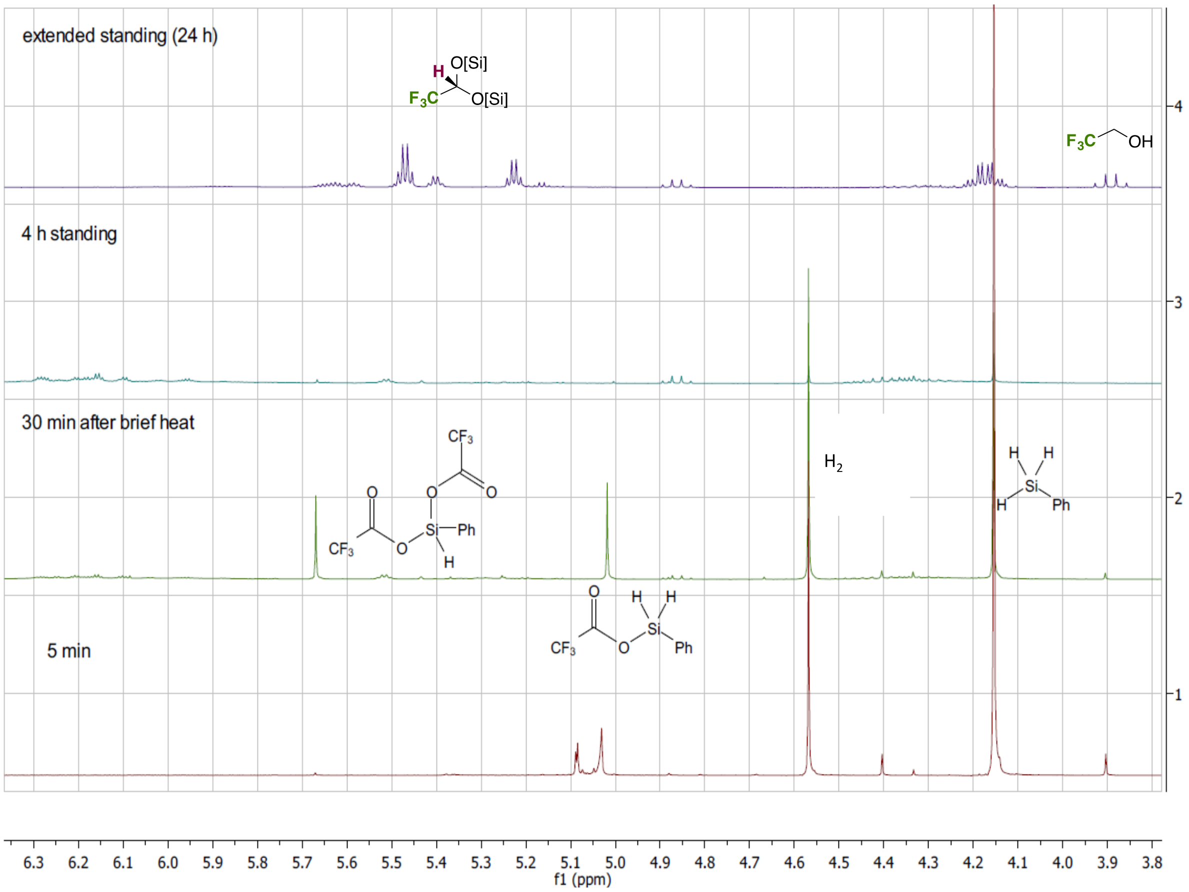

Trifluoroacetic acid (0.60 mmol) and phenylsilane (0.20 mmol) were mixed in deuterated acetonitrile in an NMR tube at rt. Triethylamine (0.02 mmol) was added and the reaction followed by NMR. Hydrogen evolution was observed, and dissolved hydrogen identified at 4.57 ppm. The reaction was briefly heated to reflux with a heat gun, and allowed to stand at rt for 30 min. Species consistent with previously observed silyl ester species[9] were noted at 5.02 and 5.67 ppm, and traces of acetal type species were also apparent, suggesting the rates of formation and reduction of the trifluoroacetoxysilyl esters were similar. (Note that acetals are not observed if the reaction is repeated with acetic acid.) No trifluoroacetaldehyde was observed at this point. After 4 h of standing, the initially observed silyl ester species had begun to react further, and evidence of polymeric siloxy species were deduced by the observation of multiple overlapping peaks between 4 and 7 ppm, indicative of a range of silyl hydride species. After standing at room temperature in the NMR tube overnight, a small amount of trifluoroacetaldehyde was visible (9.43 ppm); as was trifluoroethanol (3.98 ppm) and possible derivatives. Importantly, several species consistent with known trifluoroacetaldehyde bissilyl acetals were identifiable (q, 5.2-5.7 ppm).[10]

Aldehydes with strong electon withdrawing groups are known to exist preferentially as the corresponding hydrate.[1,11] Amines are also known to form the hemiaminals with trifluoroacetaldehyde.[11] Such behaviour of carbonyl groups is a function of the electron withdrawing capabilities of the R (e.g. CF_3) group, and it appears to be the electron deficient nature of these acids that promote in situ reduction in this reaction, consistent with the pK_a study.

Supplementary Figure 5. Acid competition experiment

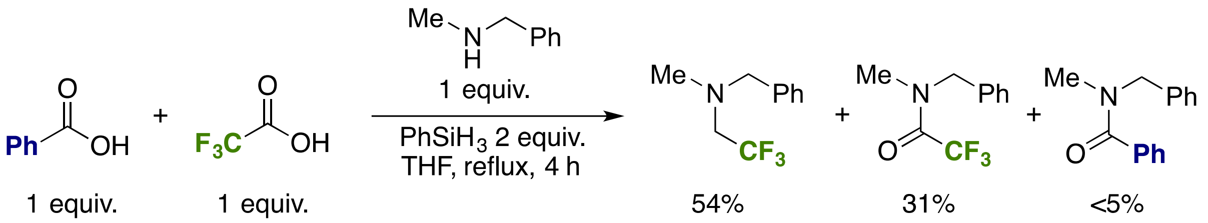

Use of benzoic acid in the reaction does not leading to benzyl derivatives of the amine. Its reduced acidity reduces the amount of trifluoroethylamine product observed, although more amine is seen than if it were absent.

Benzoic acid (0.50 mmol), trifluoroacetic acid (0.50 mmol) and *N*-methyl-1-phenylmethanamine (0.50 mmol) were combined in a flask and THF (0.5 mL) added. The reaction was heated to reflux for 1 min, phenylsilane (1.00 mmol) added, and the reaction stirred for 4 h. After this time, the reaction was concentrated and the conversion to each product determined by integration of convenient ^{1}H-NMR signals and comparison to 0.25 mmol of 1,1,2,2-tetrachloroethane as an internal standard. The analysis indicated that there was a 54% conversion to the trifluoroethylated amine, with 31% present as the trifluoroacetamide, composed as two rotamers. The benzamide was present in small amounts, again present as rotamers.

The role of the excess acid and strength of acid.

In the absence of trifluoroacetic acid, the above reaction leads to a moderate yield of benzamide. The competition experiment between trifluoroacetic acid and benzoic acid, shown above, indicates that it is the high electrophilicity of the trifluoroacetoxysilyl ester that induces *in situ* reduction, and that the pK_a study likely reflects ease of reduction of the different silyl esters. Additionally, it reveals further the role of the additional equivalent of acid. In this case, the extra equivalent is less acidic than when a second equivalent of TFA is employed, but does add additional acidity compared to the case in which there is only one equivalent of TFA. Accordingly, the amount of amine formed (54%) is greater than with a single equivalent of TFA (~0%), but lower than when two equivalents of TFA are used (>80%). Additionally, Entries 4 and 5 in Supplementary Table 1 show that using 1 equivalent of TFA and introducing a second equivalent of acid as the amine HCl salt gives slightly higher yields (+ 10%) than the control experiment (entry 1) in which only 1 equivalent of TFA was used and no HCl.
Note also that additional amines of lower basicity (pyridines) are tolerated in the scope to some extent, as they do not compete too strongly for protonation. Performing the trifluoroethylation reaction in neat pyridine, however, gives only amide product. When basic amines are formed *in situ* (see 3.14 below), the conversion to amine suffers.

Amine Identity

Anilines tend to preferentially form the trifluoroacetamide in the standard trifluoroethylation reaction (anilines are more sluggish to react with typical acids, e.g. benzoic acid).[9] Mimura has shown anilines require more forcing conditions to undergo reductive amination reactions with trifluoroacetaldehyde derivatives, as they exist largely as the hemiaminals (and need to be the imine/iminium to be reduced).[1]
Primary amines are variable substrates in the trifluoroethylation reaction, tending to prefer to form amide (we have occasionally isolated up to 50% of the trifluoroethylamine product of primary amines). This may be because primary amines form neutral imine species instead of iminium ions in the hemiaminal equilibrium. Because the equilibrium lies largely towards the hemiaminals,[1] it is only the secondary amine (iminium) intermediates that are reductively trapped by phenylsilane, which is slower to reduce neutral species, normally requiring an activating agent).

Supplementary Figure 6. General form of competing reactions and mass balance analysis

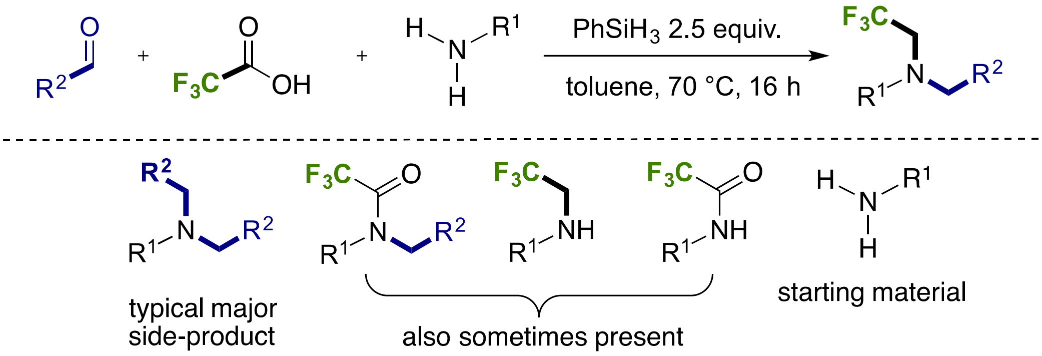

Supplementary Figure 7. Example of competing reactions and mass balance analysis

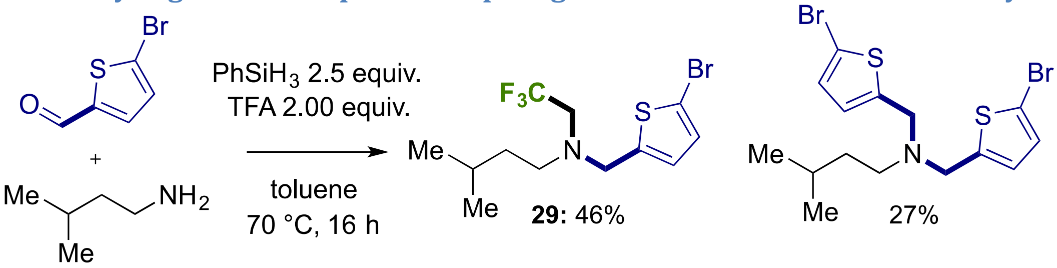

The major competing product in the typical reaction is trifluoroacetamide. In the three-component coupling (above), over-alkylation is often a competing species observed. The resulting tertiary amine can vitiate the excess acidity of the reaction, which releases free amine, which increases tertiary trifluoroacetamide formation. Likewise, this leaves primary amine unreacted, which can go on to form amide (and occasionally the secondary trifluoroethylamine). For difficult reactions, therefore, it is beneficial to alkylate the primary amine separately, and then subjecting the secondary amine to our trifluoroethylation protocol. Another potential problem is the trapping of the acetal species as aminals. Below, an intramolecular trapping experiment shows that if the aldehyde level intermediate is prevented from forming the iminium ion, reduction is slowed.

Supplementary Figure 8. Intramolecular trapping of partially reduced intermediates

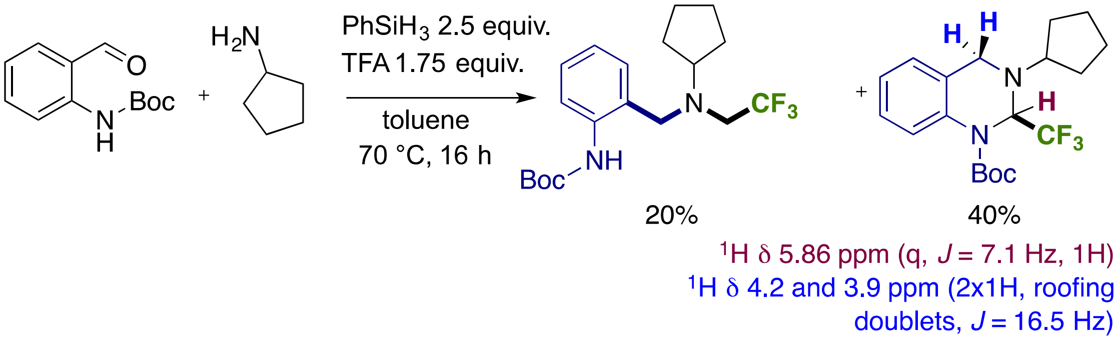

Likewise, if the amine substrate is bidentate and can react with the initial aldehyde/imine to form a cyclic aminal species the reaction often stalls if reduction of this species is slow.

Supplementary Figure 9. HPLC Data for compound *ent*-26

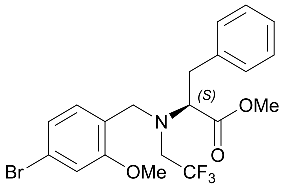

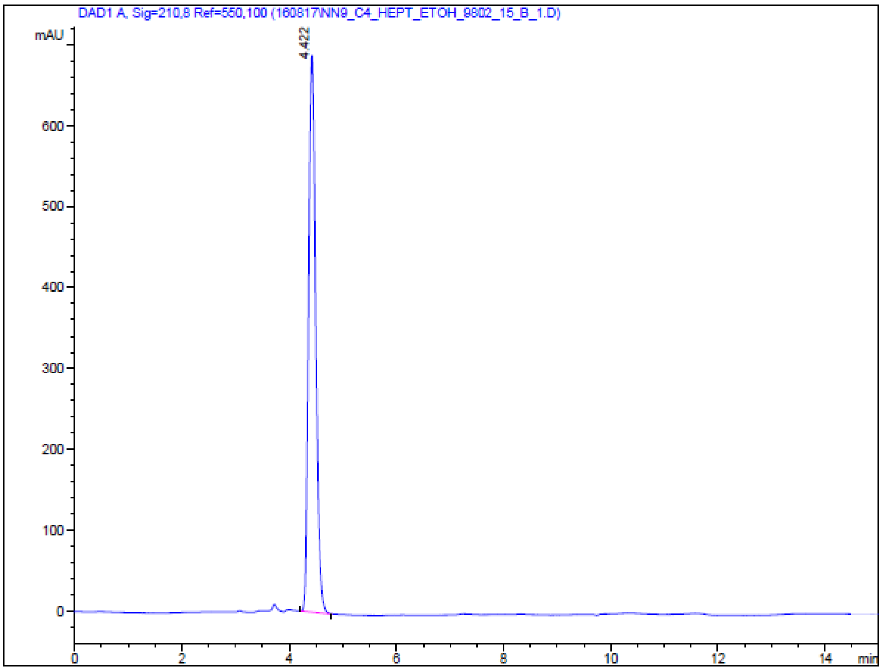

#	Meas. R	Height	Width	Area	Area %	Symmetr
1	4.422	688.424	0.153	6.321e3	100.000	0.869

Separations performed by Reach Separations, (BioCity Nottingham, Pennyfoot Street, Nottingham NG1 1GF).
http://www.reachseparations.com/

Column Details Lux C4 (4.6 mm x 250 mm, 5 µm)
Column Temperature Ambient
Flow Rate 1 mL/min
Detector Wavelength 210 nm
Injection Volume 1.0 uL
Isocratic Conditions 98:02 HEPT:EtOH (0.1% v/v NH_3)

Method: The sample was dissolved to 1 mg/mL in methanol and was then analysed by SFC and HPLC. The result indicated an enantiomeric excess of >99% for both enantiomers.
methyl *N*-(4-bromo-2-methoxybenzyl)-*N*-(2,2,2-trifluoroethyl)-*L*-phenylalaninate (***ent*-26**)

Supplementary Figure 10. HPLC Data for mixture of compound 26 and *ent*-26

HPLC trace for an arbitrary mixture of both enantiomers of methyl *N*-(4-bromo-2-methoxybenzyl)-*N*-(2,2,2-trifluoroethyl)- phenylalaninate (**26**) to confirm separation of enantiomers:

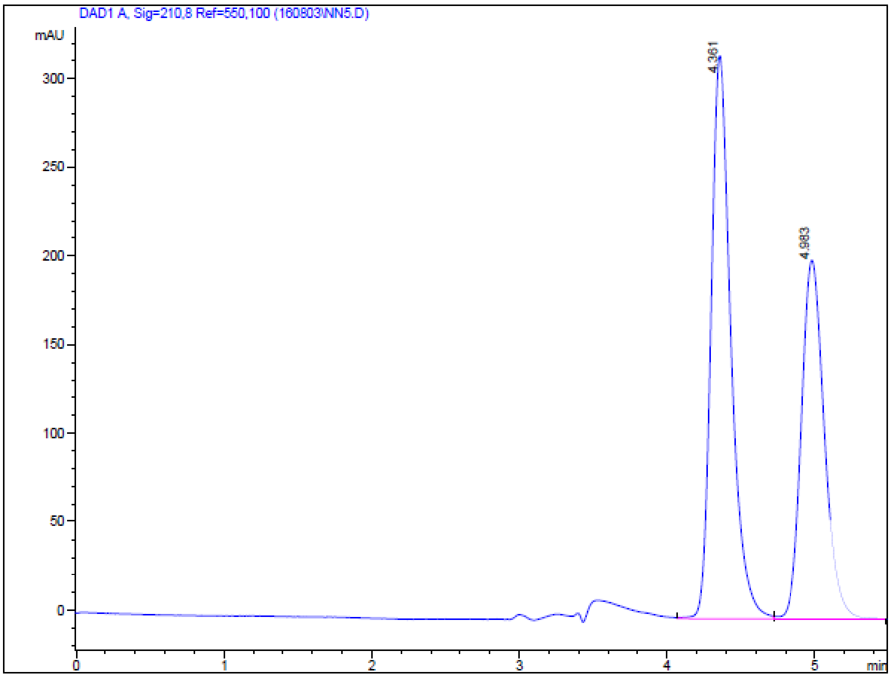

#	Meas. R	Height	Width	Area	Area %	Symmetr
1	4.361	305.609	0.163	3.003e3	57.922	0.801
2	4.983	200.635	0.183	2.182e3	42.078	0.854

UV trace for the components of the mixture (identical as expected for two enantiomers).

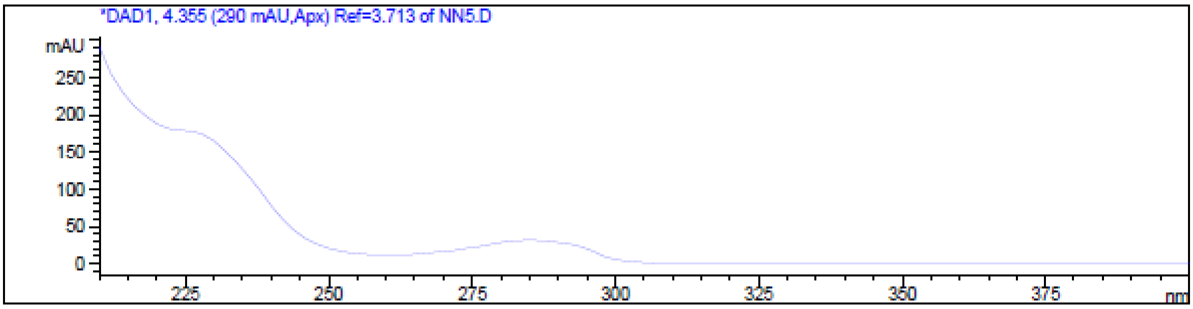

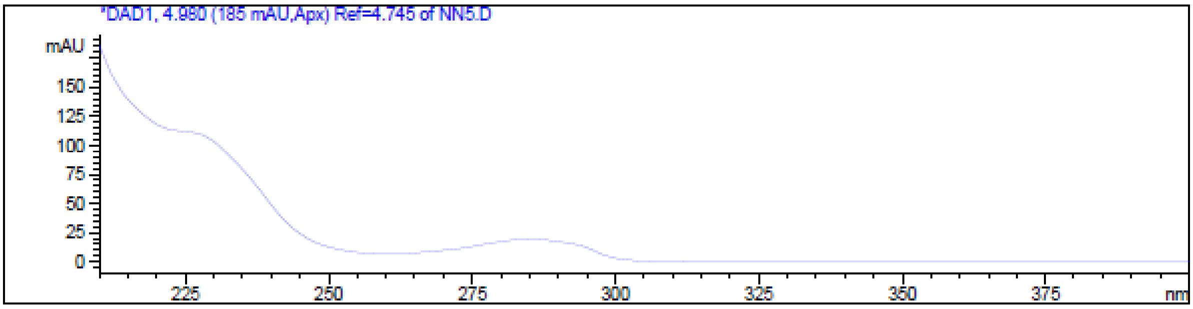

Supplementary Figure 11.

^{1}H-NMR (400 MHz, $CDCl_3$) *N*-benzyl-2,2,2-trifluoro-*N*-methylethan-1-amine (**1**)

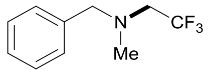

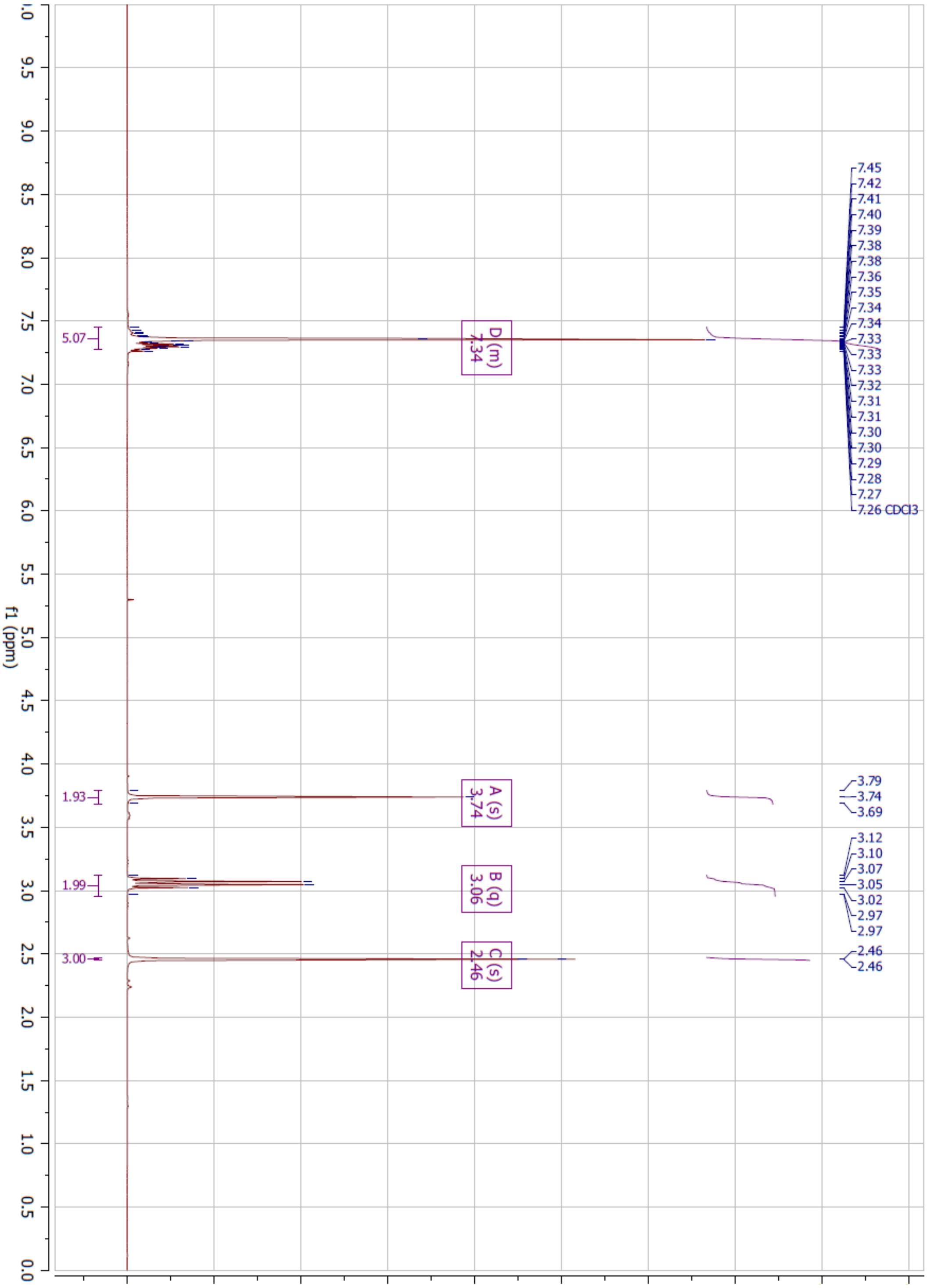

Supplementary Figure 12.

^{13}C-NMR (101 MHz, $CDCl_3$) *N*-benzyl-2,2,2-trifluoro-*N*-methylethan-1-amine (**1**)

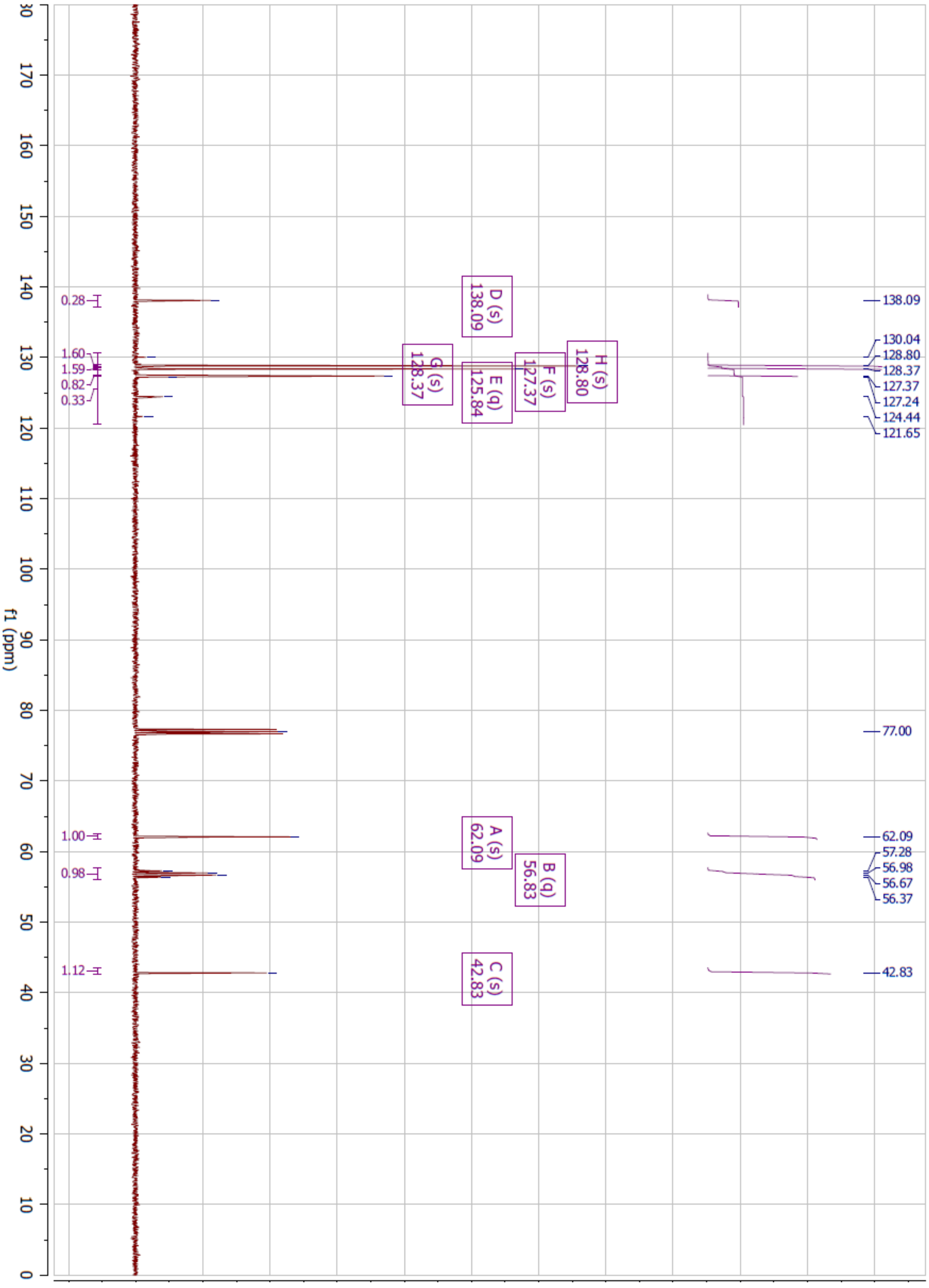

Supplementary Figure 13.

^{19}F-NMR (376 MHz, $CDCl_3$) *N*-benzyl-2,2,2-trifluoro-*N*-methylethan-1-amine (**1**)

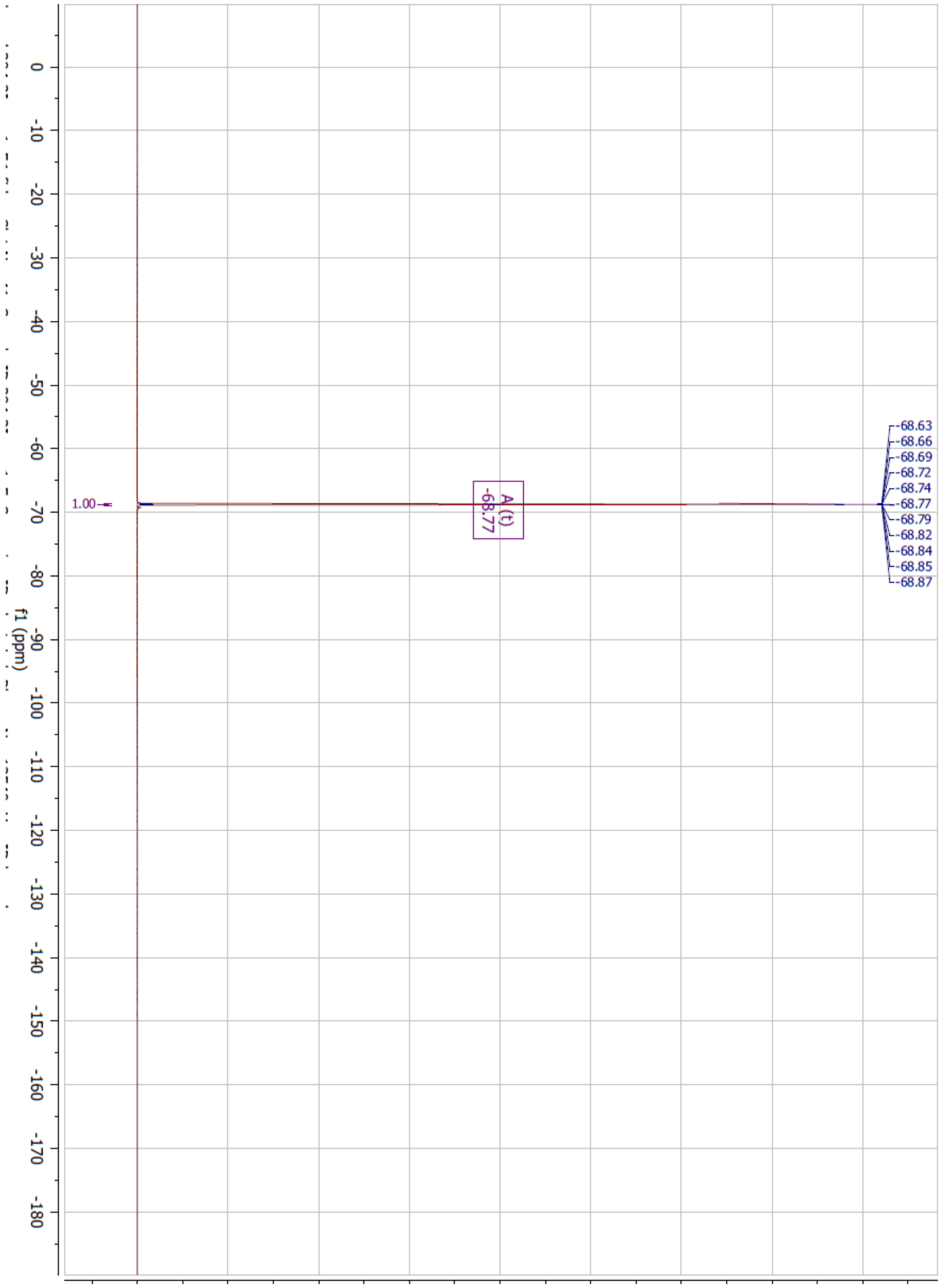

Supplementary Figure 14.

^{1}H-NMR (400 MHz, CDCl$_3$) 2,2,2-trifluoro-N-methyl-N-(2-(pyridin-2-yl)ethyl)ethan-1-amine (**2**)

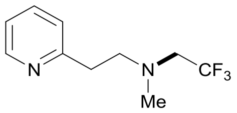

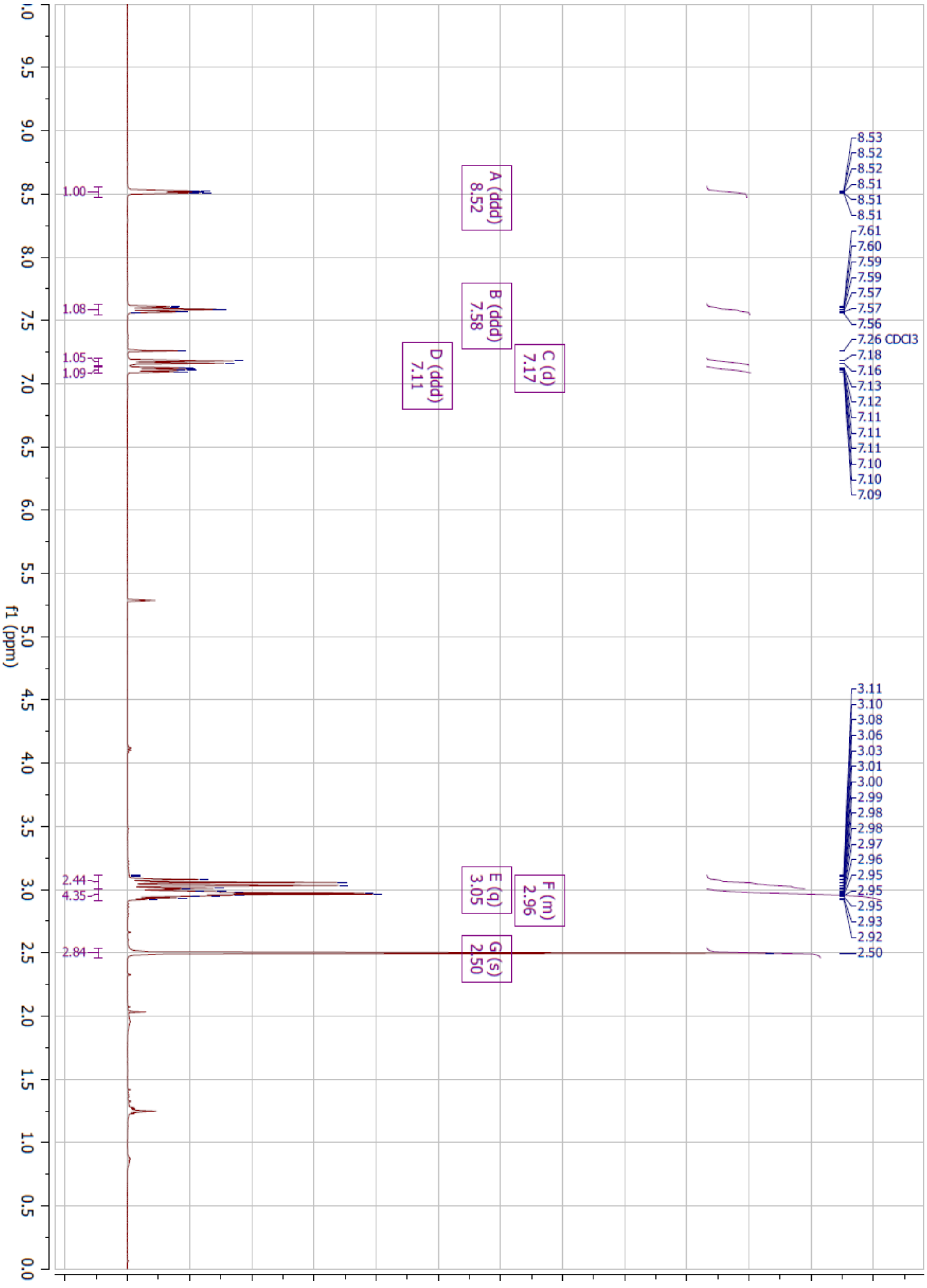

Supplementary Figure 15.

^{13}C-NMR (101 MHz, $CDCl_3$) 2,2,2-trifluoro-N-methyl-N-(2-(pyridin-2-yl)ethyl)ethan-1-amine (**2**)

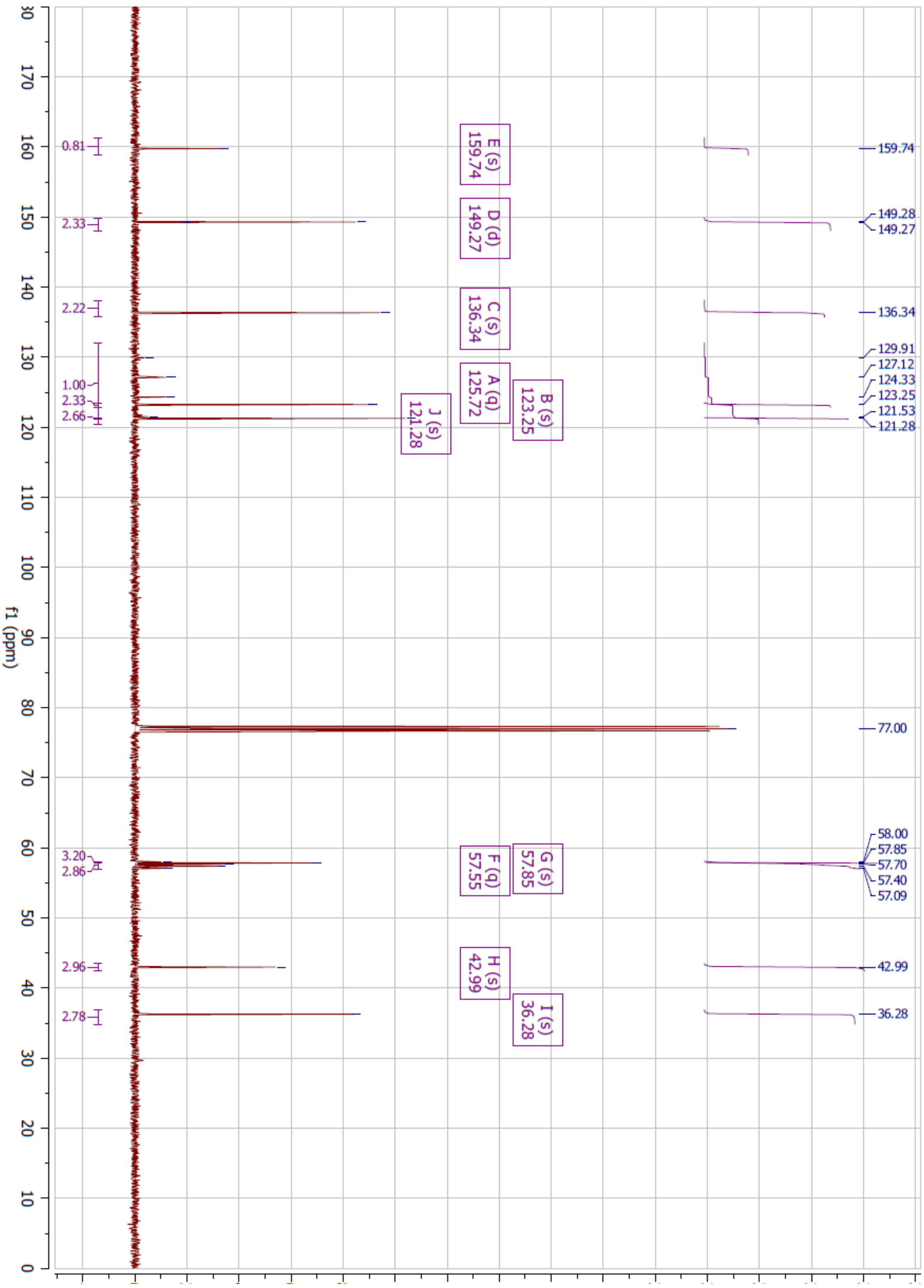

Supplementary Figure 16.

^{19}F-NMR (376 MHz, $CDCl_3$) 2,2,2-trifluoro-N-methyl-N-(2-(pyridin-2-yl)ethyl)ethan-1-amine (**2**)

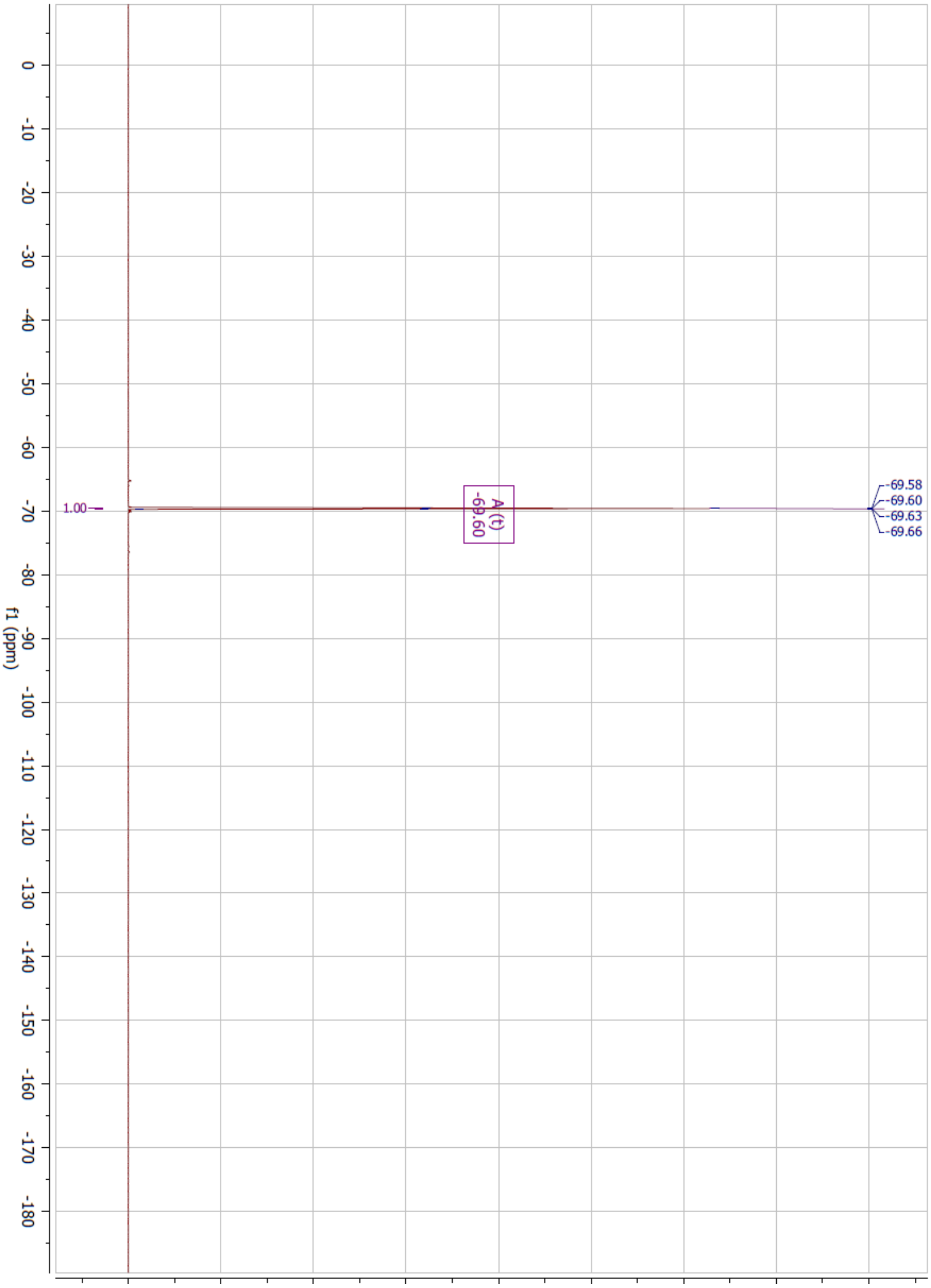

Supplementary Figure 17.

^{1}H-NMR (270 MHz, D_2O) *N,N*-diethyl-2,2,2-trifluoroethan-1-amine hydrochloride (**3**)

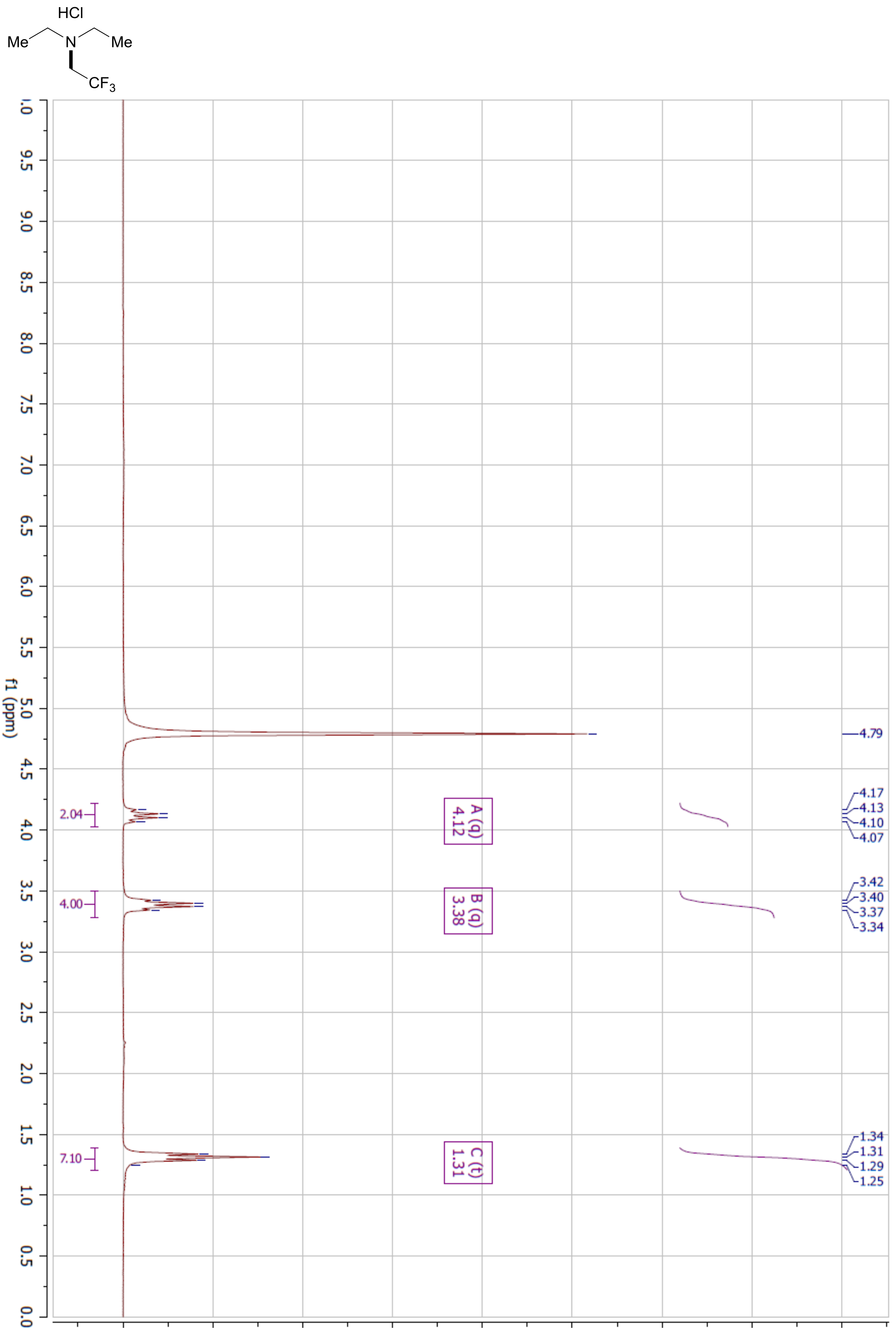

Supplementary Figure 18.
^{13}C-NMR (126 MHz, D_2O) *N,N*-diethyl-2,2,2-trifluoroethan-1-amine hydrochloride (**3**)

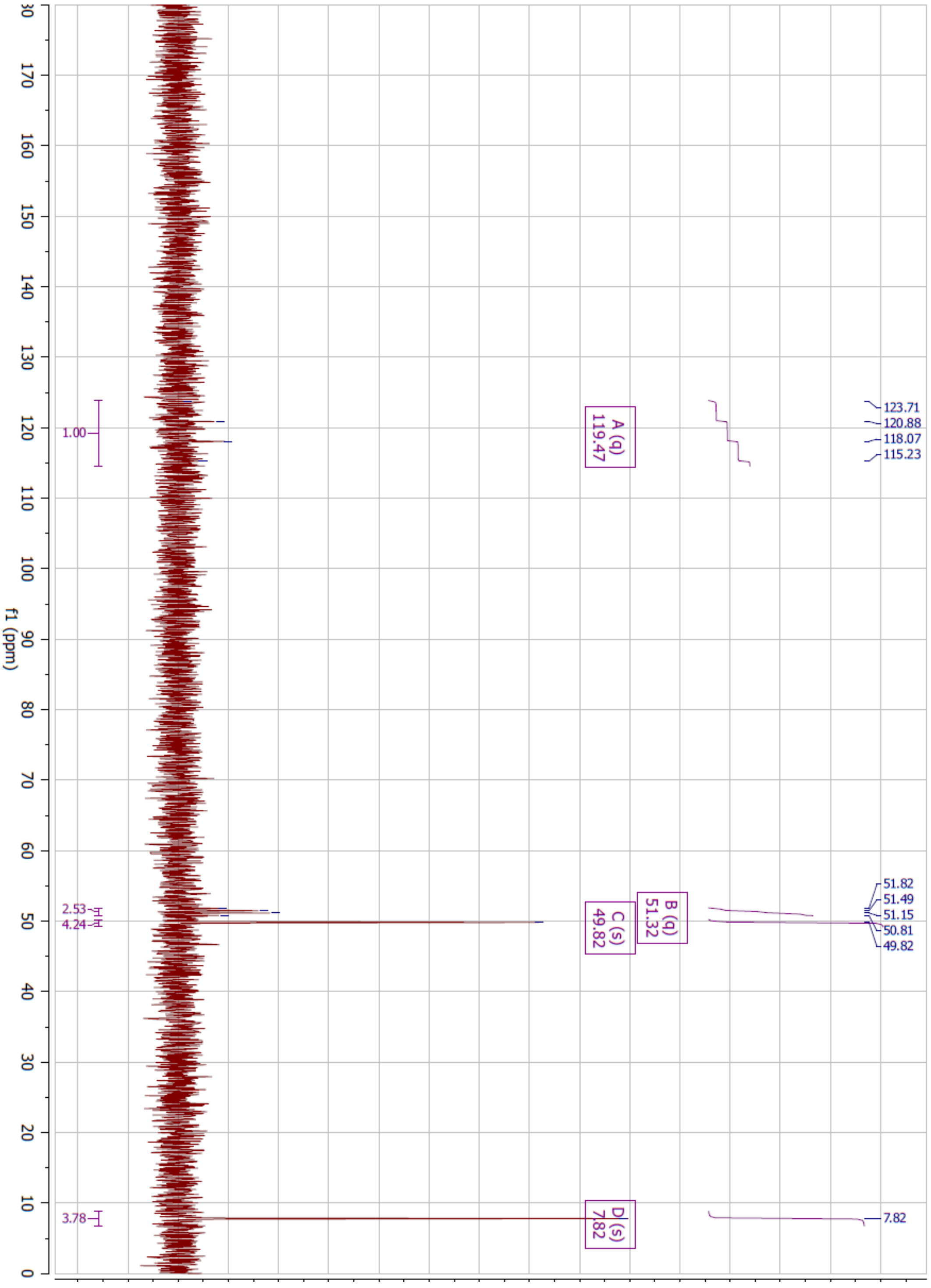

Supplementary Figure 19.

^{19}F-NMR (376 MHz, D_2O) *N,N*-diethyl-2,2,2-trifluoroethan-1-amine hydrochloride (**3**)

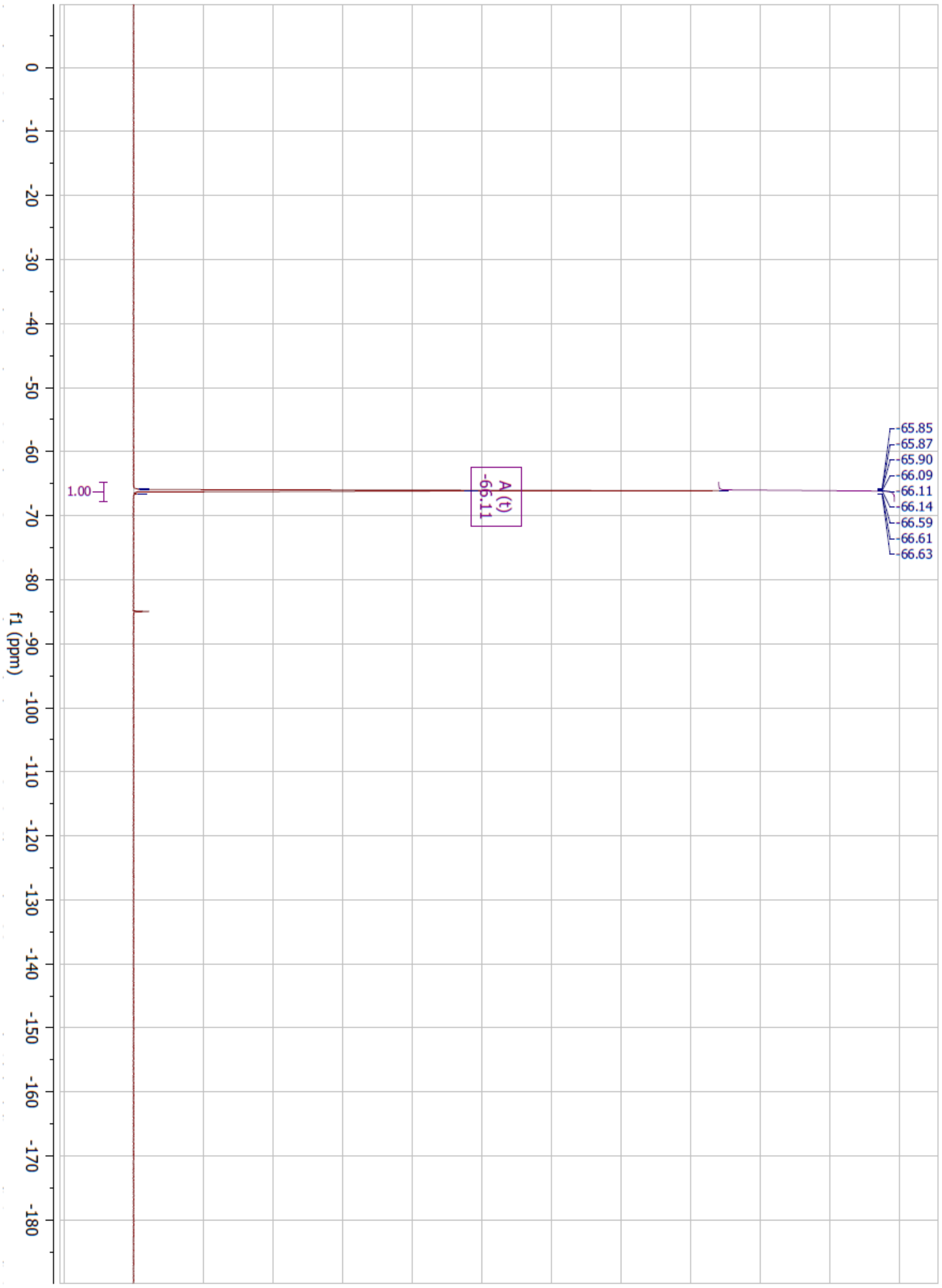

Supplementary Figure 20.

^{1}H-NMR (270 MHz, $CDCl_3$) 2-(2,2,2-trifluoroethyl)-1,2,3,4-tetrahydroisoquinoline (**4**)

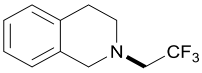

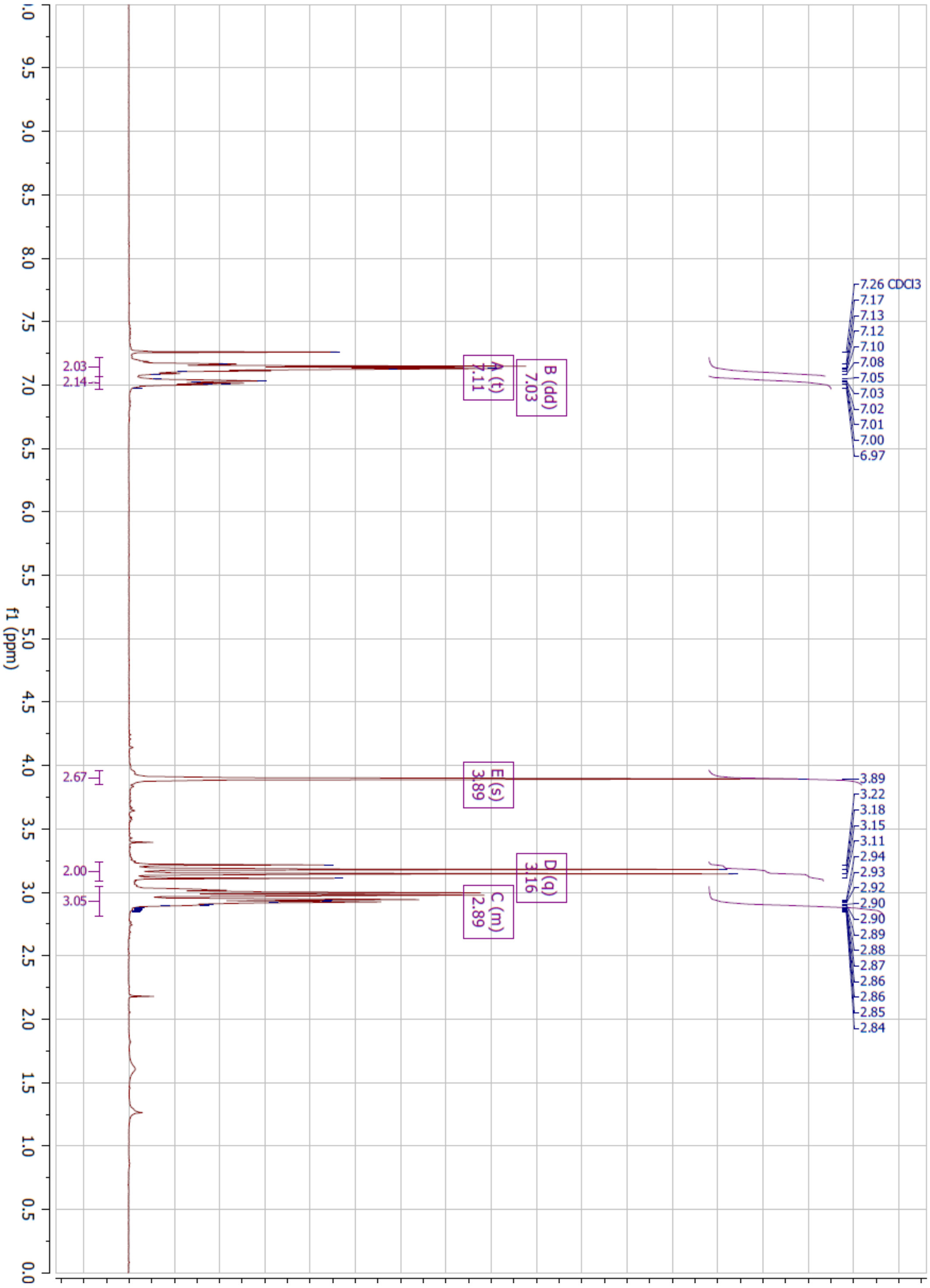

Supplementary Figure 21.

^{13}C-NMR (68 MHz, $CDCl_3$) 2-(2,2,2-trifluoroethyl)-1,2,3,4-tetrahydroisoquinoline (**4**)

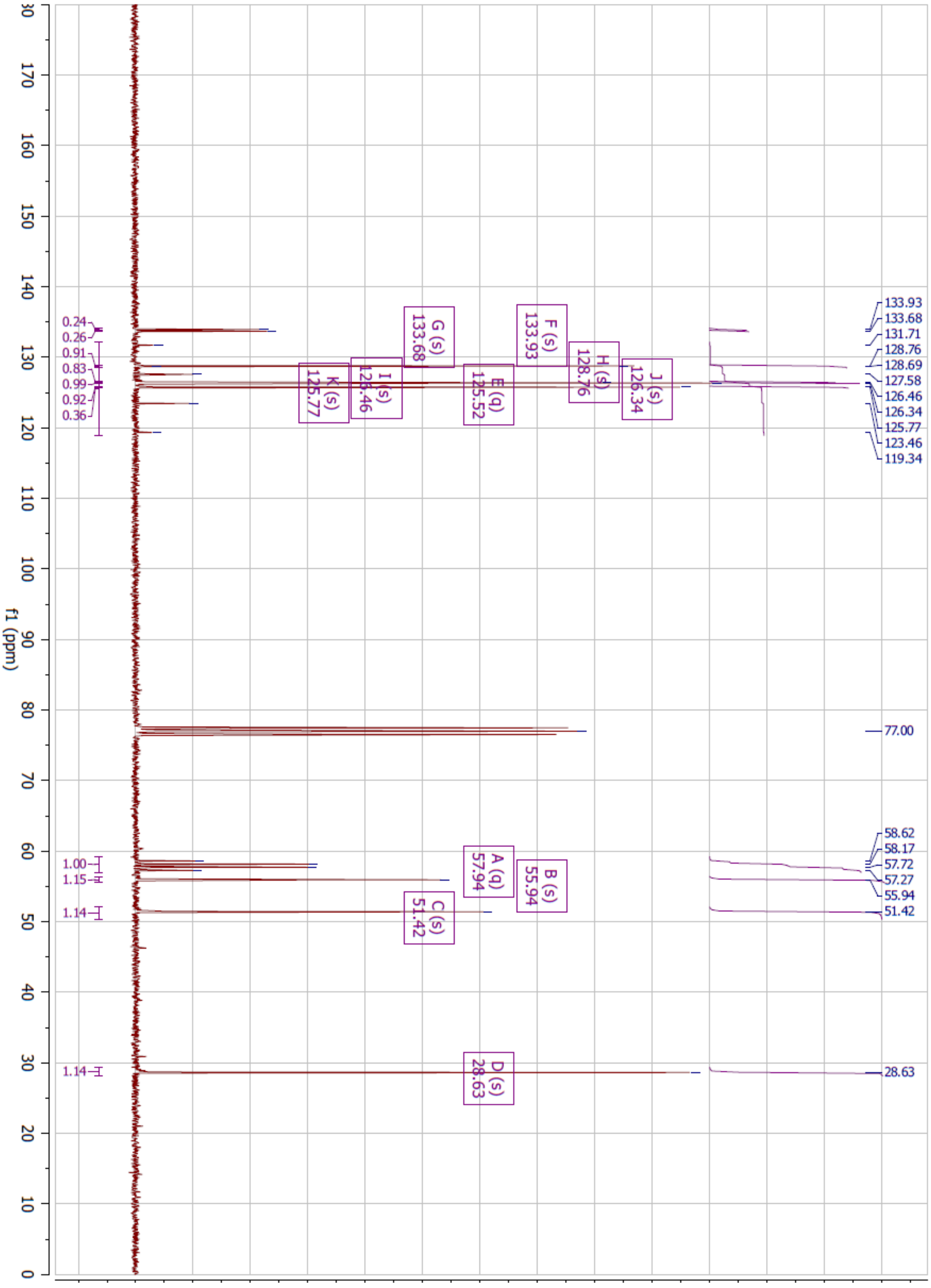

Supplementary Figure 22.

^{19}F-NMR (376 MHz, $CDCl_3$) 2-(2,2,2-trifluoroethyl)-1,2,3,4-tetrahydroisoquinoline (**4**)

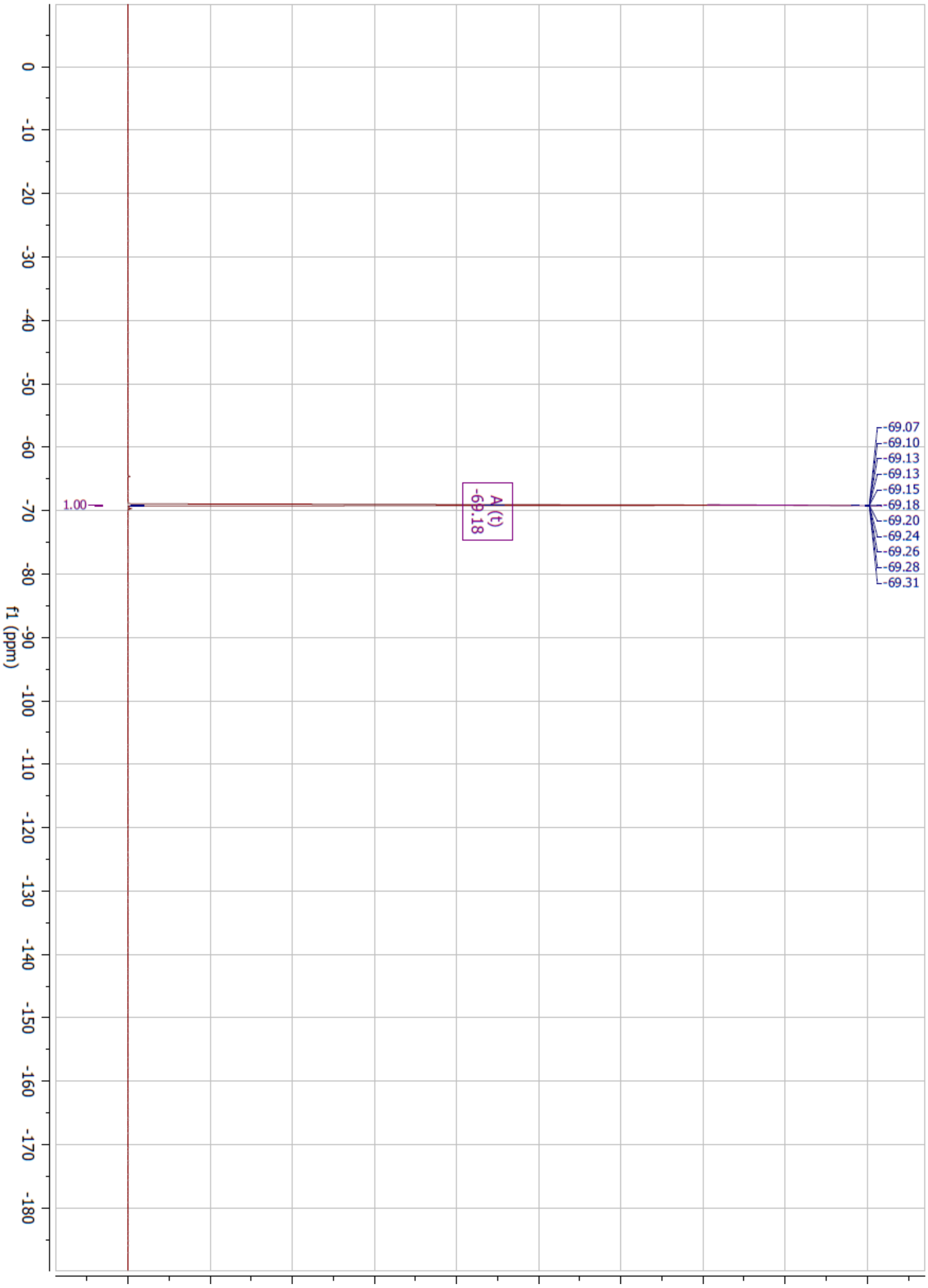

Supplementary Figure 23.

^{1}H-NMR (400 MHz, $CDCl_3$) 1-methyl-4-(2,2,2-trifluoroethyl)piperazine (**5**)

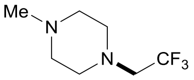

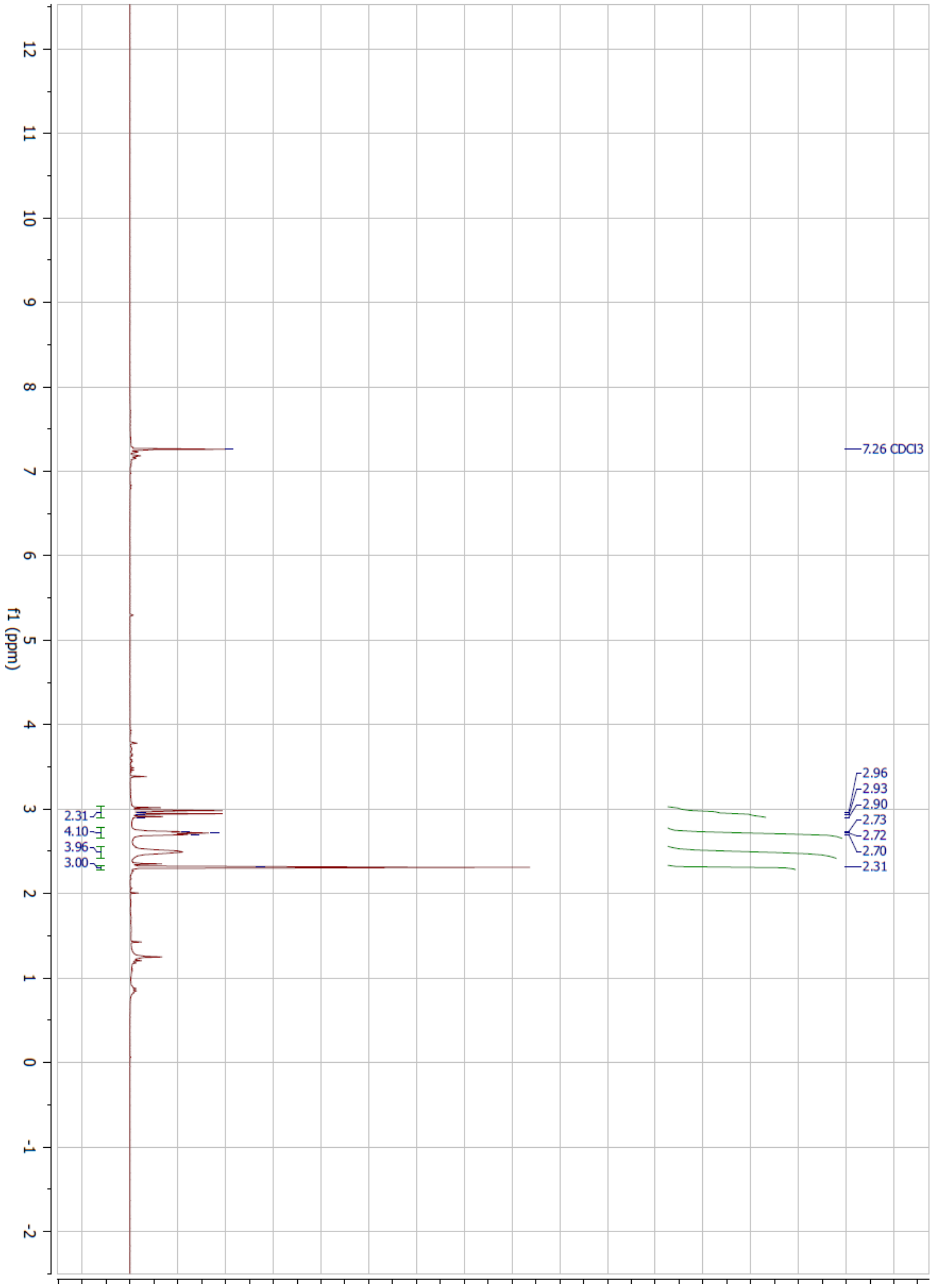

Supplementary Figure 24.

^{13}C-NMR (101 MHz, $CDCl_3$) 1-methyl-4-(2,2,2-trifluoroethyl)piperazine (**5**)

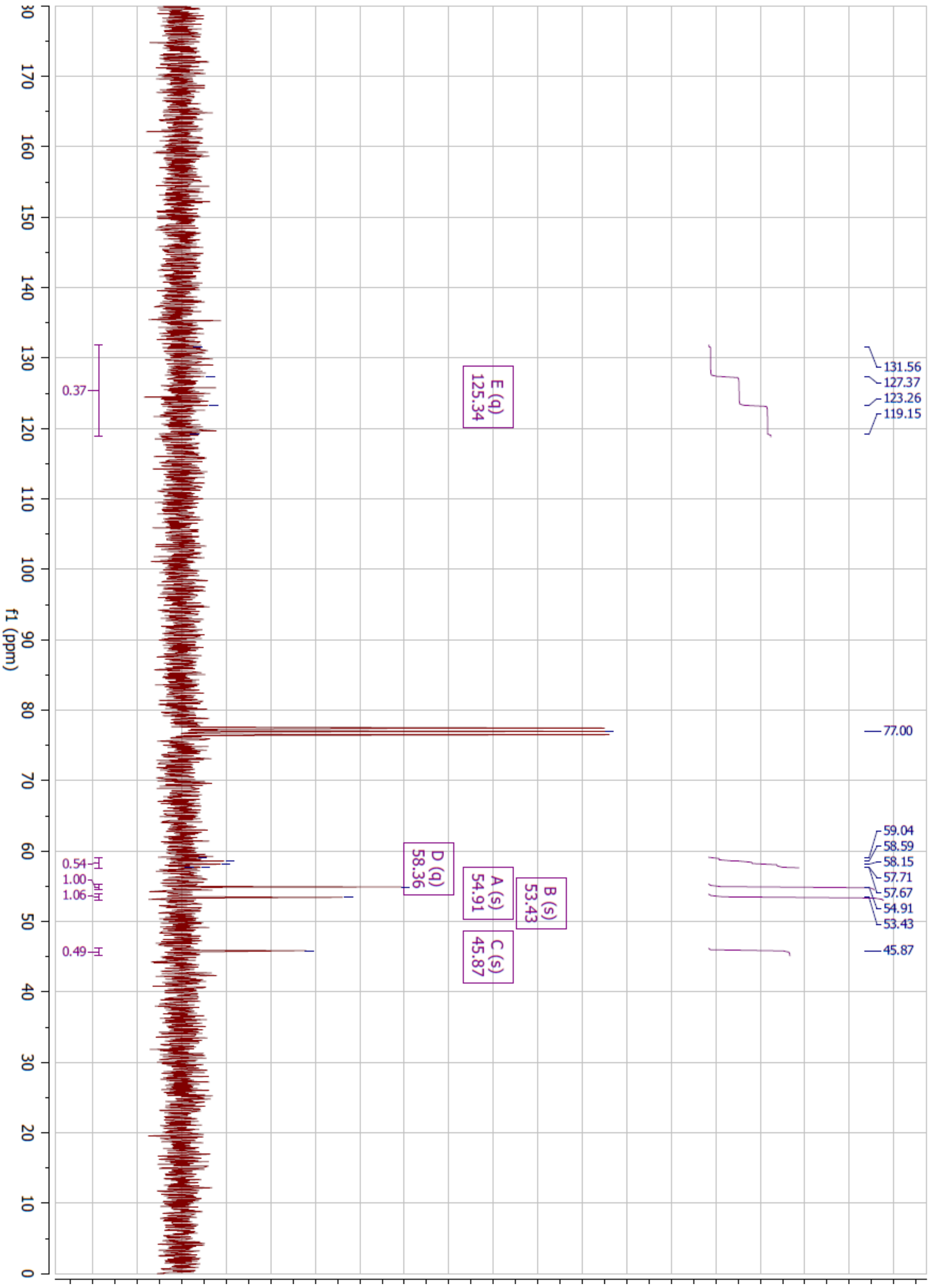

Supplementary Figure 25.
^{19}F-NMR (376 MHz, $CDCl_3$) 1-methyl-4-(2,2,2-trifluoroethyl)piperazine (**5**)

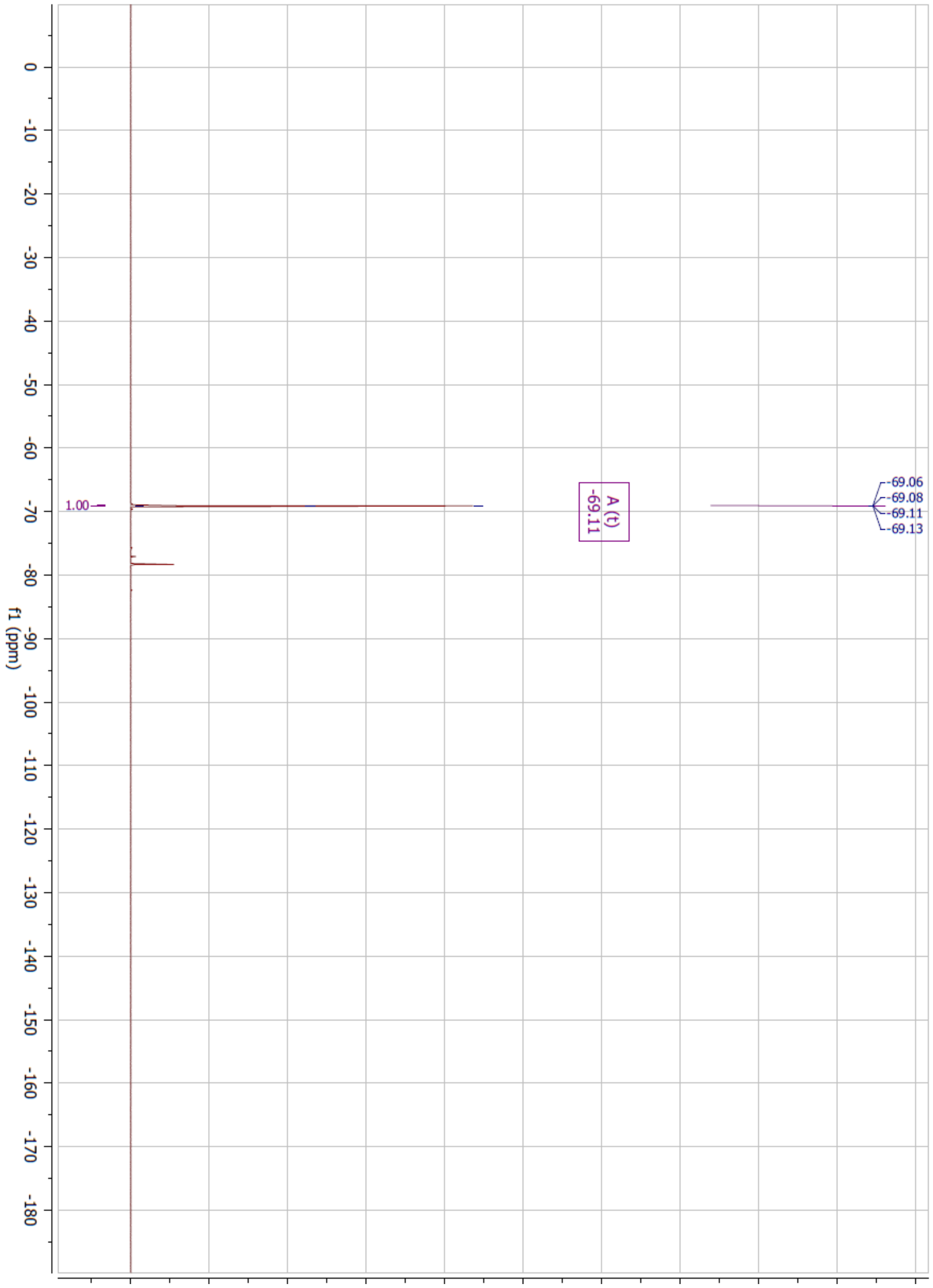

Supplementary Figure 26.

^{1}H-NMR (400 MHz, $CDCl_3$) 4-(2,2,2-trifluoroethyl)morpholine (**6**)

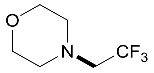

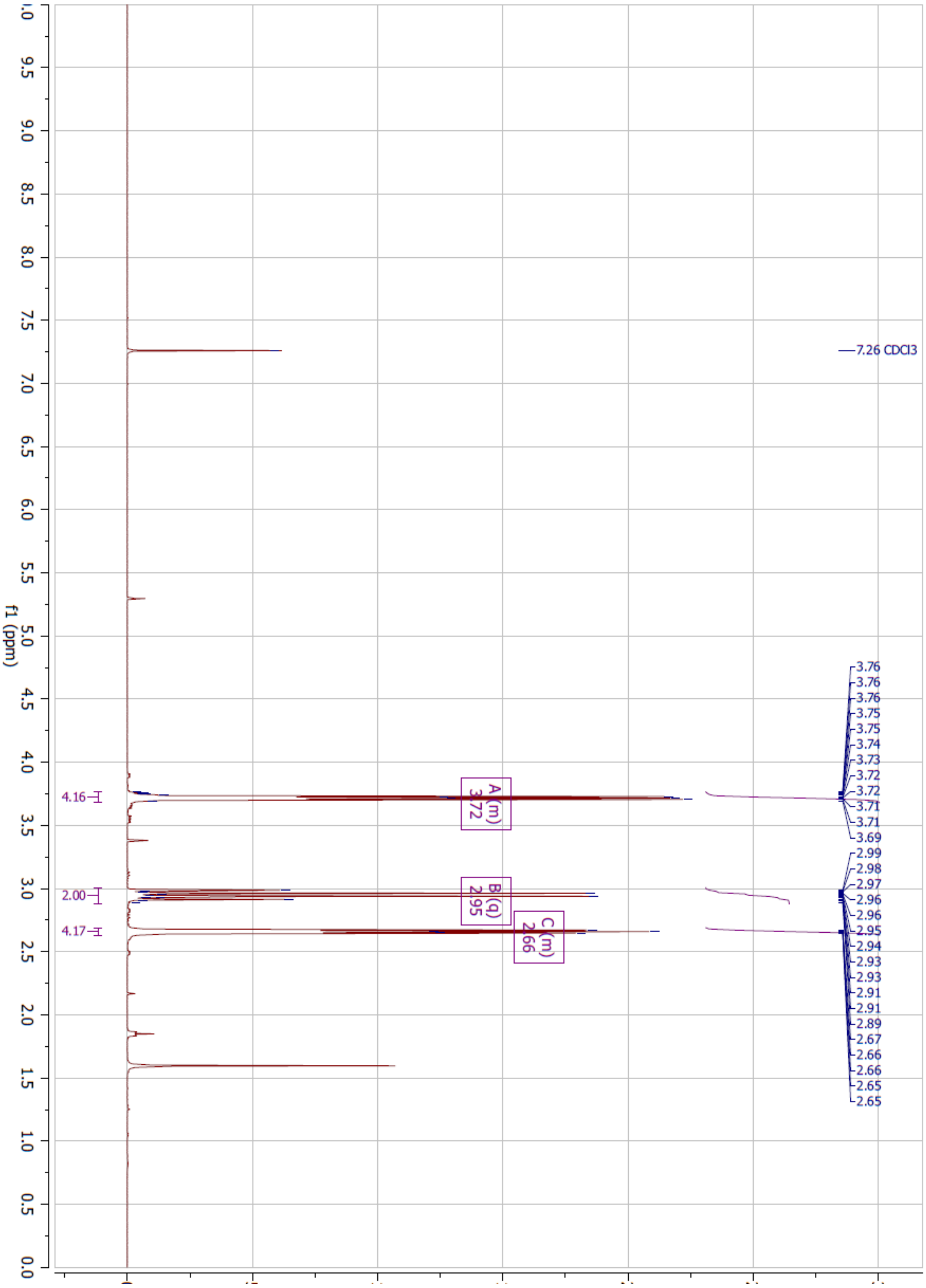

Supplementary Figure 27.

^{13}C-NMR (101 MHz, $CDCl_3$) 4-(2,2,2-trifluoroethyl)morpholine (**6**)

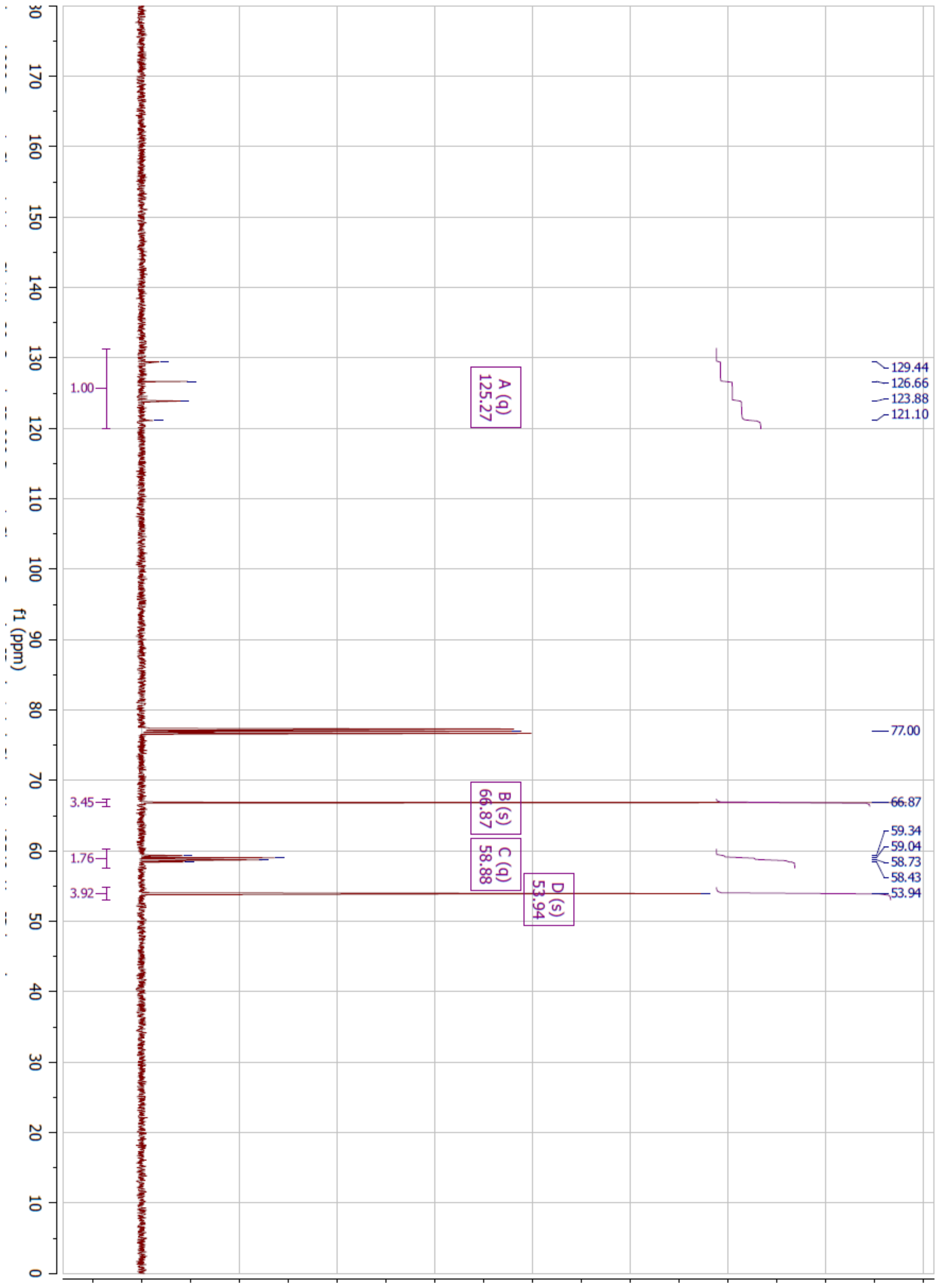

Supplementary Figure 28.

^{19}F-NMR (376 MHz, $CDCl_3$) 4-(2,2,2-trifluoroethyl)morpholine (**6**)

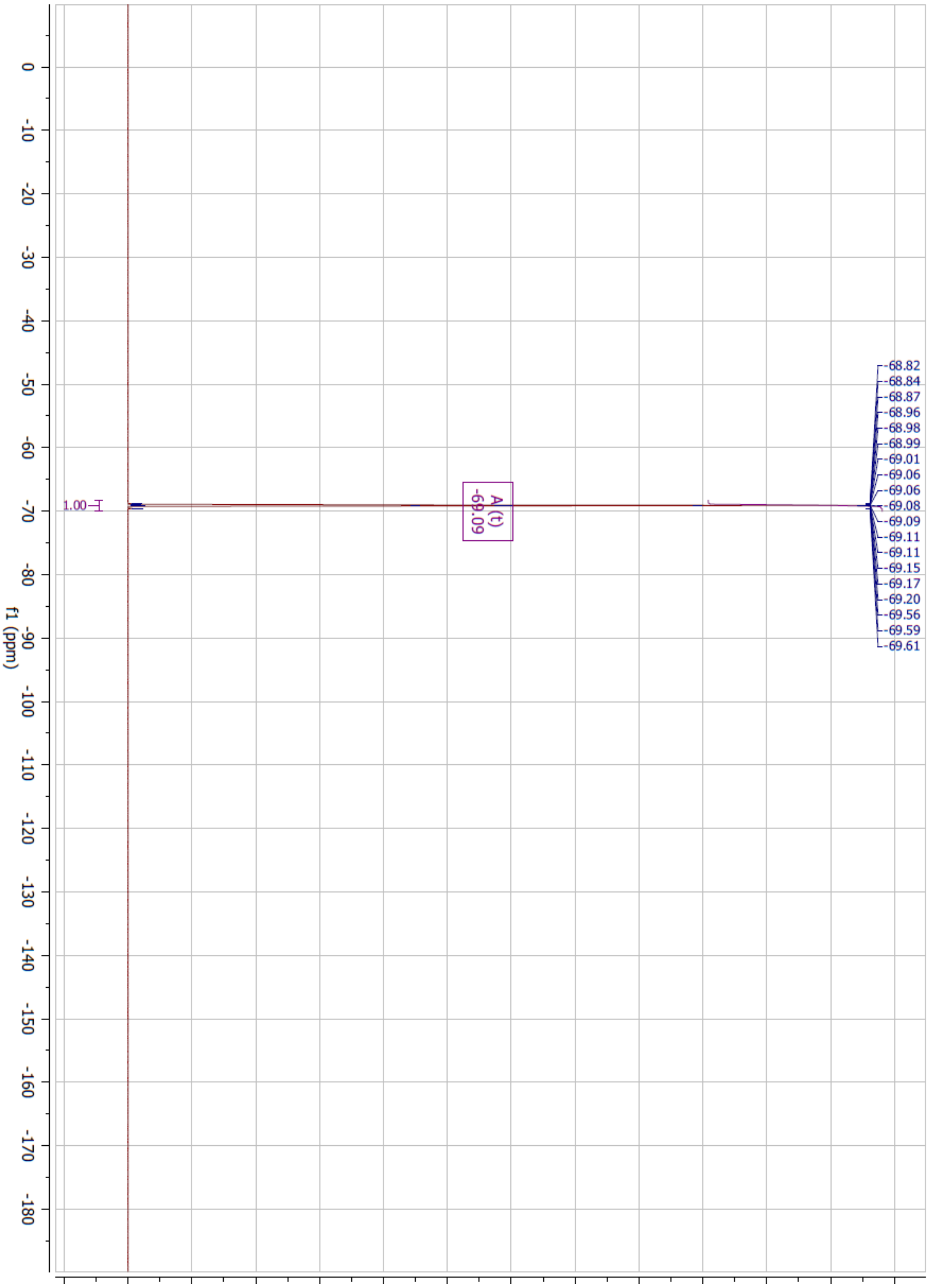

Supplementary Figure 29.

^{1}H-NMR (400 MHz, $CDCl_3$) 4-((tert-butyldimethylsilyl)oxy)-1-(2,2,2-trifluoroethyl)piperidine (**7**)

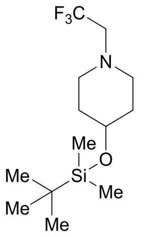

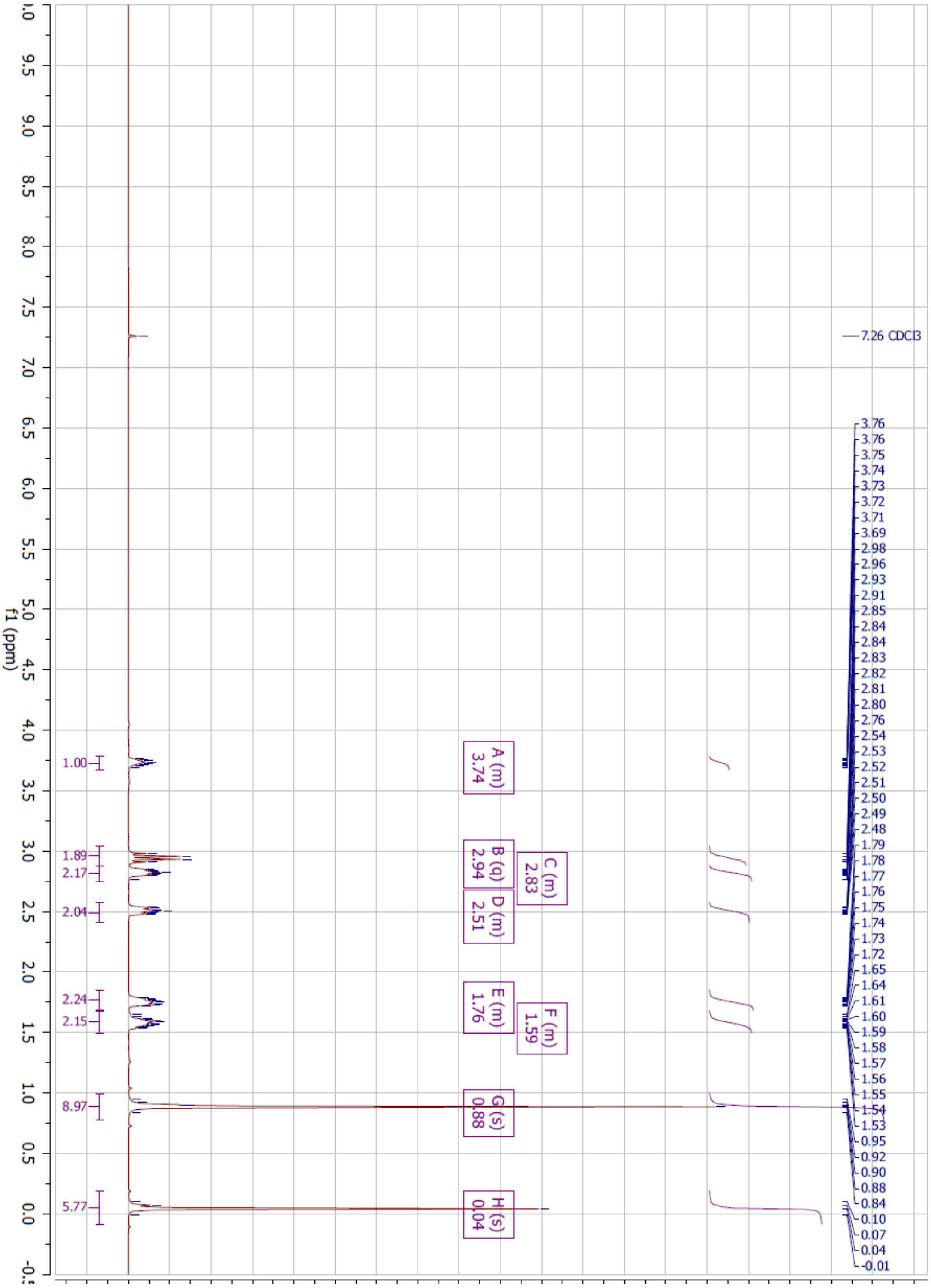

Supplementary Figure 30.

^{13}C-NMR (101 MHz, $CDCl_3$) 4-((tert-butyldimethylsilyl)oxy)-1-(2,2,2-trifluoroethyl)piperidine (**7**)

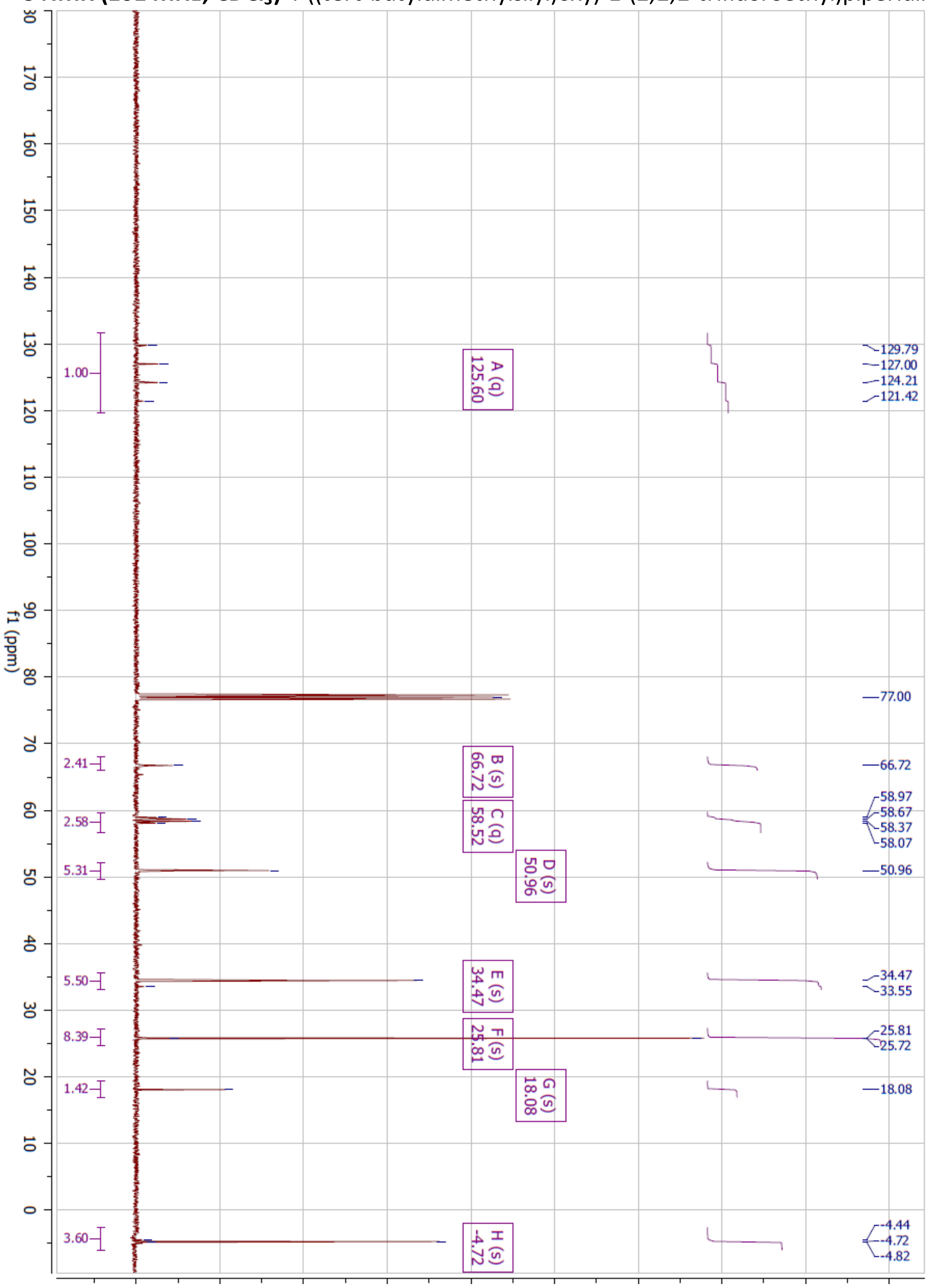

Supplementary Figure 31.
^{19}F-NMR (376 MHz, $CDCl_3$) 4-((tert-butyldimethylsilyl)oxy)-1-(2,2,2-trifluoroethyl)piperidine (**7**)

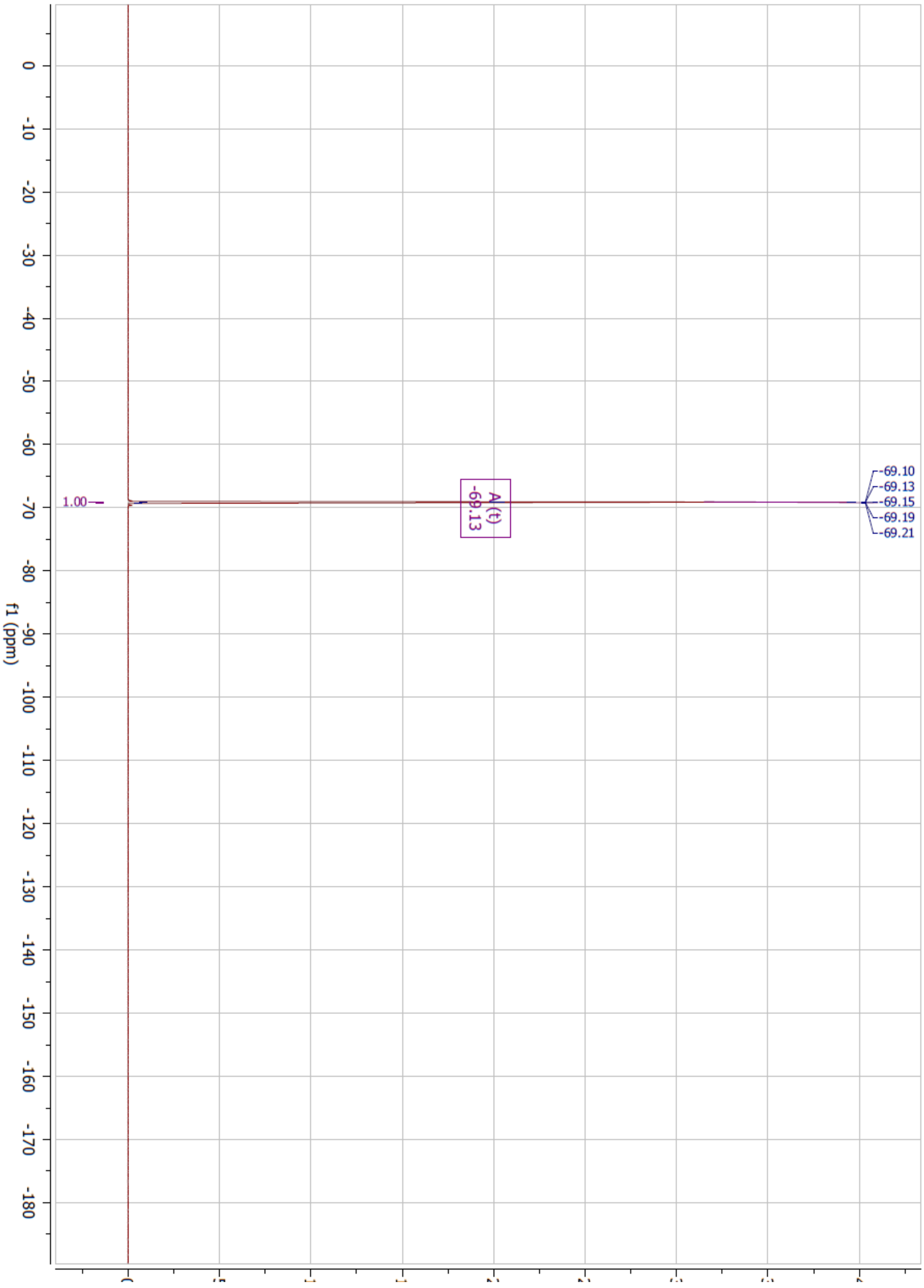

Supplementary Figure 32.

^{1}H-NMR (400 MHz, $CDCl_3$) ethyl 1-(2,2,2-trifluoroethyl)piperidine-2-carboxylate (**8**)

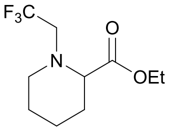

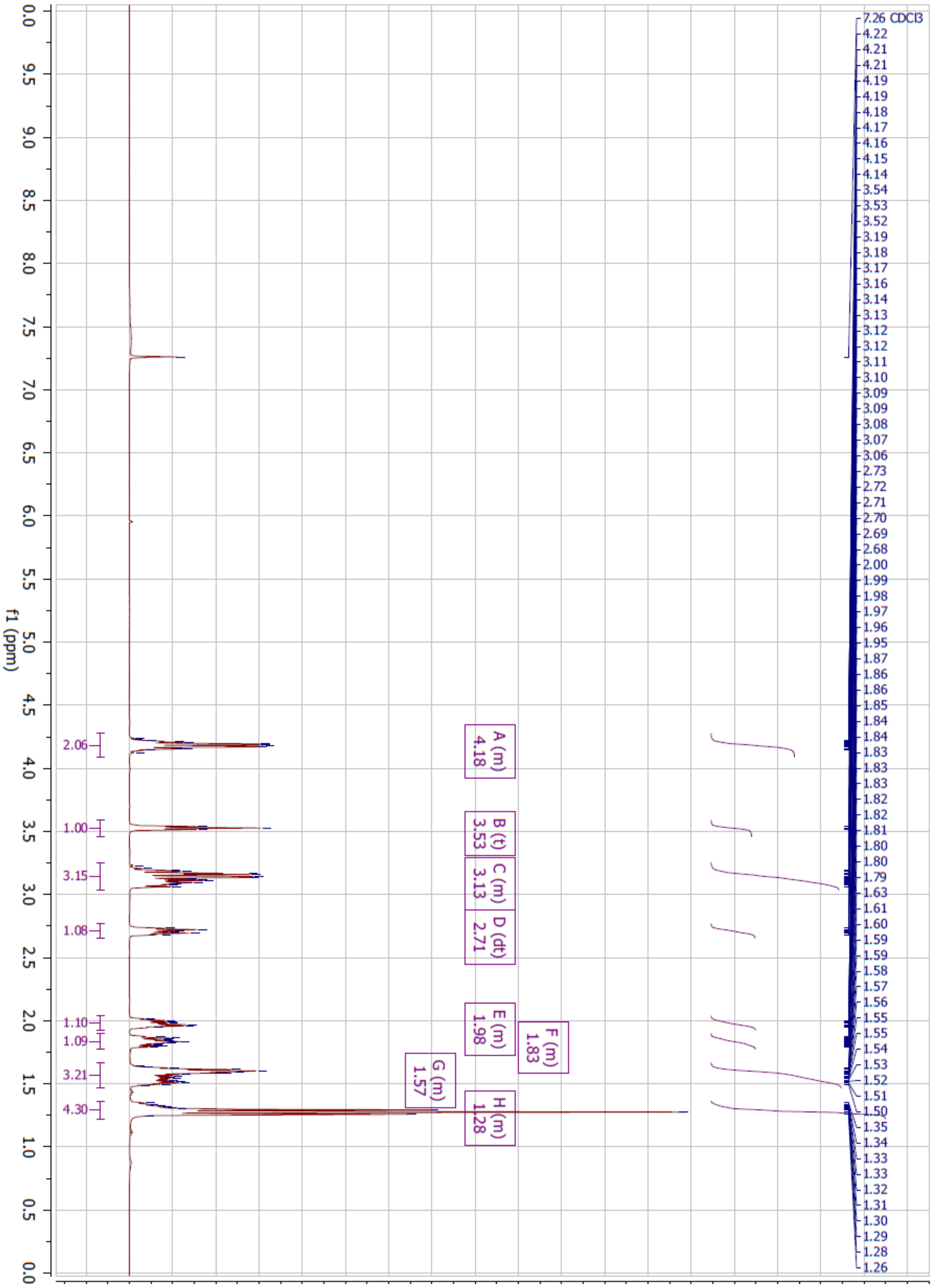

Supplementary Figure 33.

^{13}C-NMR (101 MHz, $CDCl_3$) ethyl 1-(2,2,2-trifluoroethyl)piperidine-2-carboxylate (**8**)

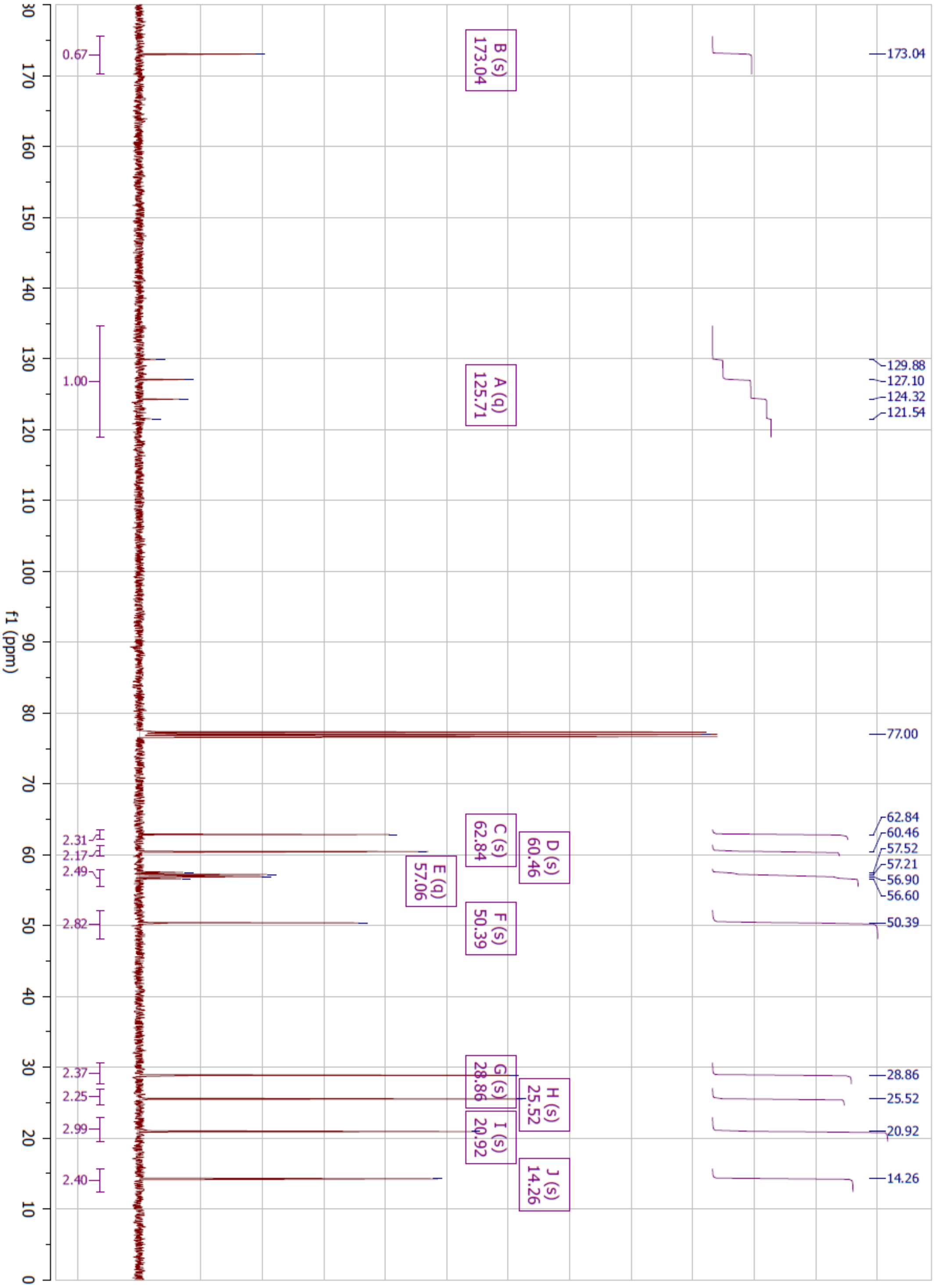

Supplementary Figure 34.

^{19}F-NMR (376 MHz, $CDCl_3$) ethyl 1-(2,2,2-trifluoroethyl)piperidine-2-carboxylate (**8**)

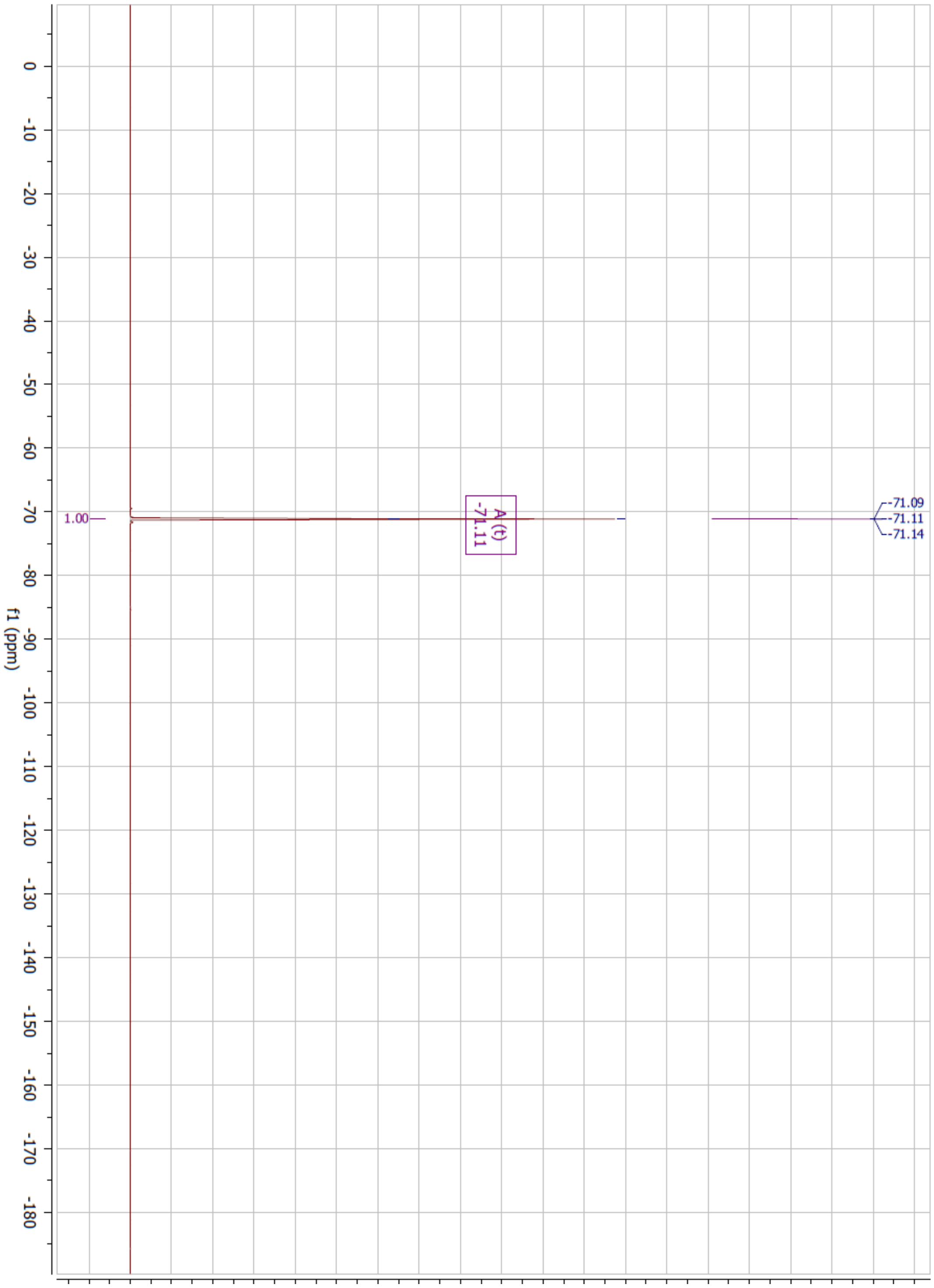

Supplementary Figure 35.

^{1}H-NMR (400 MHz, $CDCl_3$) 1-(2,2,2-trifluoroethyl)piperidin-4-ol (**9**)

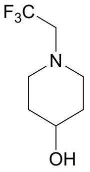

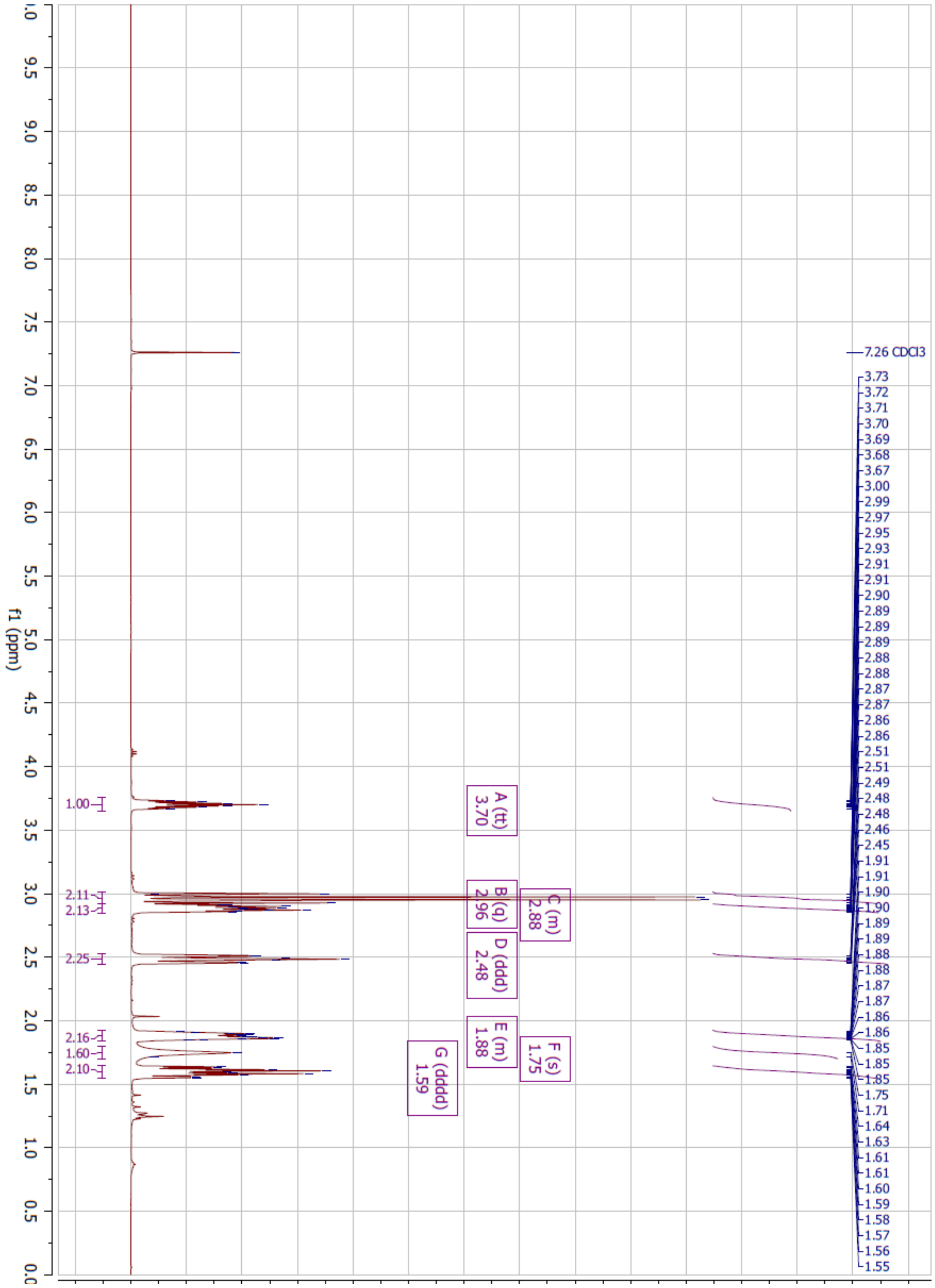

Supplementary Figure 36.

^{13}C-NMR (101 MHz, $CDCl_3$) 1-(2,2,2-trifluoroethyl)piperidin-4-ol (**9**)

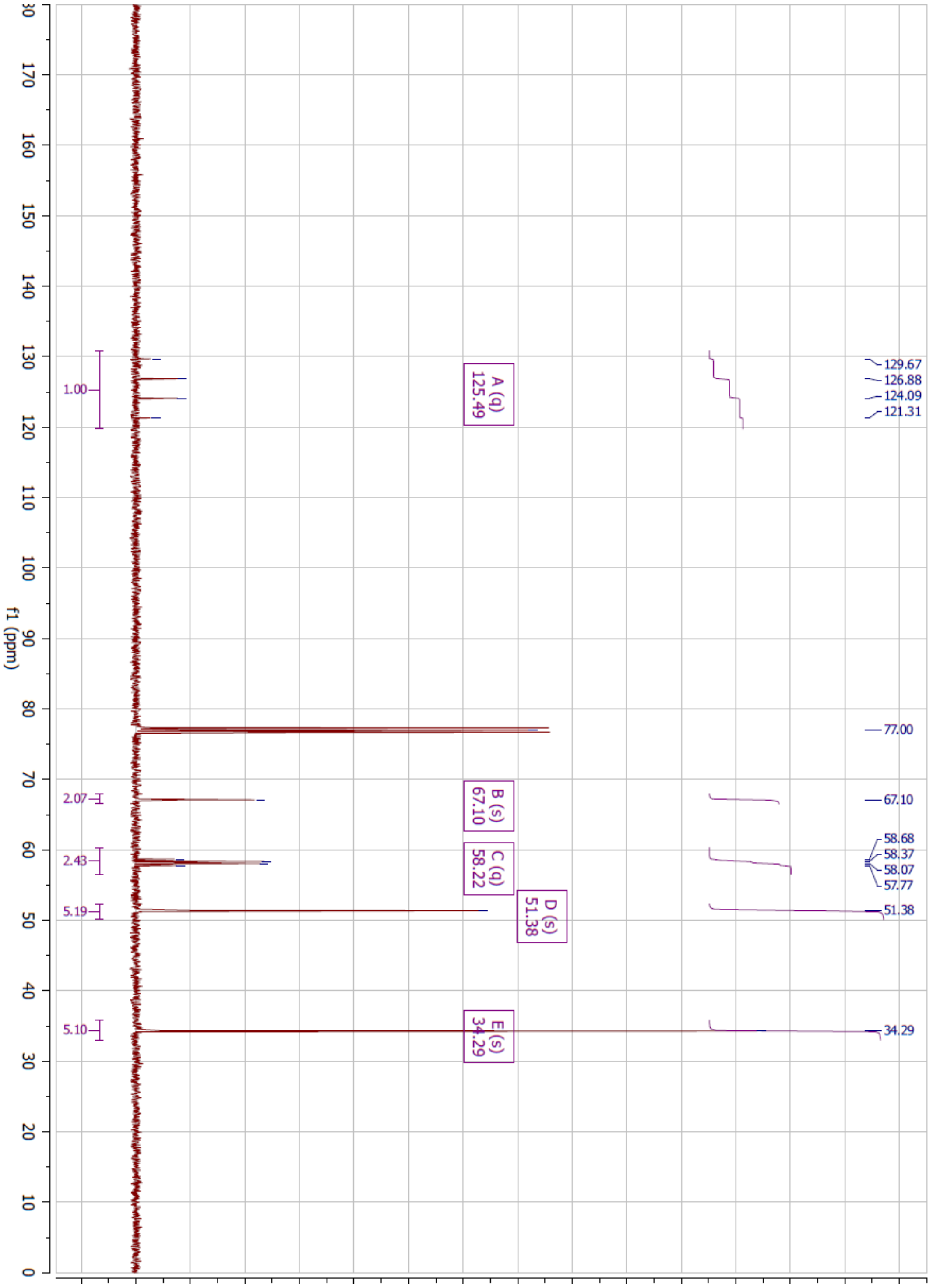

Supplementary Figure 37.

^{19}F-NMR (376 MHz, $CDCl_3$) 1-(2,2,2-trifluoroethyl)piperidin-4-ol (**9**)

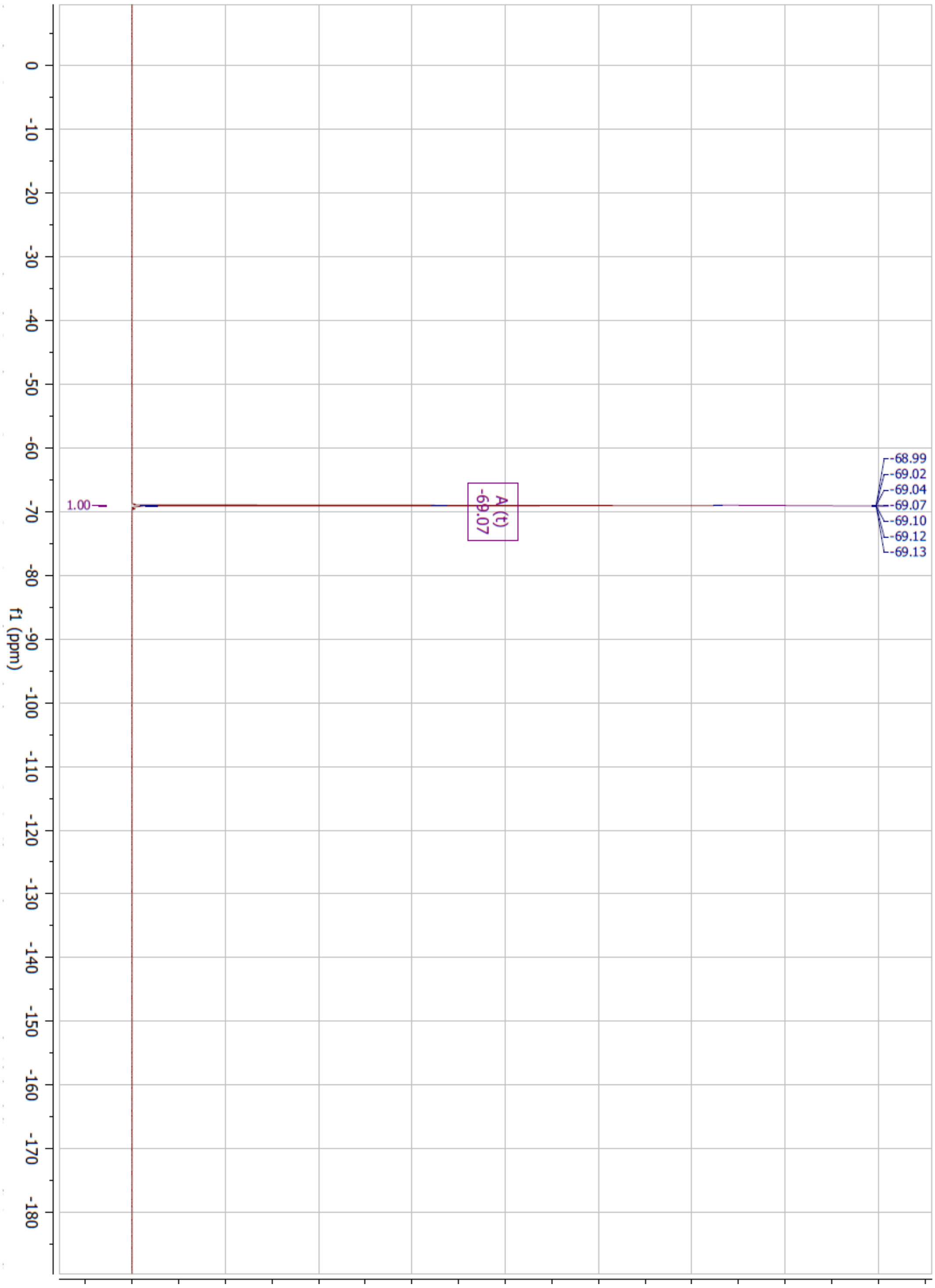

Supplementary Figure 38.

^{1}H-NMR (400 MHz, $CDCl_3$) *N*,*N*-dibenzyl-2,2,2-trifluoroethan-1-amine (**10**)

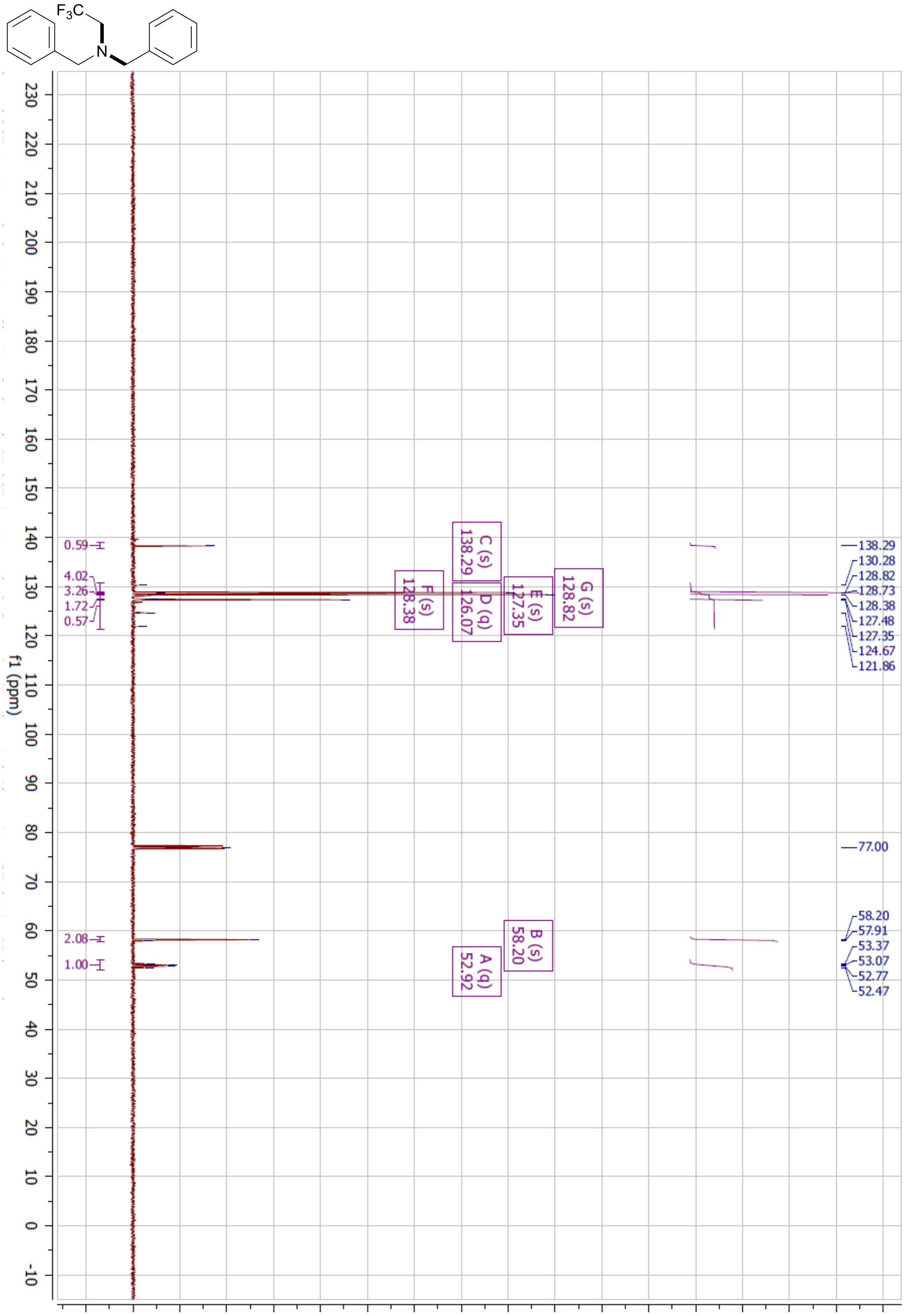

Supplementary Figure 39.

^{13}C-NMR (101 MHz, $CDCl_3$) *N*,*N*-dibenzyl-2,2,2-trifluoroethan-1-amine (**10**)

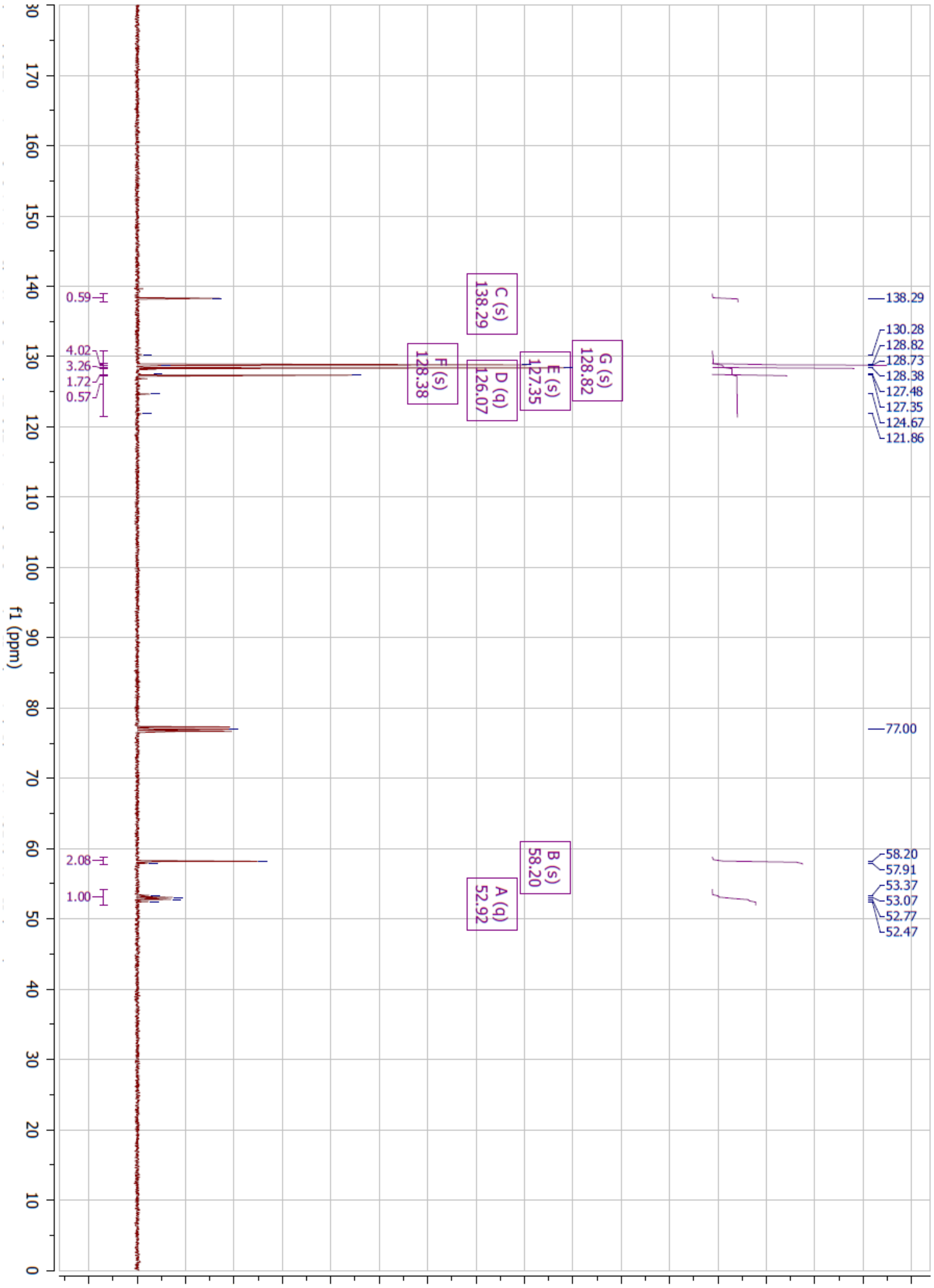

Supplementary Figure 40.

^{19}F-NMR (376 MHz, $CDCl_3$) *N*,*N*-dibenzyl-2,2,2-trifluoroethan-1-amine (**10**)

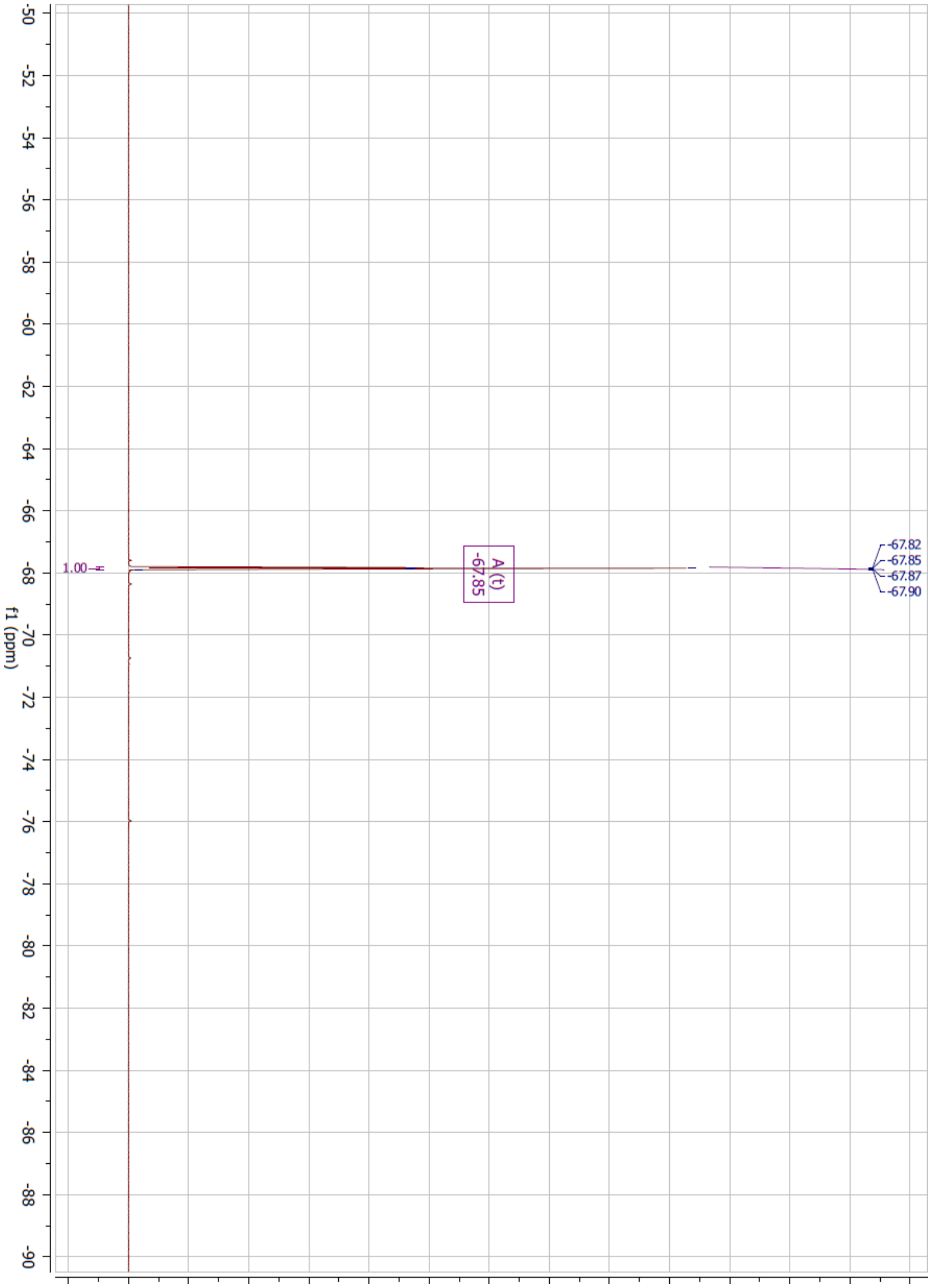

Supplementary Figure 41.

^{1}H-NMR (400 MHz, $CDCl_3$) *N*-(4-nitrobenzyl)-3-phenyl-*N*-(2,2,2-trifluoroethyl)propan-1-amine (**11**)

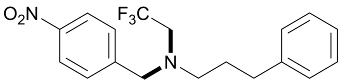

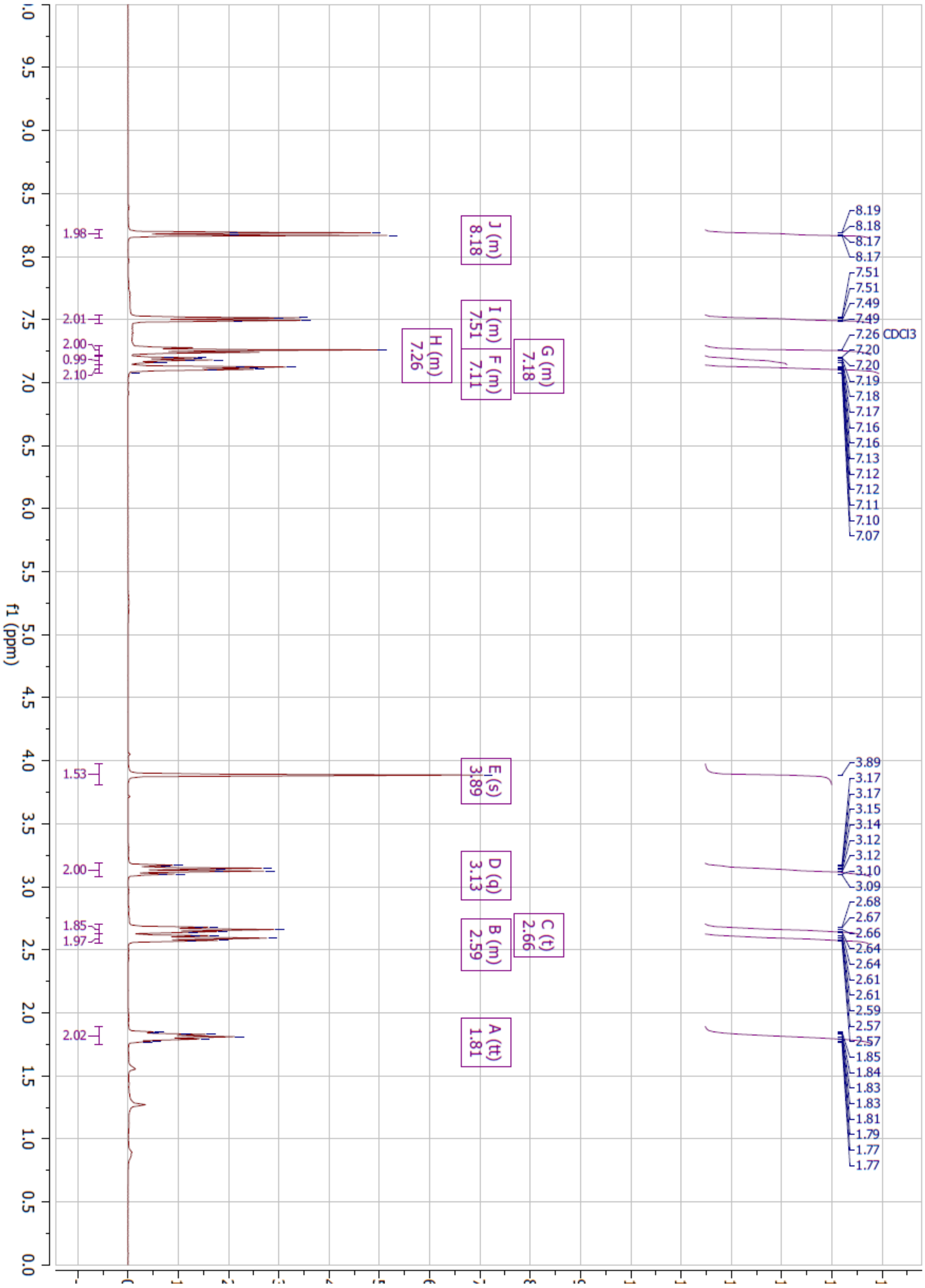

Supplementary Figure 42.

^{13}C-NMR (101 MHz, $CDCl_3$) *N*-(4-nitrobenzyl)-3-phenyl-*N*-(2,2,2-trifluoroethyl)propan-1-amine (**11**)

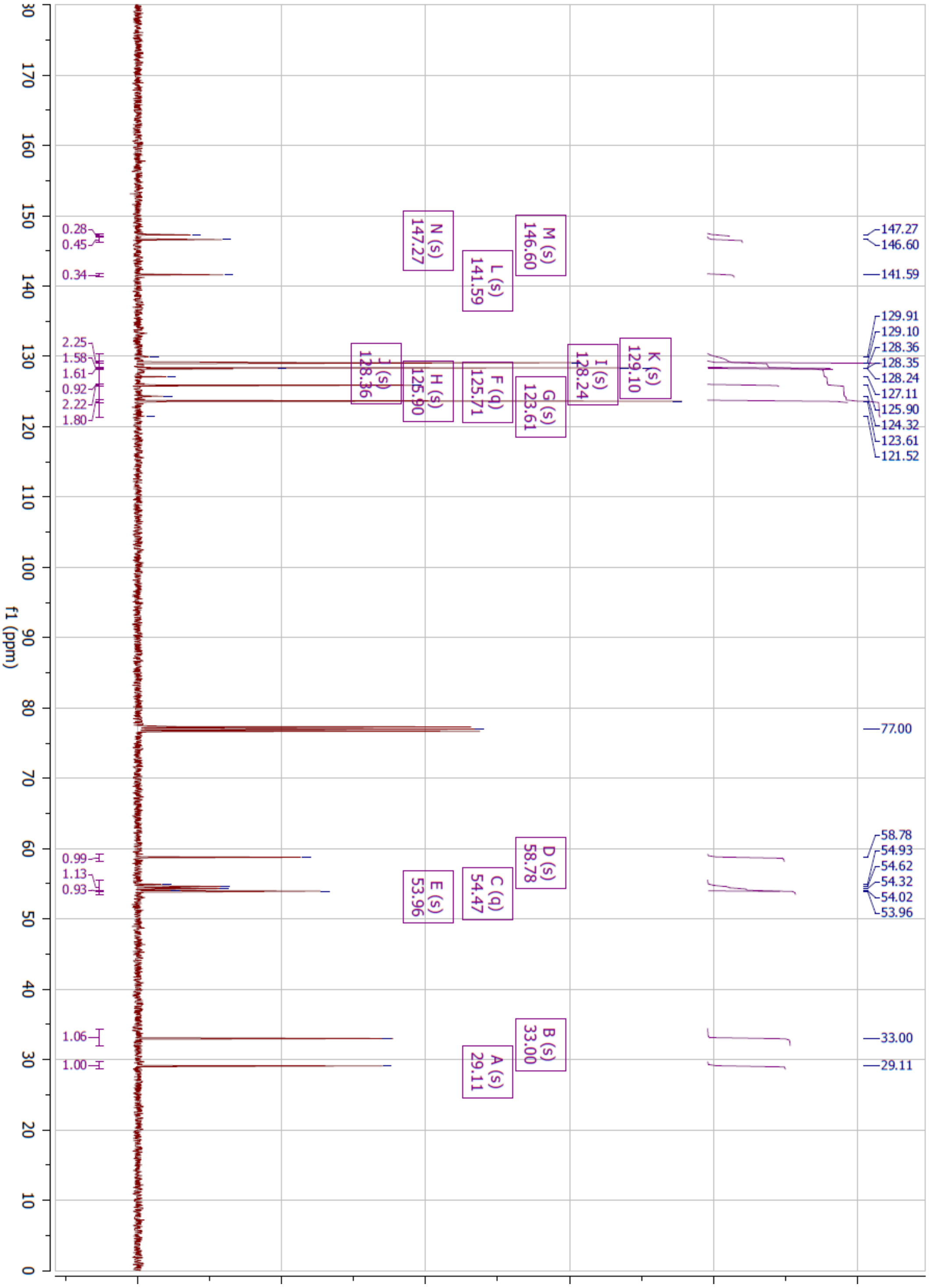

Supplementary Figure 43.

^{19}F-NMR (376 MHz, $CDCl_3$) *N*-(4-nitrobenzyl)-3-phenyl-*N*-(2,2,2-trifluoroethyl)propan-1-amine (**11**)

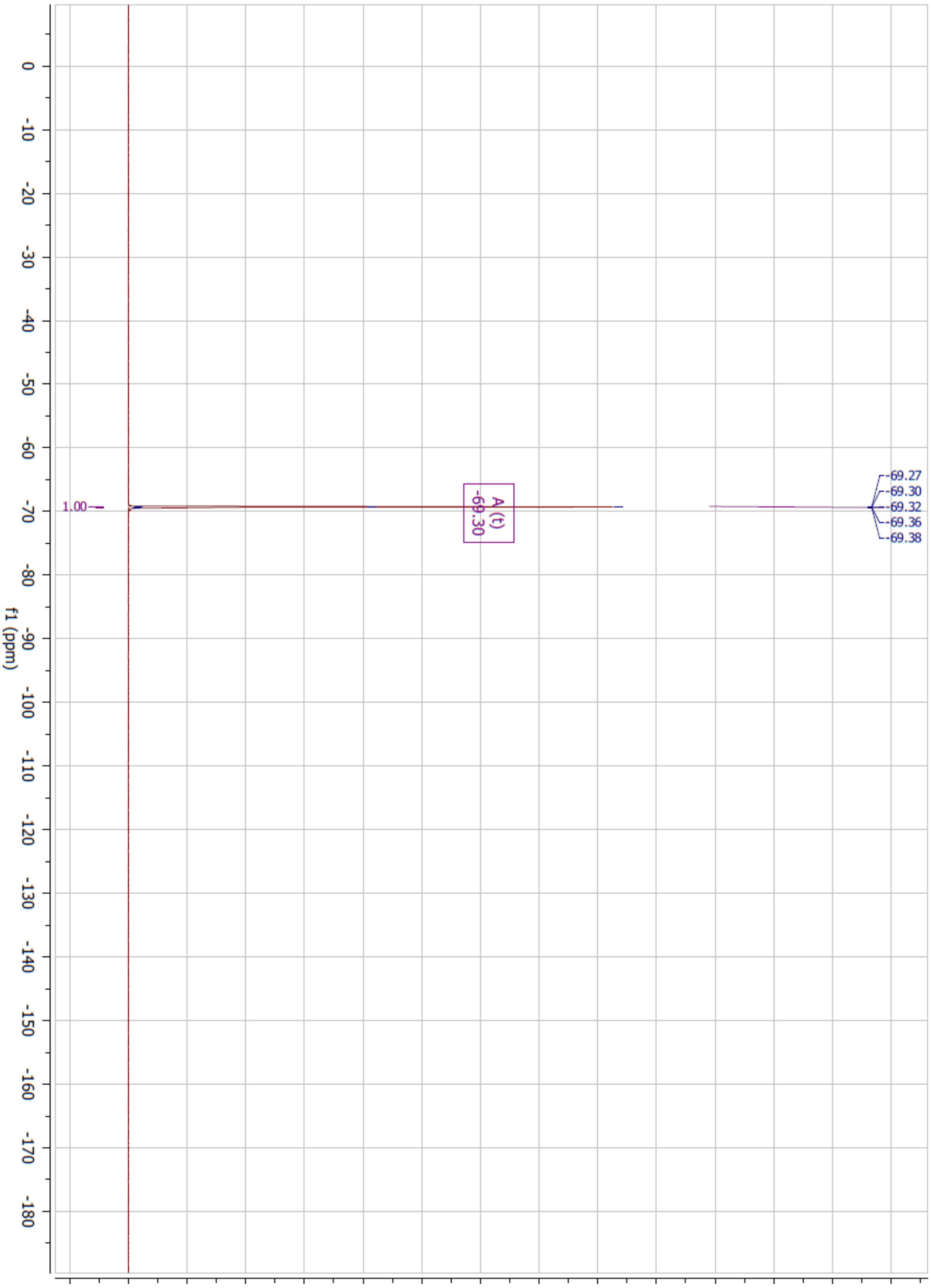

Supplementary Figure 44.

^{1}H-NMR (400 MHz, $CDCl_3$) 4-(((3-phenylpropyl)(2,2,2-trifluoroethyl)amino)methyl)benzonitrile (**12**)

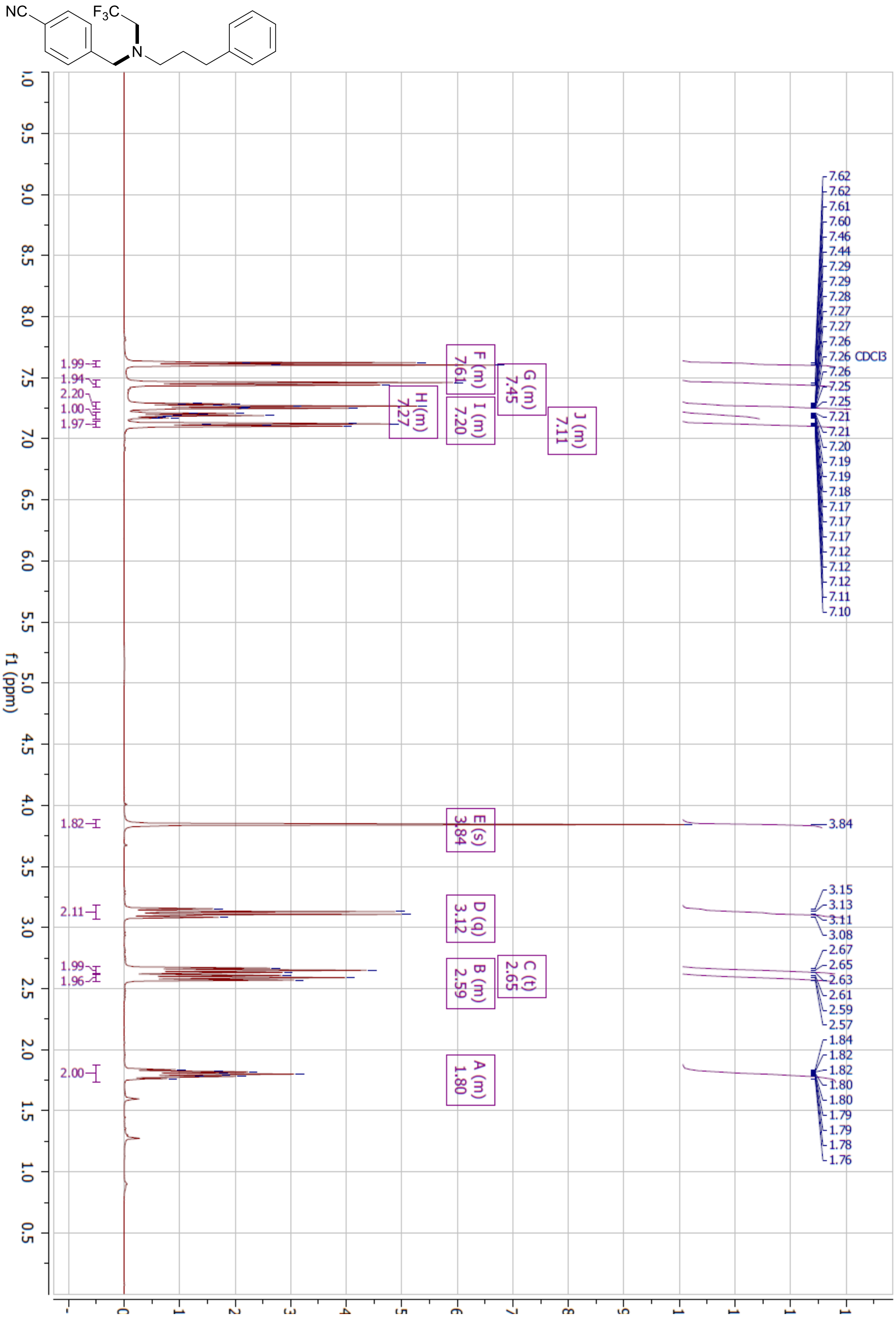

Supplementary Figure 45.

^{13}C-NMR (101 MHz, $CDCl_3$) 4-(((3-phenylpropyl)(2,2,2-trifluoroethyl)amino)methyl)benzonitrile (**12**)

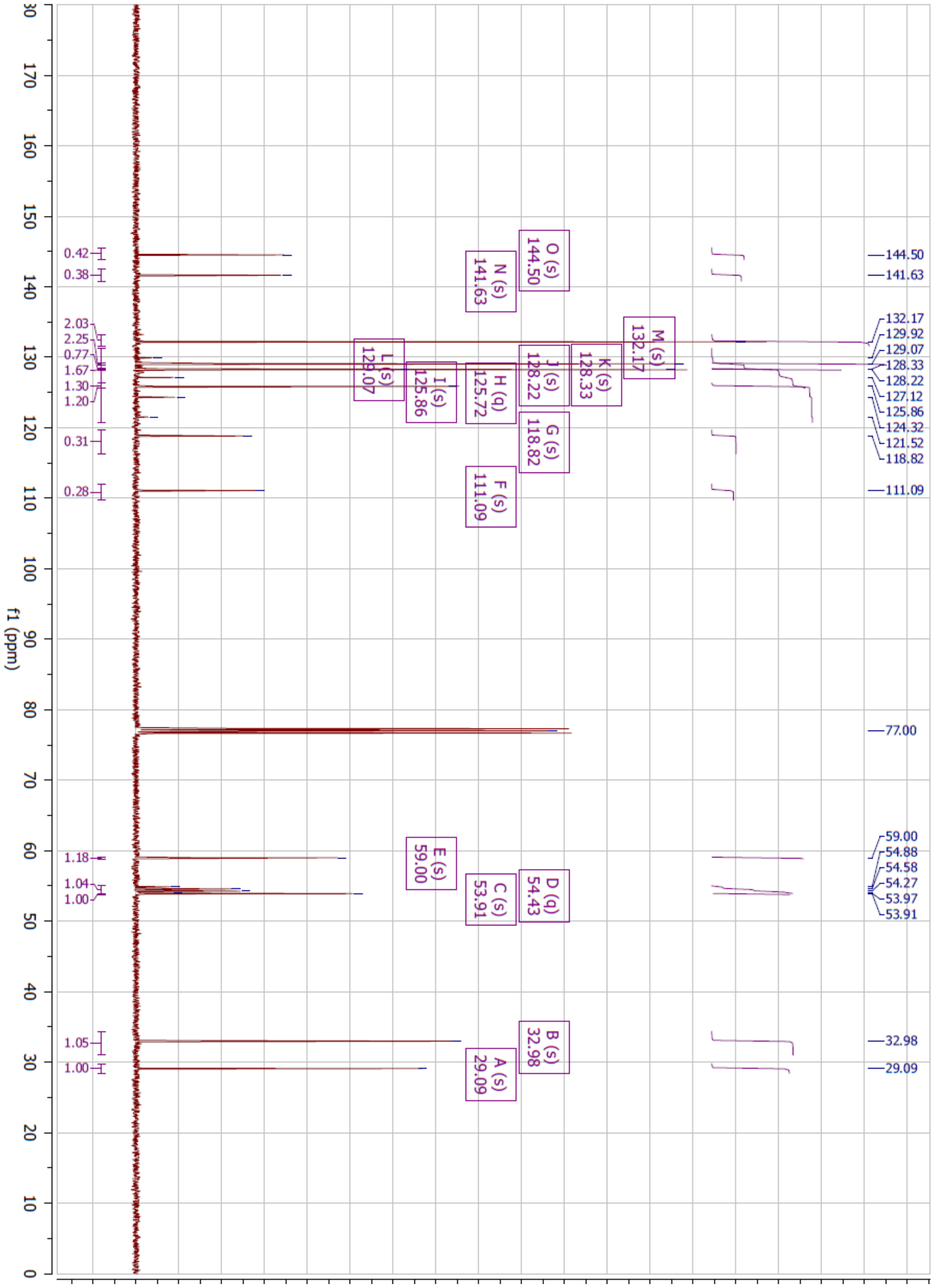

Supplementary Figure 46.

^{19}F-NMR (376 MHz, $CDCl_3$) 4-(((3-phenylpropyl)(2,2,2-trifluoroethyl)amino)methyl)benzonitrile (**12**)

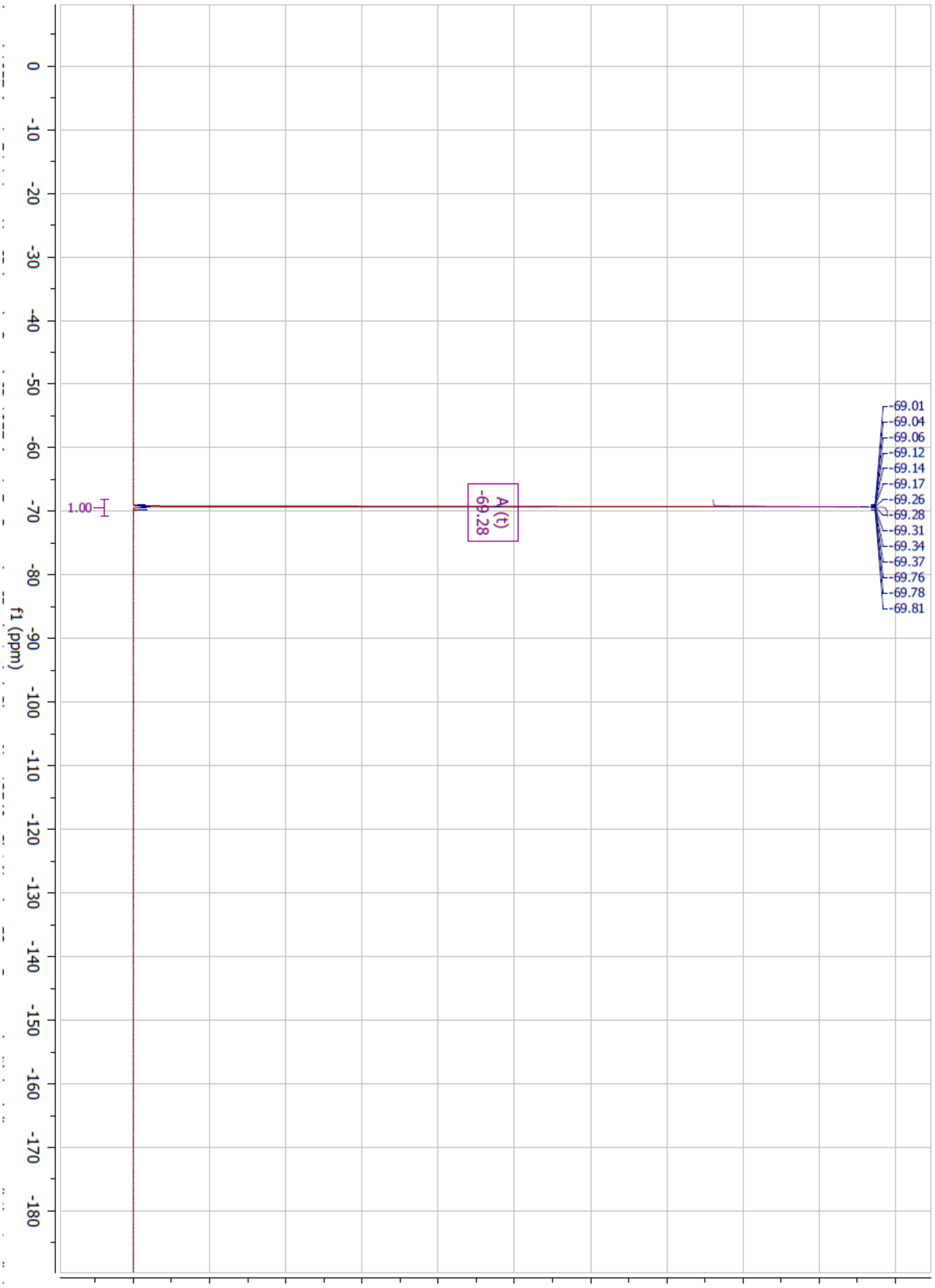

Supplementary Figure 47.

^{1}H-NMR (400 MHz, $CDCl_3$) *N*-(4-methoxybenzyl)-3-phenyl-*N*-(2,2,2-trifluoroethyl)propan-1-amine (**13**)

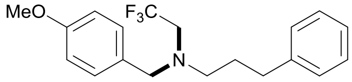

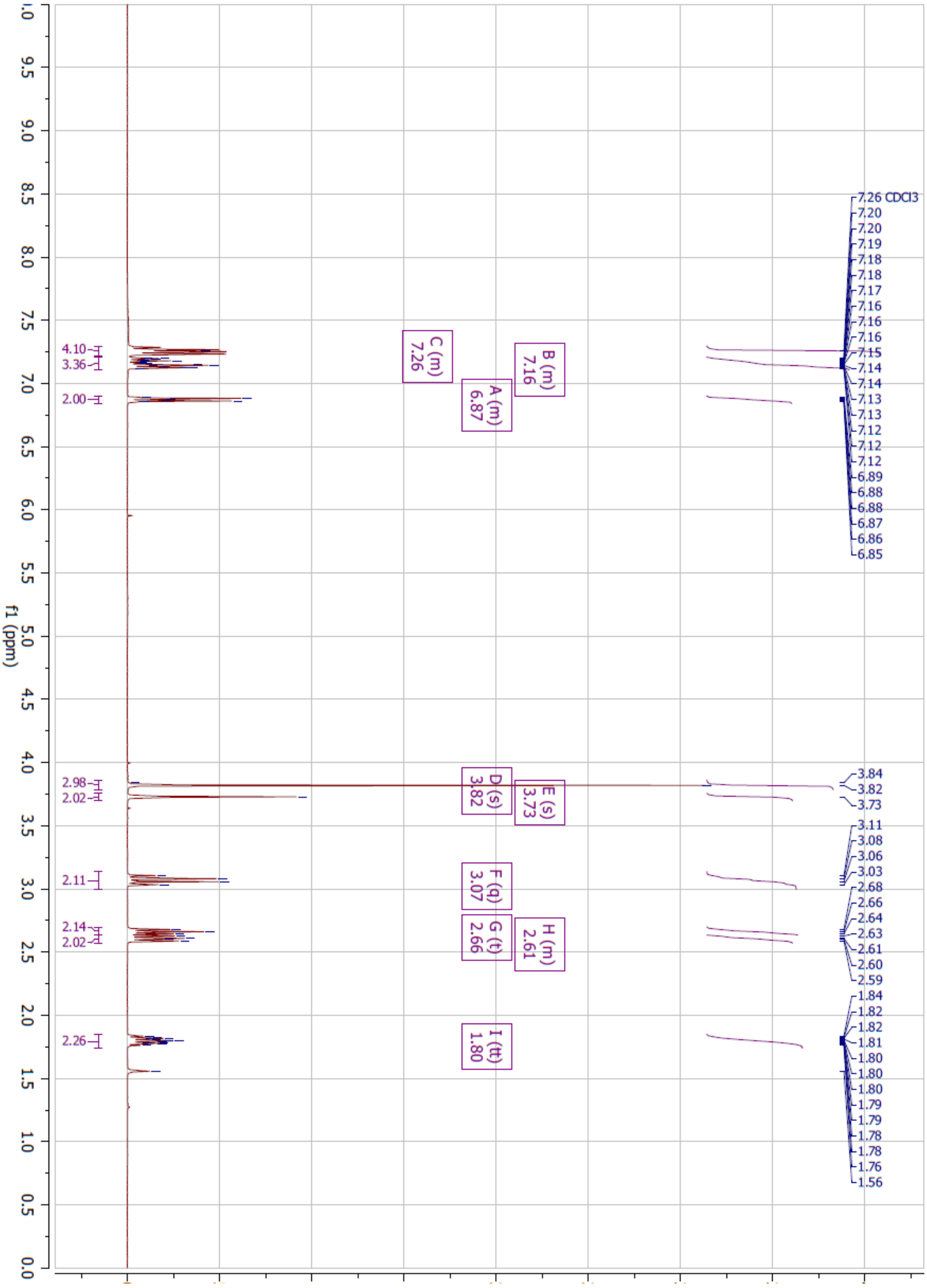

Supplementary Figure 48.

^{13}C-NMR (101 MHz, $CDCl_3$) *N*-(4-methoxybenzyl)-3-phenyl-*N*-(2,2,2-trifluoroethyl)propan-1-amine (**13**)

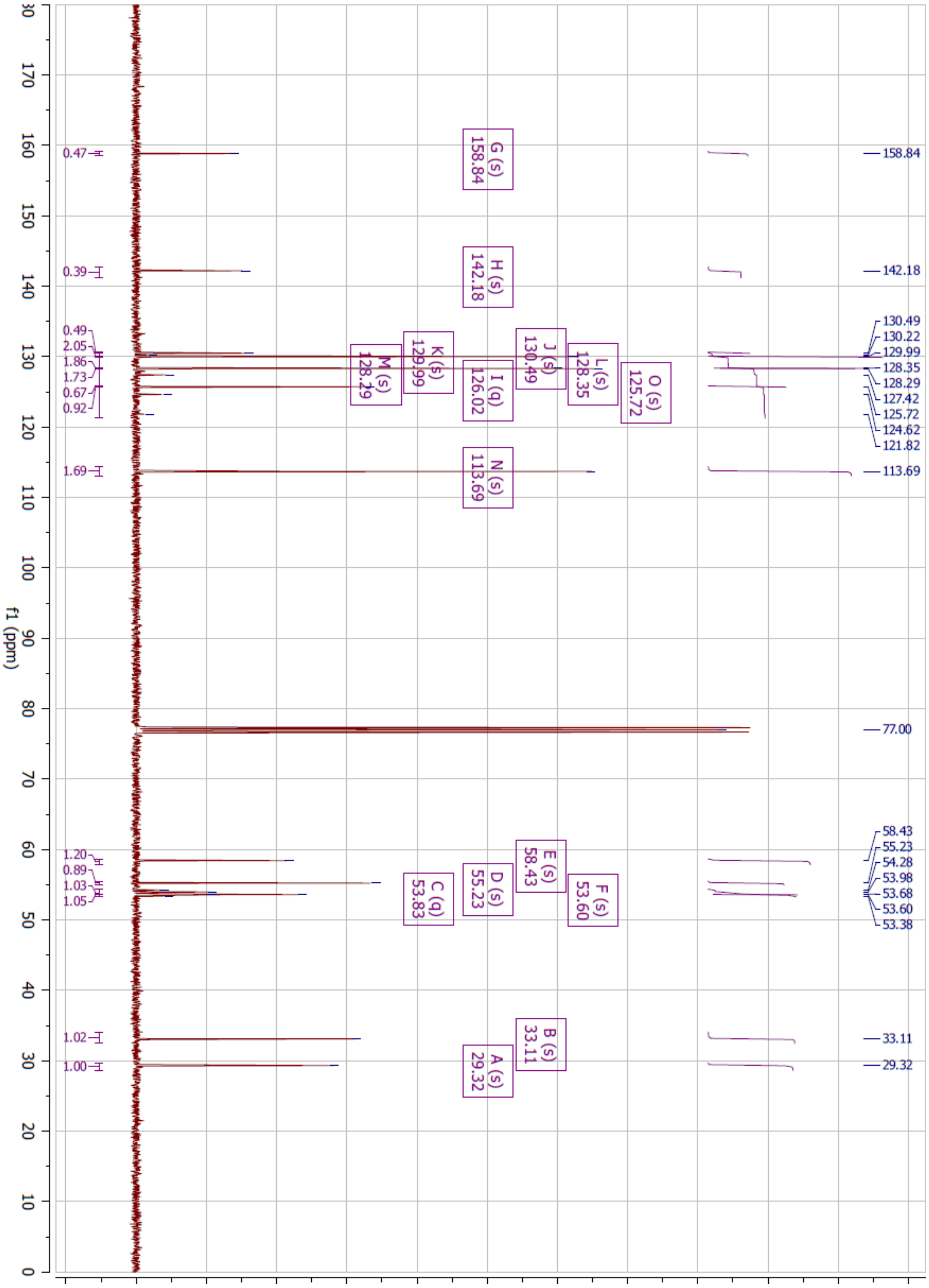

Supplementary Figure 49.

^{19}F-NMR (376 MHz, $CDCl_3$) *N*-(4-methoxybenzyl)-3-phenyl-*N*-(2,2,2-trifluoroethyl)propan-1-amine (**13**)

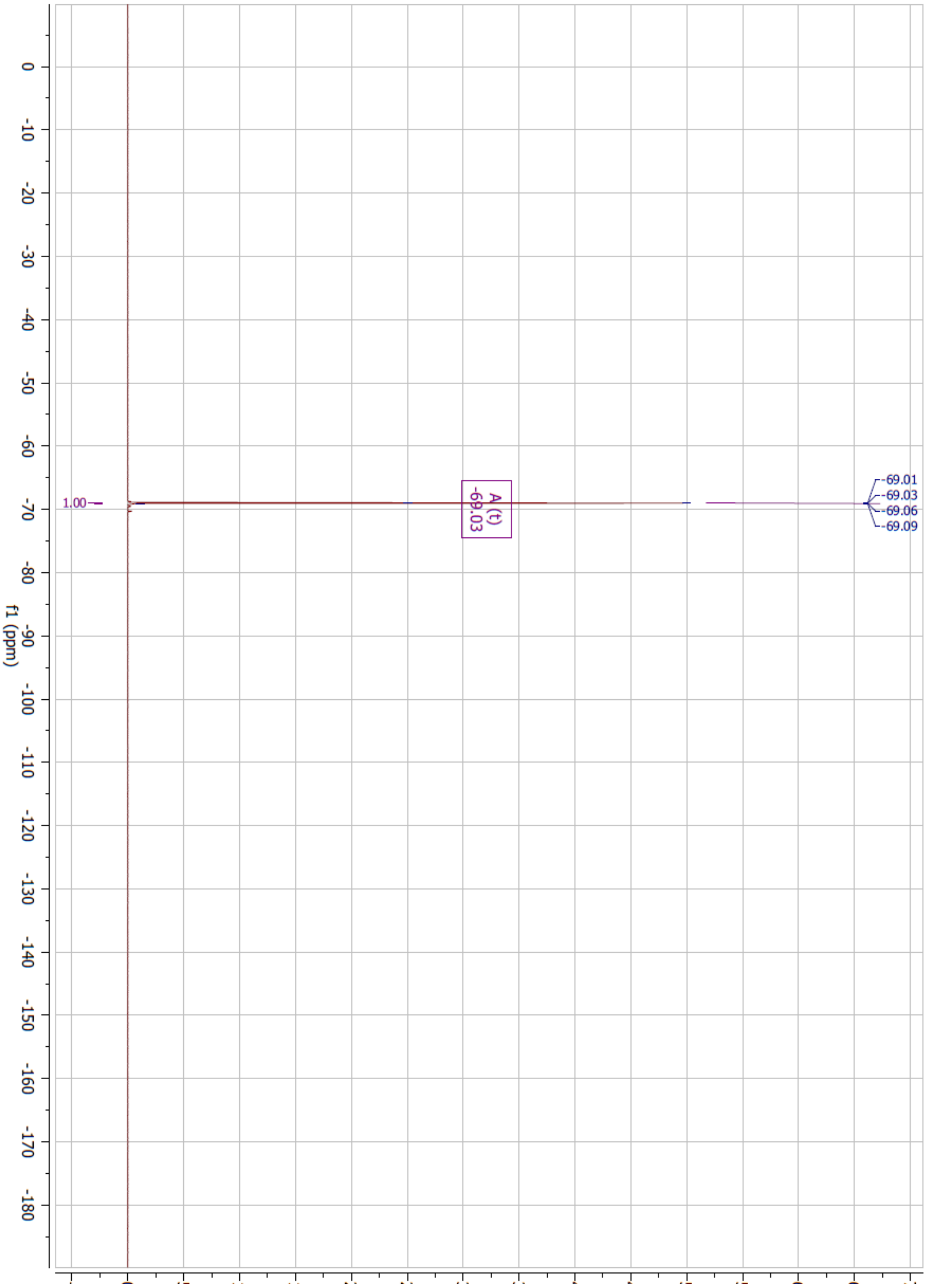

Supplementary Figure 50.

^{1}H-NMR (400 MHz, $CDCl_3$) *N*-(3,5-dimethoxybenzyl)-3-phenyl-*N*-(2,2,2-trifluoroethyl)propan-1-amine (**14**)

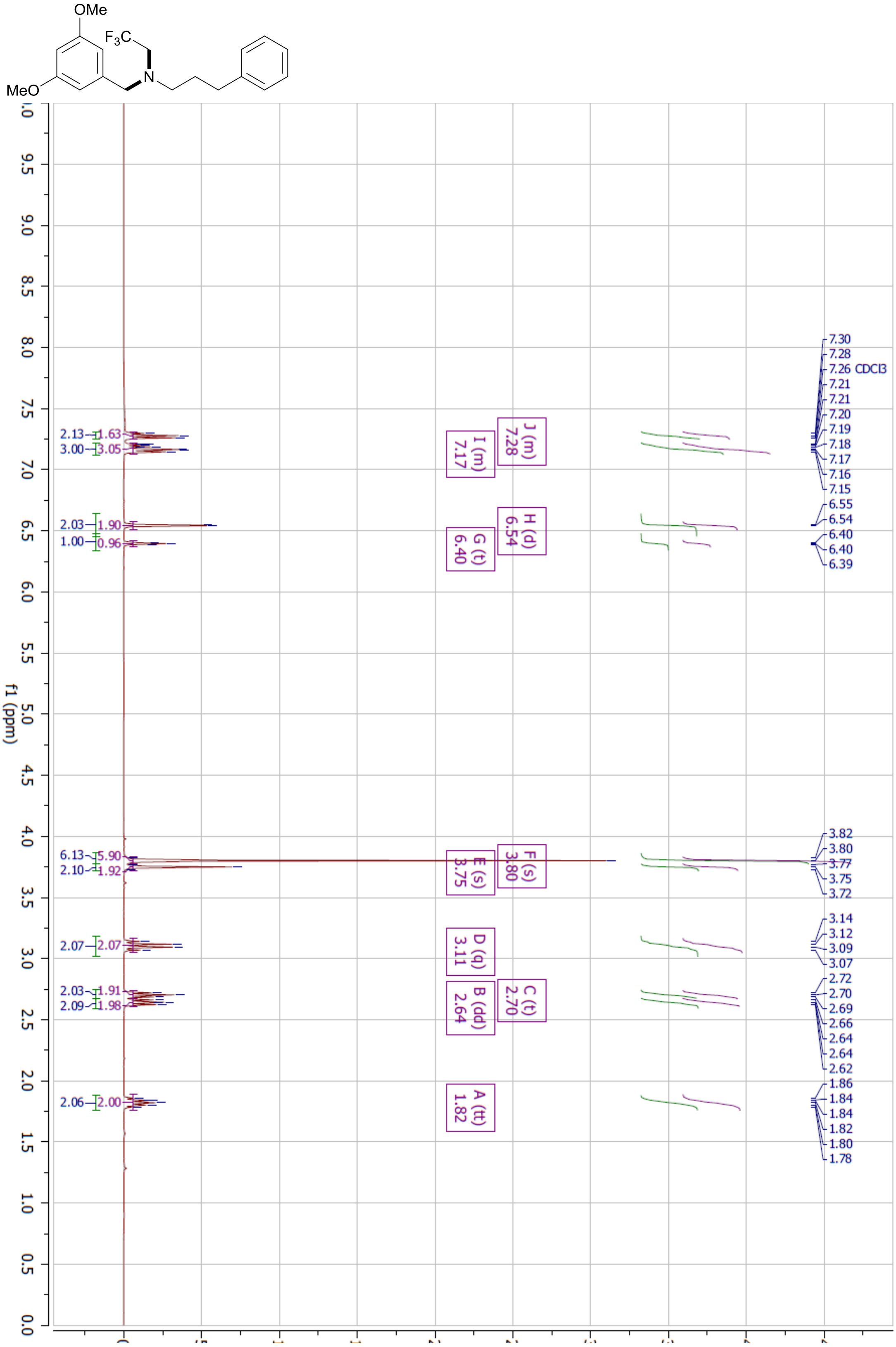

Supplementary Figure 51.

^{13}C-NMR (101 MHz, $CDCl_3$) *N*-(3,5-dimethoxybenzyl)-3-phenyl-*N*-(2,2,2-trifluoroethyl)propan-1-amine (**14**)

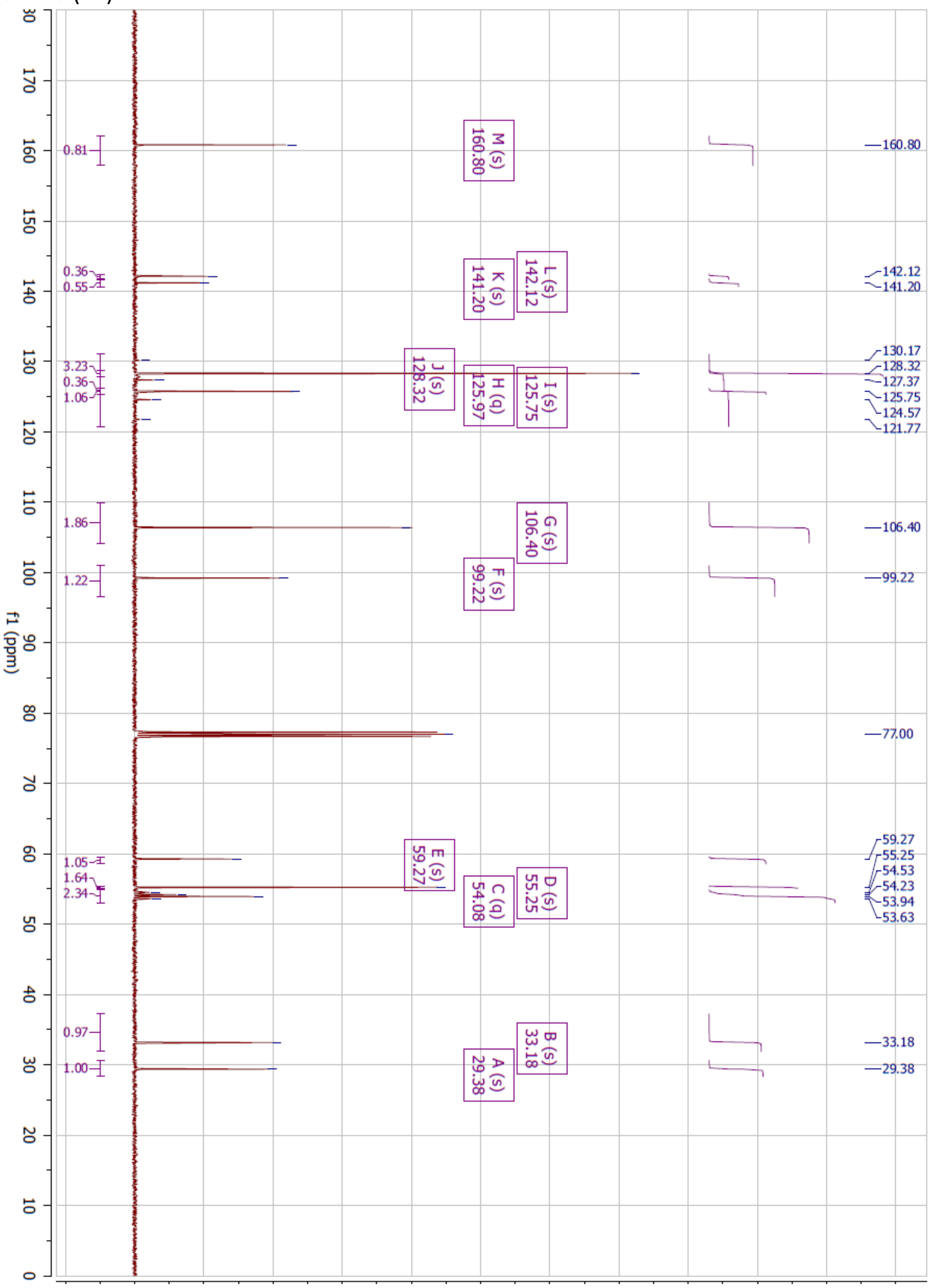

Supplementary Figure 52.
^{19}F-NMR (376 MHz, $CDCl_3$) *N*-(3,5-dimethoxybenzyl)-3-phenyl-*N*-(2,2,2-trifluoroethyl)propan-1-amine (**14**)

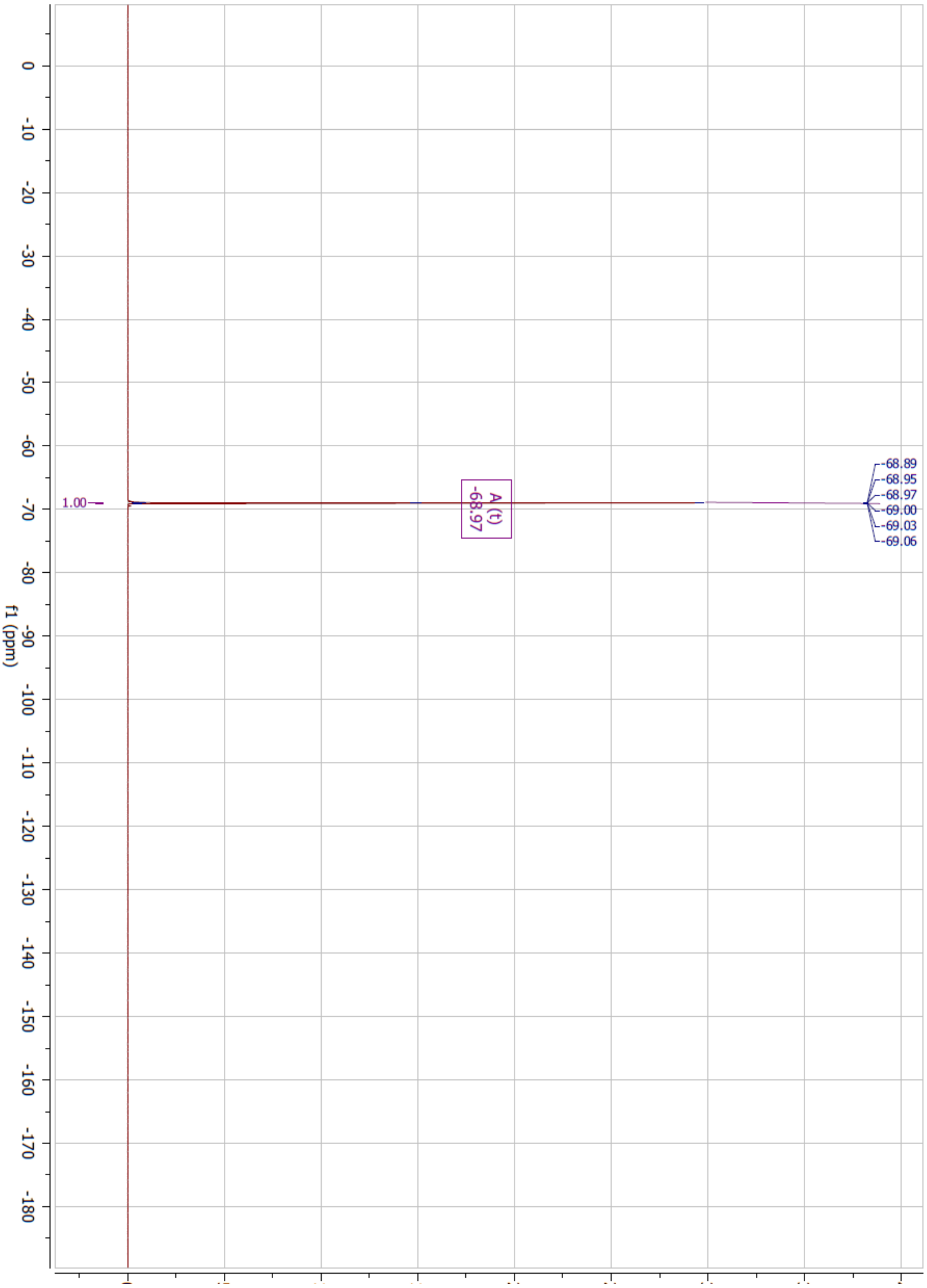

Supplementary Figure 53.

^{1}H-NMR (400 MHz, $CDCl_3$) *N*-(2-(allyloxy)benzyl)-3-phenyl-*N*-(2,2,2-trifluoroethyl)propan-1-amine (**15**)

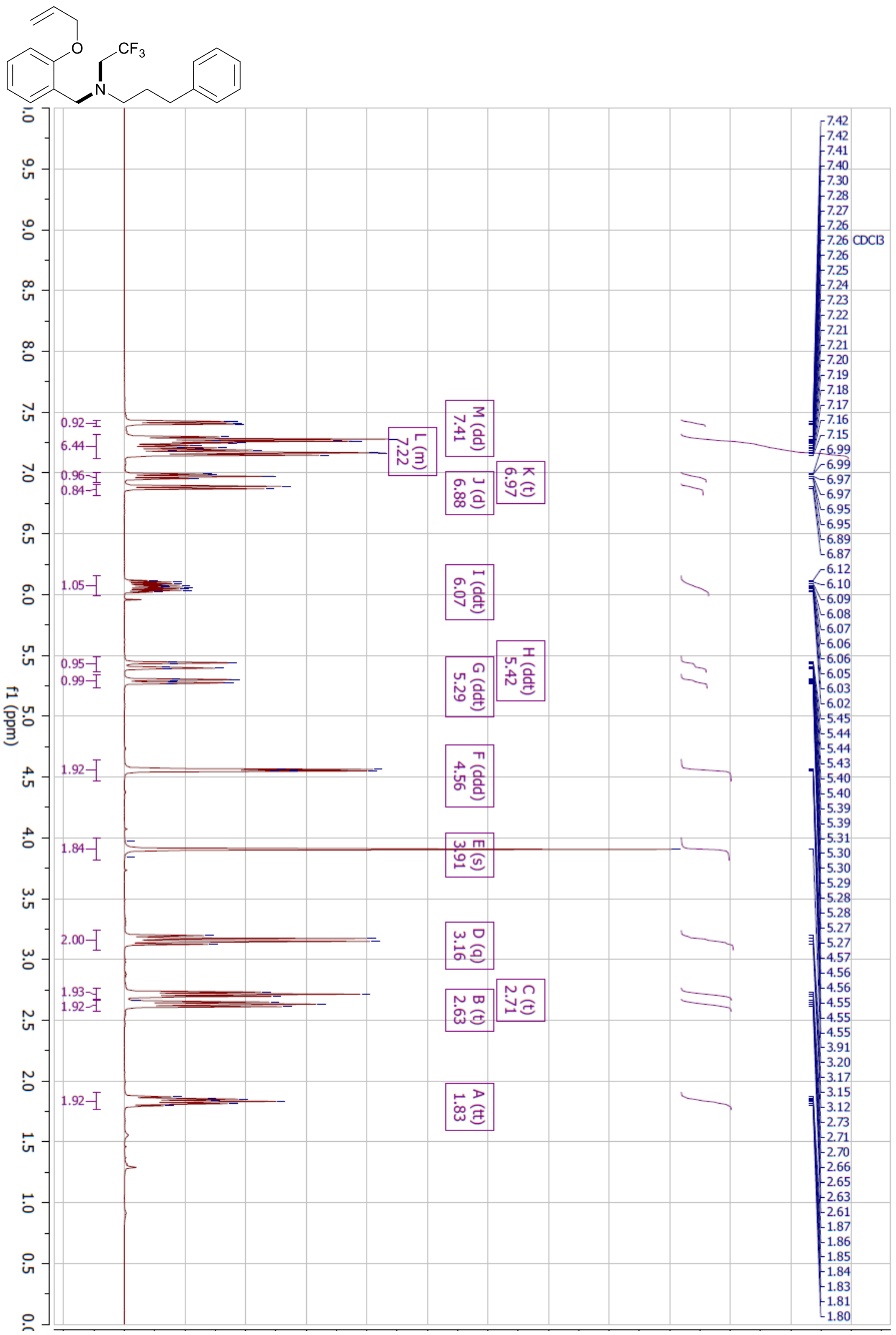

Supplementary Figure 54.

^{13}C-NMR (101 MHz, $CDCl_3$) *N*-(2-(allyloxy)benzyl)-3-phenyl-*N*-(2,2,2-trifluoroethyl)propan-1-amine (**15**)

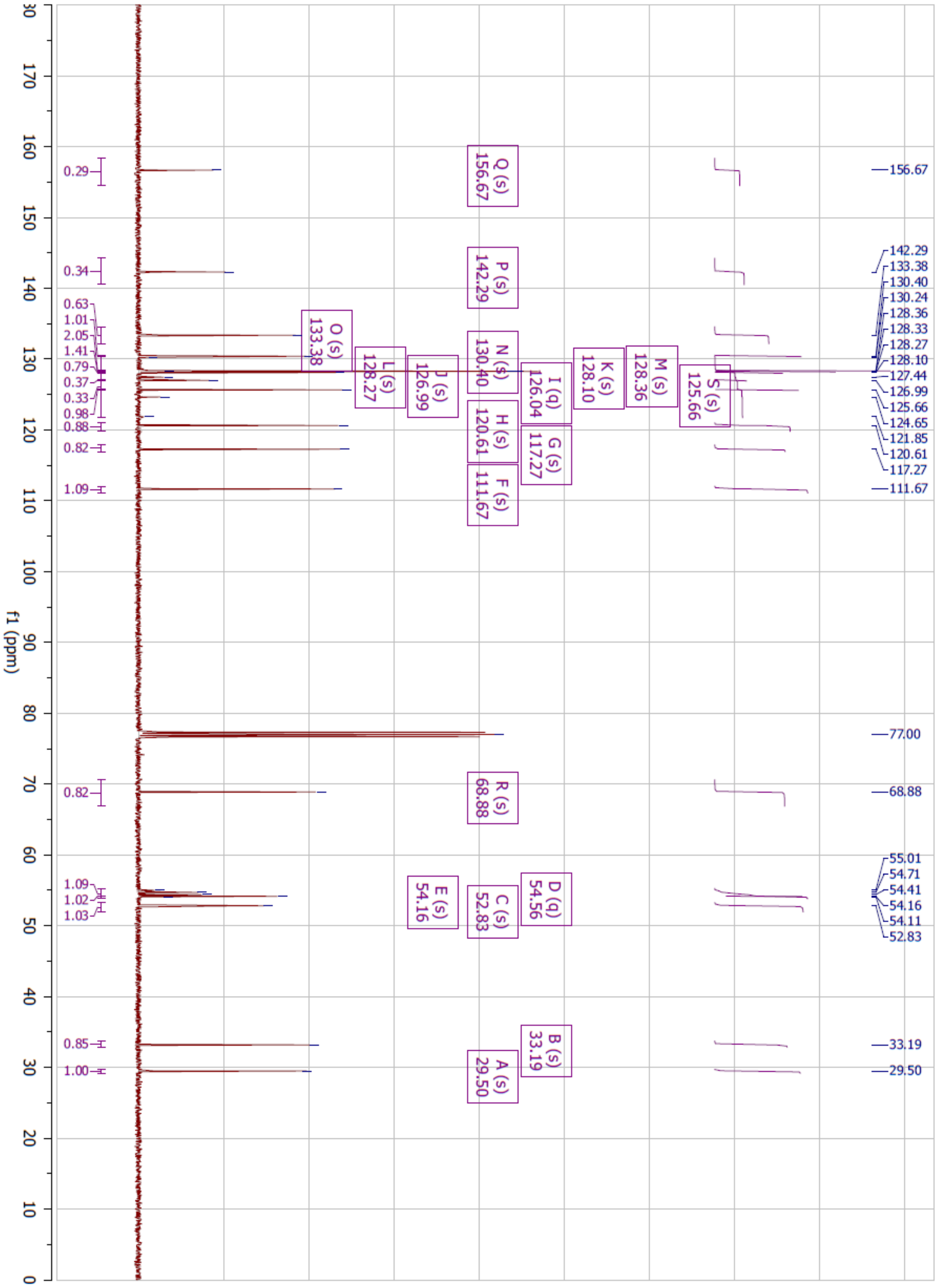

Supplementary Figure 55.

^{19}F-NMR (376 MHz, $CDCl_3$) *N*-(2-(allyloxy)benzyl)-3-phenyl-*N*-(2,2,2-trifluoroethyl)propan-1-amine (**15**)

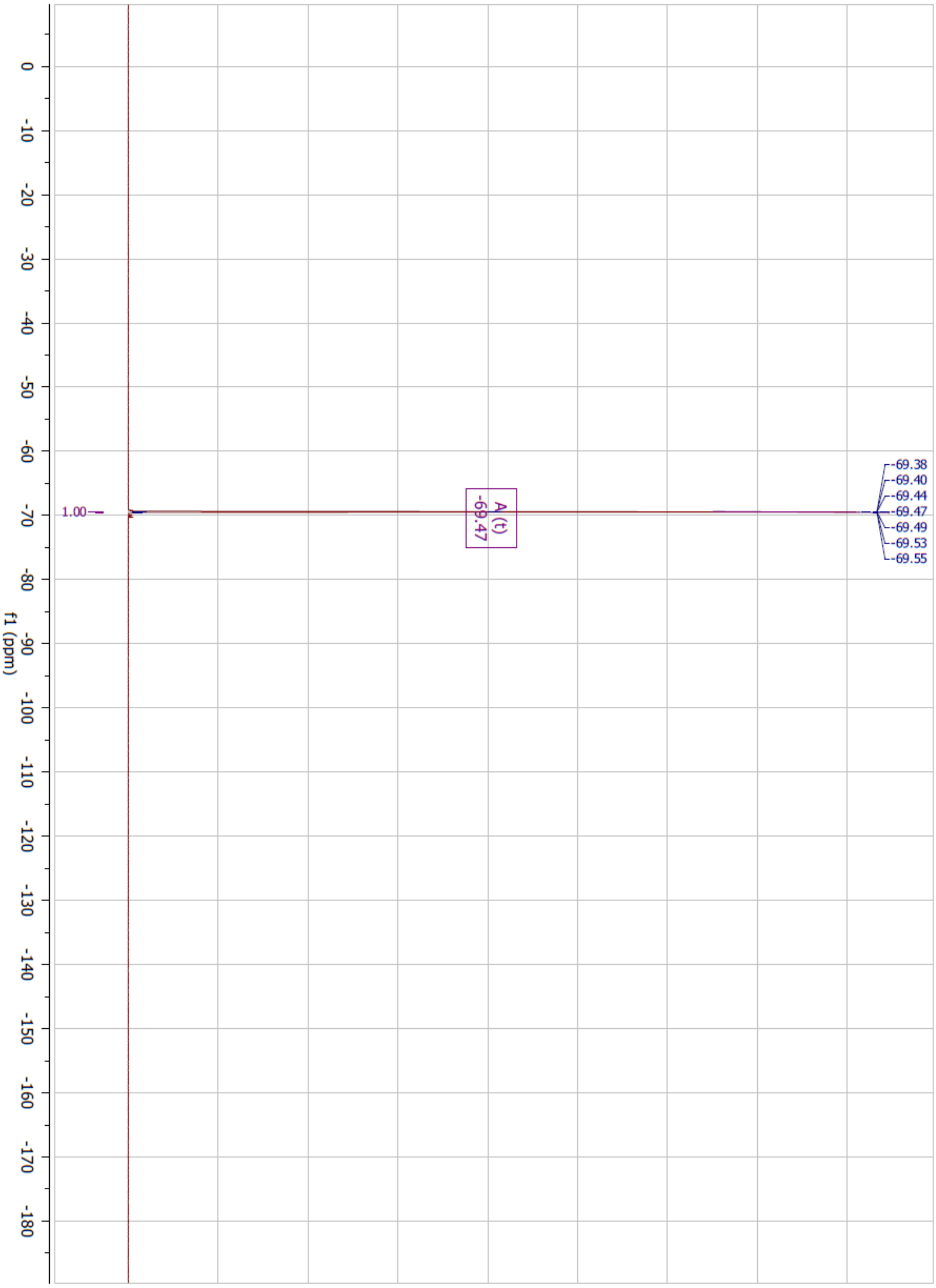

Supplementary Figure 56.

^{1}H-NMR (400 MHz, $CDCl_3$) 4-(((3-phenylpropyl)(2,2,2-trifluoroethyl)amino)methyl)phenyl acetate (**16**)

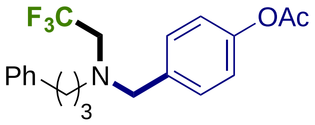

16: 47%

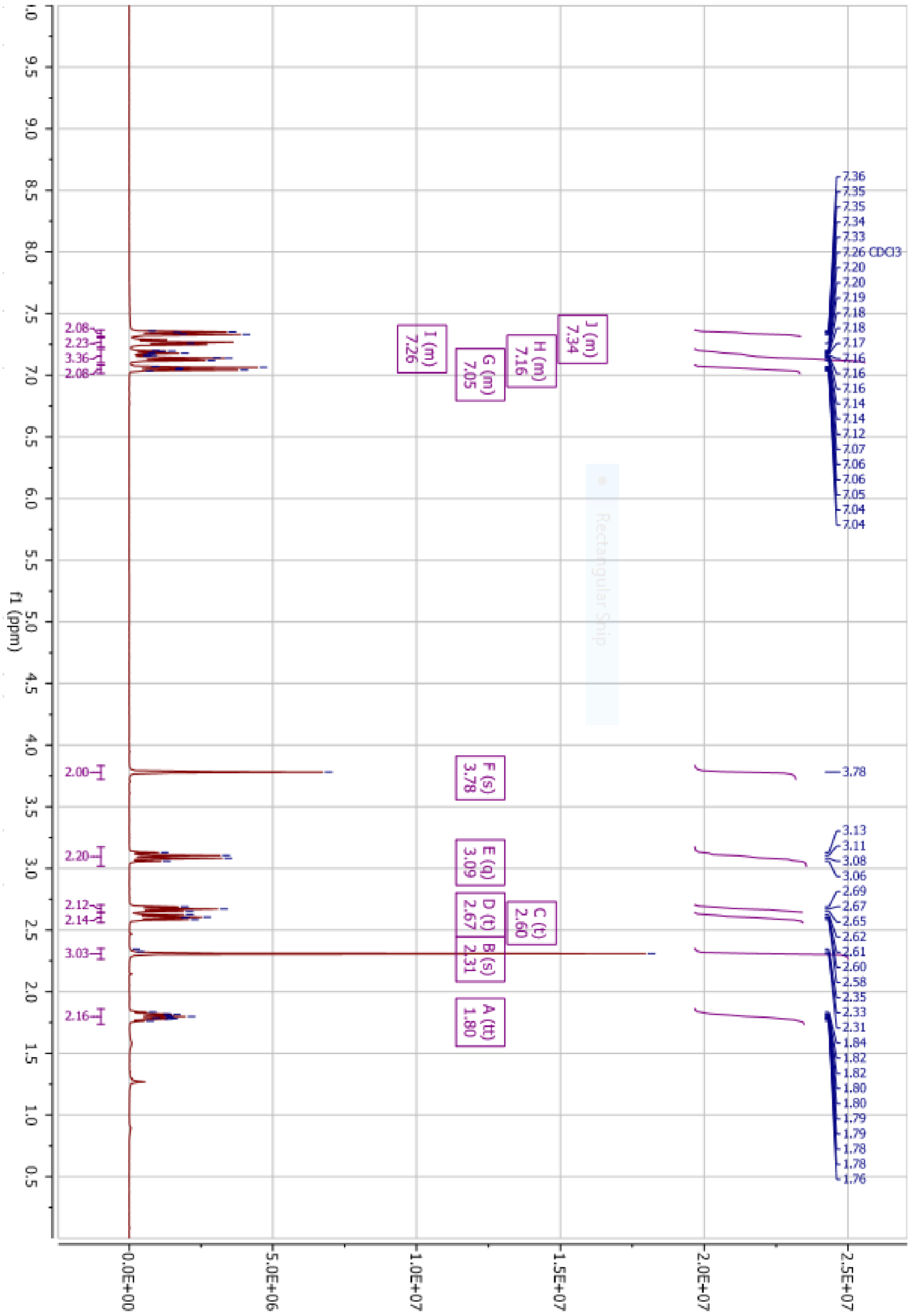

Supplementary Figure 57.

^{13}C-NMR (101 MHz, $CDCl_3$) 4-(((3-phenylpropyl)(2,2,2-trifluoroethyl)amino)methyl)phenyl acetate (**16**)

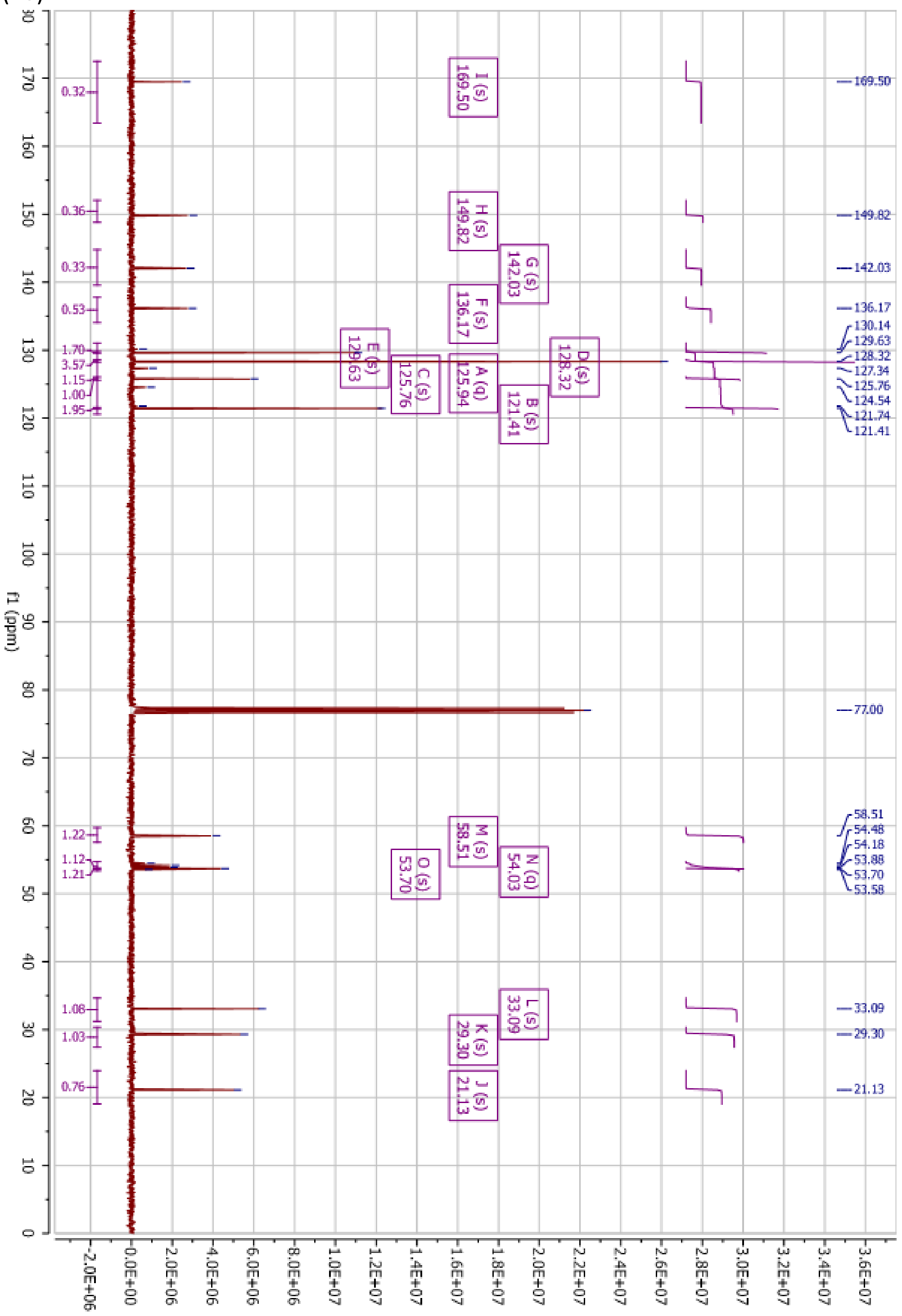

Supplementary Figure 58.

^{19}F-NMR (376 MHz, $CDCl_3$) 4-(((3-phenylpropyl)(2,2,2-trifluoroethyl)amino)methyl)phenyl acetate (**16**)

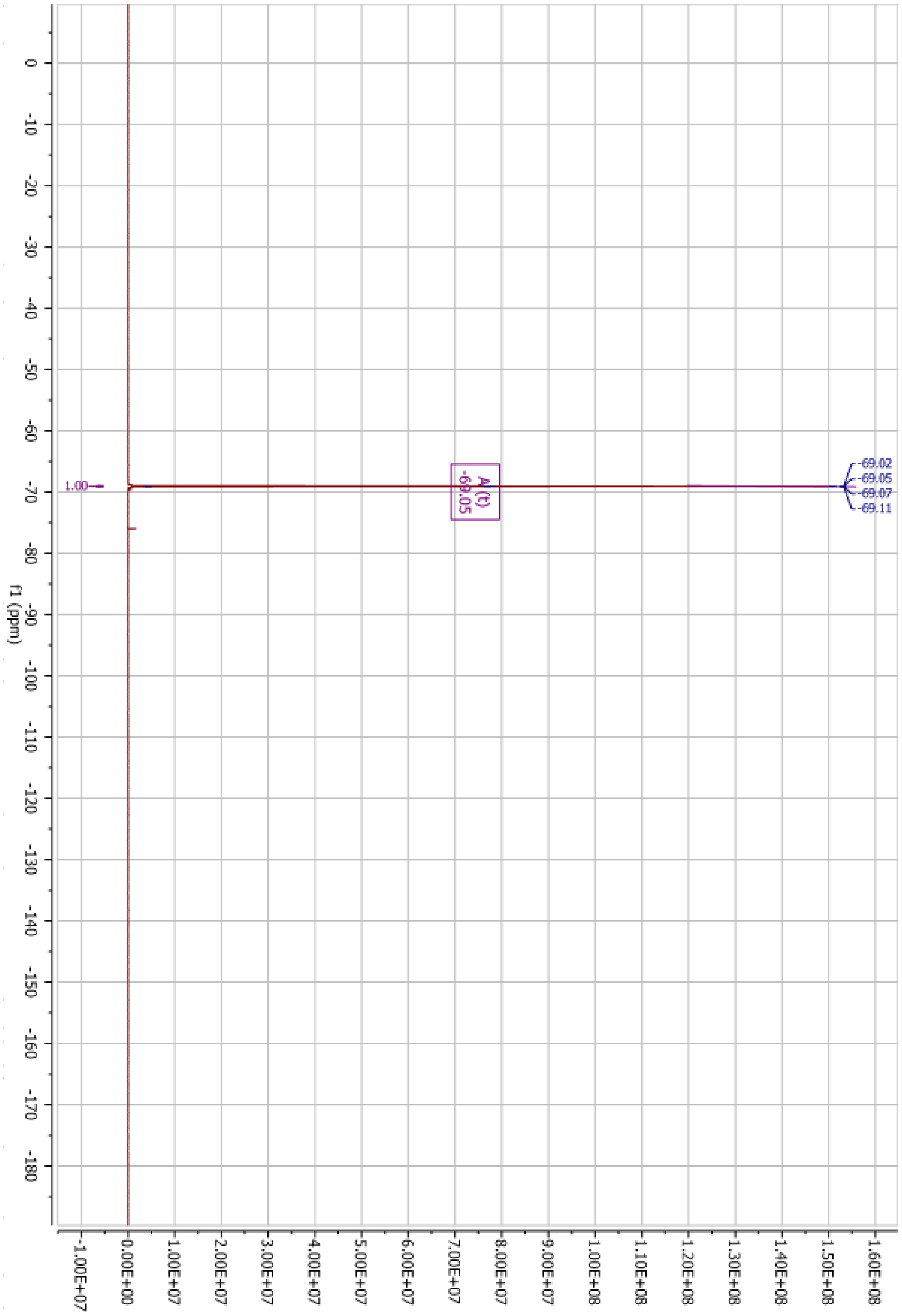

Supplementary Figure 59.

^{1}H-NMR (400 MHz, $CDCl_3$)

N,*N*-dimethyl-4-(((3-phenylpropyl)(2,2,2-trifluoroethyl)amino)methyl)aniline (**17**)

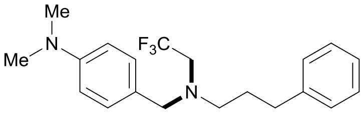

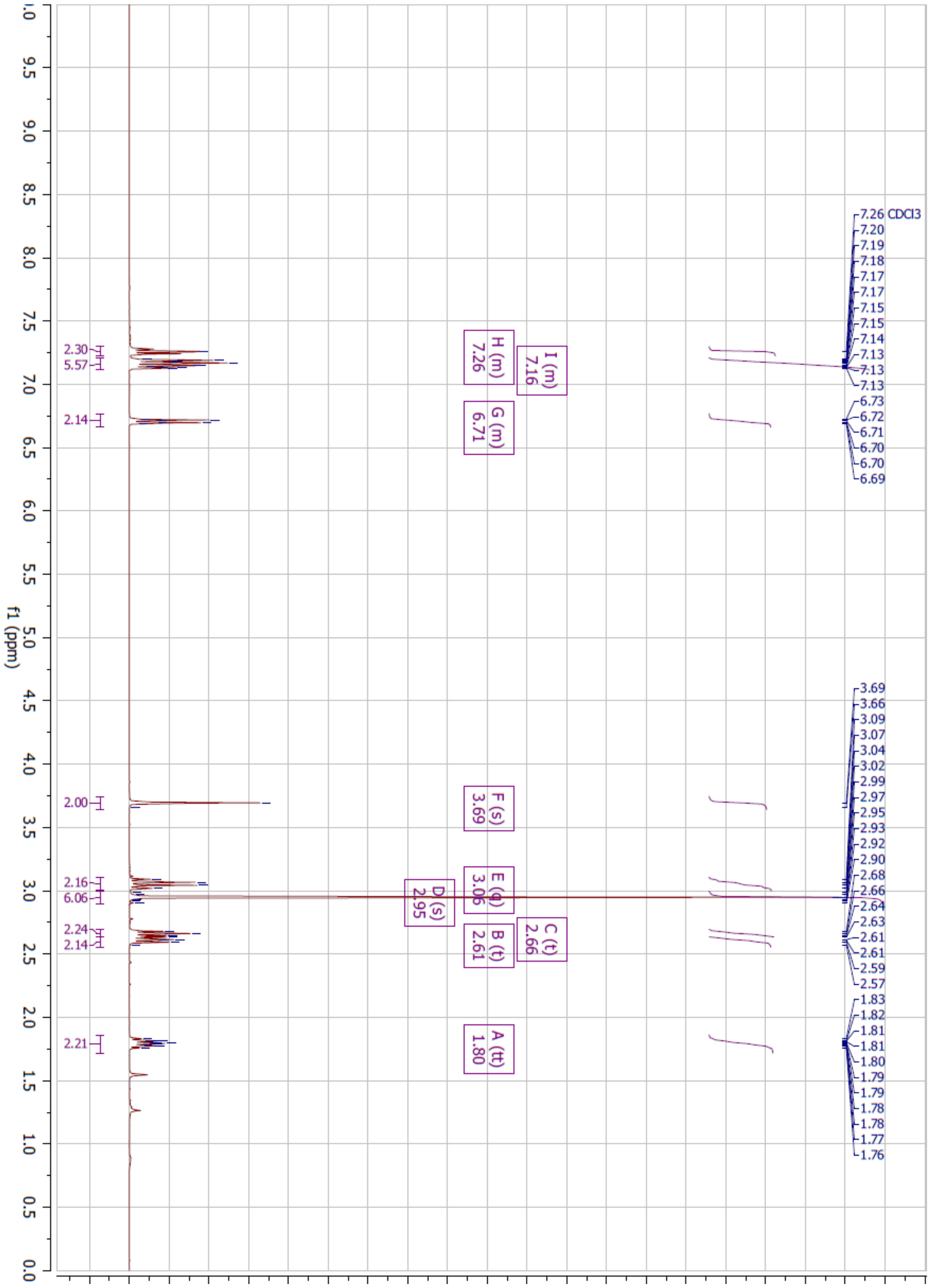

Supplementary Figure 60.

^{13}C-NMR (101 MHz, $CDCl_3$)

N,*N*-dimethyl-4-(((3-phenylpropyl)(2,2,2-trifluoroethyl)amino)methyl)aniline (**17**)

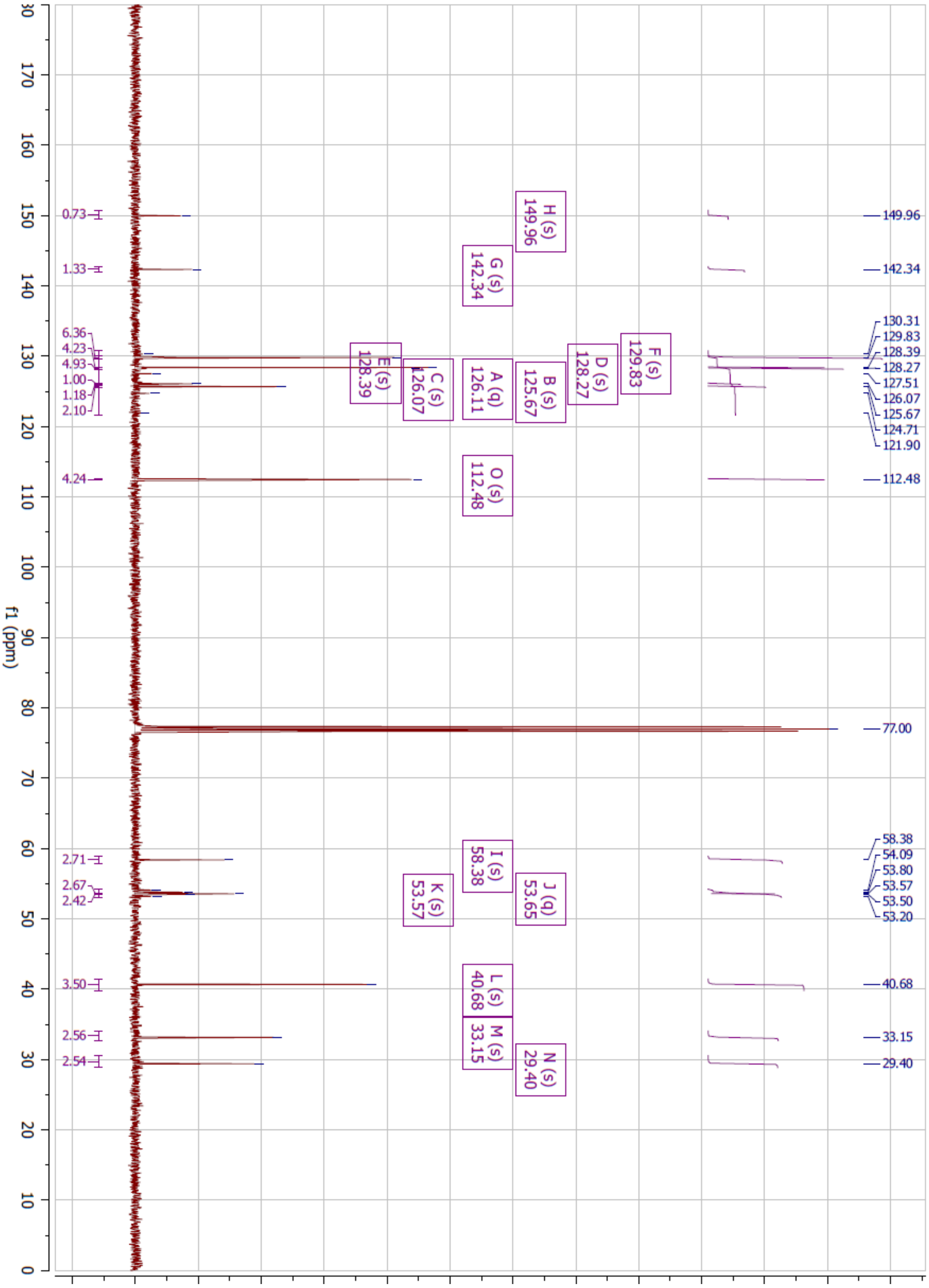

Supplementary Figure 61.

^{19}F-NMR (376 MHz, $CDCl_3$)

N,*N*-dimethyl-4-(((3-phenylpropyl)(2,2,2-trifluoroethyl)amino)methyl)aniline (**17**)

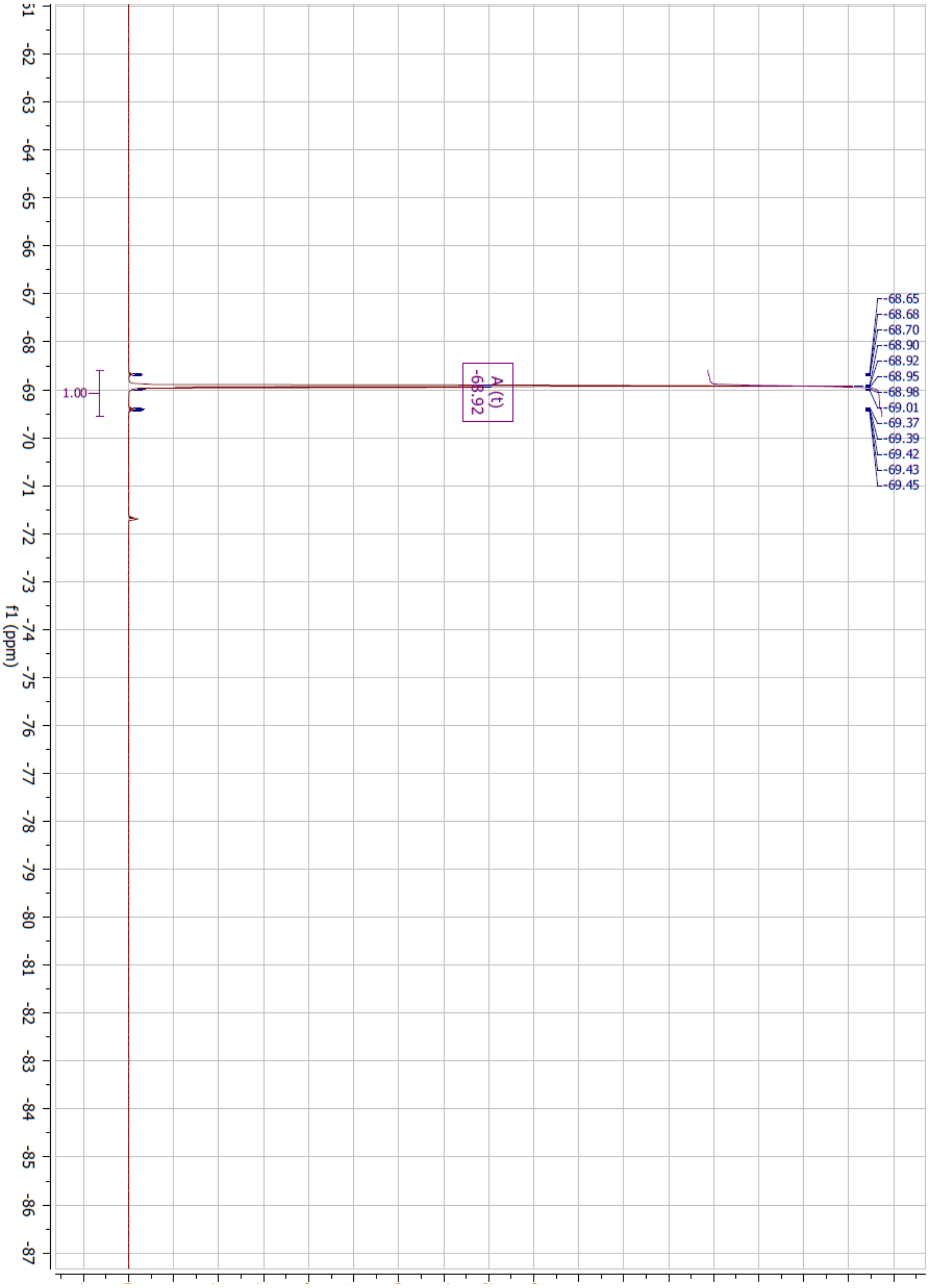

Supplementary Figure 62.

^{1}H-NMR (400 MHz, $CDCl_3$) *N*-(5-bromo-2-methoxybenzyl)-3-phenyl-*N*-(2,2,2-trifluoroethyl)propan-1-amine (**18**)

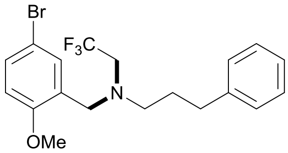

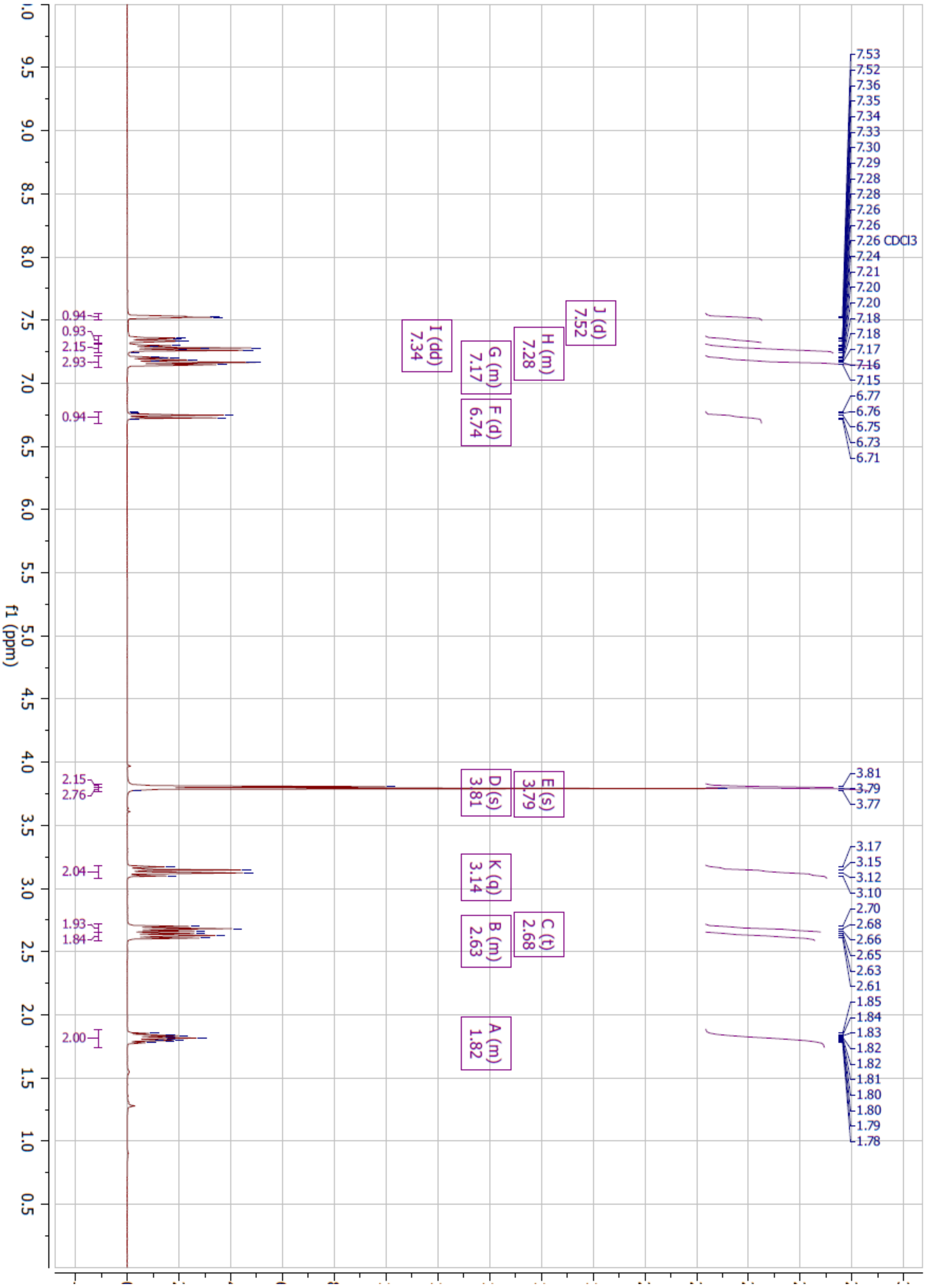

Supplementary Figure 63.

^{13}C-NMR (101 MHz, CDCl$_3$) *N*-(5-bromo-2-methoxybenzyl)-3-phenyl-*N*-(2,2,2-trifluoroethyl)propan-1-amine (**18**)

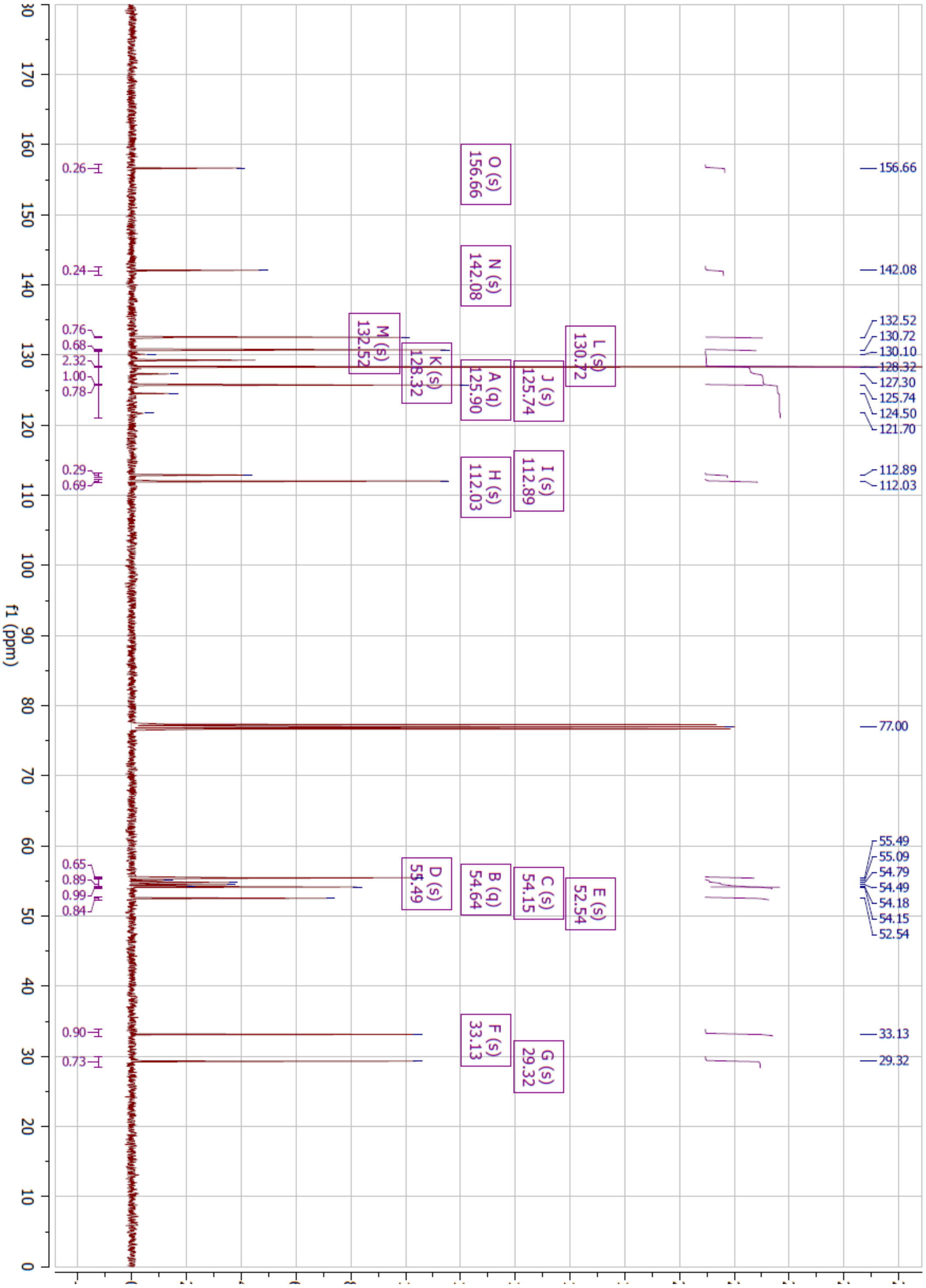

Supplementary Figure 64.
^{19}F-NMR (376 MHz, $CDCl_3$) *N*-(5-bromo-2-methoxybenzyl)-3-phenyl-*N*-(2,2,2-trifluoroethyl)propan-1-amine (**18**)

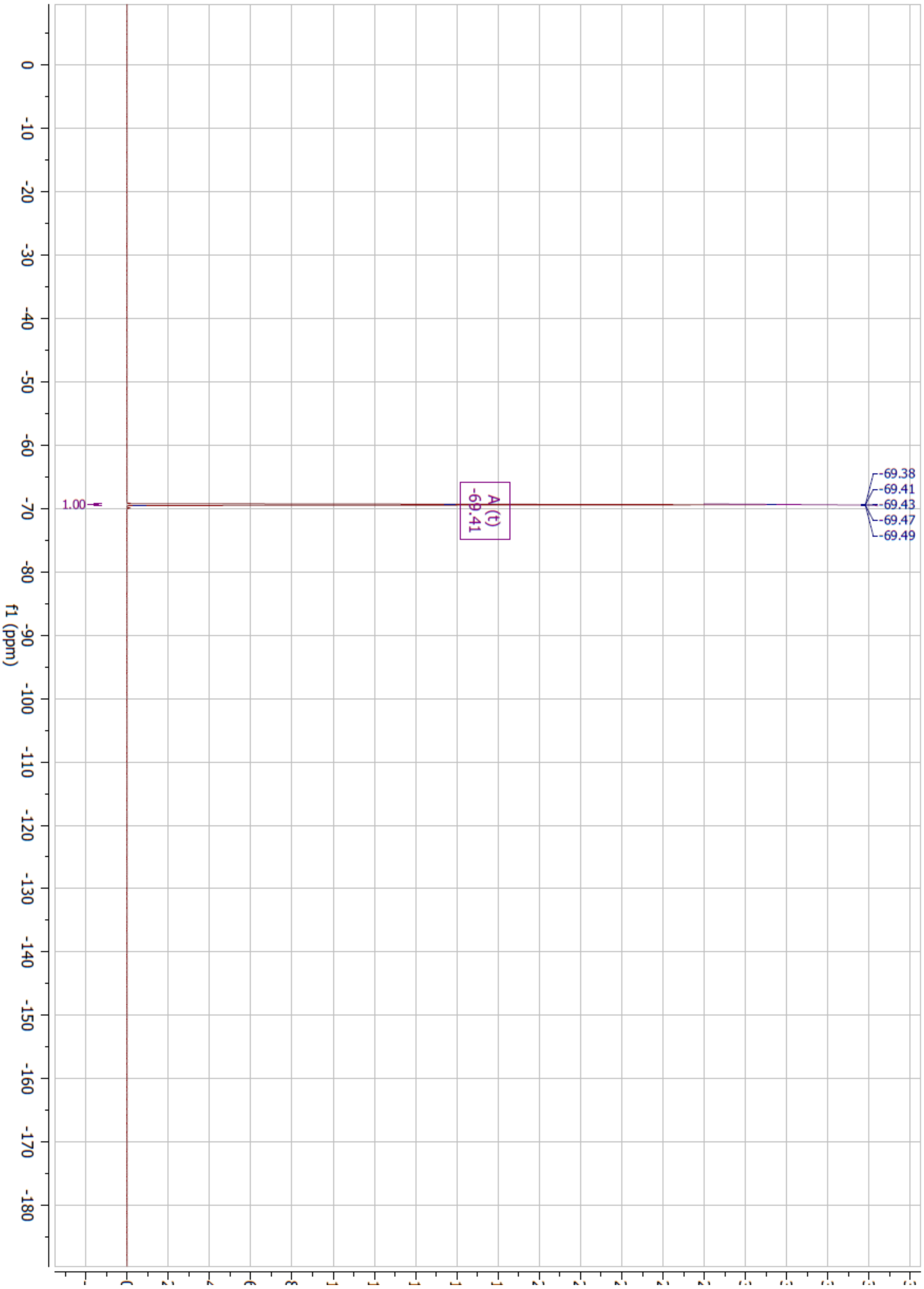

Supplementary Figure 65.

^{1}H-NMR (400 MHz, CDCl$_3$) 3-phenyl-*N*-(pyridin-3-ylmethyl)-*N*-(2,2,2-trifluoroethyl)propan-1-amine (**19**)

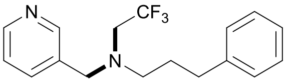

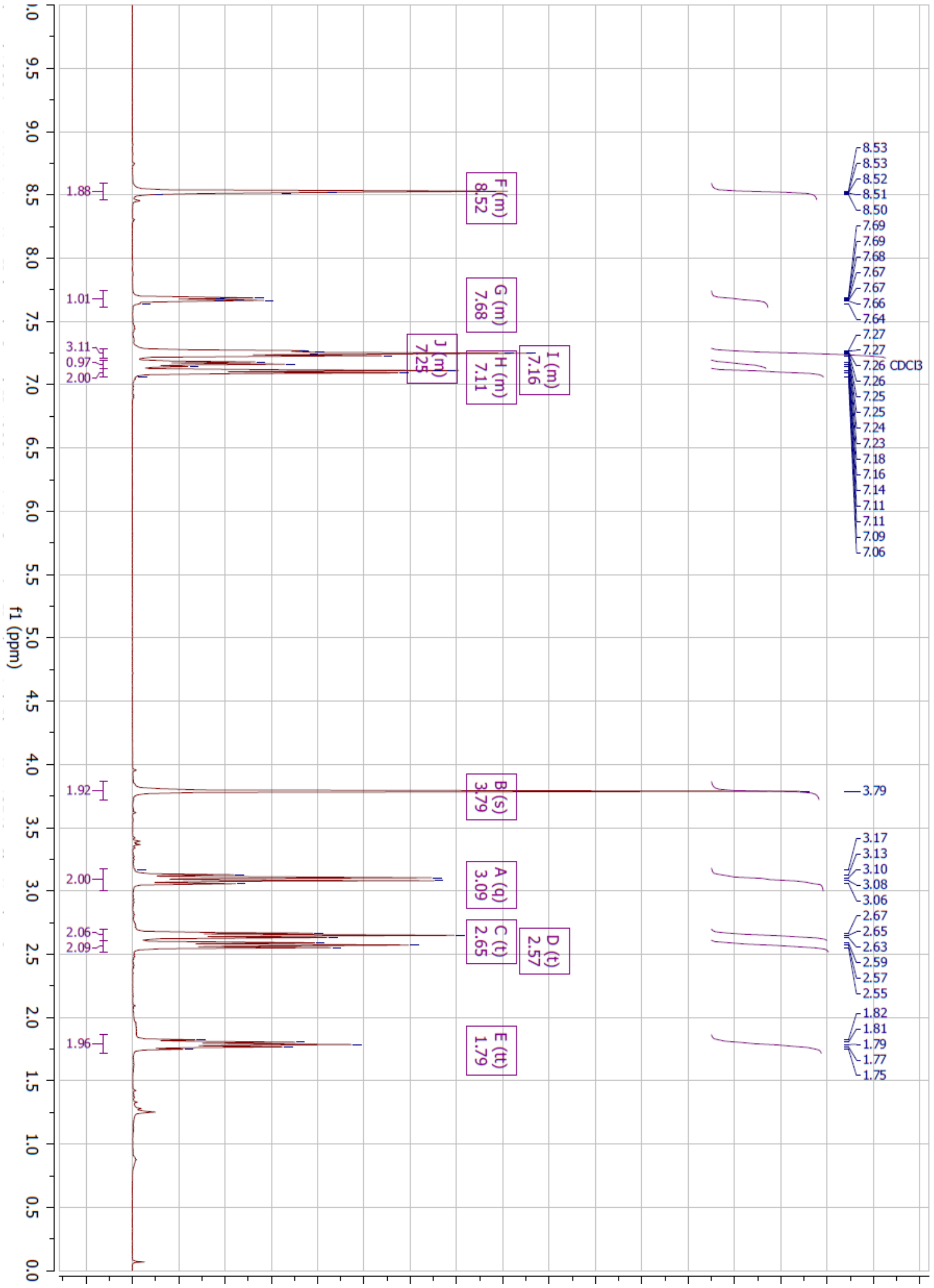

Supplementary Figure 66.

^{13}**C-NMR (101 MHz, CDCl$_3$)** 3-phenyl-*N*-(pyridin-3-ylmethyl)-*N*-(2,2,2-trifluoroethyl)propan-1-amine (**19**)

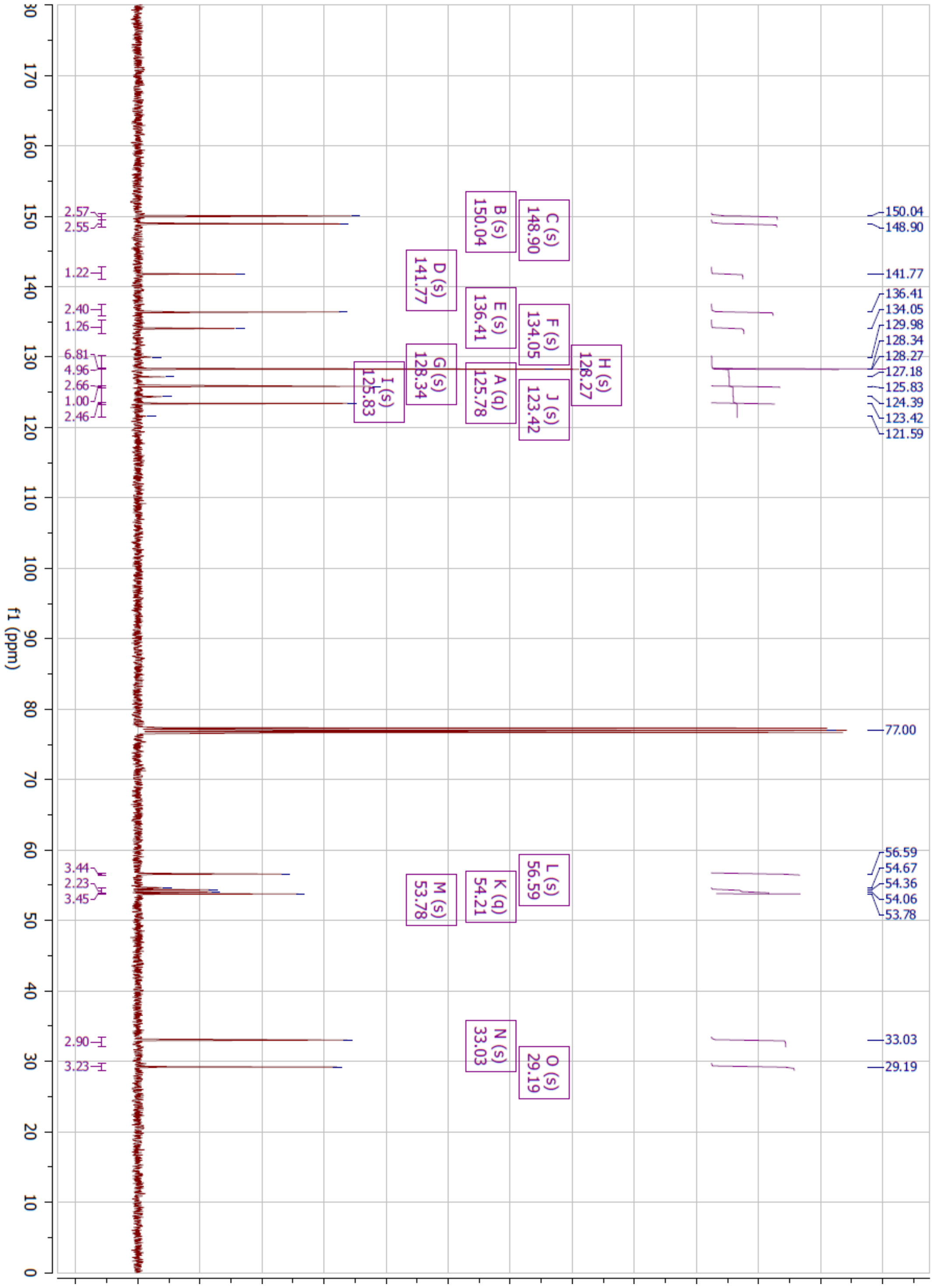

Supplementary Figure 67.

^{19}F-NMR (376 MHz, $CDCl_3$) 3-phenyl-*N*-(pyridin-3-ylmethyl)-*N*-(2,2,2-trifluoroethyl)propan-1-amine (**19**)

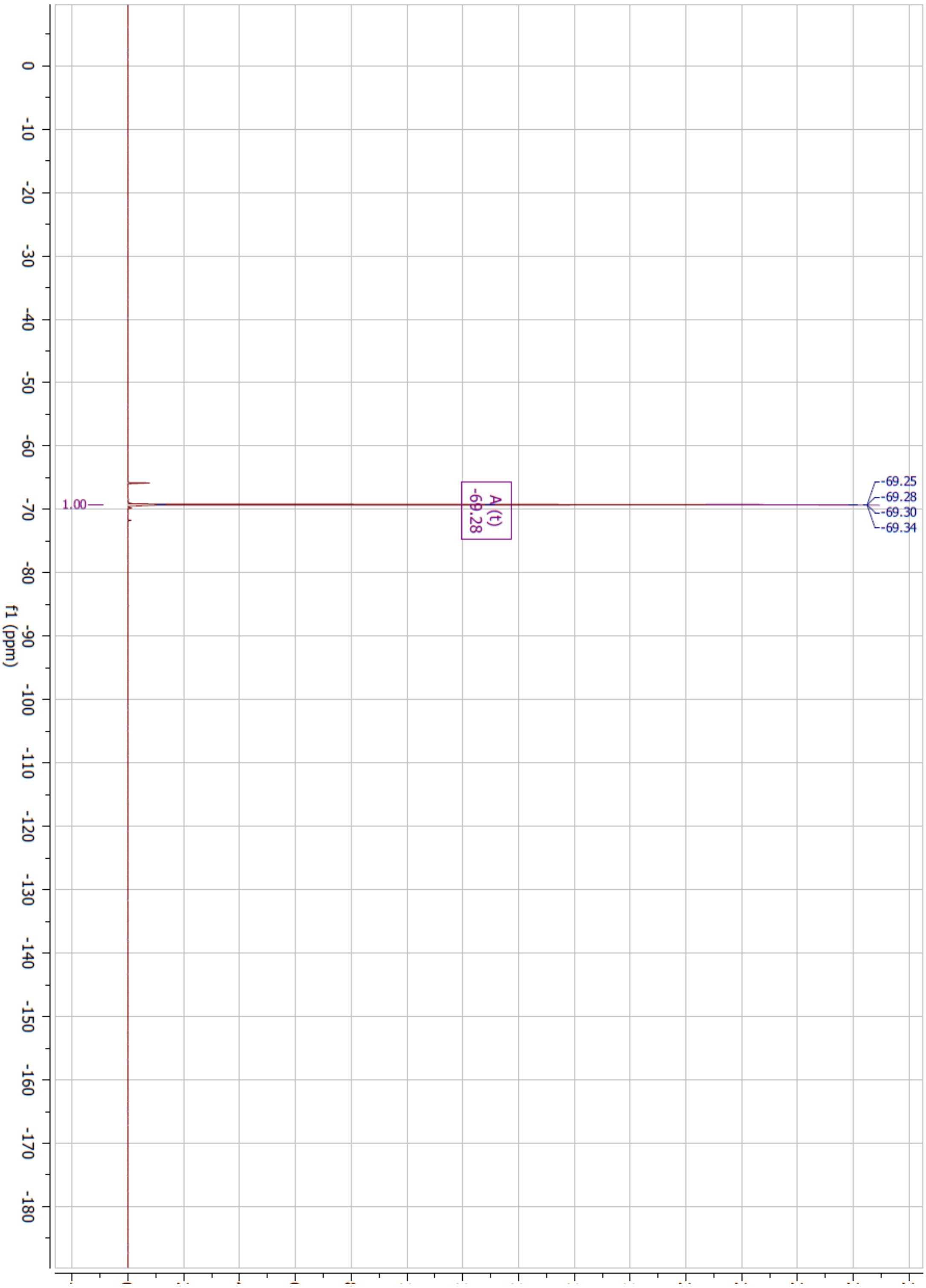

Supplementary Figure 68.

^{1}H-NMR (400 MHz, $CDCl_3$) *N*-(cyclohexylmethyl)-3-phenyl-*N*-(2,2,2-trifluoroethyl)propan-1-amine (**20**)

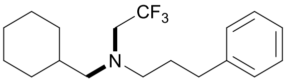

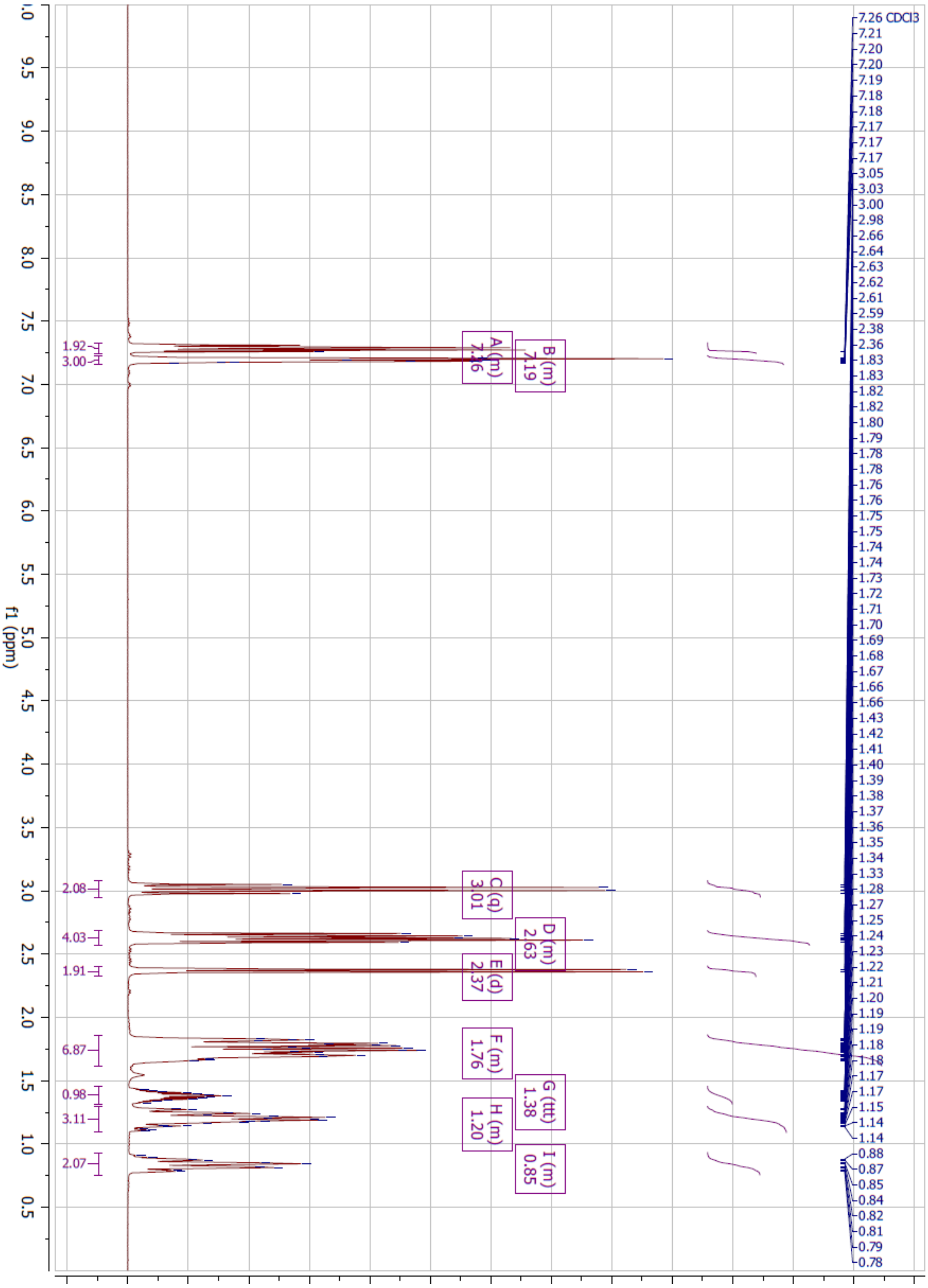

Supplementary Figure 69.

^{13}C-NMR (101 MHz, $CDCl_3$) *N*-(cyclohexylmethyl)-3-phenyl-*N*-(2,2,2-trifluoroethyl)propan-1-amine (**20**)

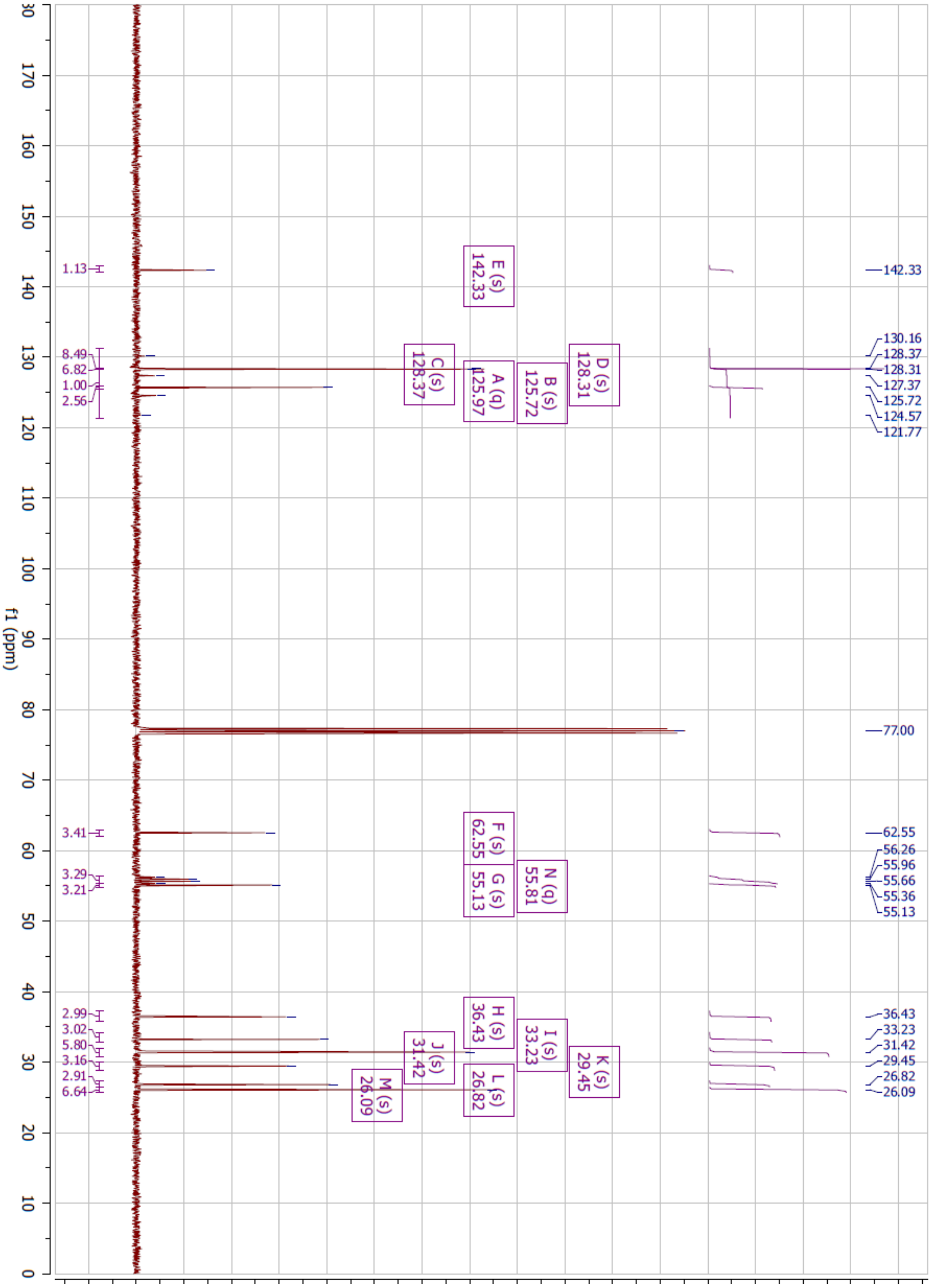

Supplementary Figure 70.

^{19}F-NMR (376 MHz, $CDCl_3$) *N*-(cyclohexylmethyl)-3-phenyl-*N*-(2,2,2-trifluoroethyl)propan-1-amine (**20**)

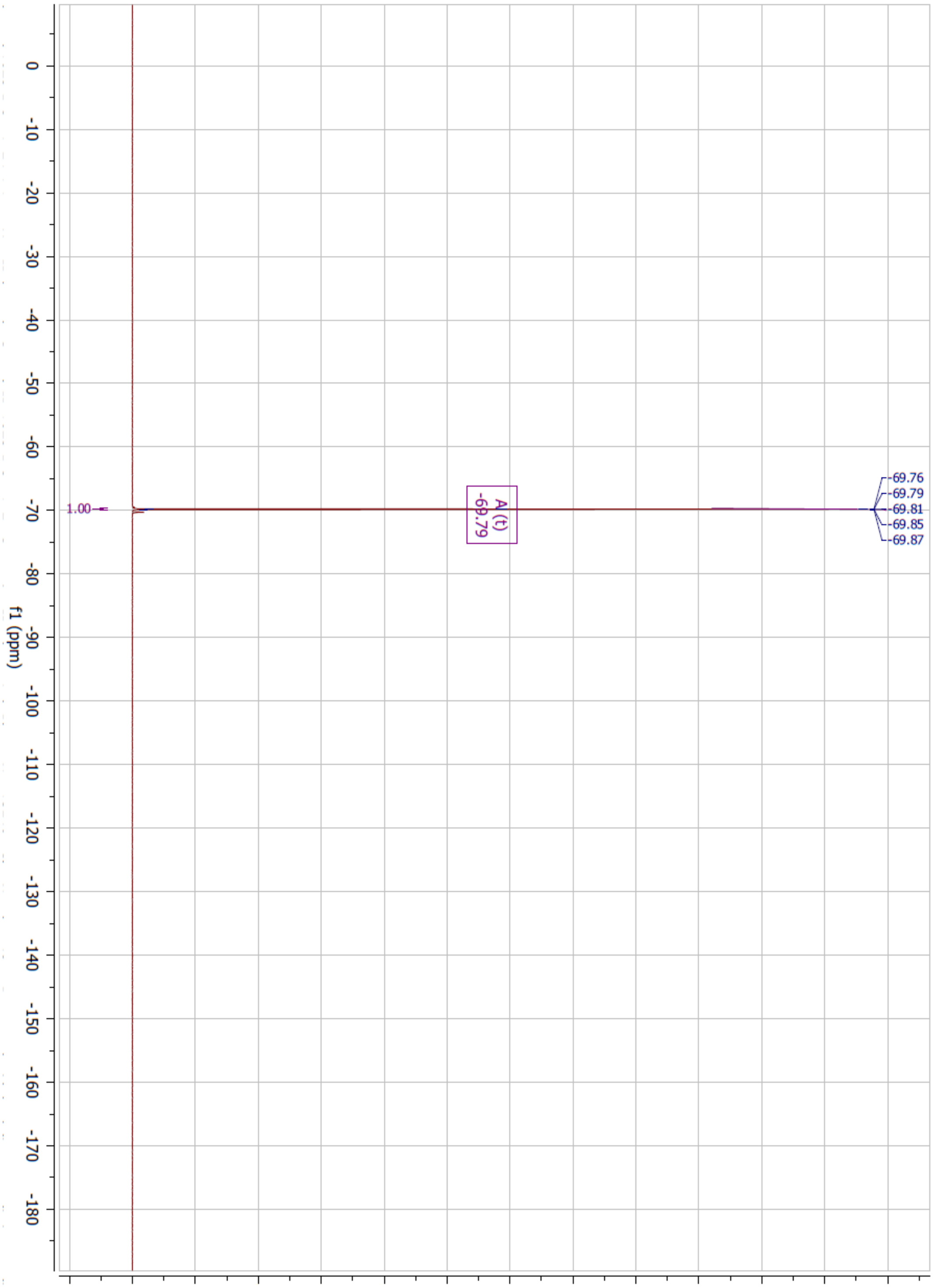

Supplementary Figure 71.

^{1}H-NMR (400 MHz, $CDCl_3$) *N*-(4-methoxybenzyl)-*N*-(2,2,2-trifluoroethyl)prop-2-en-1-amine (**21**)

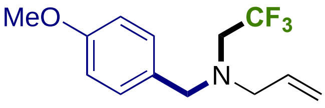

21: 63%

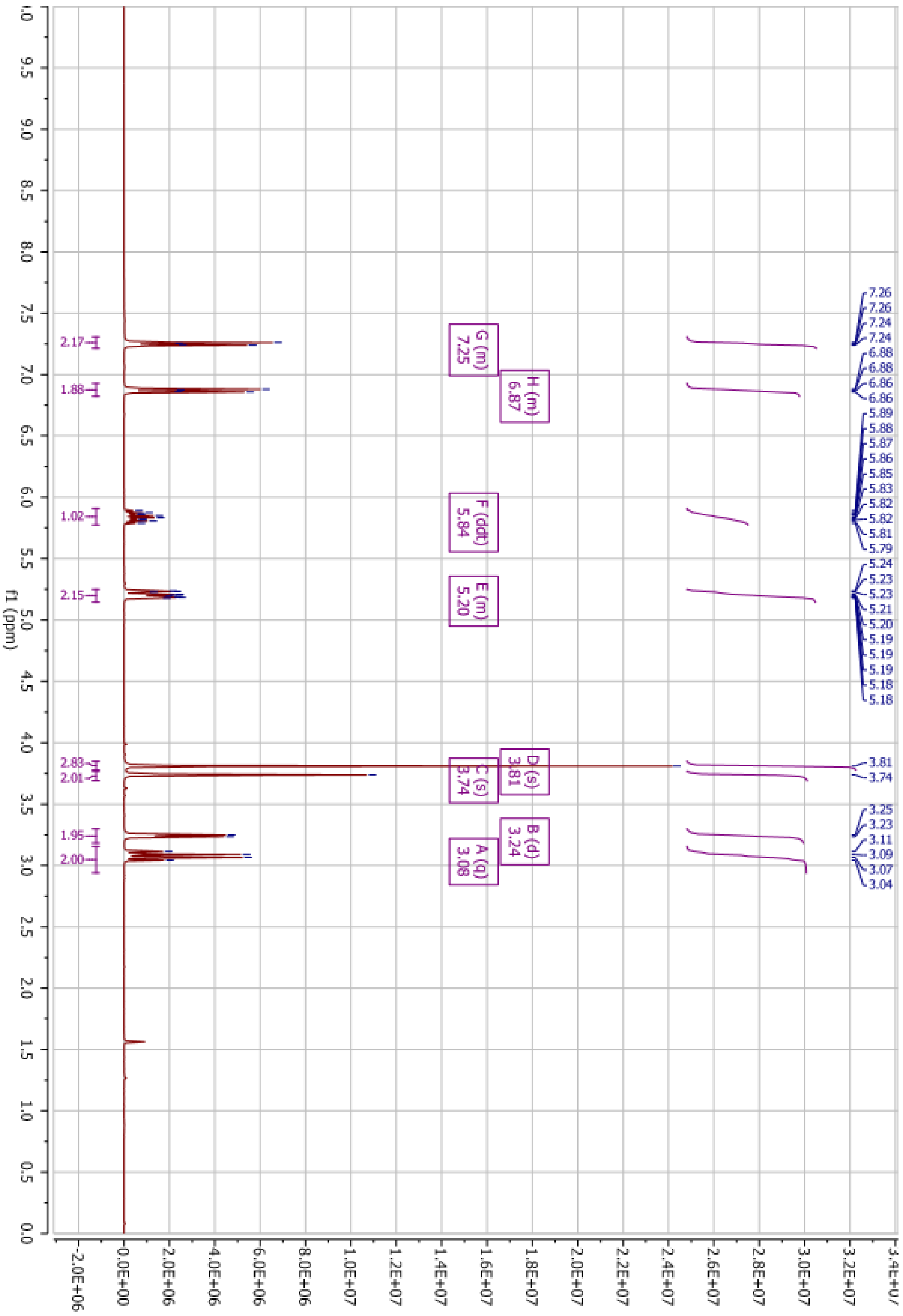

Supplementary Figure 72.

^{13}C-NMR (101 MHz, CDCl$_3$) *N*-(4-methoxybenzyl)-*N*-(2,2,2-trifluoroethyl)prop-2-en-1-amine (**21**)

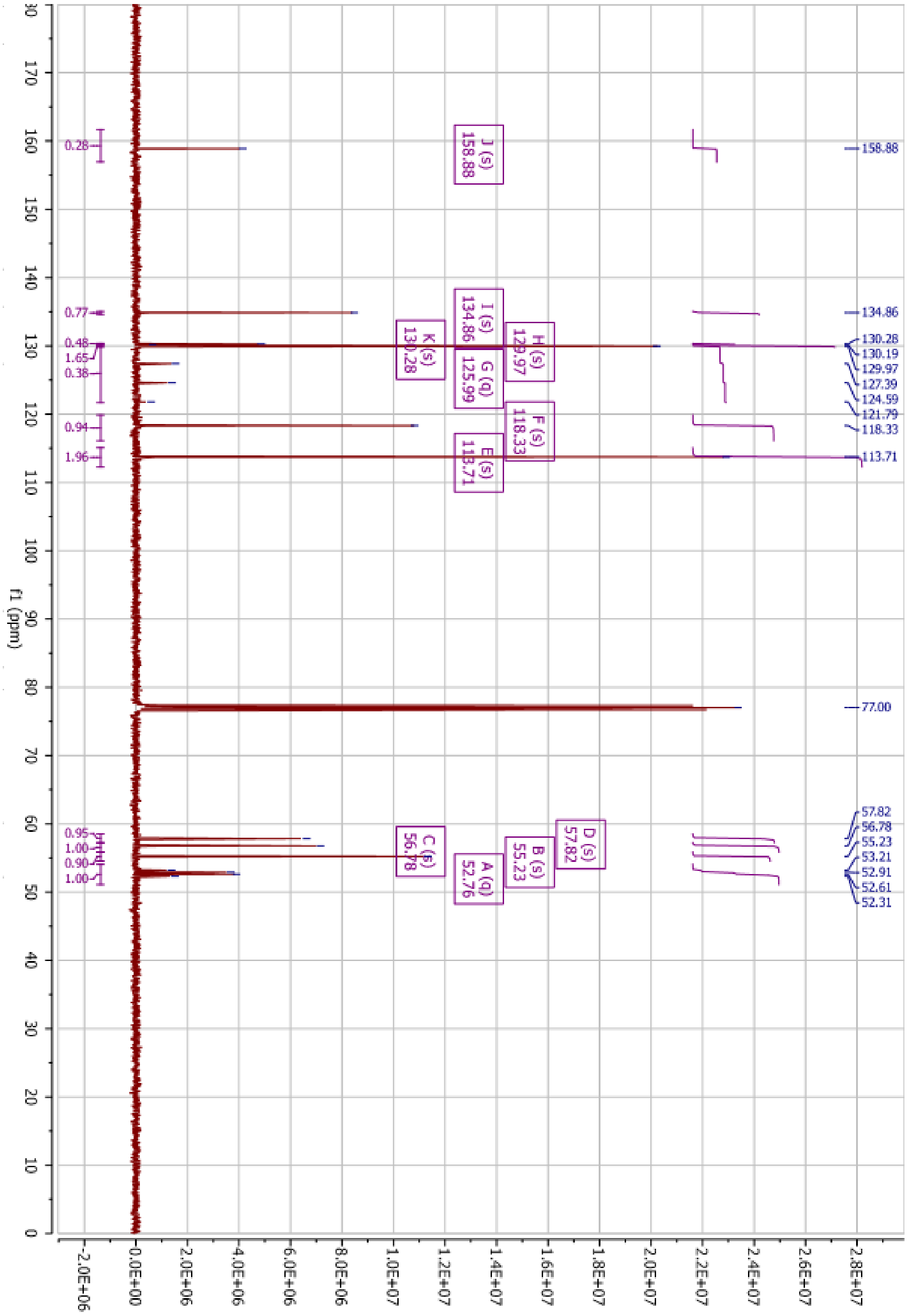

Supplementary Figure 73.

^{19}F-NMR (376 MHz, $CDCl_3$)

N-(4-methoxybenzyl)-*N*-(2,2,2-trifluoroethyl)prop-2-en-1-amine (**21**)

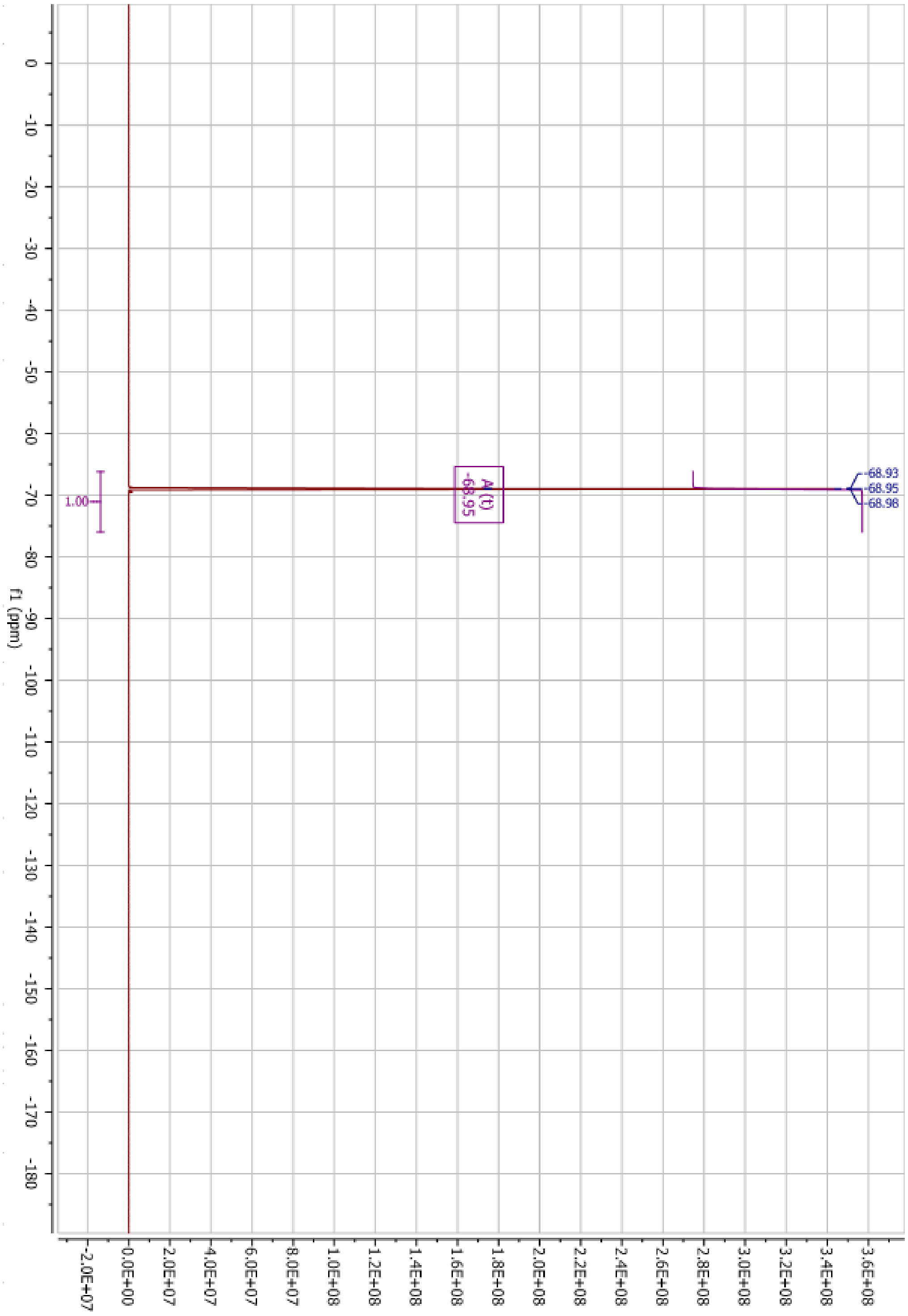

Supplementary Figure 74.

^{1}H-NMR (400 MHz, $CDCl_3$) 3-chloro-*N*-(4-methoxybenzyl)-*N*-(2,2,2-trifluoroethyl)propan-1-amine (**22**)

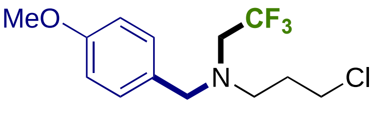

22: 67%

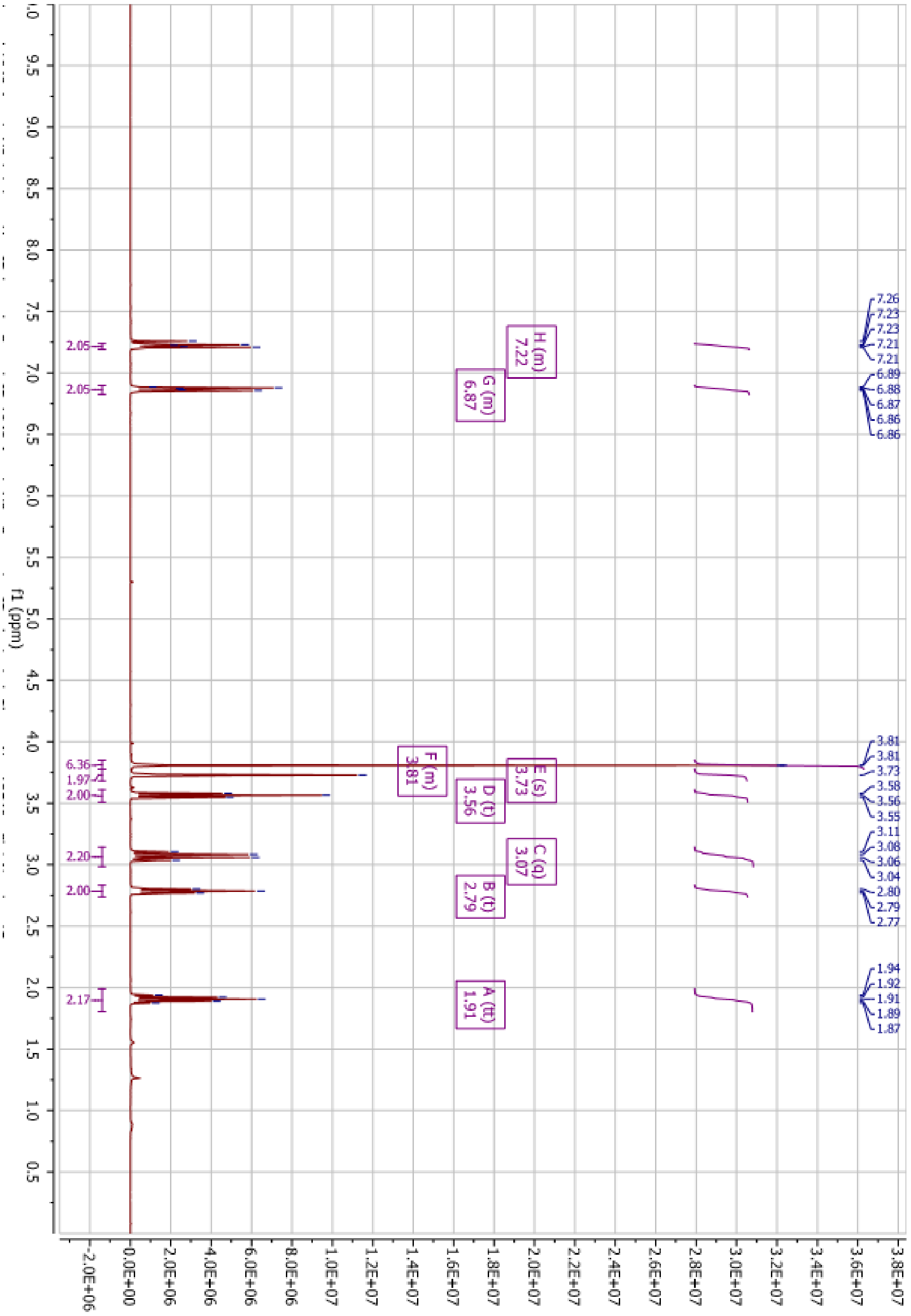

Supplementary Figure 75.

^{13}C-NMR (101 MHz, $CDCl_3$) 3-chloro-*N*-(4-methoxybenzyl)-*N*-(2,2,2-trifluoroethyl)propan-1-amine (**22**)

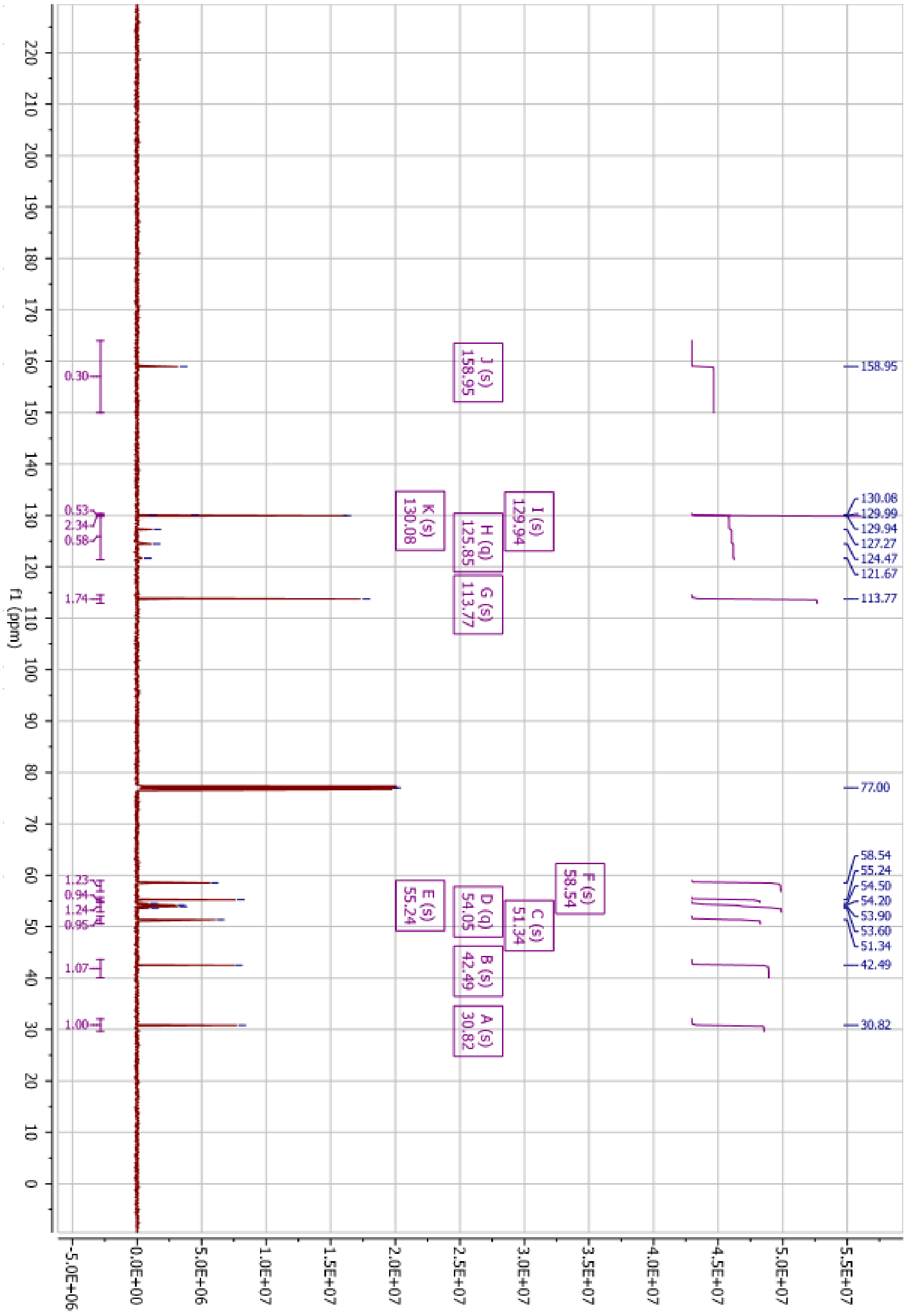

Supplementary Figure 76.

^{19}F-NMR (376 MHz, $CDCl_3$) 3-chloro-*N*-(4-methoxybenzyl)-*N*-(2,2,2-trifluoroethyl)propan-1-amine (**22**)

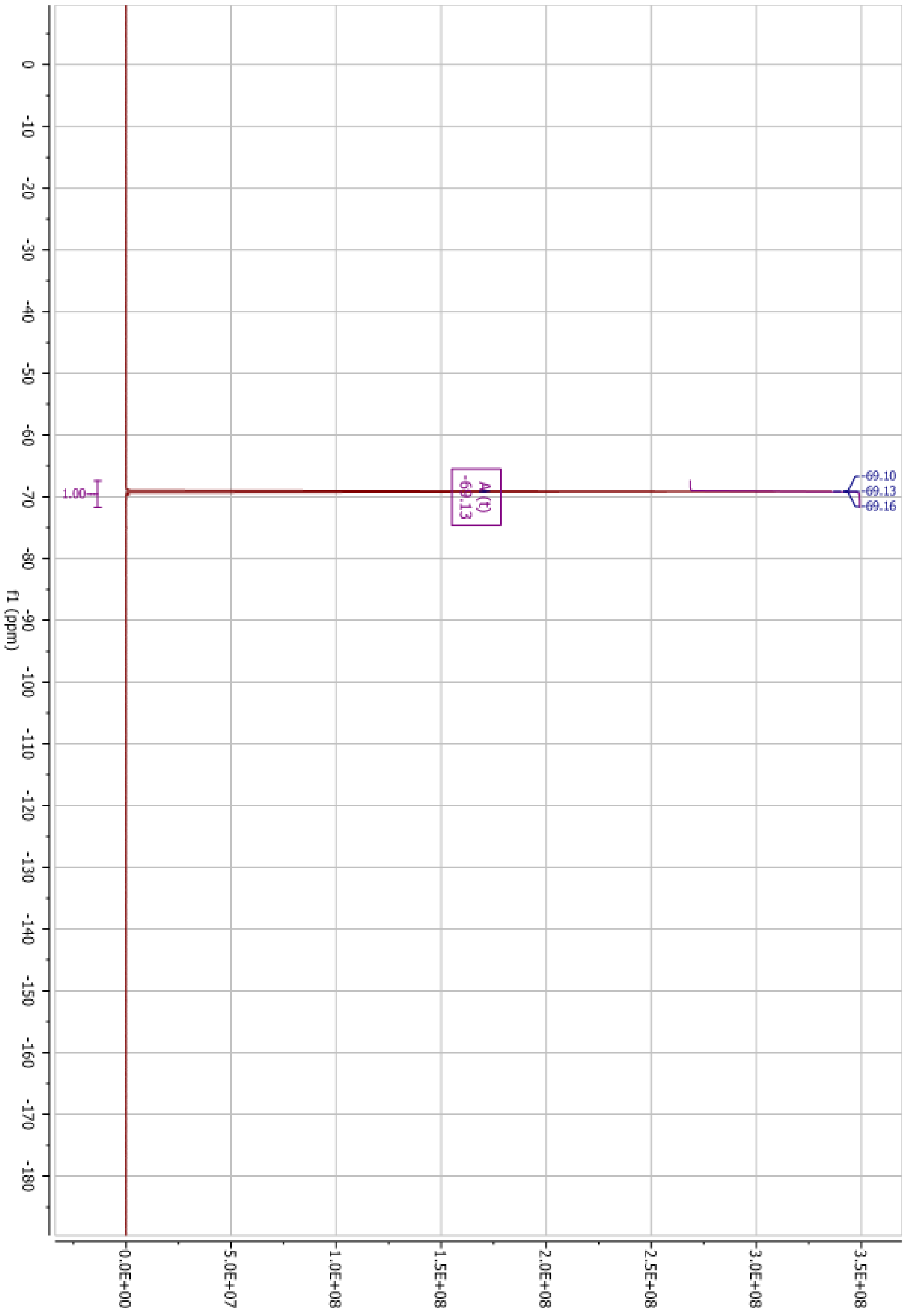

Supplementary Figure 77.

^{1}H-NMR (400 MHz, $CDCl_3$) *N*-(2-methoxyethyl)-2,2-dimethyl-*N*-(2,2,2-trifluoroethyl)propan-1-amine (**23**)

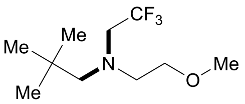

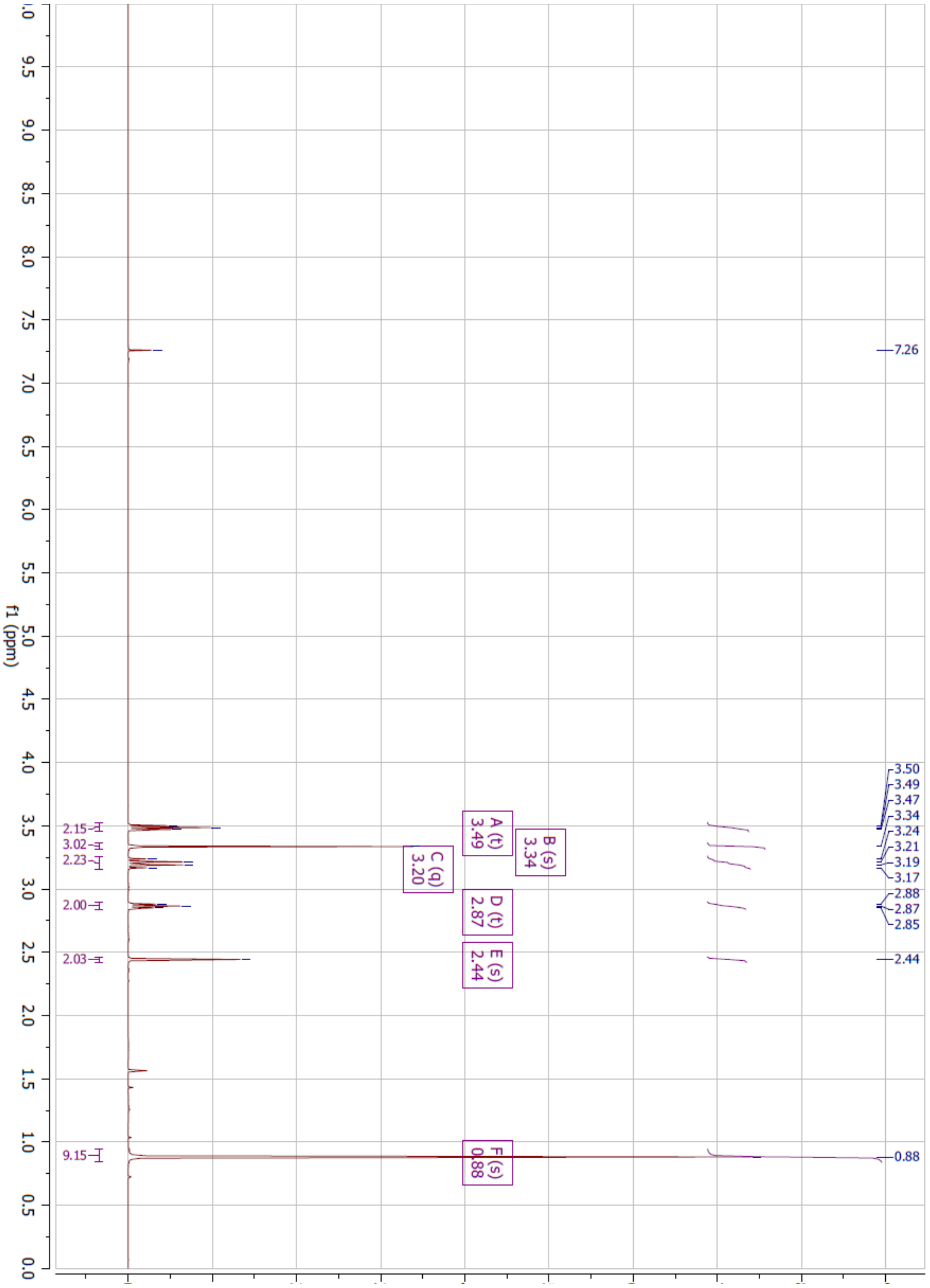

Supplementary Figure 78.

^{13}C-NMR (101 MHz, CDCl$_3$) *N*-(2-methoxyethyl)-2,2-dimethyl-*N*-(2,2,2-trifluoroethyl)propan-1-amine (**23**)

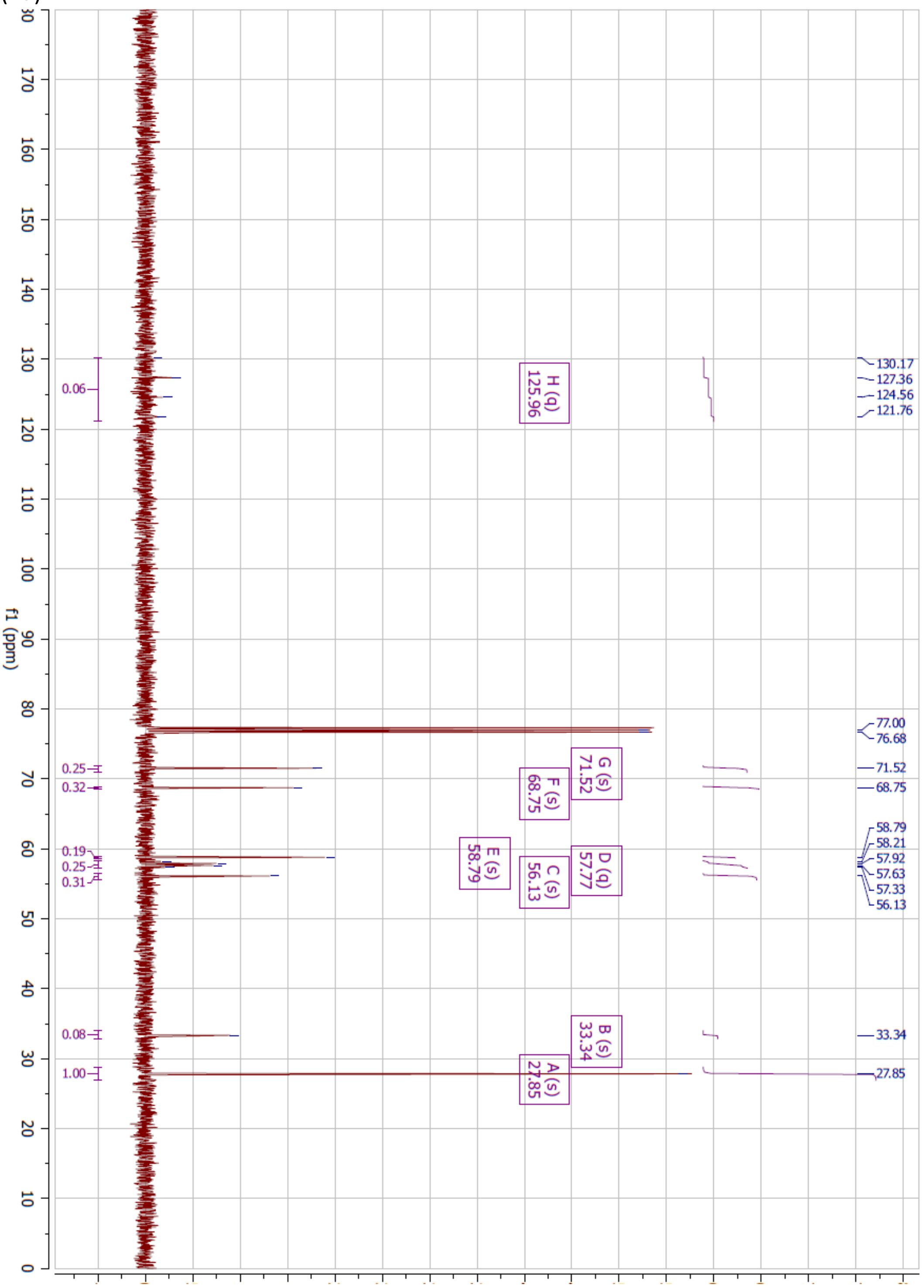

Supplementary Figure 79.
^{19}F-NMR (282 MHz, $CDCl_3$)) *N*-(2-methoxyethyl)-2,2-dimethyl-*N*-(2,2,2-trifluoroethyl)propan-1-amine (**23**)

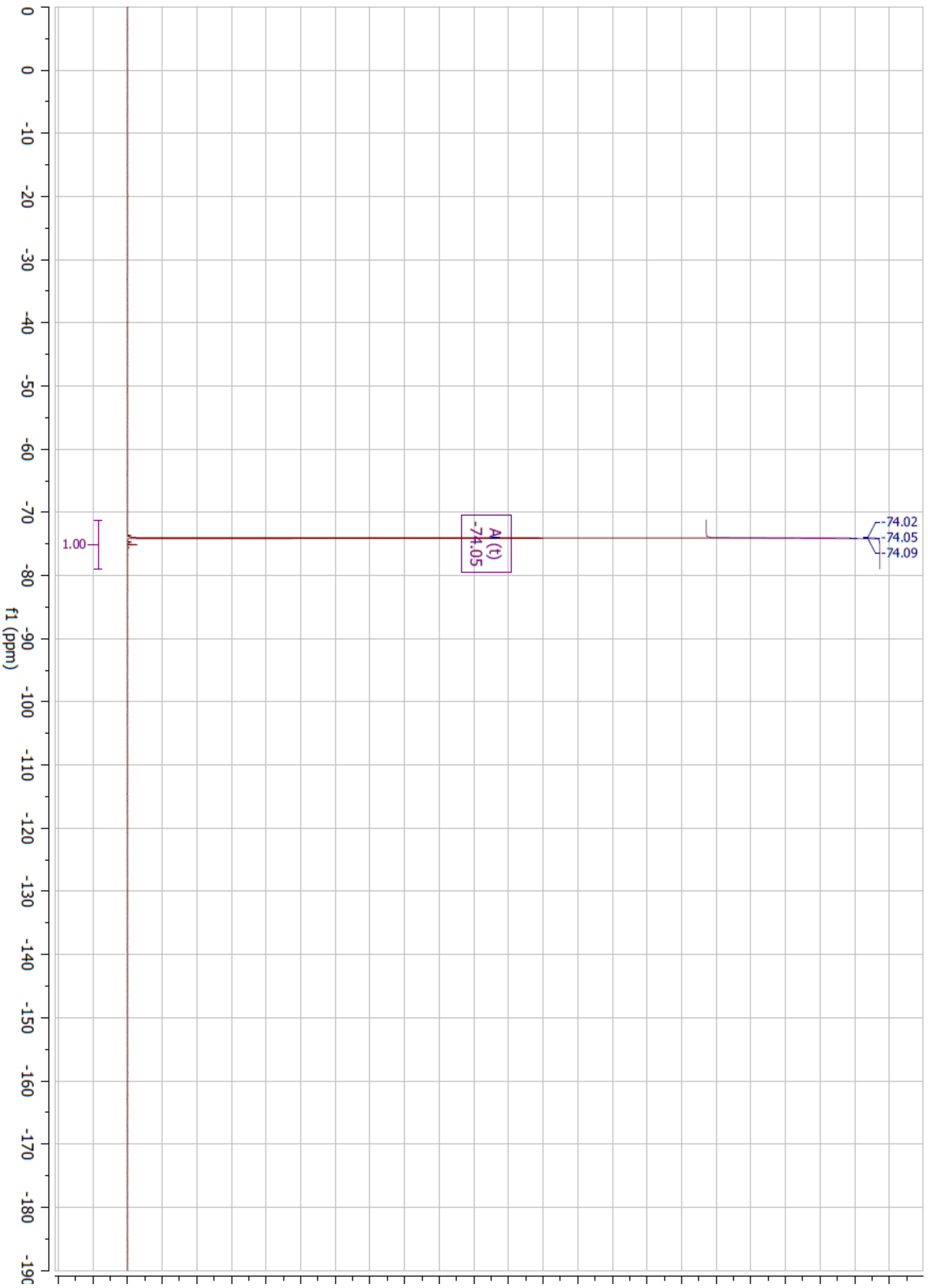

Supplementary Figure 80.

^{1}H-NMR (400 MHz, $CDCl_3$) *N*-(4-methoxybenzyl)-2-methyl-*N*-(2,2,2-trifluoroethyl)propan-2-amine (**24**)

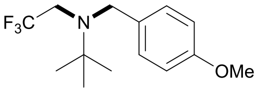

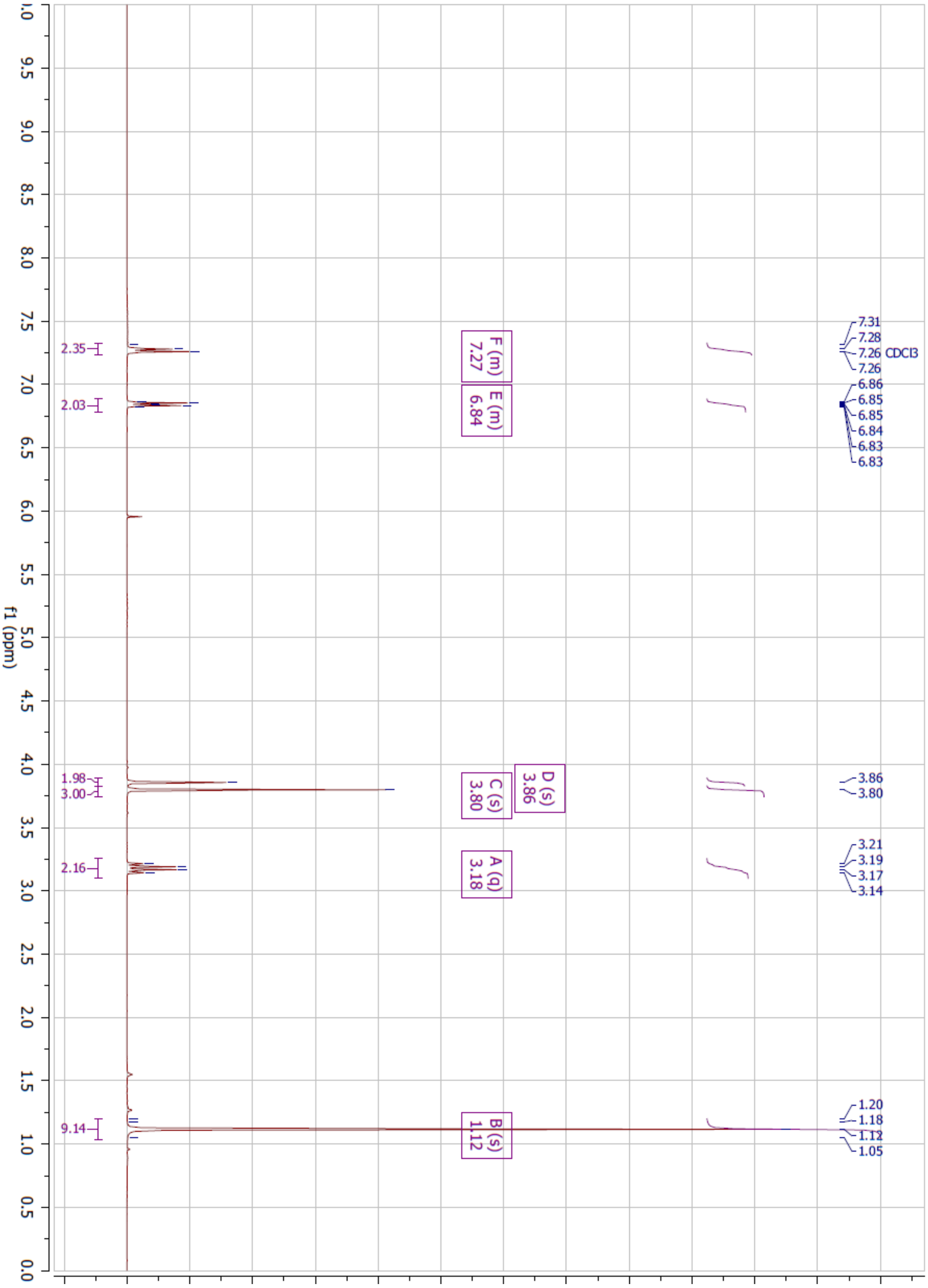

Supplementary Figure 81.

^{13}C-NMR (101 MHz, $CDCl_3$) *N*-(4-methoxybenzyl)-2-methyl-*N*-(2,2,2-trifluoroethyl)propan-2-amine (**24**)

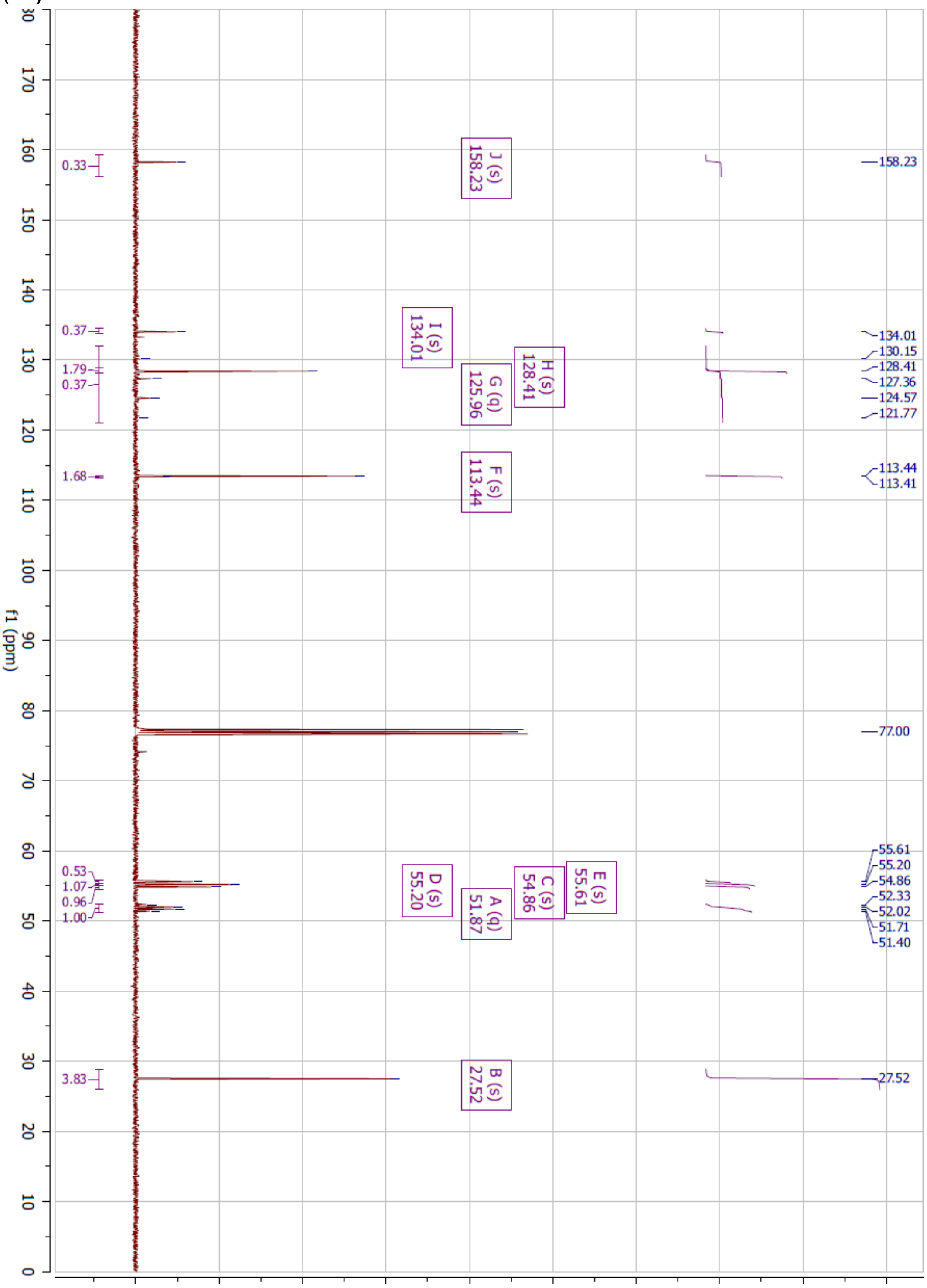

Supplementary Figure 82.
^{19}F-NMR (376 MHz, $CDCl_3$) *N*-(4-methoxybenzyl)-2-methyl-*N*-(2,2,2-trifluoroethyl)propan-2-amine (**24**)

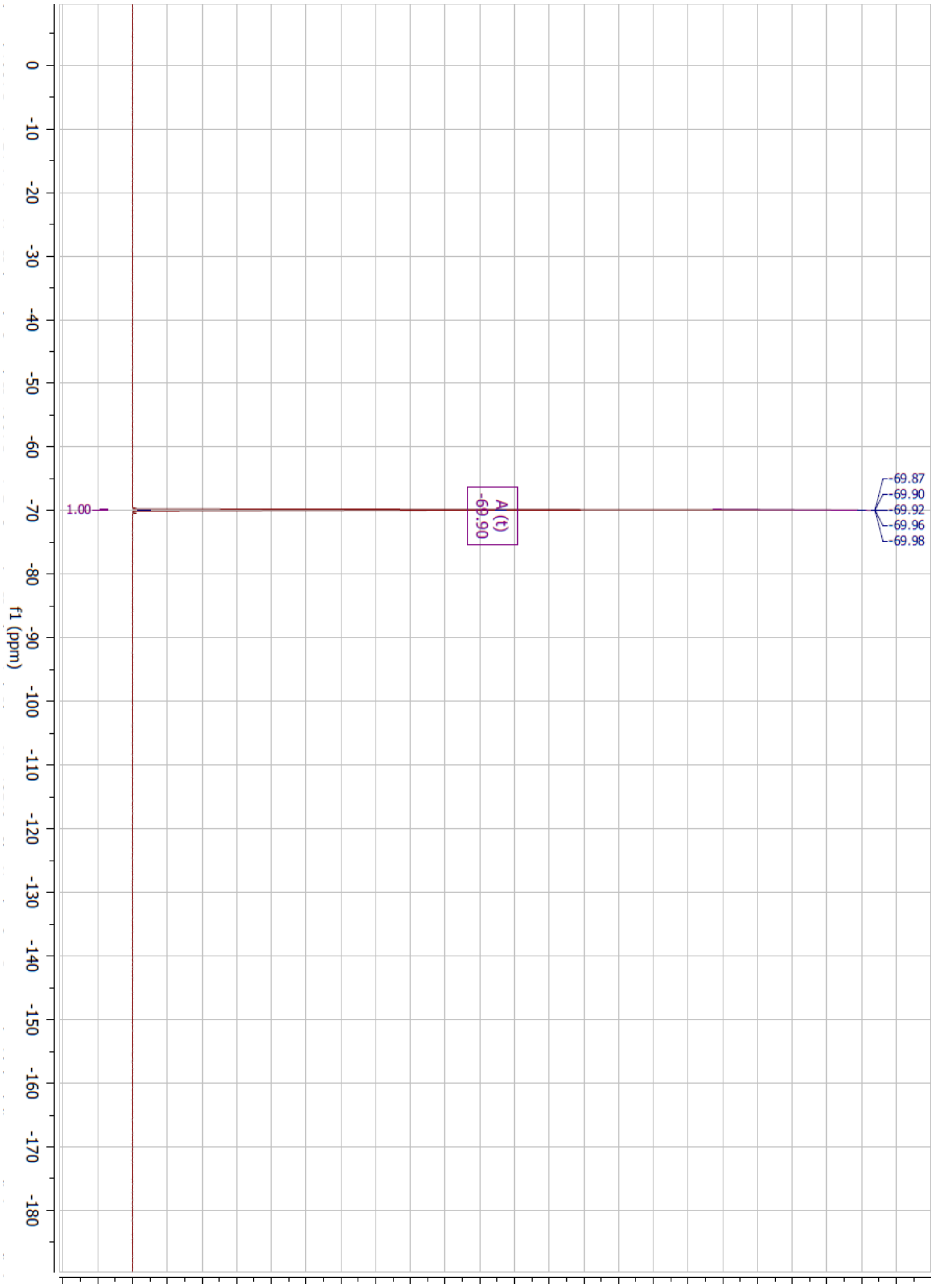

Supplementary Figure 83.

^{1}H-NMR (400 MHz, $CDCl_3$) (*R*)-*N*-(cyclohex-1-en-1-ylmethyl)-2,2,2-trifluoro-*N*-(1-phenylethyl)ethan-1-amine (**25**)

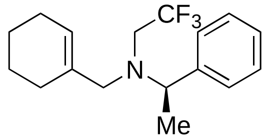

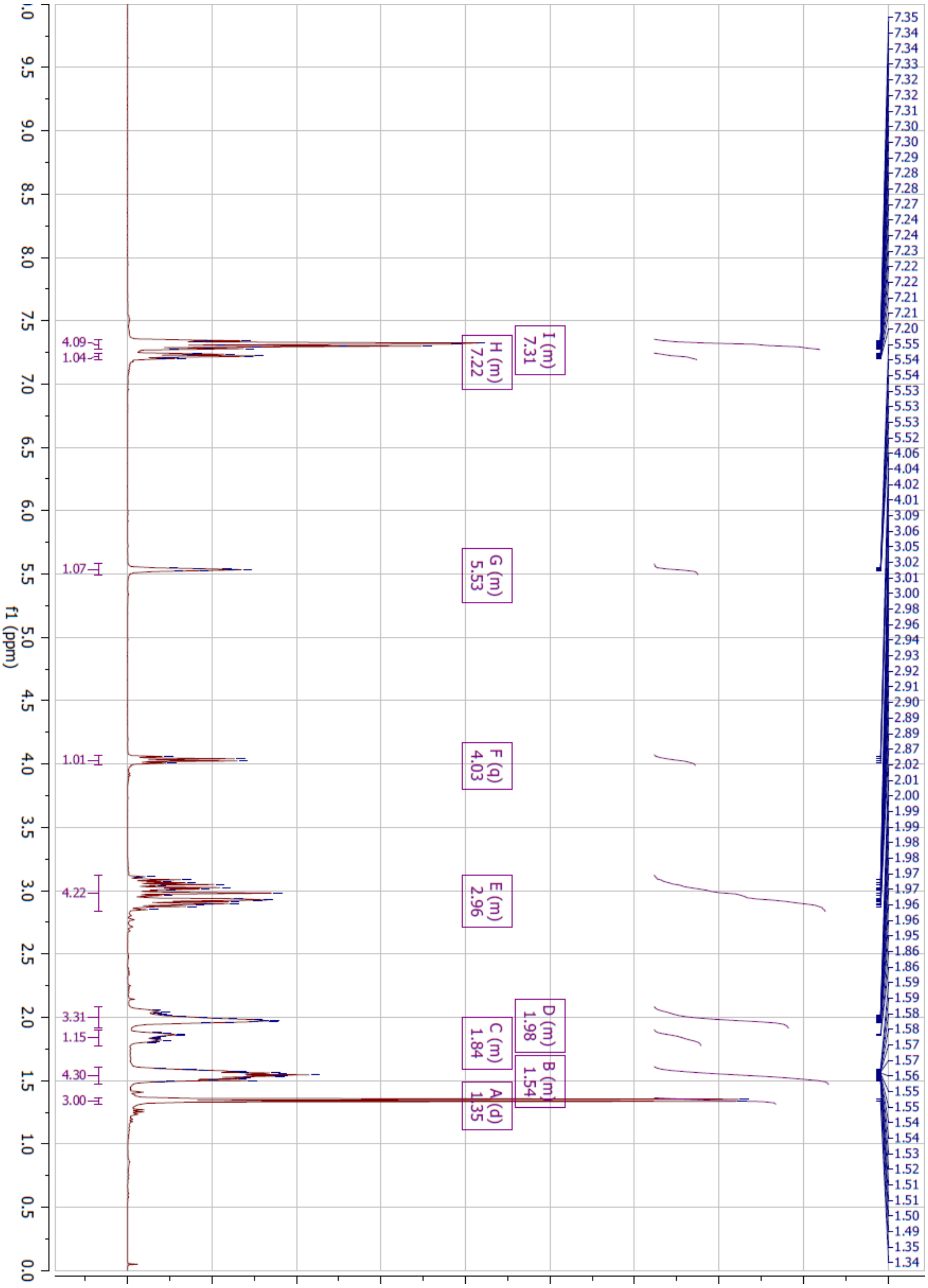

Supplementary Figure 84.

^{13}C-NMR (101 MHz, $CDCl_3$) *N*-(cyclohex-2-en-1-ylmethyl)-2,2,2-trifluoro-*N*-((*S*)-1-phenylethyl)ethan-1-amine (**25**)

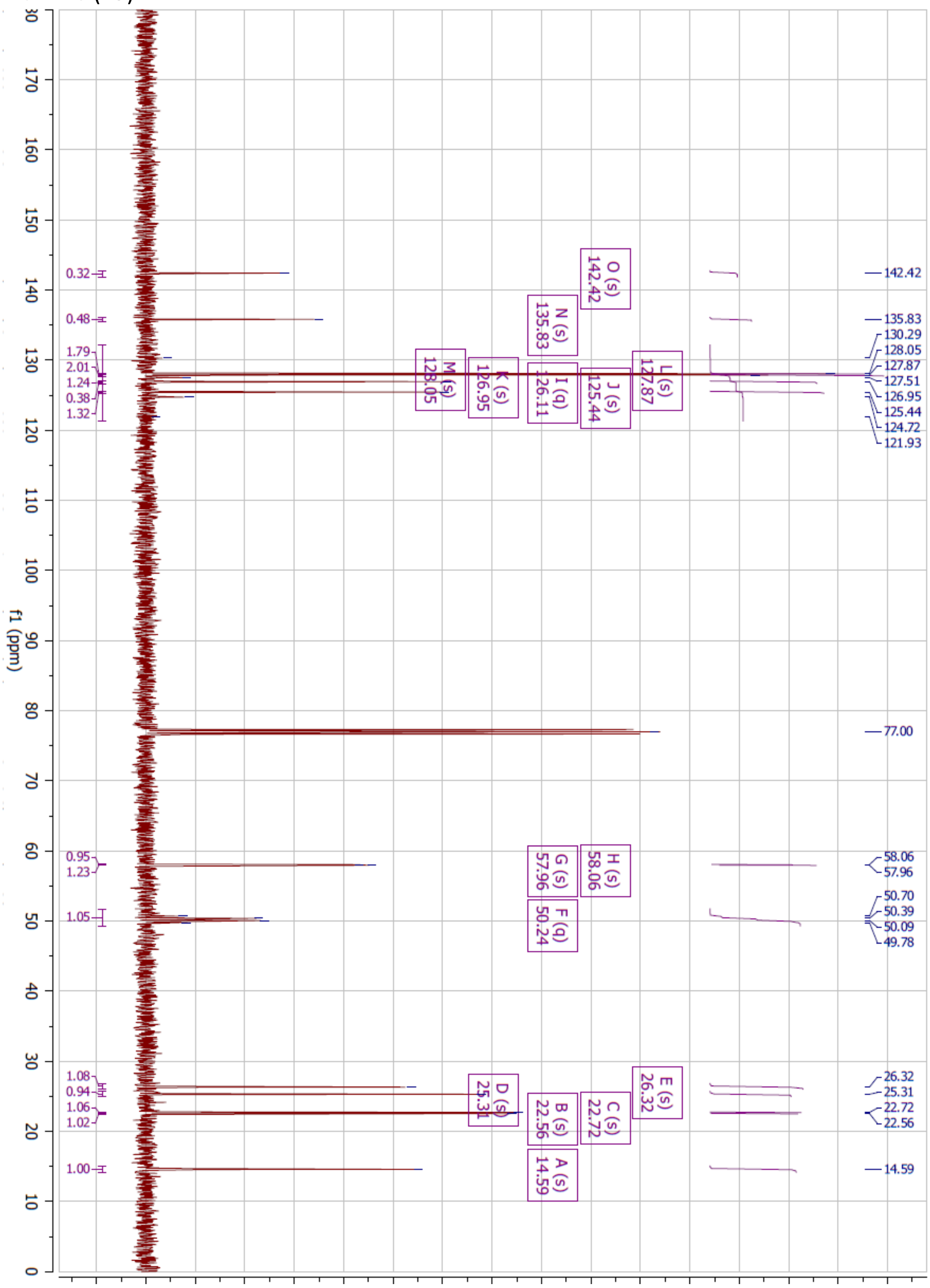

Supplementary Figure 85.
^{19}F-NMR (282 MHz, $CDCl_3$) *N*-(cyclohex-2-en-1-ylmethyl)-2,2,2-trifluoro-*N*-((*S*)-1-phenylethyl)ethan-1-amine (**25**)

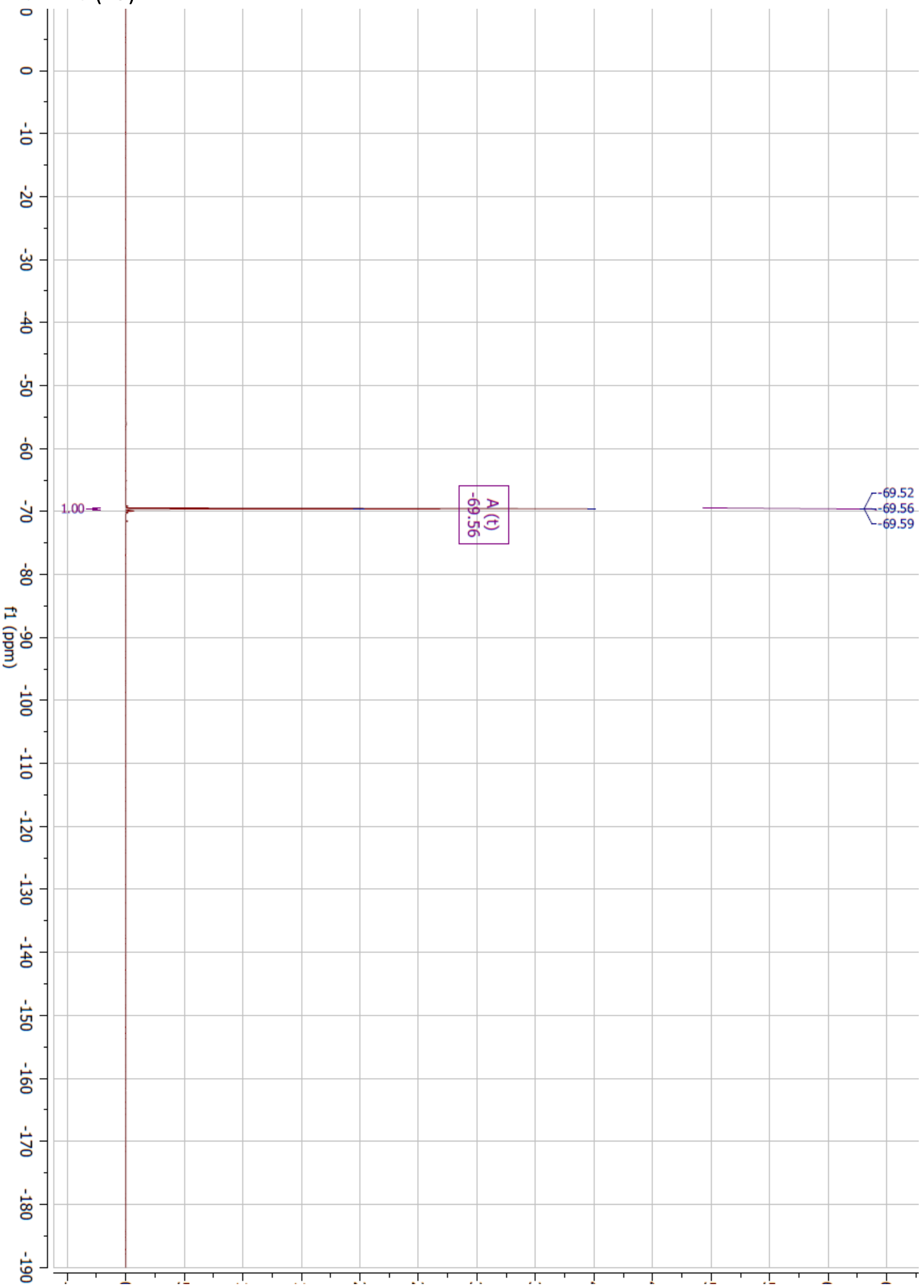

Supplementary Figure 86.

^{1}H-NMR (400 MHz, $CDCl_3$) methyl *N*-(4-bromo-2-methoxybenzyl)-*N*-(2,2,2-trifluoroethyl)-*L*-phenylalaninate (*ent*-**26**)

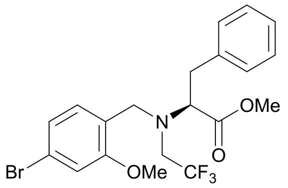

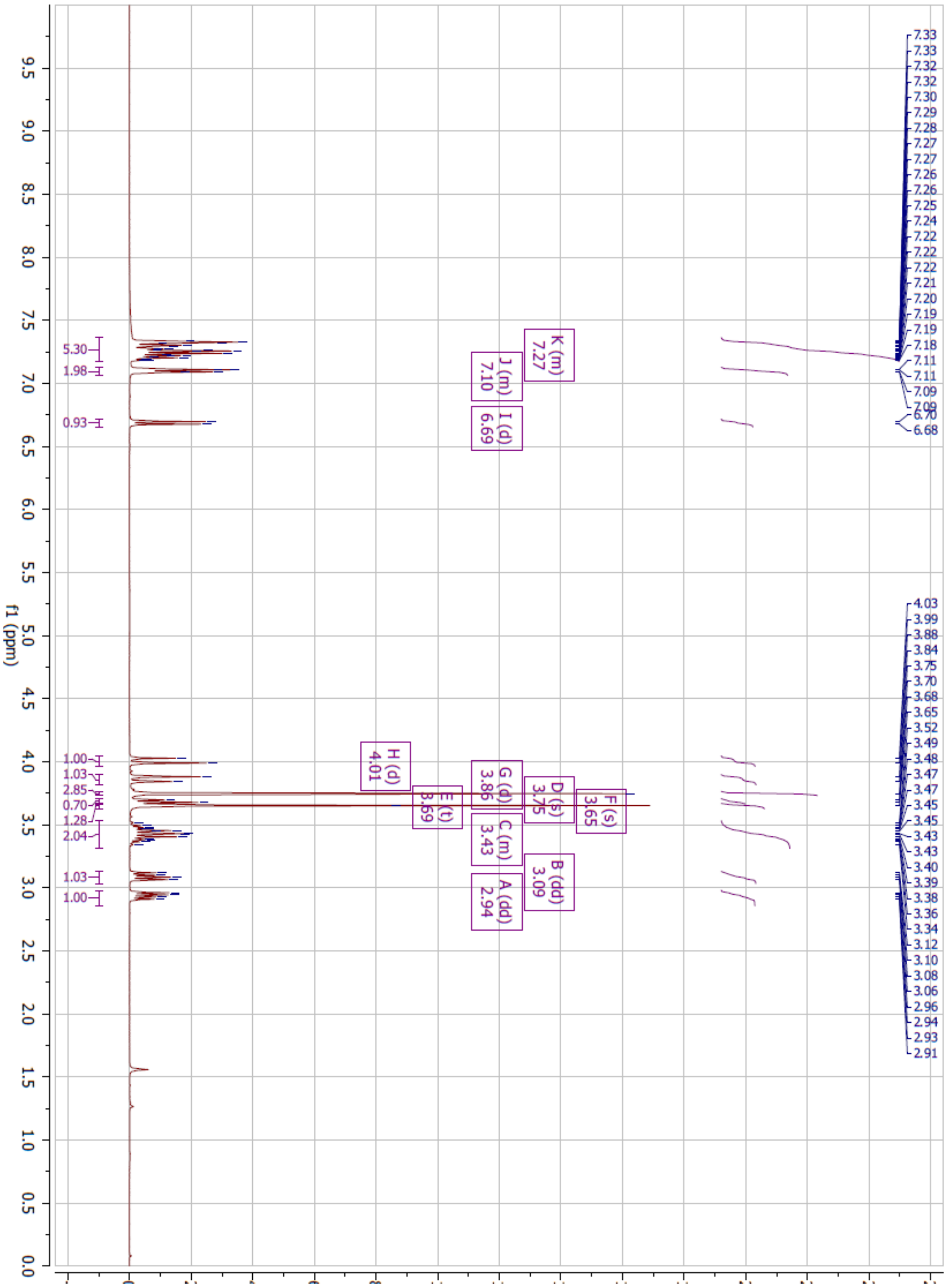

Supplementary Figure 87.
^{13}C-NMR (101 MHz, $CDCl_3$) methyl *N*-(4-bromo-2-methoxybenzyl)-*N*-(2,2,2-trifluoroethyl)-*L*-phenylalaninate (*ent*-**26**)

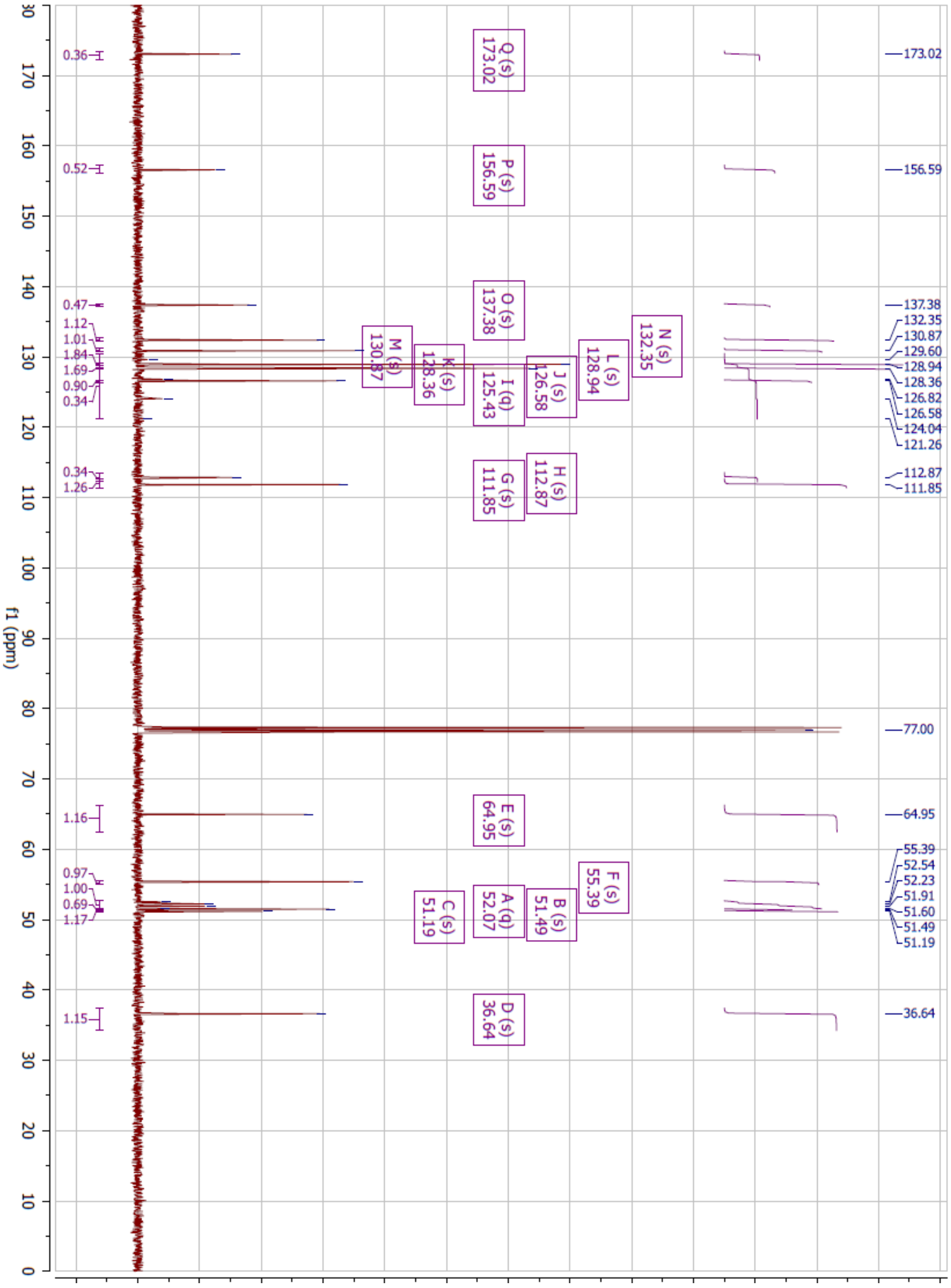

Supplementary Figure 88.
^{19}F-NMR (376 MHz, $CDCl_3$) methyl *N*-(4-bromo-2-methoxybenzyl)-*N*-(2,2,2-trifluoroethyl)-*L*-phenylalaninate (*ent*-**26**)

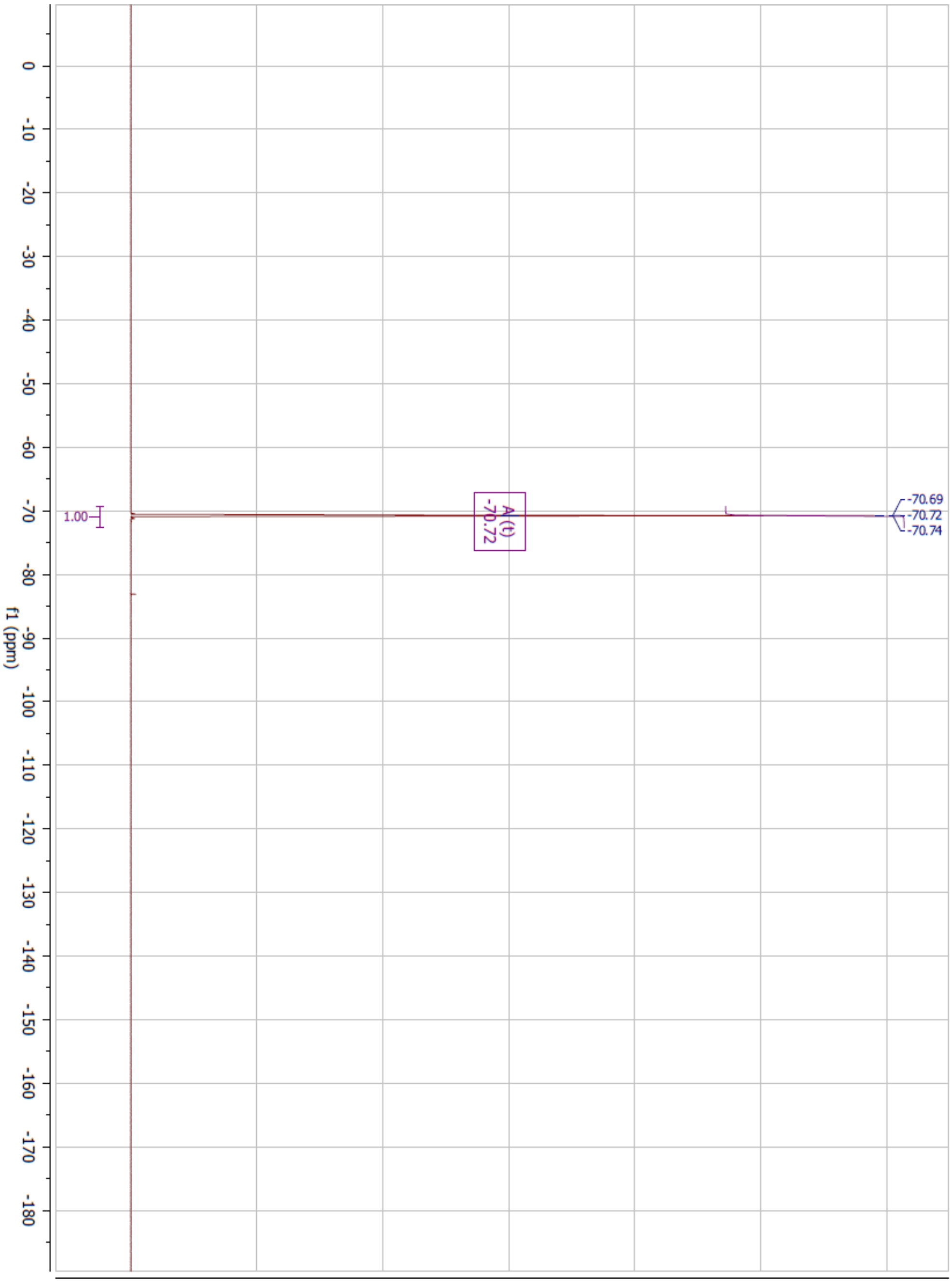

Supplementary Figure 89.

^{1}H-NMR (400 MHz, $CDCl_3$) (*E*)-*N*-(furan-2-ylmethyl)-3-phenyl-*N*-(2,2,2-trifluoroethyl)prop-2-en-1-amine (**27**)

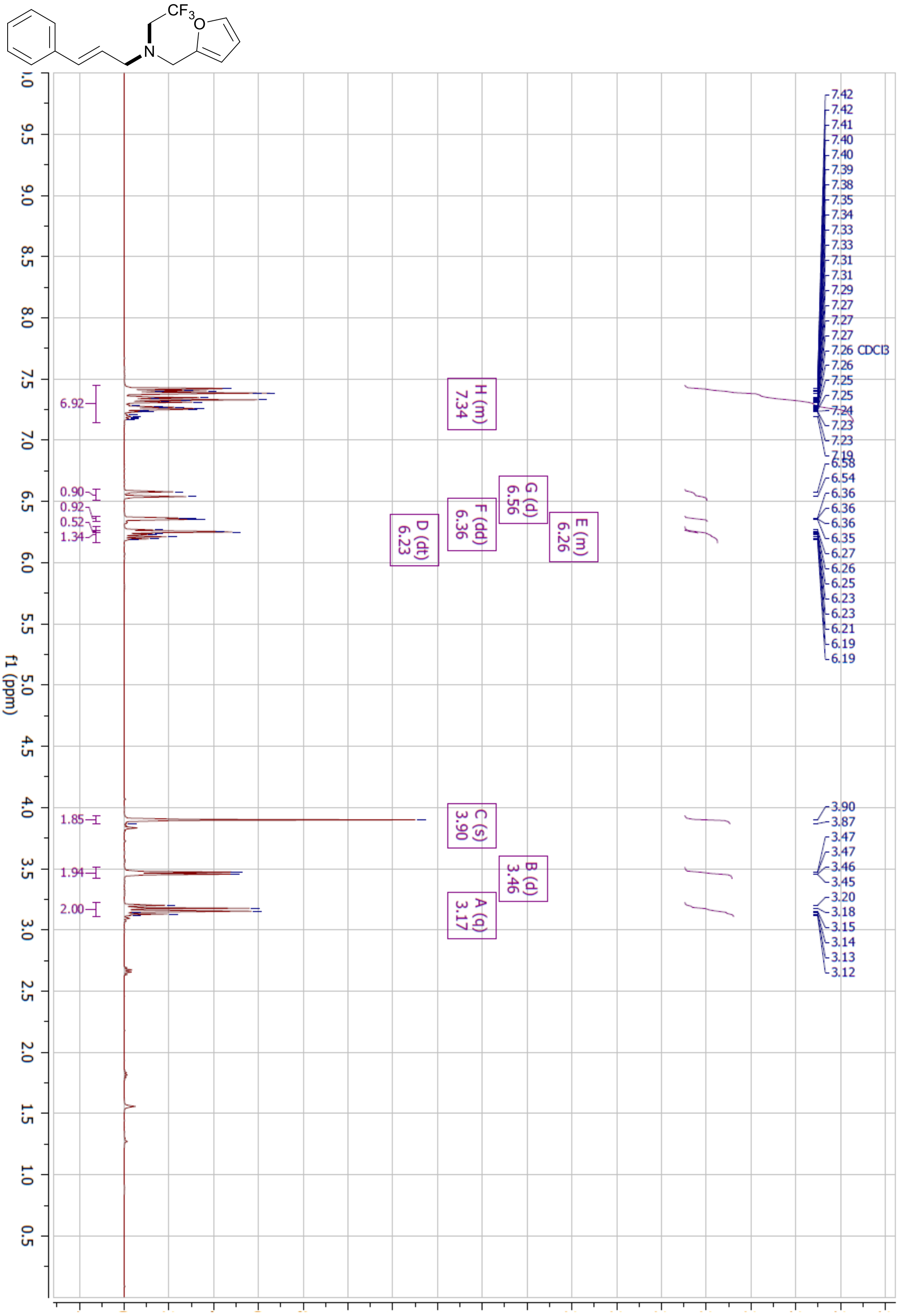

Supplementary Figure 90.
^{13}C-NMR (101 MHz, $CDCl_3$) (*E*)-*N*-(furan-2-ylmethyl)-3-phenyl-*N*-(2,2,2-trifluoroethyl)prop-2-en-1-amine (**27**)

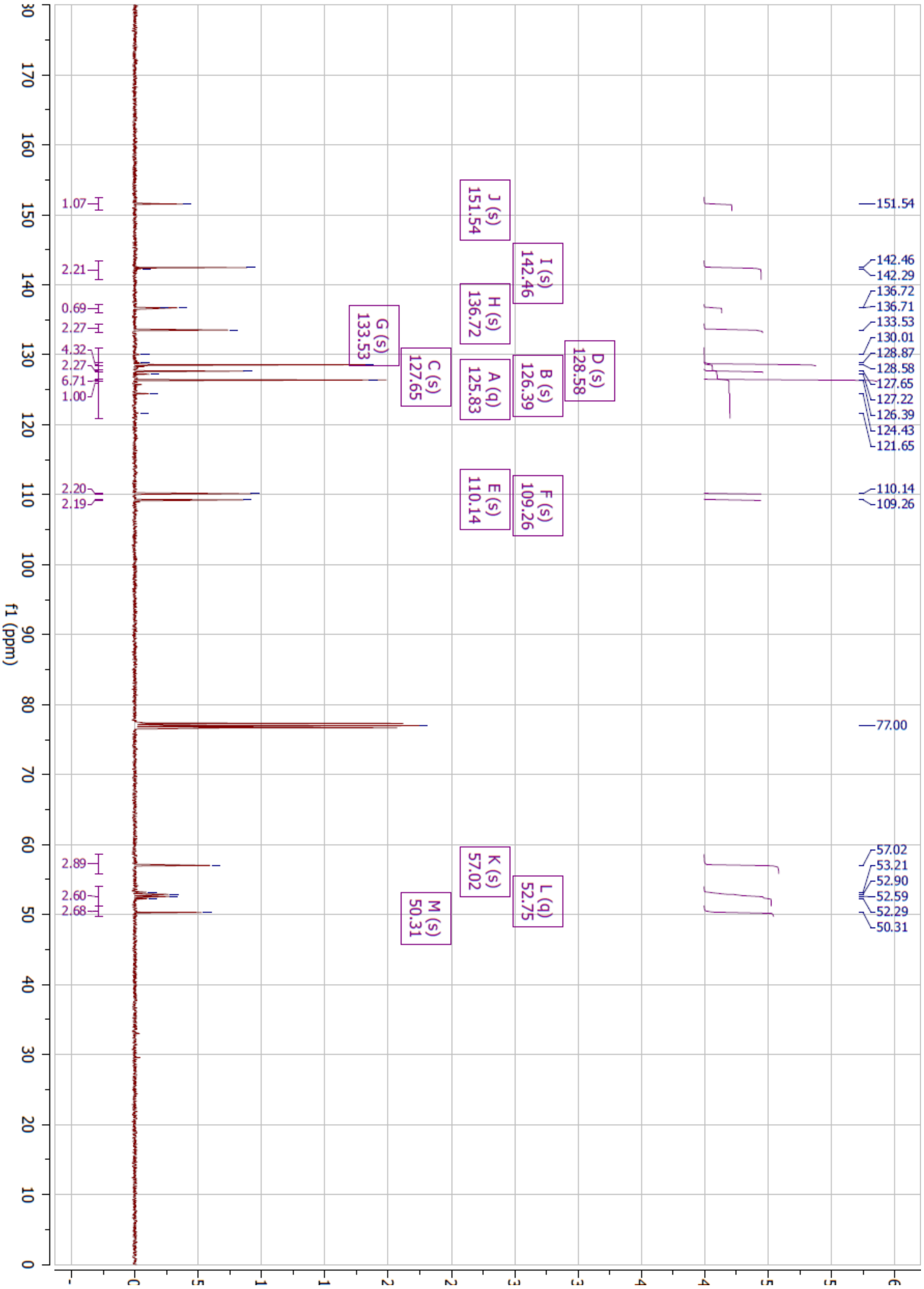

Supplementary Figure 91.

^{19}F-NMR (376 MHz, $CDCl_3$) (*E*)-*N*-(furan-2-ylmethyl)-3-phenyl-*N*-(2,2,2-trifluoroethyl)prop-2-en-1-amine (**27**)

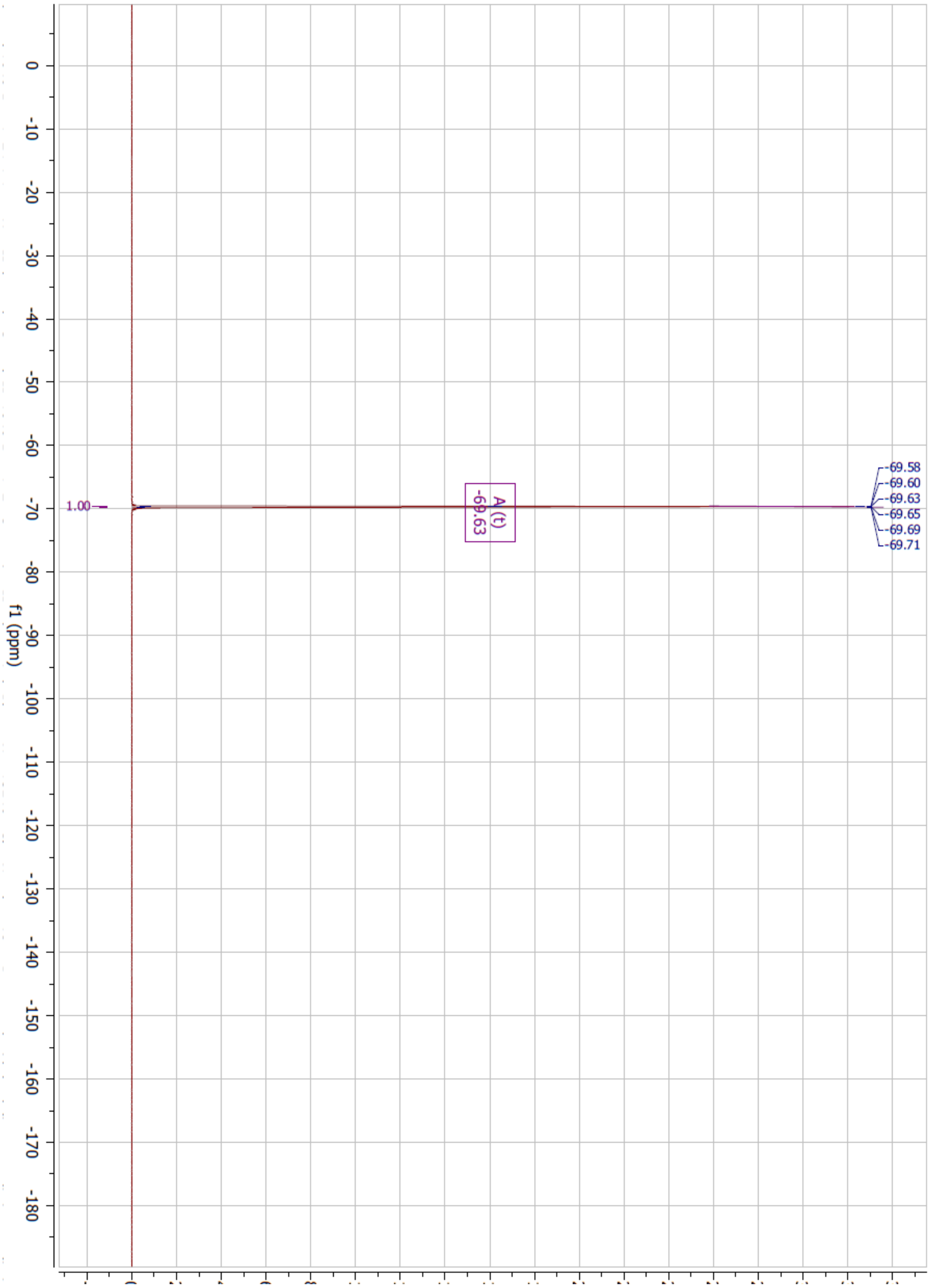

Supplementary Figure 92.

^{1}H-NMR (400 MHz, $CDCl_3$) *N*-((5-bromofuran-2-yl)methyl)-*N*-(2-chlorobenzyl)-2,2,2-trifluoroethan-1-amine (**28**)

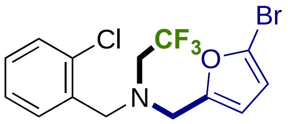

28: 43%

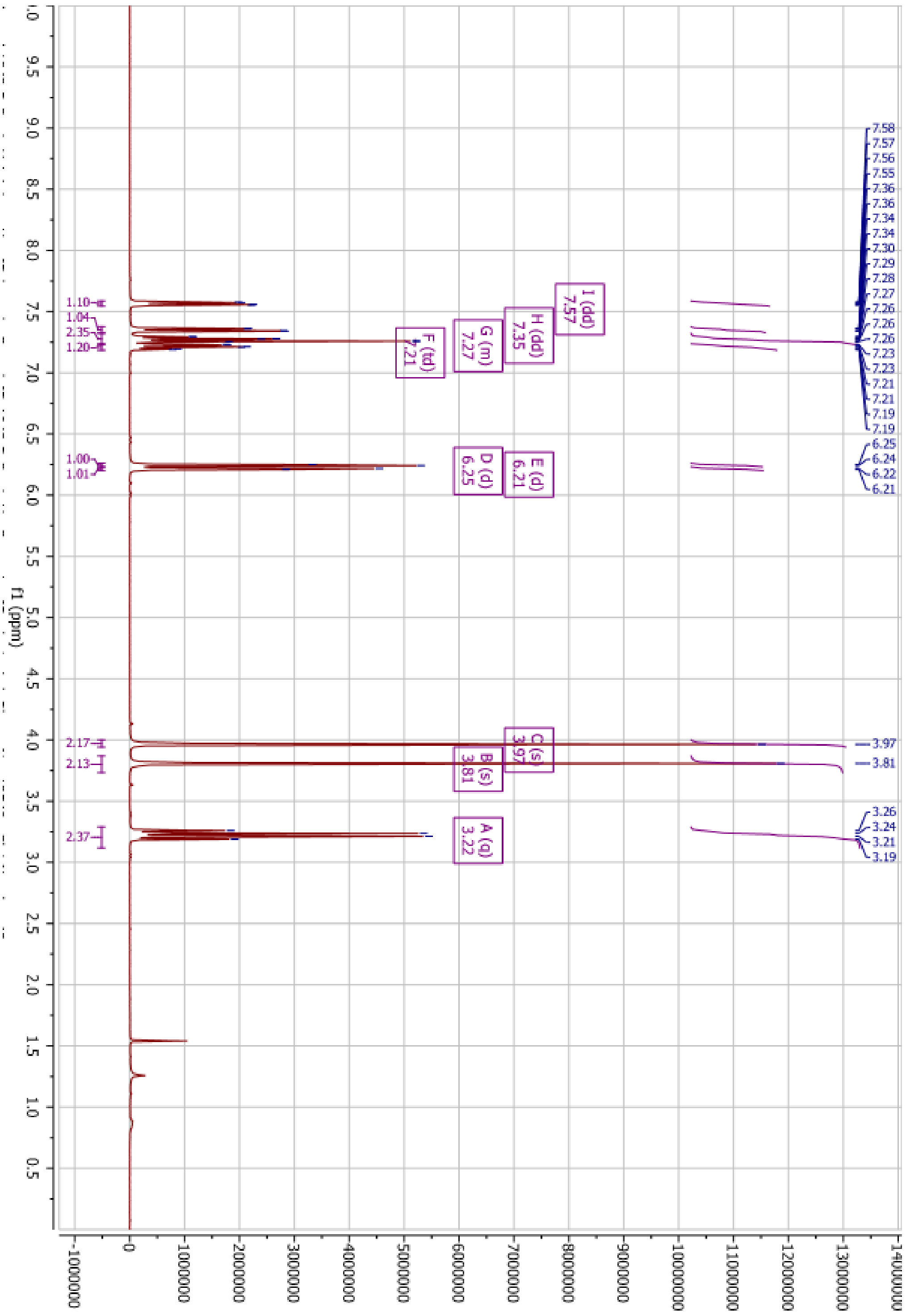

Supplementary Figure 93.

^{13}C-NMR (101 MHz, CDCl$_3$) *N*-((5-bromofuran-2-yl)methyl)-*N*-(2-chlorobenzyl)-2,2,2-trifluoroethan-1-amine (**28**)

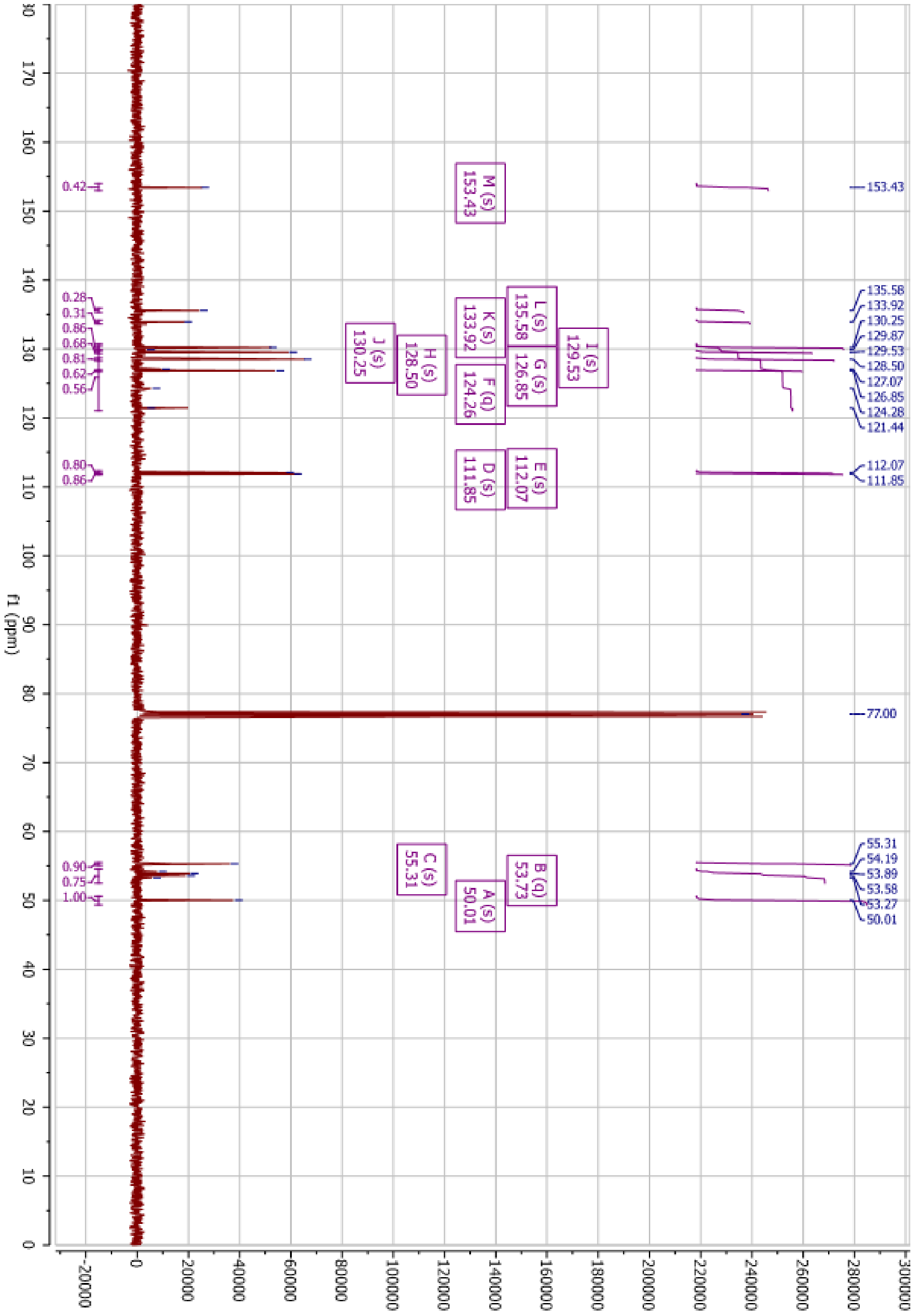

Supplementary Figure 94.
^{19}F-NMR (376 MHz, $CDCl_3$) *N*-((5-bromofuran-2-yl)methyl)-*N*-(2-chlorobenzyl)-2,2,2-trifluoroethan-1-amine (**28**)

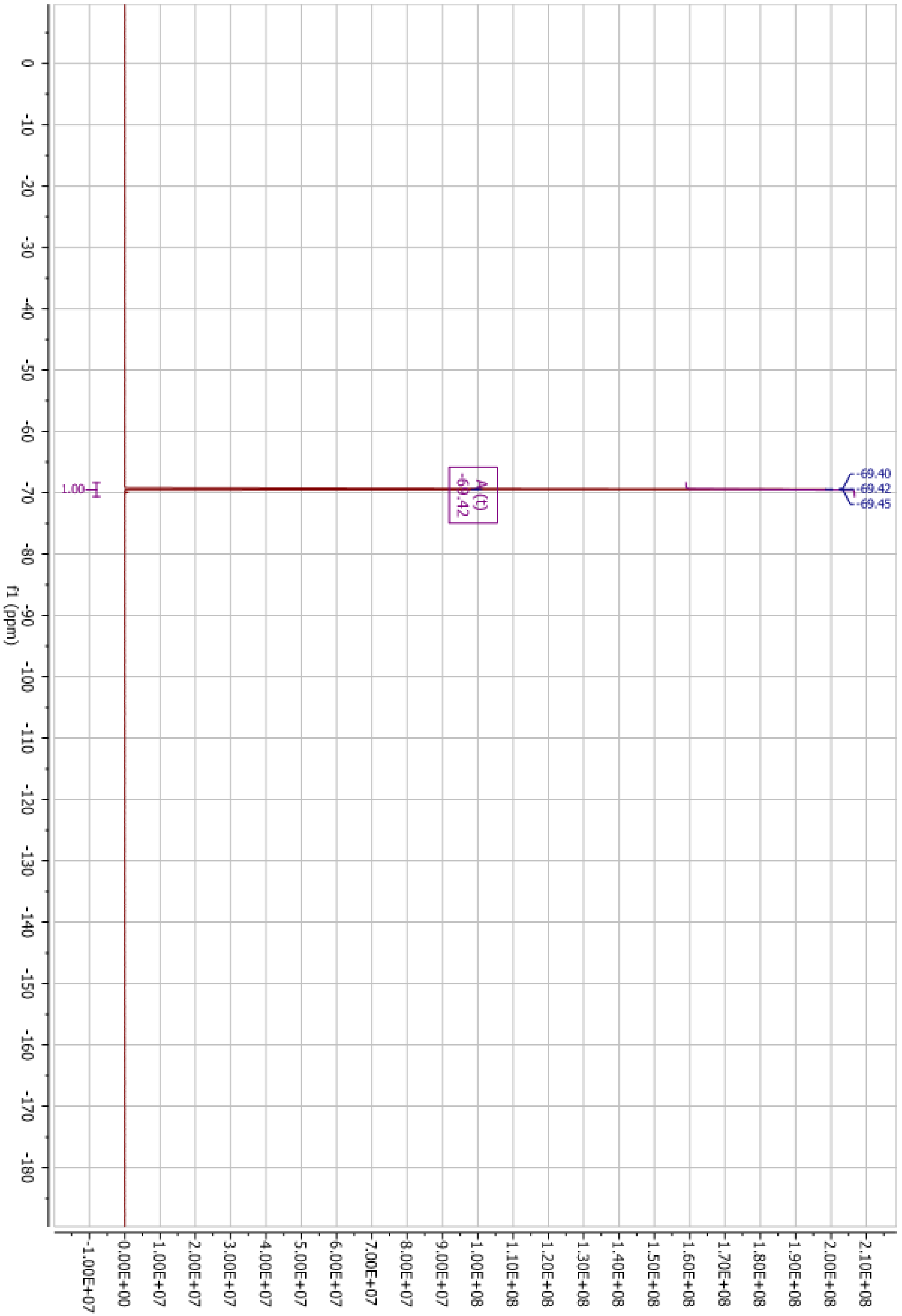

Supplementary Figure 95.
^{1}H-NMR (400 MHz, $CDCl_3$) *N*-((5-bromothiophen-2-yl)methyl)-3-methyl-*N*-(2,2,2-trifluoroethyl)butan-1-amine (**29**)

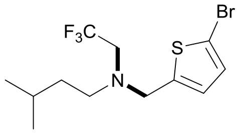

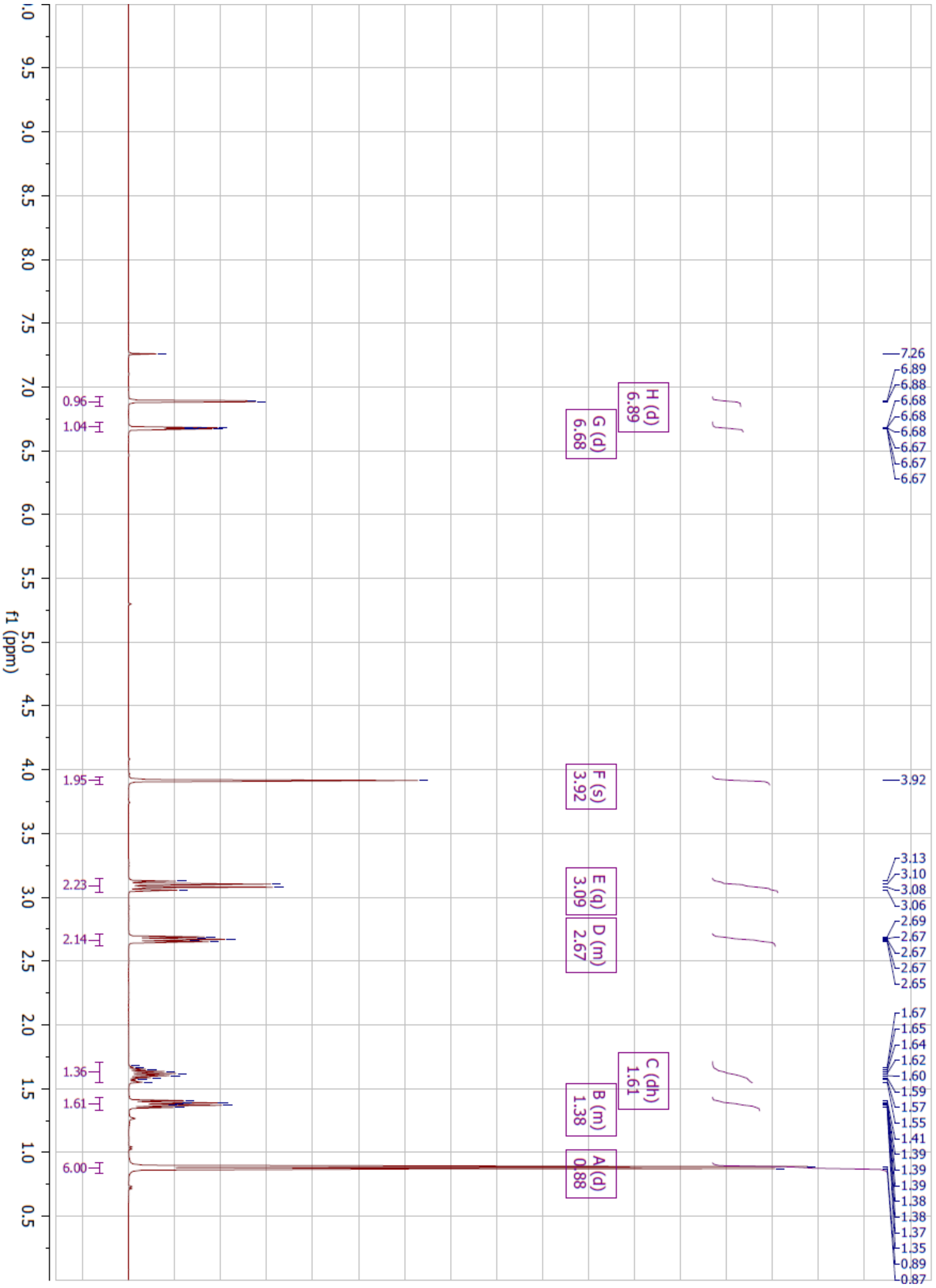

Supplementary Figure 96.
^{13}C-NMR (101 MHz, $CDCl_3$) *N*-((5-bromothiophen-2-yl)methyl)-3-methyl-*N*-(2,2,2-trifluoroethyl)butan-1-amine (**29**)

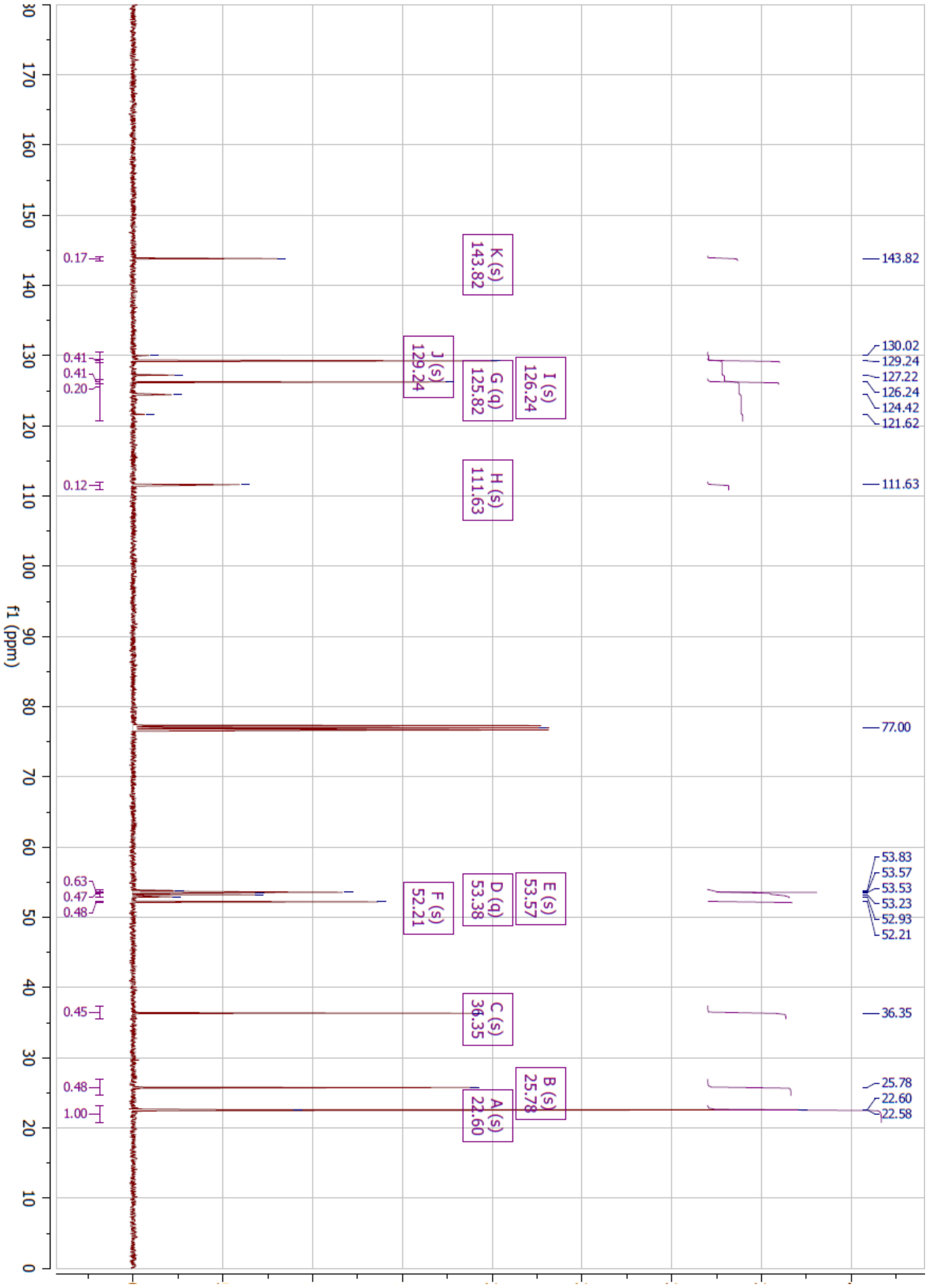

Supplementary Figure 97.
^{19}F-NMR (376 MHz, $CDCl_3$) *N*-((5-bromothiophen-2-yl)methyl)-3-methyl-*N*-(2,2,2-trifluoroethyl)butan-1-amine (**29**)

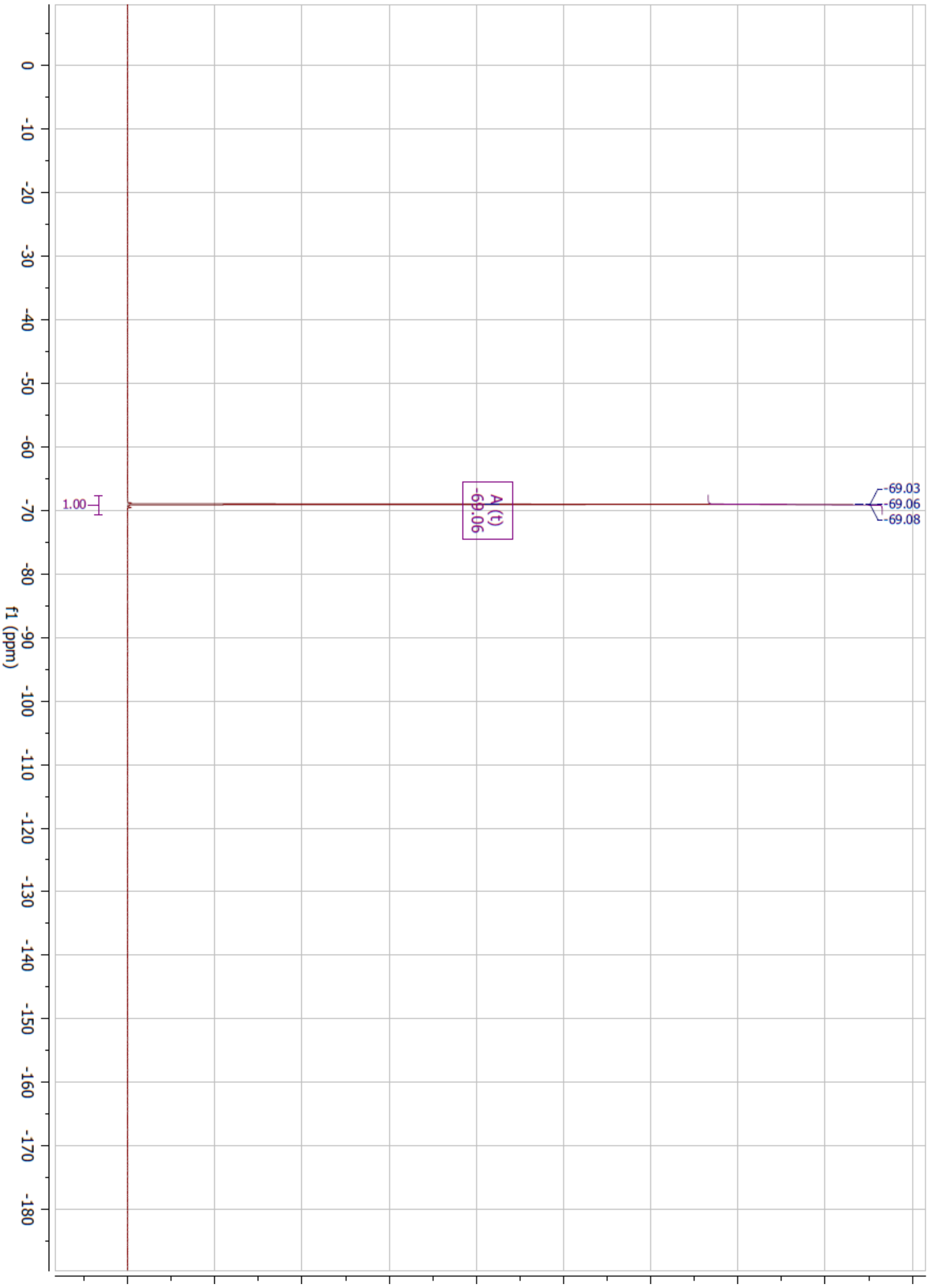

Supplementary Figure 98.

^{1}H-NMR (400 MHz, $CDCl_3$) (*E*)-3-(4-methoxyphenyl)-*N*-(3-phenylpropyl)-*N*-(2,2,2-trifluoroethyl)prop-2-en-1-amine (**30**)

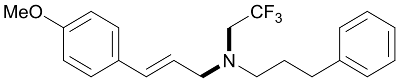

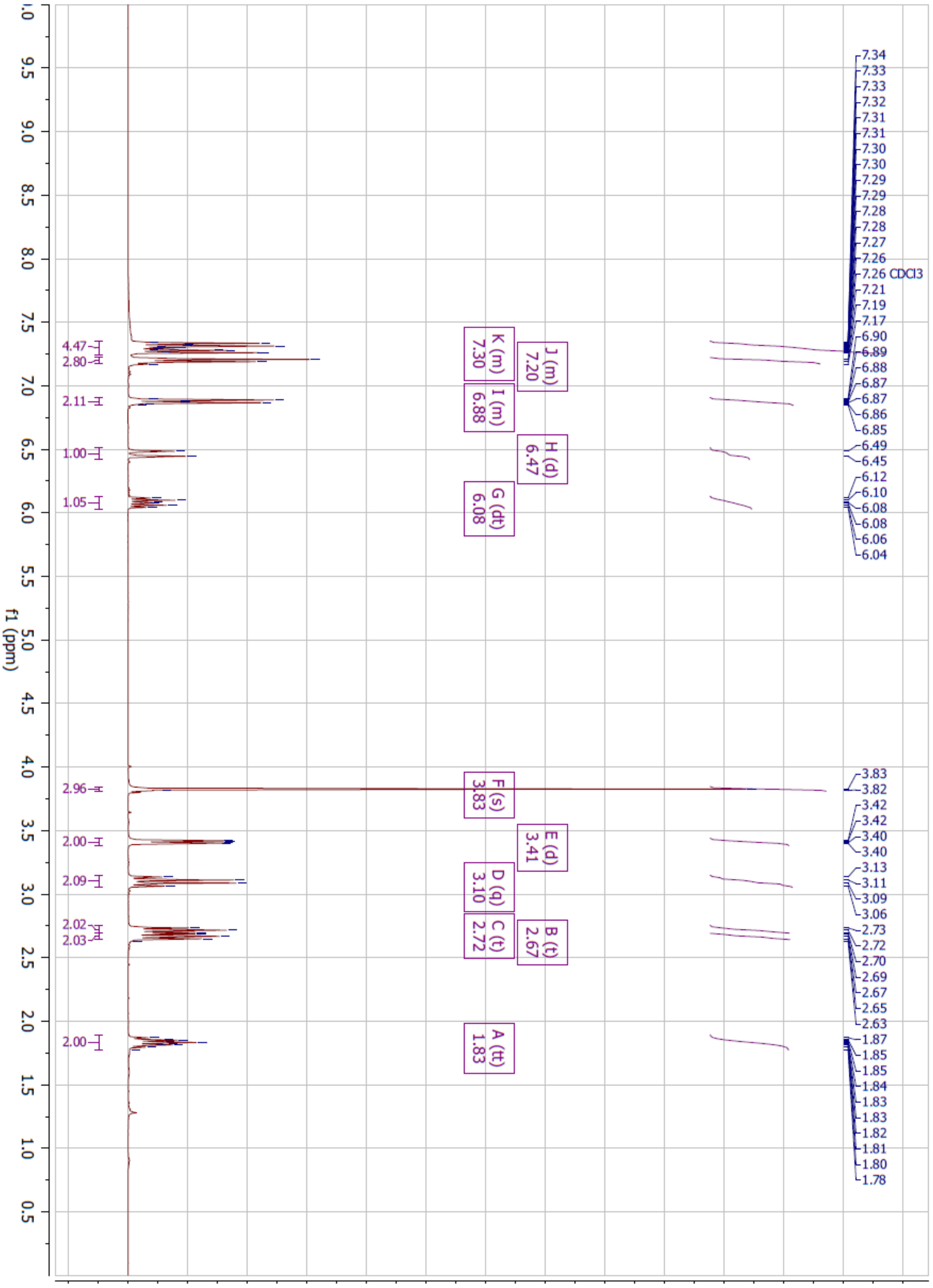

Supplementary Figure 99.
^{13}C-NMR (101 MHz, $CDCl_3$) (*E*)-3-(4-methoxyphenyl)-*N*-(3-phenylpropyl)-*N*-(2,2,2-trifluoroethyl)prop-2-en-1-amine (**30**)

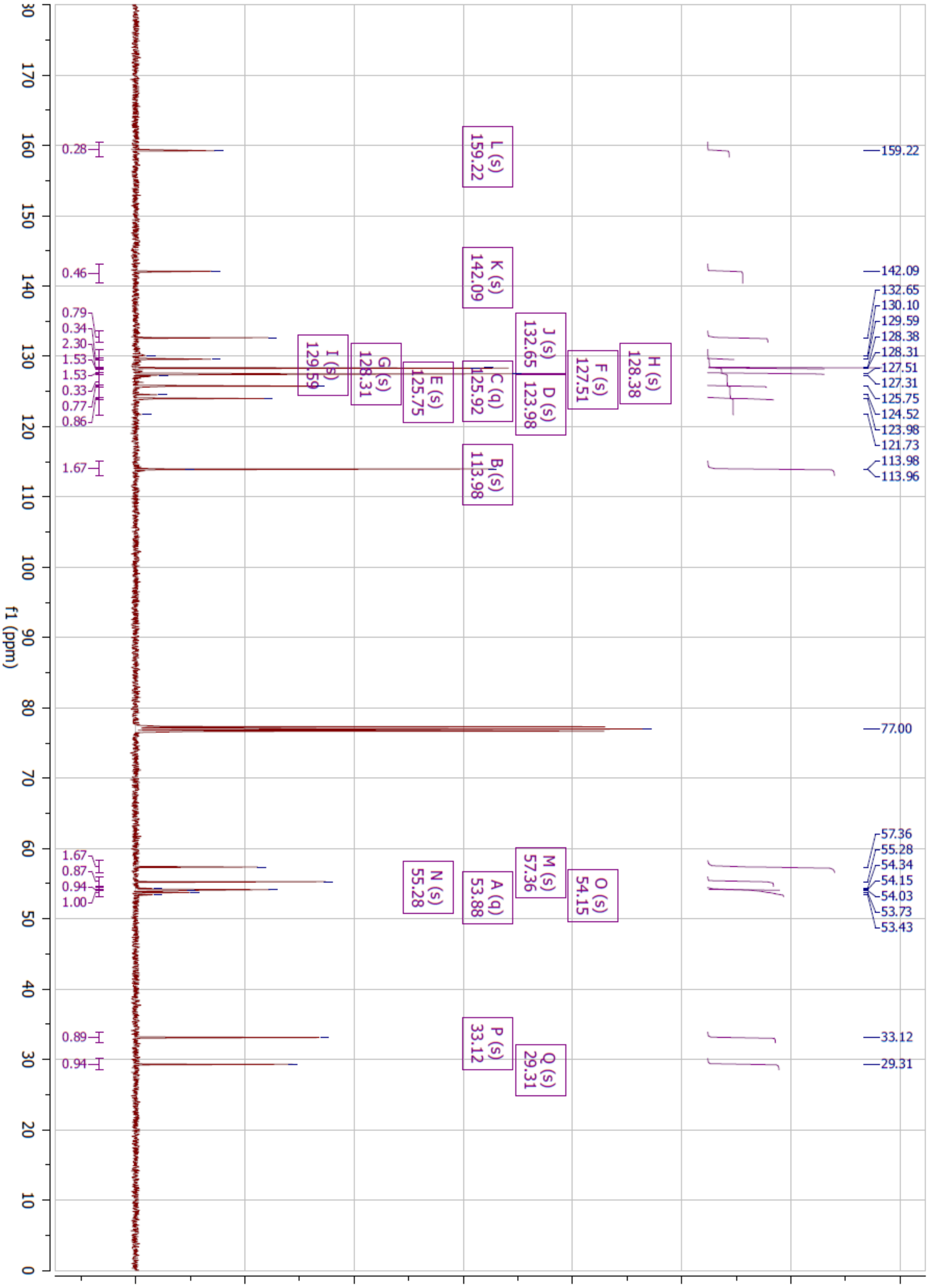

Supplementary Figure 100.

^{19}F-NMR (376 MHz, $CDCl_3$) (*E*)-3-(4-methoxyphenyl)-*N*-(3-phenylpropyl)-*N*-(2,2,2-trifluoroethyl)prop-2-en-1-amine (**30**)

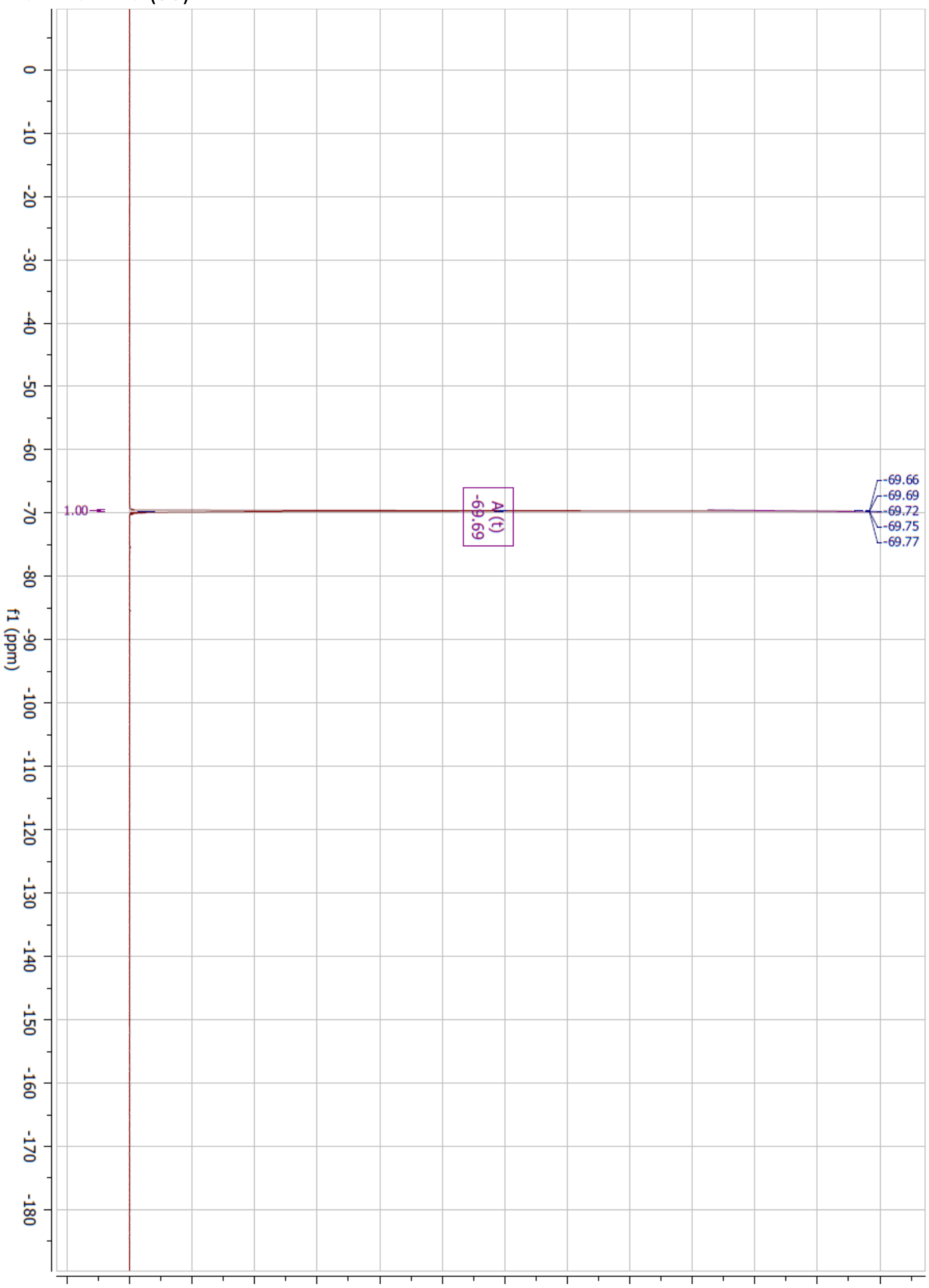

Supplementary Figure 101.

^{1}H-NMR (400 MHz, $CDCl_3$) *(E)*-*N*-(2,2,2-trifluoroethyl)-*N*-(undec-2-en-1-yl)cyclohexanamine *(major: 93%)* (**31**)

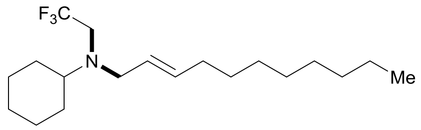

as 93:7 mixture of trans:cis

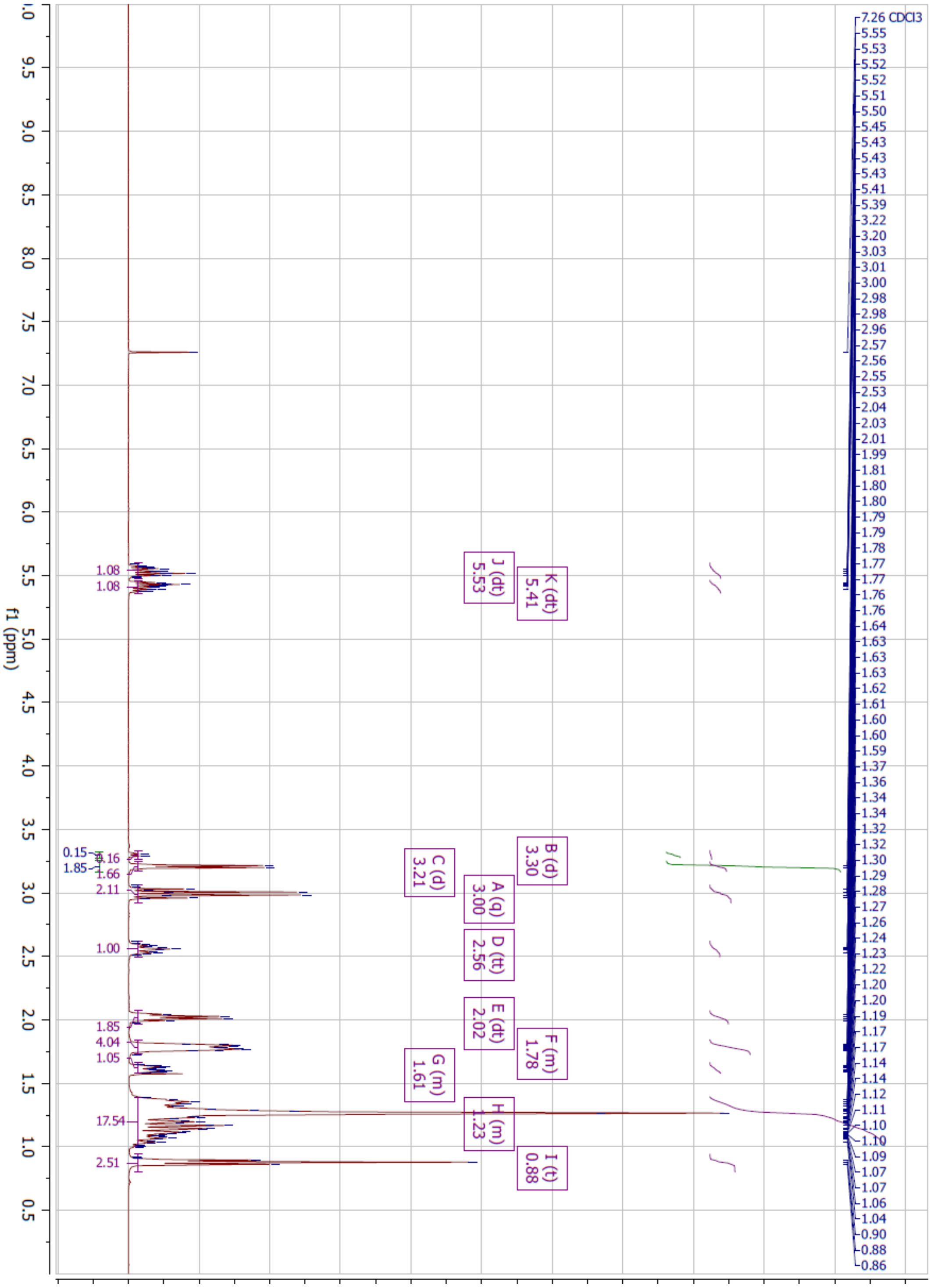

Supplementary Figure 102.
^{13}C-NMR (101 MHz, $CDCl_3$) *(E)*-*N*-(2,2,2-trifluoroethyl)-*N*-(undec-2-en-1-yl)cyclohexanamine *(major: 93%)* (**31**)

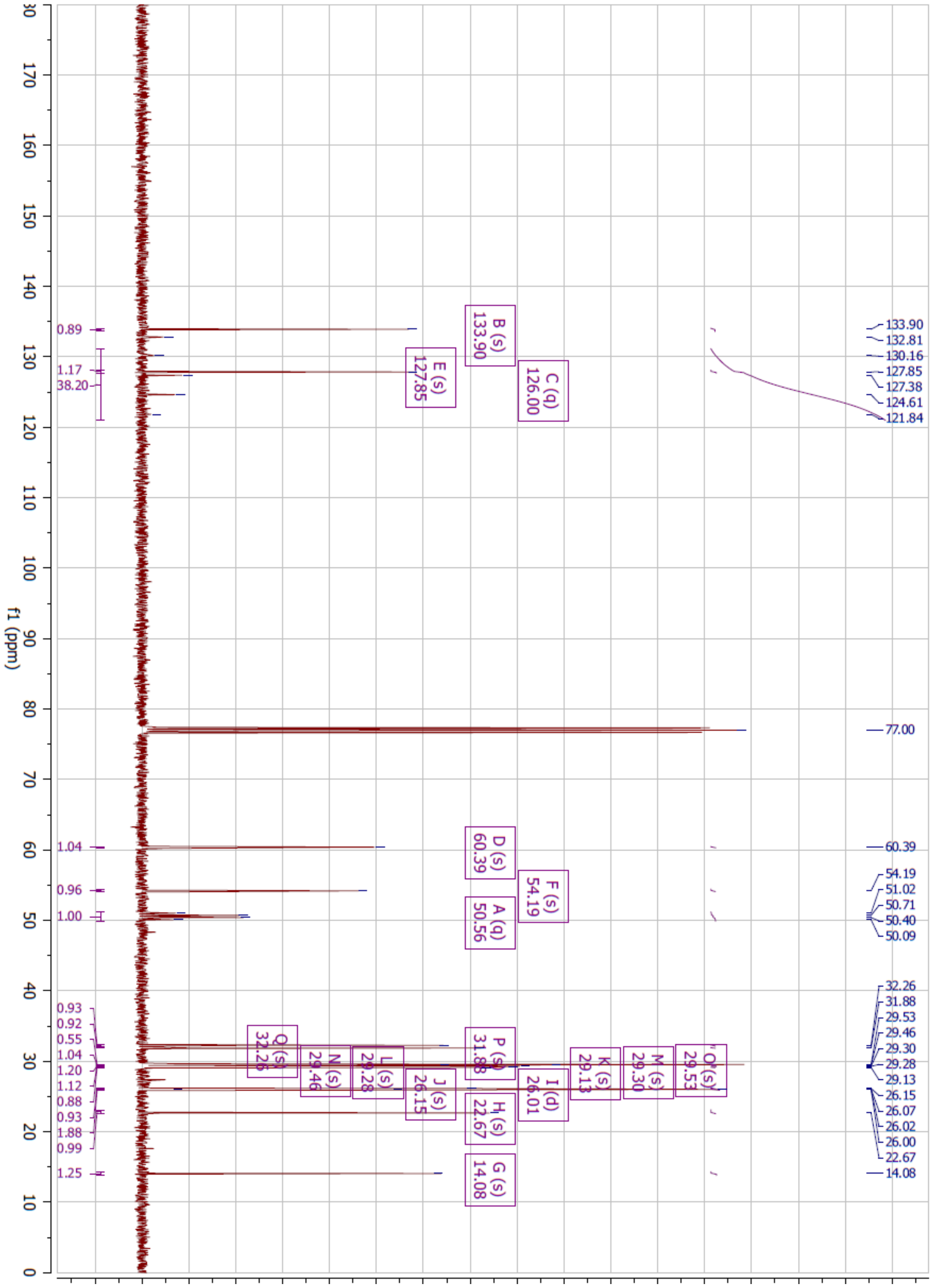

Supplementary Figure 103.

^{19}F-NMR (376 MHz, $CDCl_3$) *(E)*-*N*-(2,2,2-trifluoroethyl)-*N*-(undec-2-en-1-yl)cyclohexanamine *(major: 93%)* (**31**)

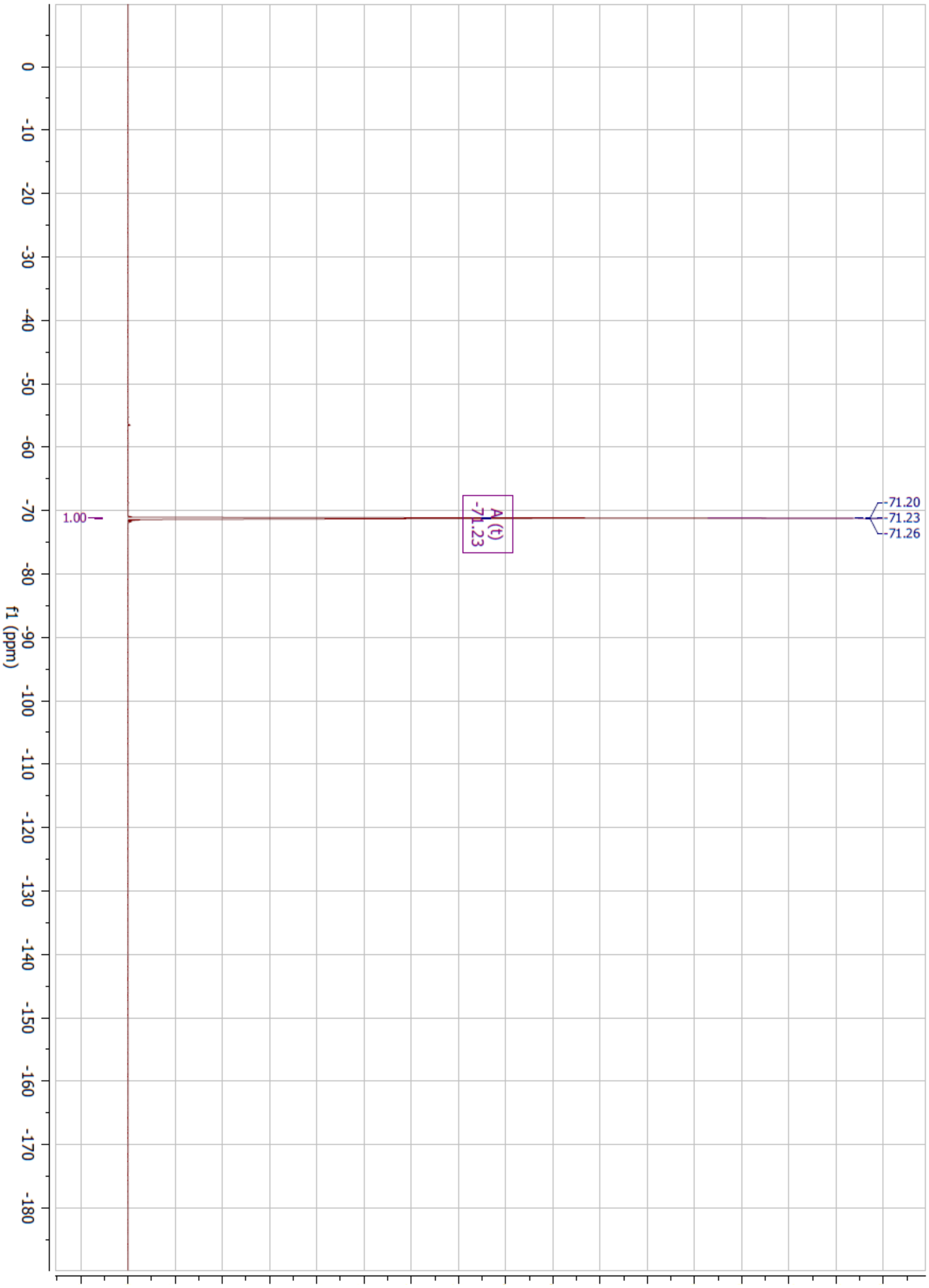

Supplementary Figure 104.

^{1}H-NMR (400 MHz, $CDCl_3$) (*S*)-*N*-(1-phenylethyl)-*N*-(2,2,2-trifluoroethyl)nonan-1-amine (**32**)

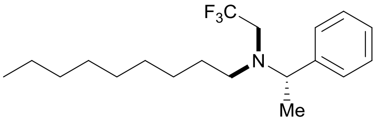

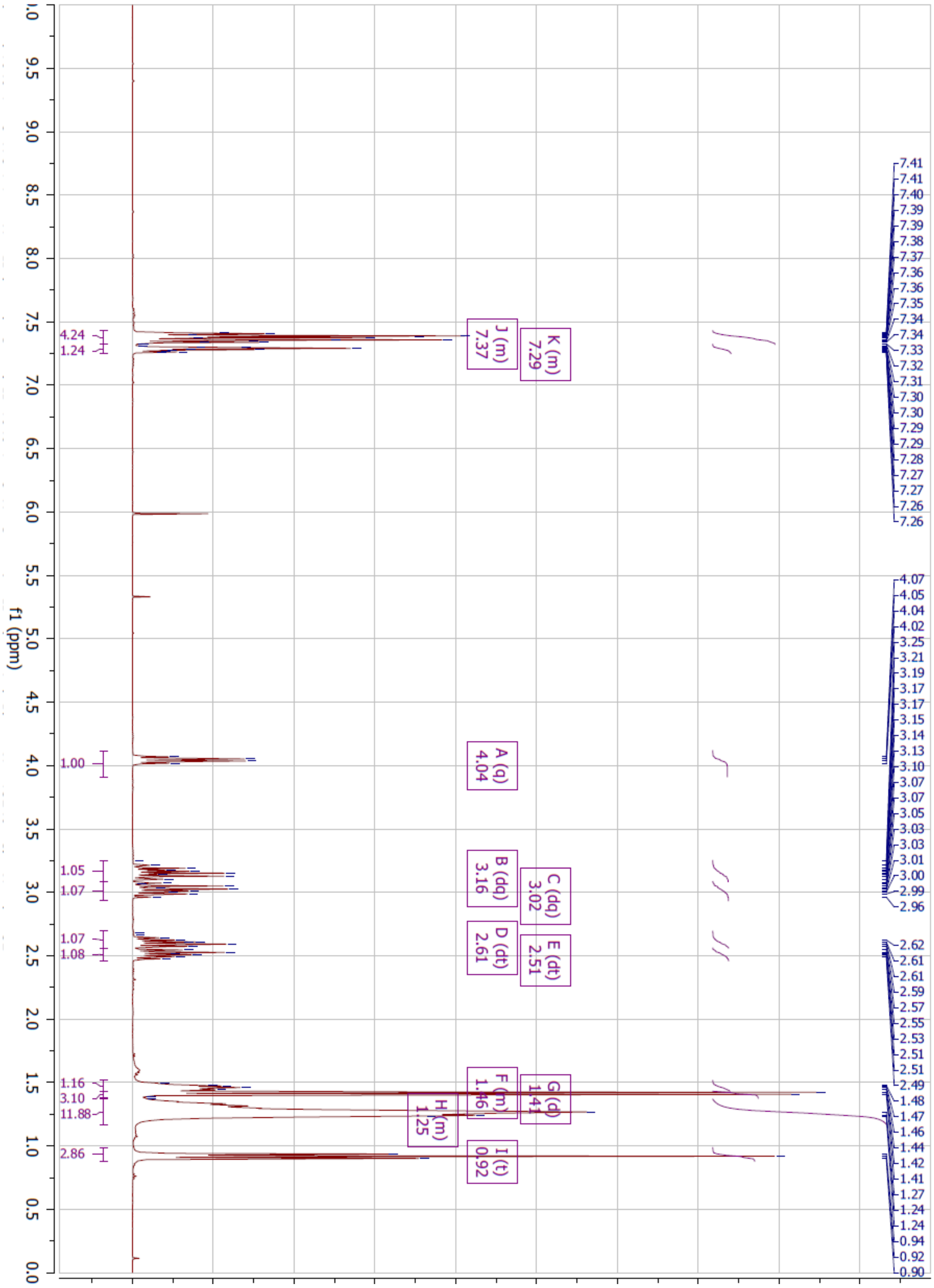

Supplementary Figure 105.

^{13}C-NMR (101 MHz, $CDCl_3$) (*S*)-*N*-(1-phenylethyl)-*N*-(2,2,2-trifluoroethyl)nonan-1-amine (**32**)

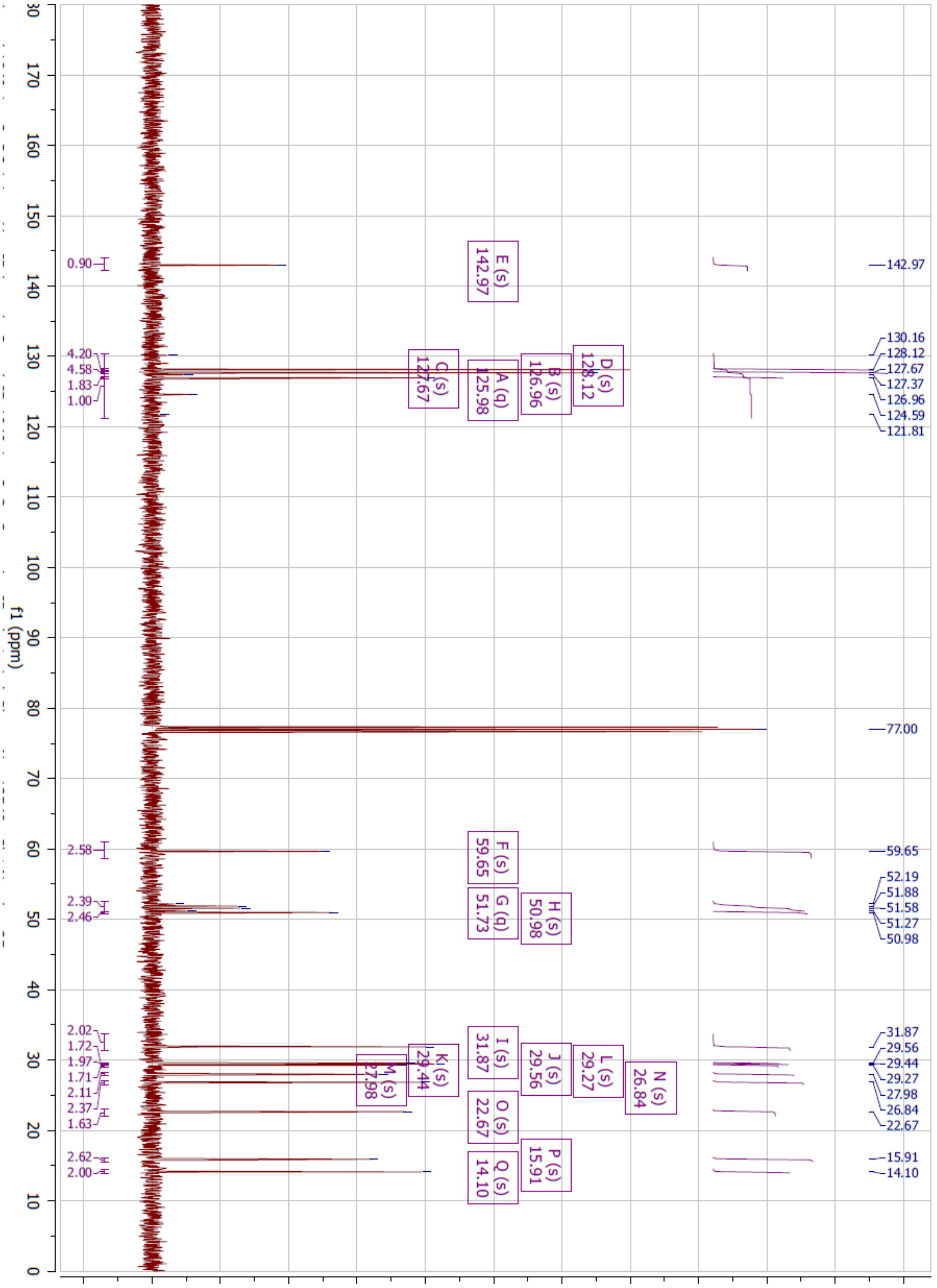

Supplementary Figure 106.

^{19}F-NMR (376 MHz, $CDCl_3$) (*S*)-*N*-(1-phenylethyl)-*N*-(2,2,2-trifluoroethyl)nonan-1-amine (**32**)

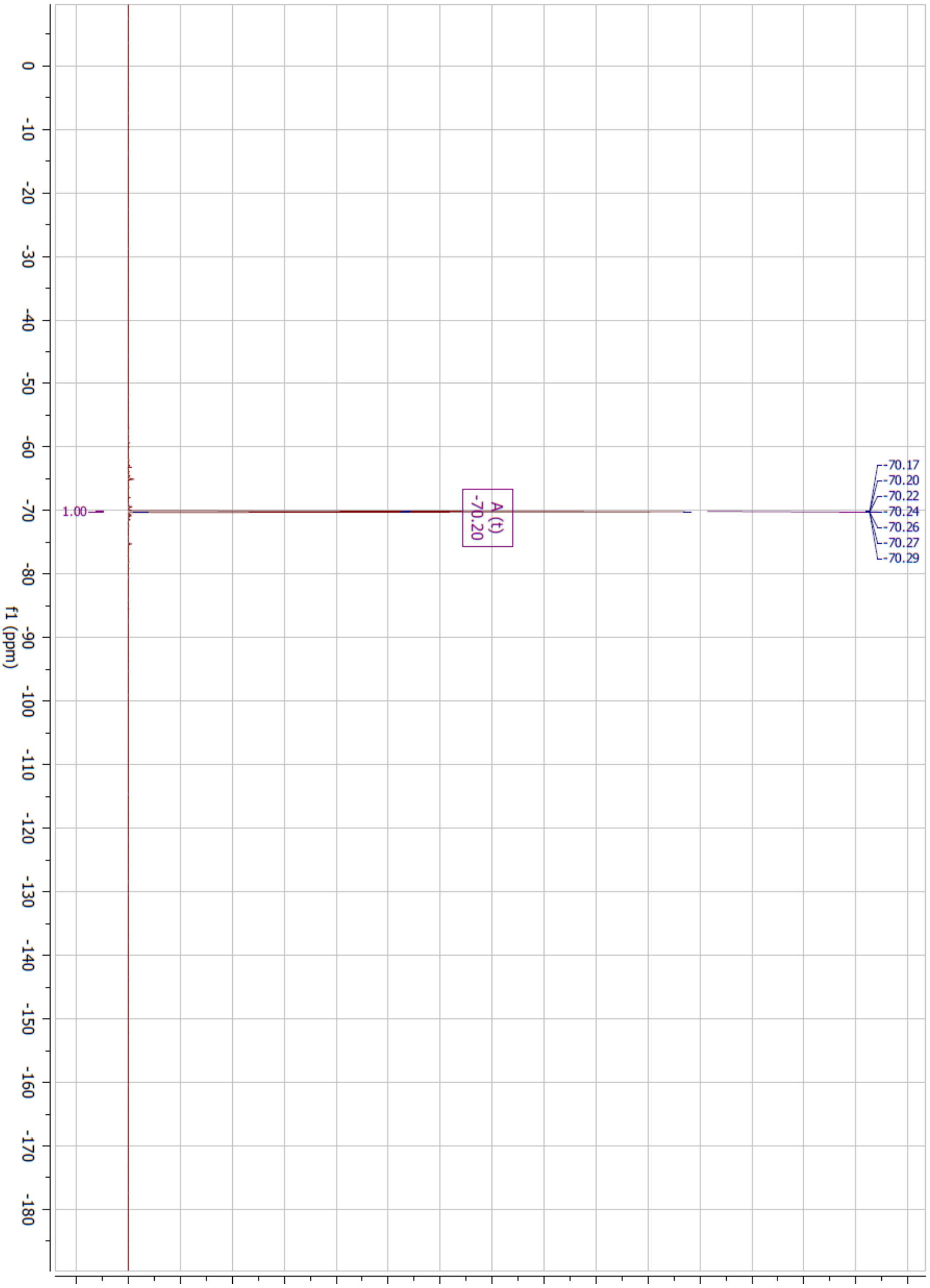

Supplementary Figure 107.
^{1}H-NMR (400 MHz, $CDCl_3$) *tert*-butyl (6-((5-bromo-2-methoxybenzyl)(2,2,2-trifluoroethyl)amino)hexyl)carbamate (**33**)

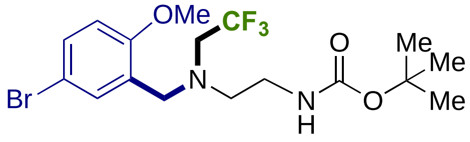

33: 43%

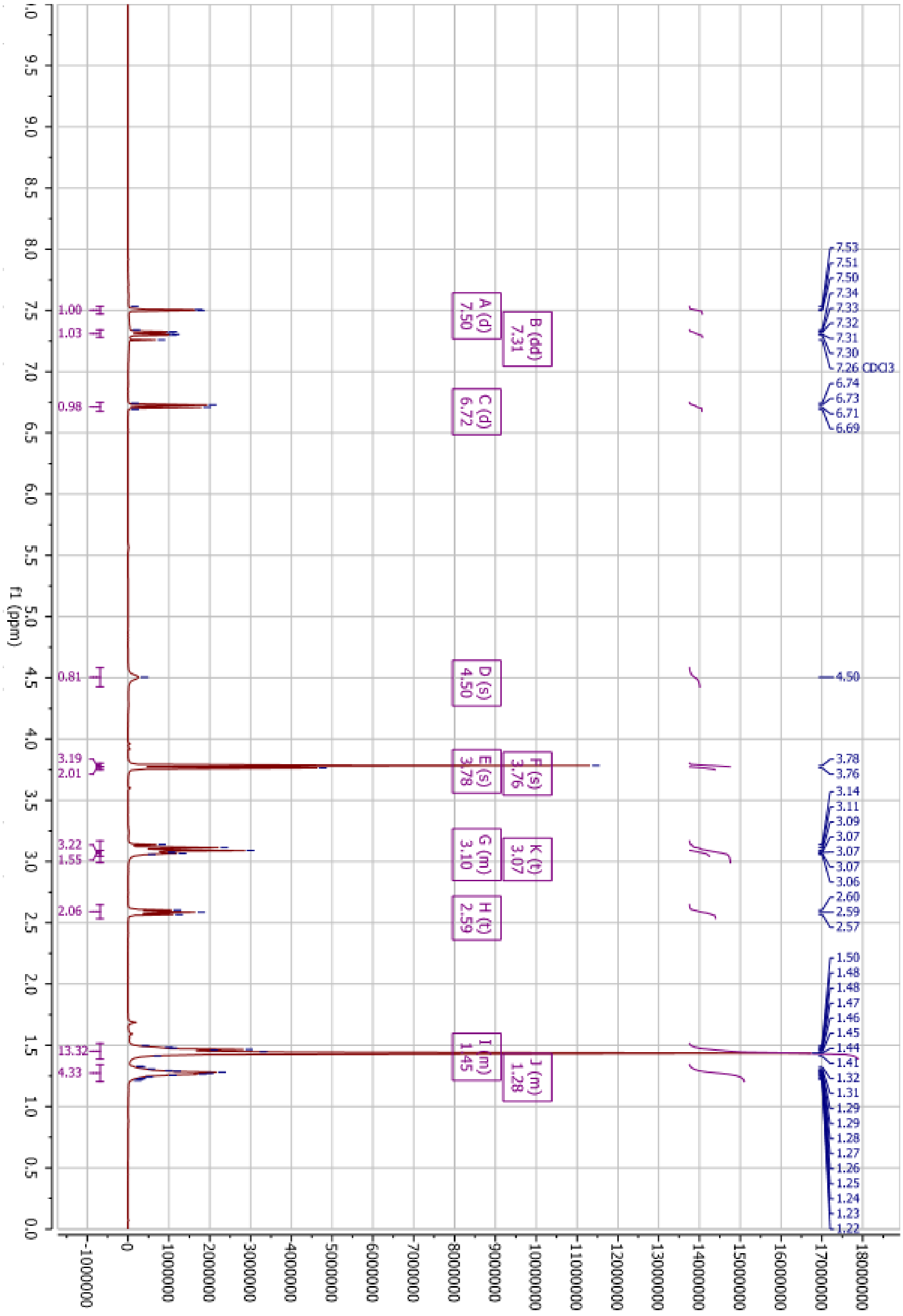

Supplementary Figure 108.
^{13}C-NMR (101 MHz, $CDCl_3$) *tert*-butyl (6-((5-bromo-2-methoxybenzyl)(2,2,2-trifluoroethyl)amino)hexyl)carbamate (**33**)

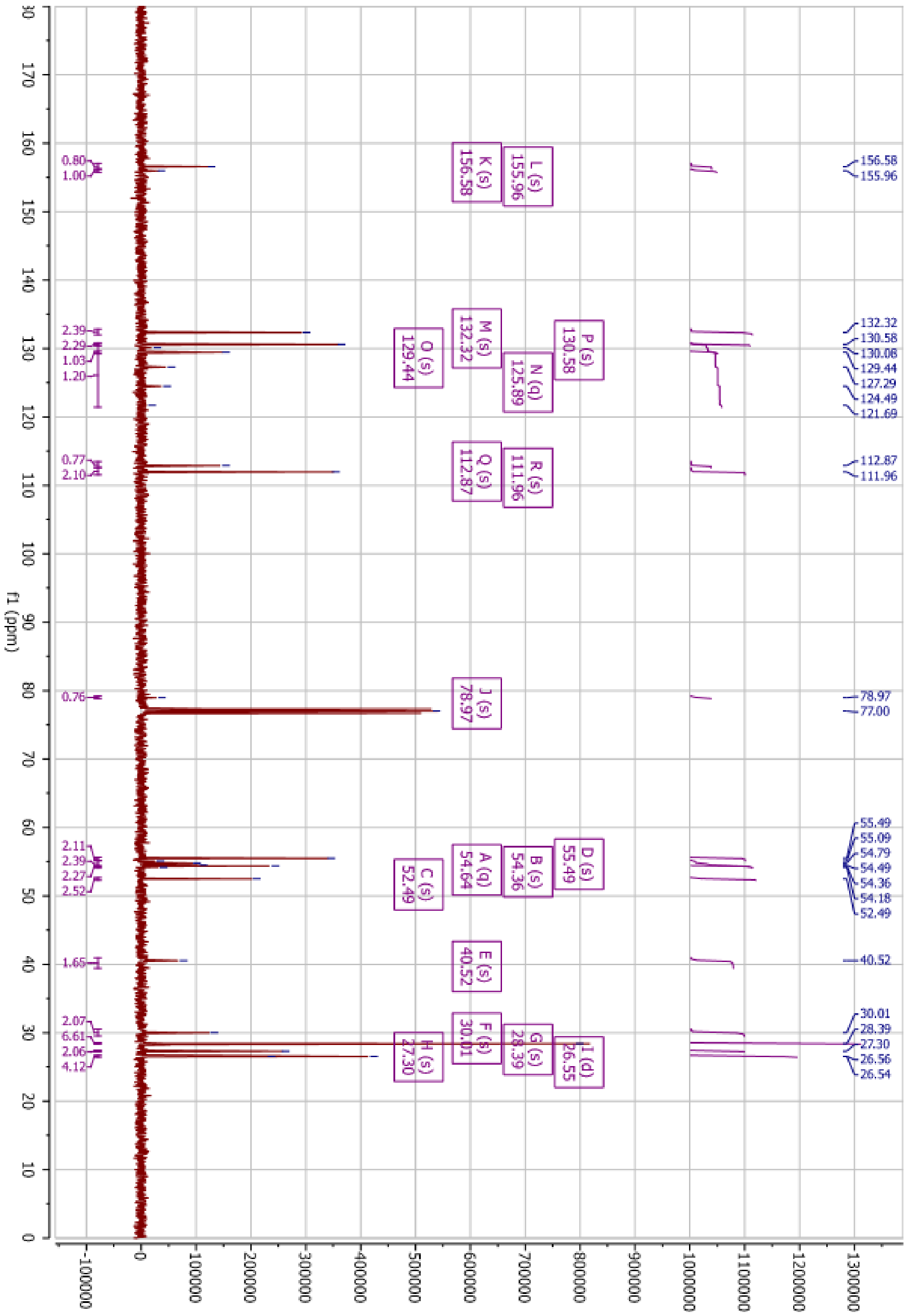

Supplementary Figure 109.
^{19}F-NMR (376 MHz, $CDCl_3$) *tert*-butyl (6-((5-bromo-2-methoxybenzyl)(2,2,2-trifluoroethyl)amino)hexyl)carbamate (**33**)

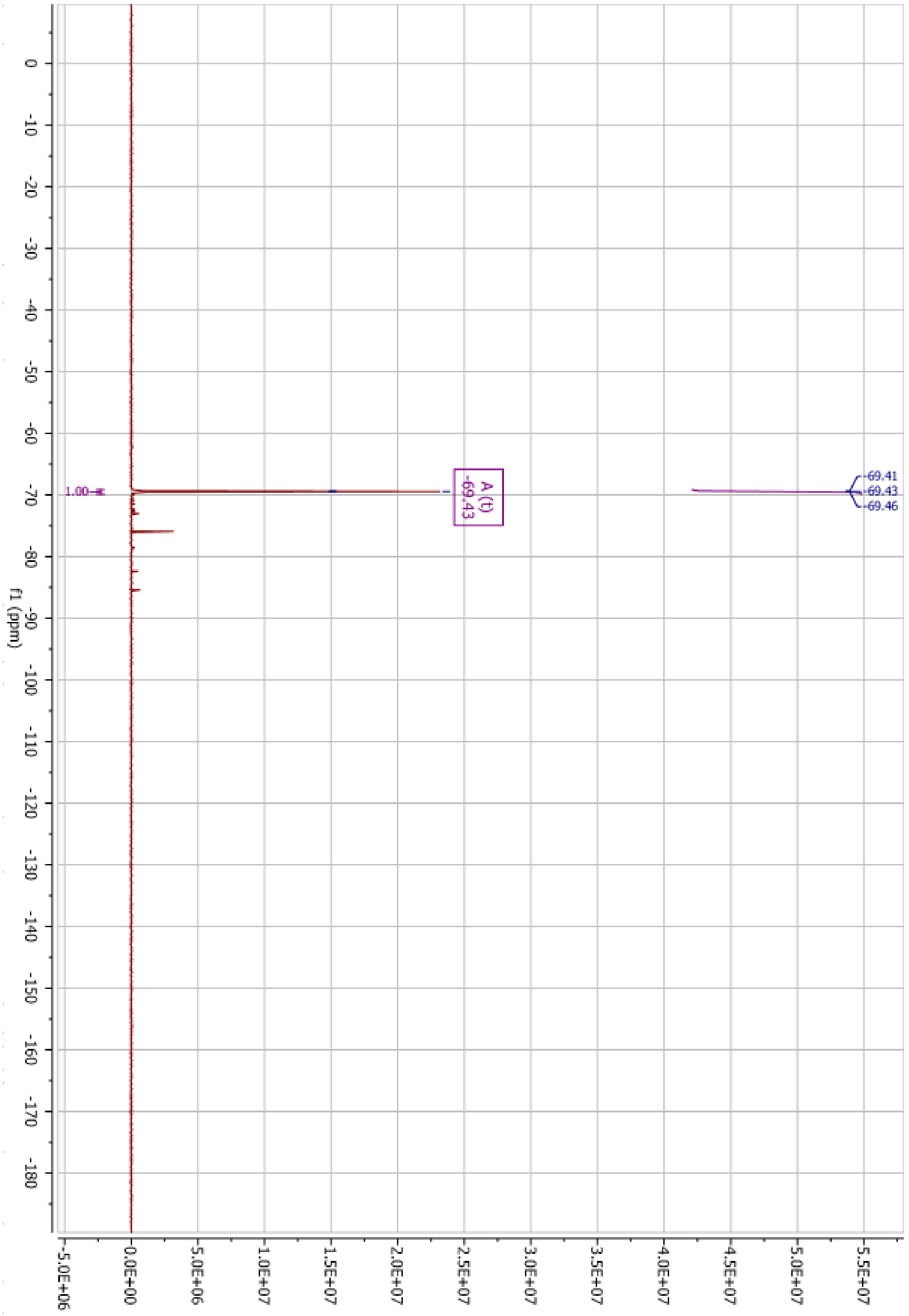

Supplementary Figure 110.

^{1}H-NMR (400 MHz, $CDCl_3$)

1-(3-((3,7-dimethyloct-6-en-1-yl)(2,2,2-trifluoroethyl)amino)propyl)pyrrolidin-2-one (**34**)

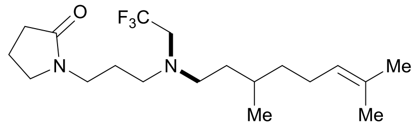

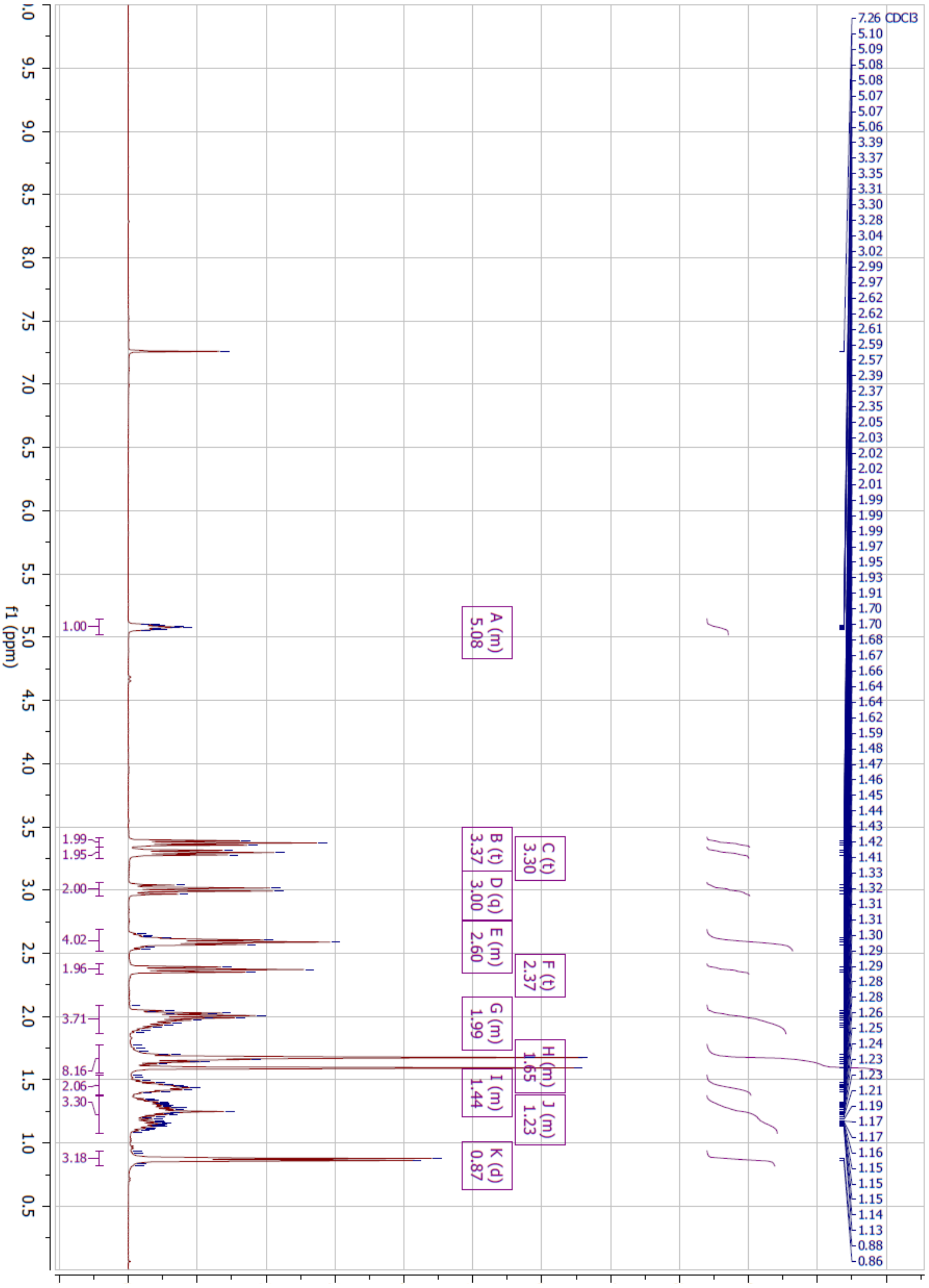

Supplementary Figure 111.

^{13}C-NMR (101 MHz, $CDCl_3$)

1-(3-((3,7-dimethyloct-6-en-1-yl)(2,2,2-trifluoroethyl)amino)propyl)pyrrolidin-2-one (**34**)

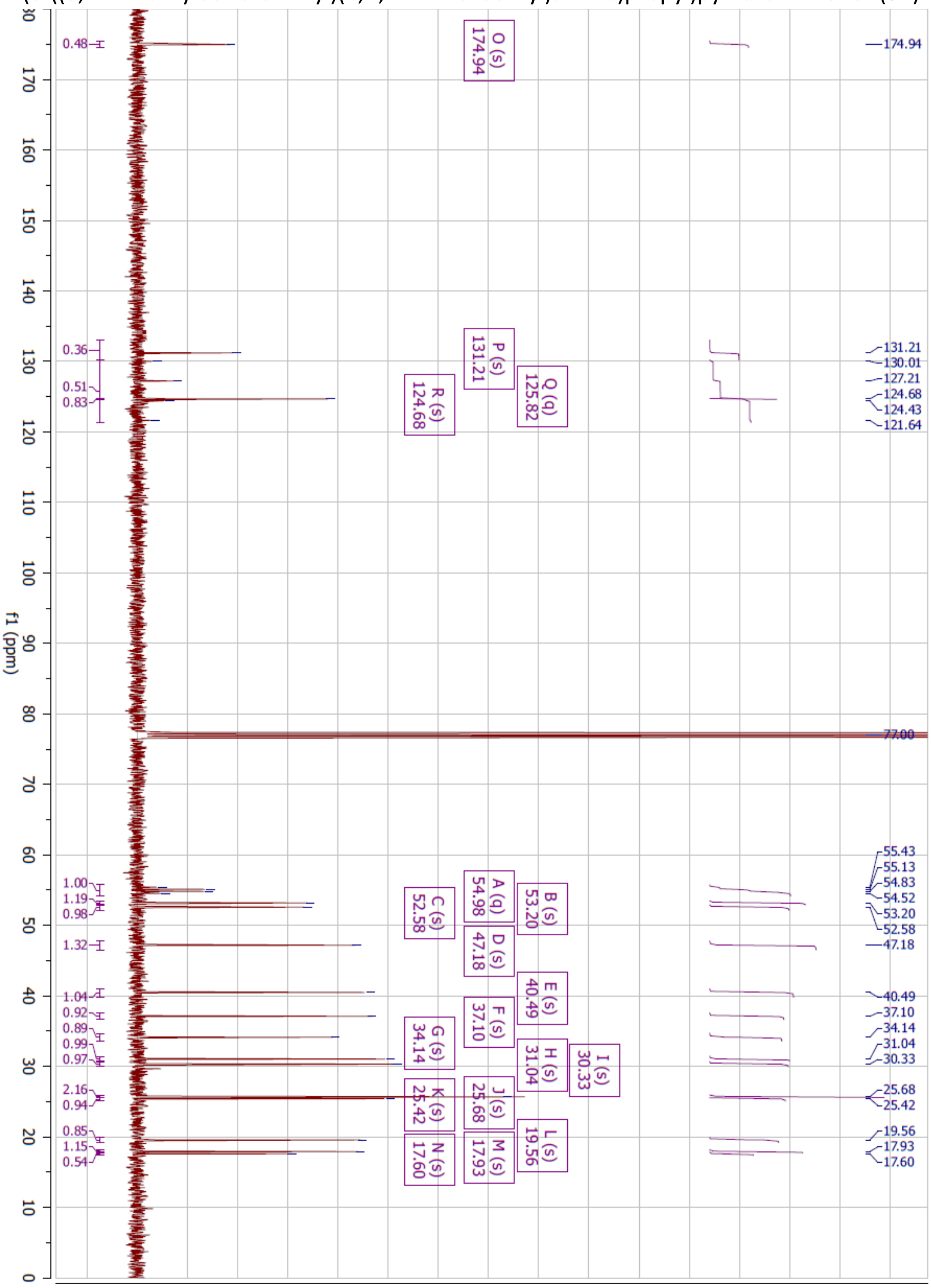

Supplementary Figure 112.

^{19}F-NMR (376 MHz, $CDCl_3$)

1-(3-((3,7-dimethyloct-6-en-1-yl)(2,2,2-trifluoroethyl)amino)propyl)pyrrolidin-2-one (**34**)

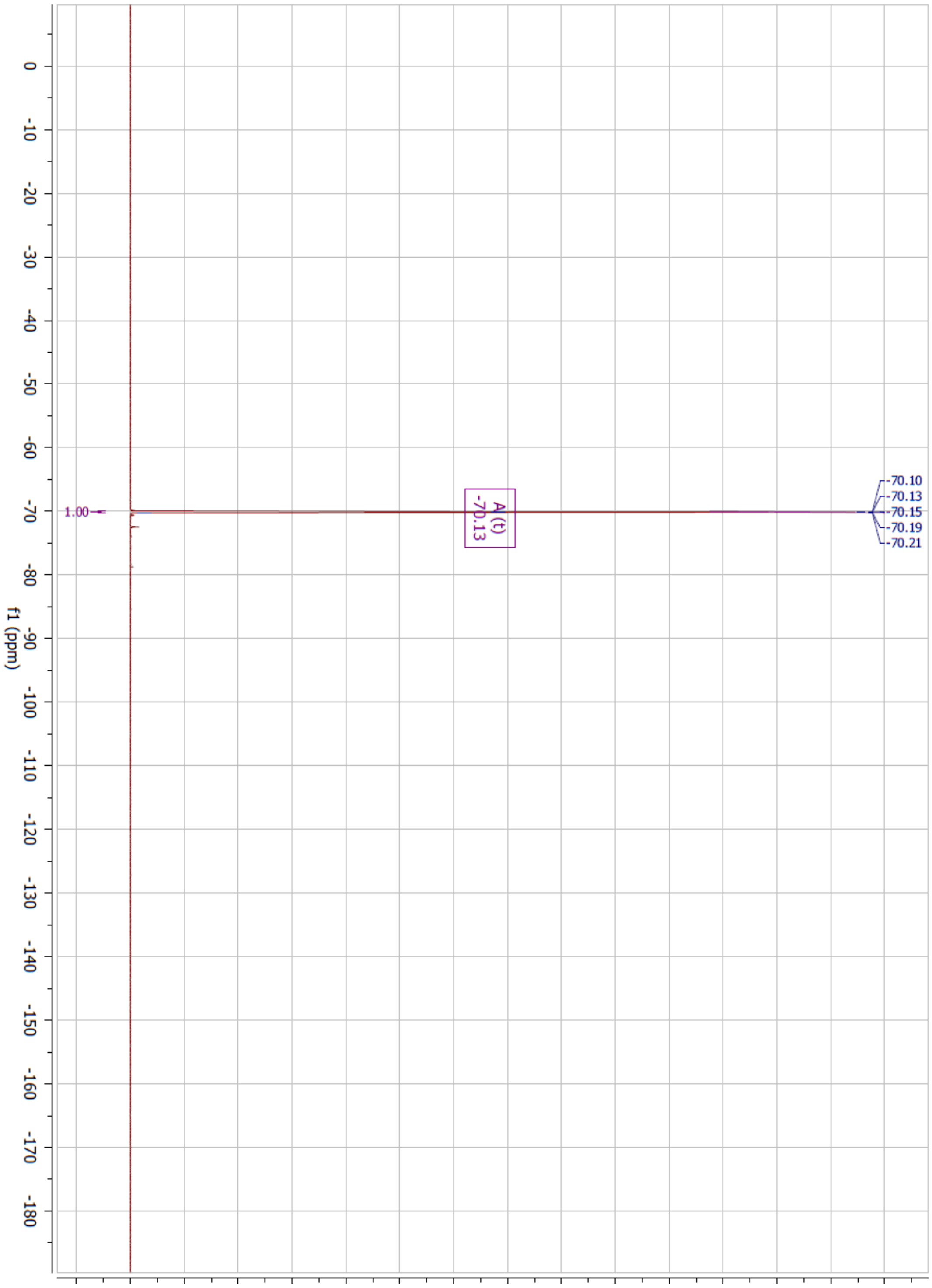

Supplementary Figure 113.
^{1}H-NMR (400 MHz, $CDCl_3$) *N*-((6-bromobenzo[*d*][1,3]dioxol-5-yl)methyl)-*N*-(2,2,2-trifluoroethyl)cyclohexanamine (**35**)

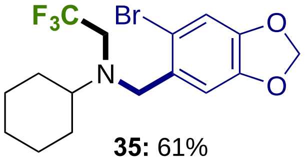

35: 61%

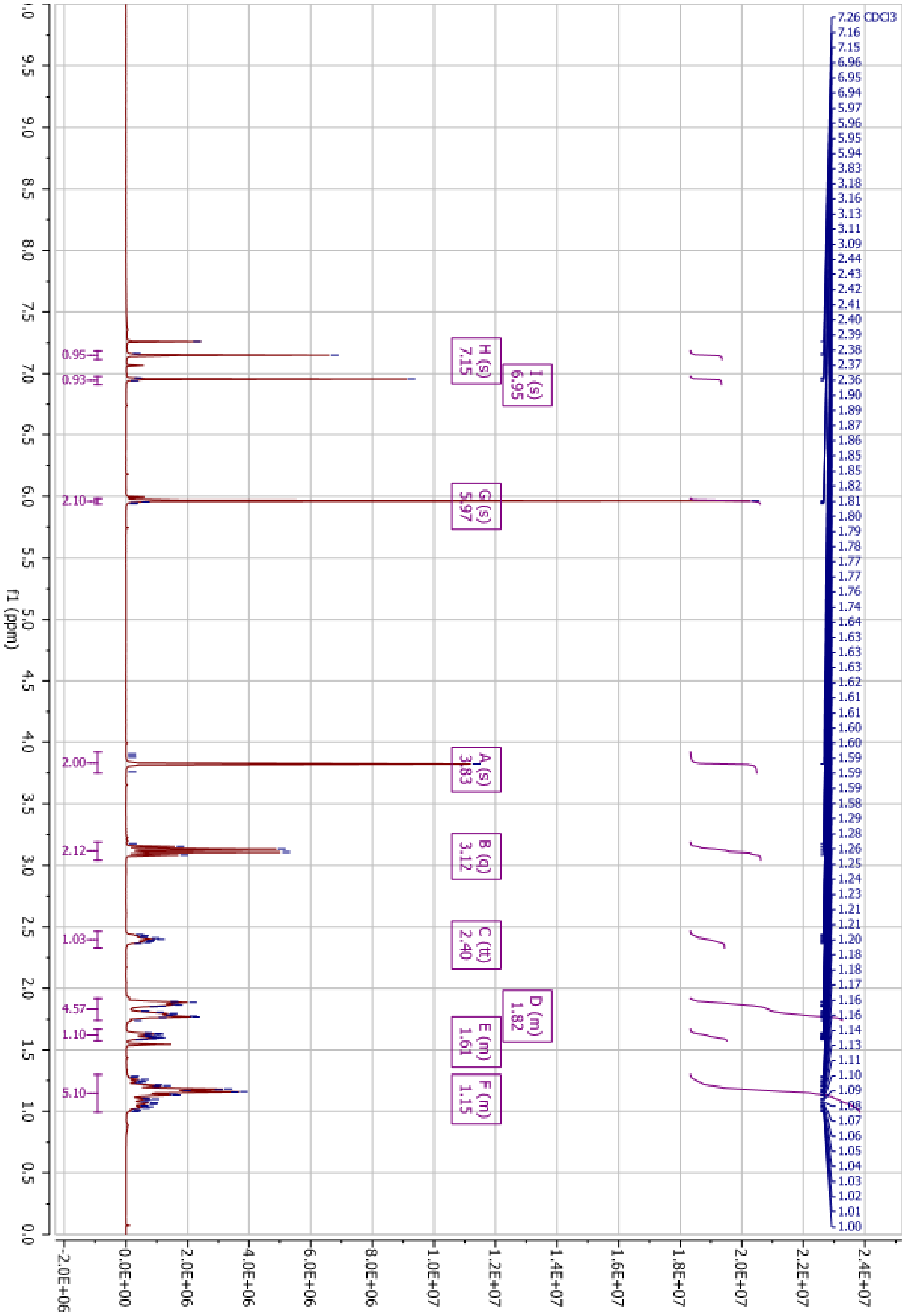

Supplementary Figure 114.

^{13}C-NMR (101 MHz, $CDCl_3$) *N*-((6-bromobenzo[*d*][1,3]dioxol-5-yl)methyl)-*N*-(2,2,2-trifluoroethyl)cyclohexanamine (**35**)

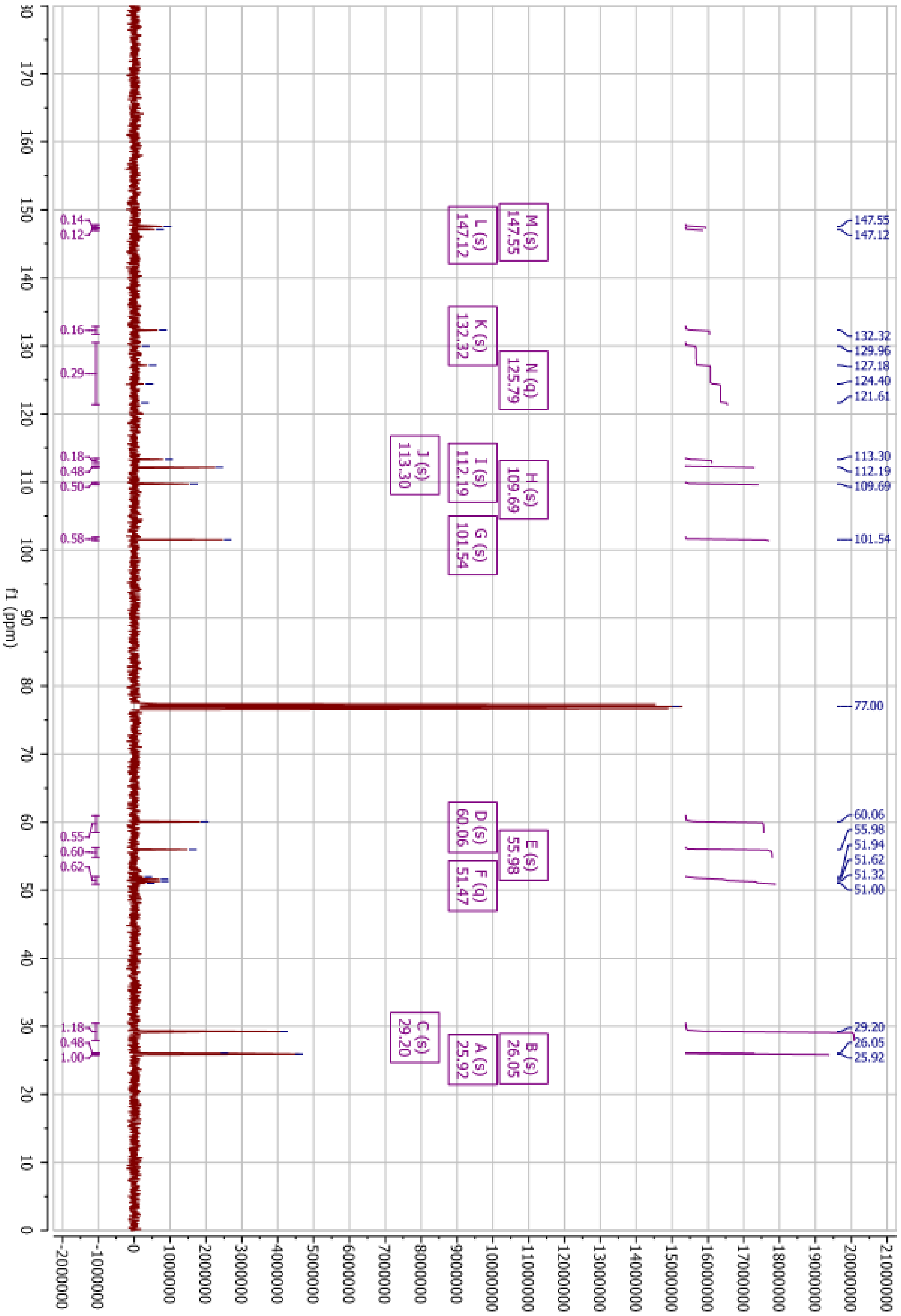

Supplementary Figure 115.
^{19}**F-NMR (376 MHz, CDCl$_3$)** *N*-((6-bromobenzo[*d*][1,3]dioxol-5-yl)methyl)-*N*-(2,2,2-trifluoroethyl)cyclohexanamine (**35**)

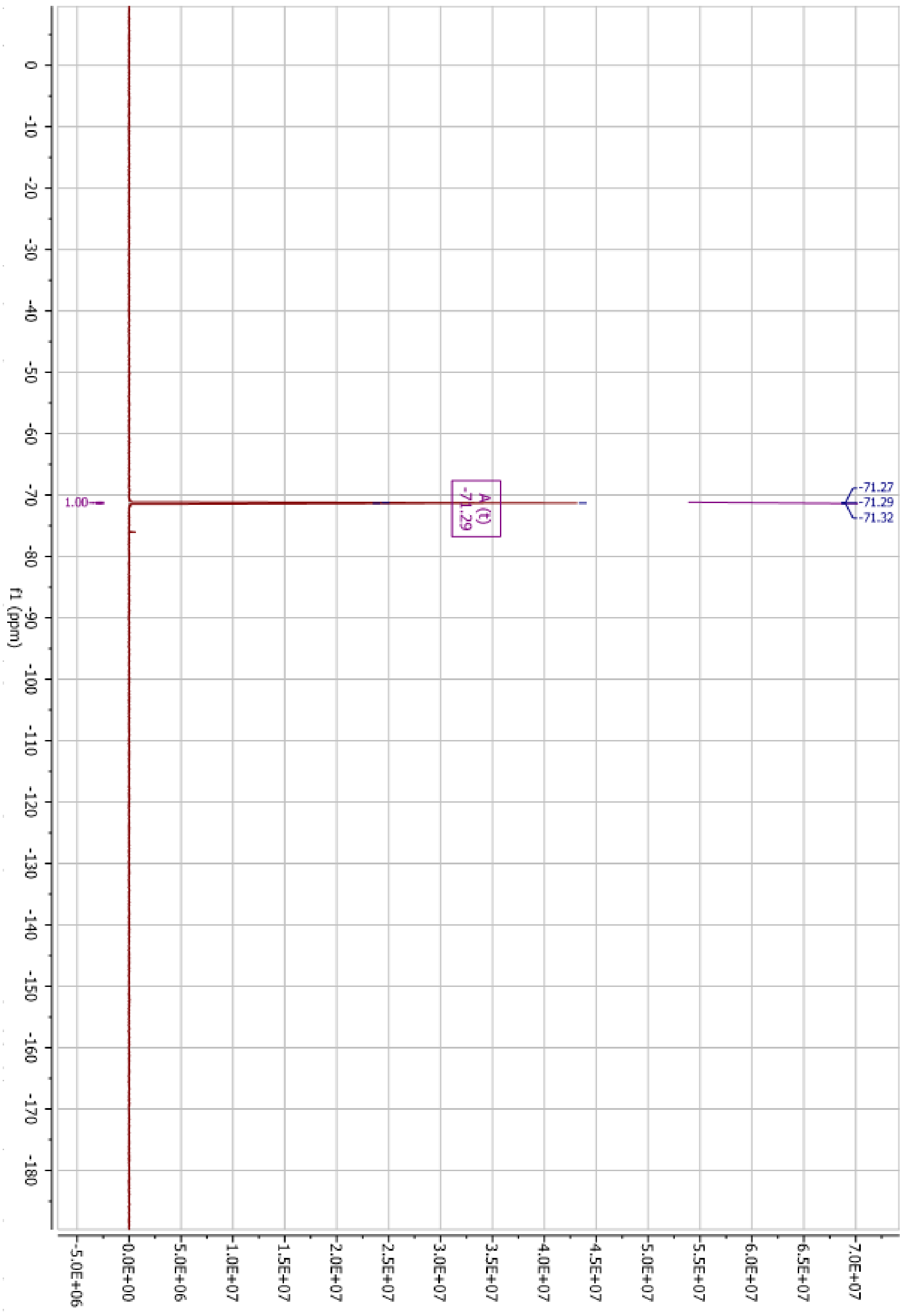

Supplementary Figure 116.

^{1}H-NMR (400 MHz, $CDCl_3$) *N*-benzyl-2,2,2-trifluoro-*N*-(1-phenylethyl)ethan-1-amine (**36**)

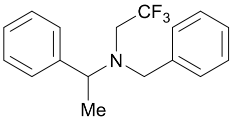

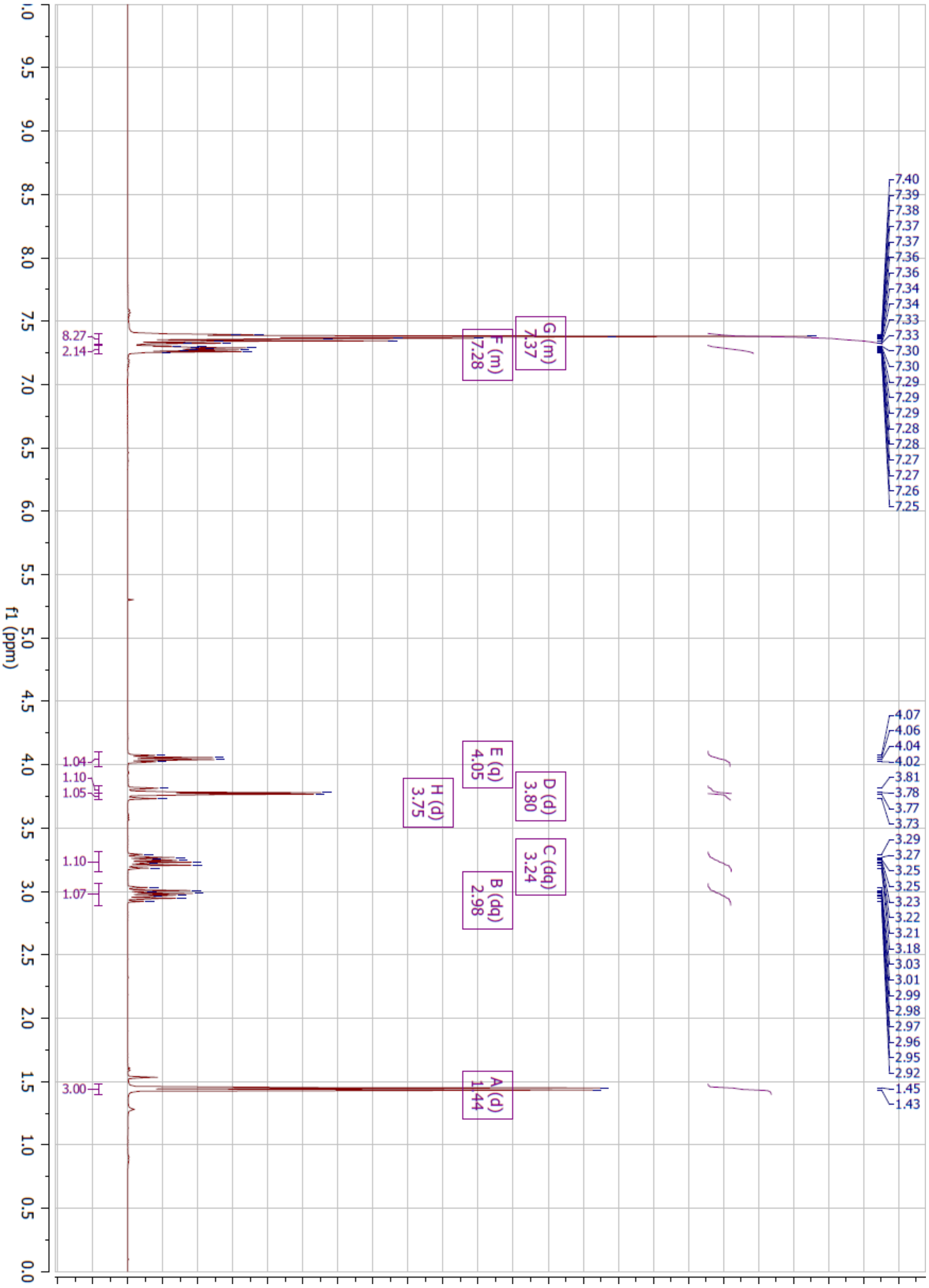

Supplementary Figure 117.

^{13}C-NMR (101 MHz, $CDCl_3$) *N*-benzyl-2,2,2-trifluoro-*N*-(1-phenylethyl)ethan-1-amine (**36**)

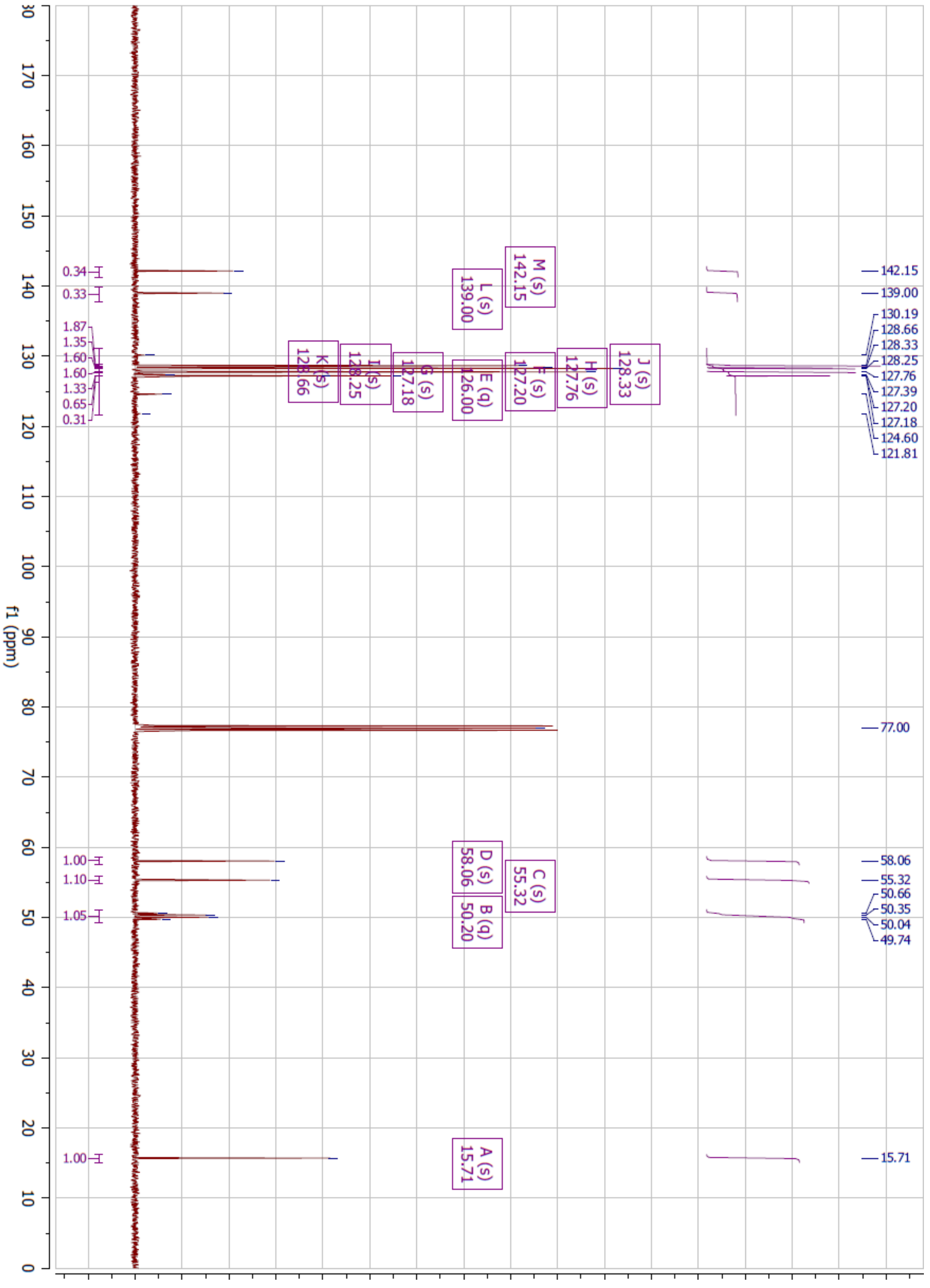

Supplementary Figure 118.

^{19}F-NMR (376 MHz, $CDCl_3$) *N*-benzyl-2,2,2-trifluoro-*N*-(1-phenylethyl)ethan-1-amine (**36**)

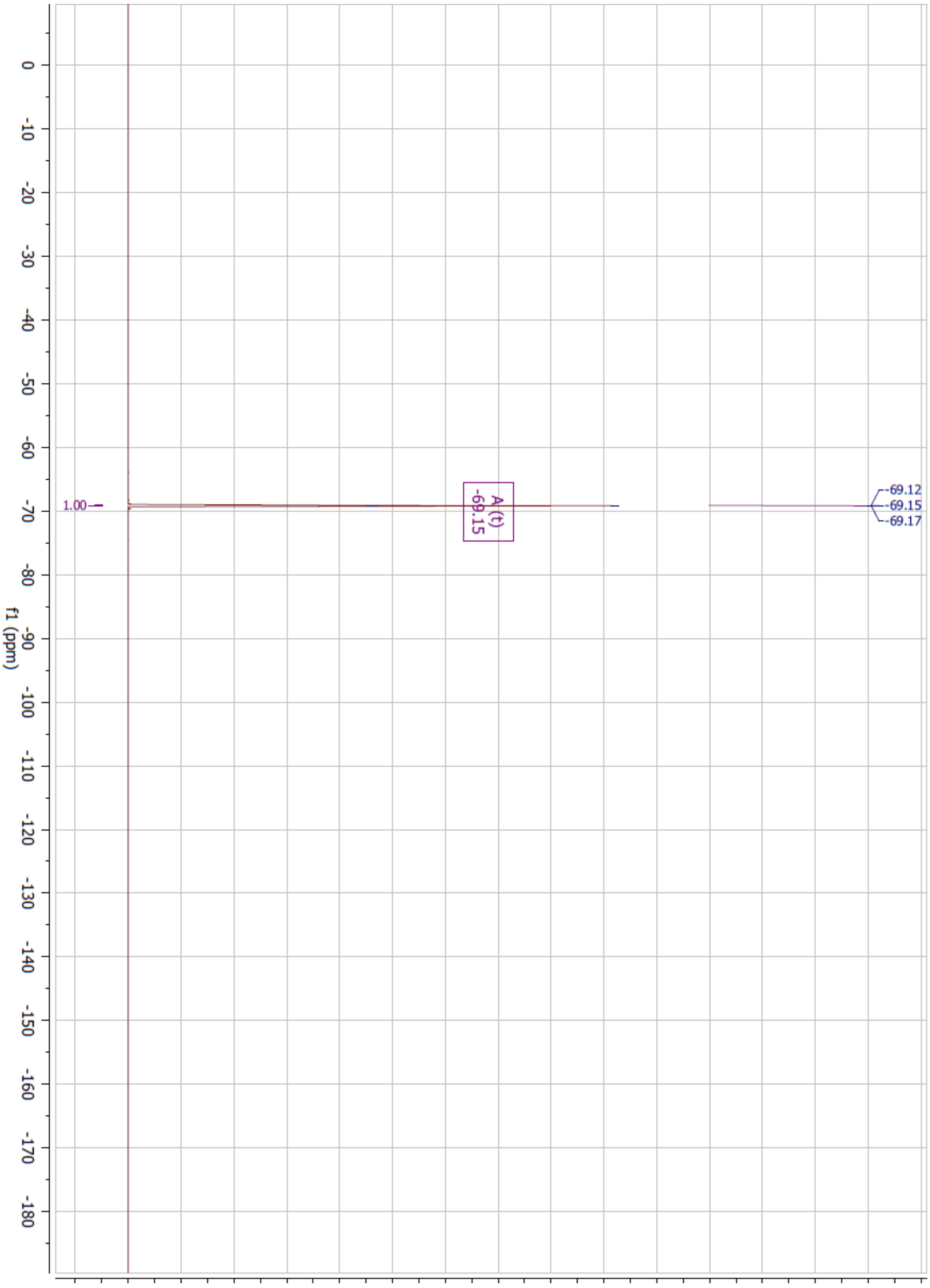

Supplementary Figure 119.

[1]**H-NMR (400 MHz, $CDCl_3$)** *N*-(3-phenylpropyl)-*N*-(2,2,2-trifluoroethyl)-2,3-dihydro-1H-inden-1-amine (**37**)

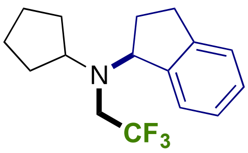

37: 20%

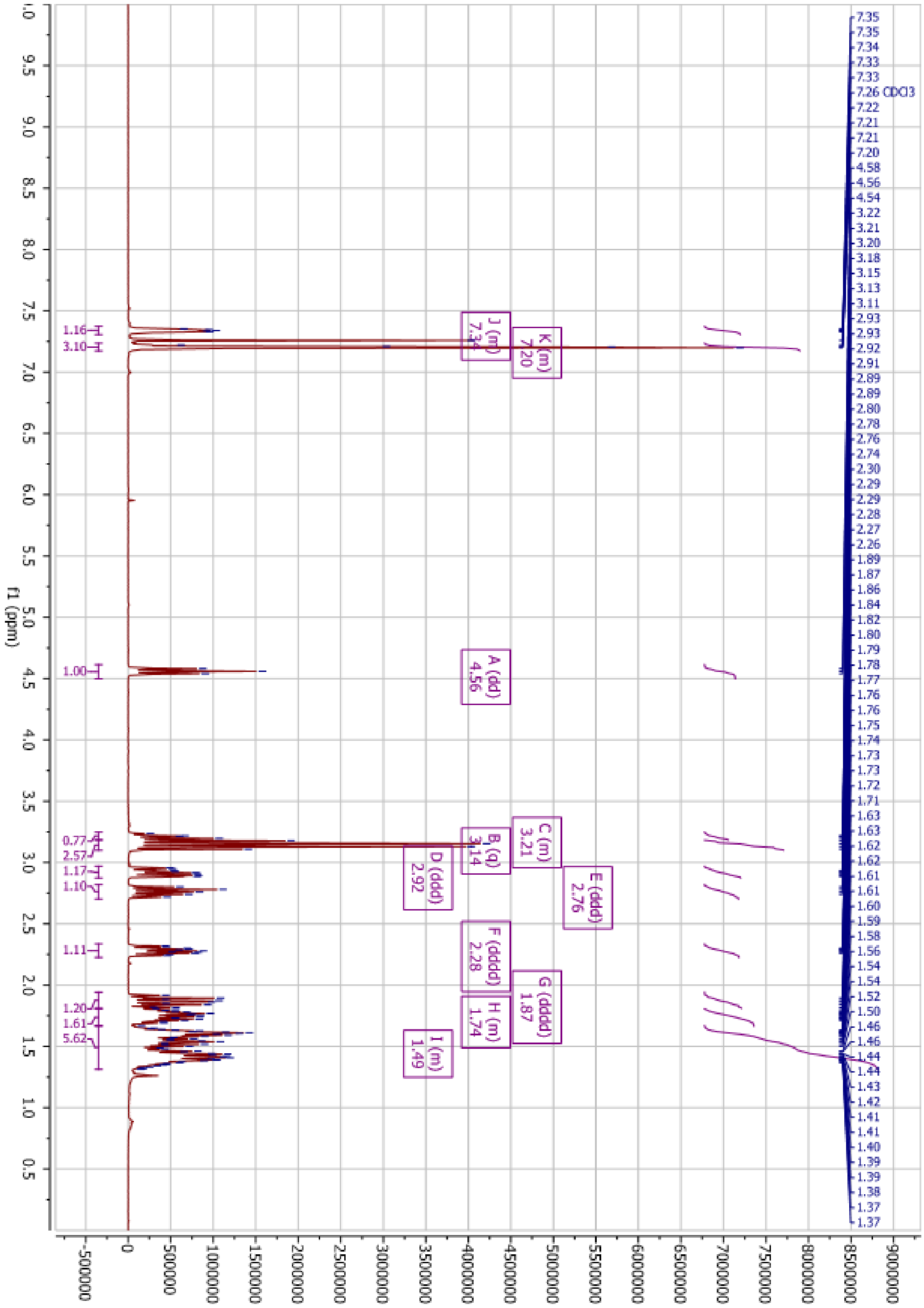

Supplementary Figure 120.
^{13}**C-NMR (101 MHz, $CDCl_3$)** *N*-(3-phenylpropyl)-*N*-(2,2,2-trifluoroethyl)-2,3-dihydro-1H-inden-1-amine (**37**)

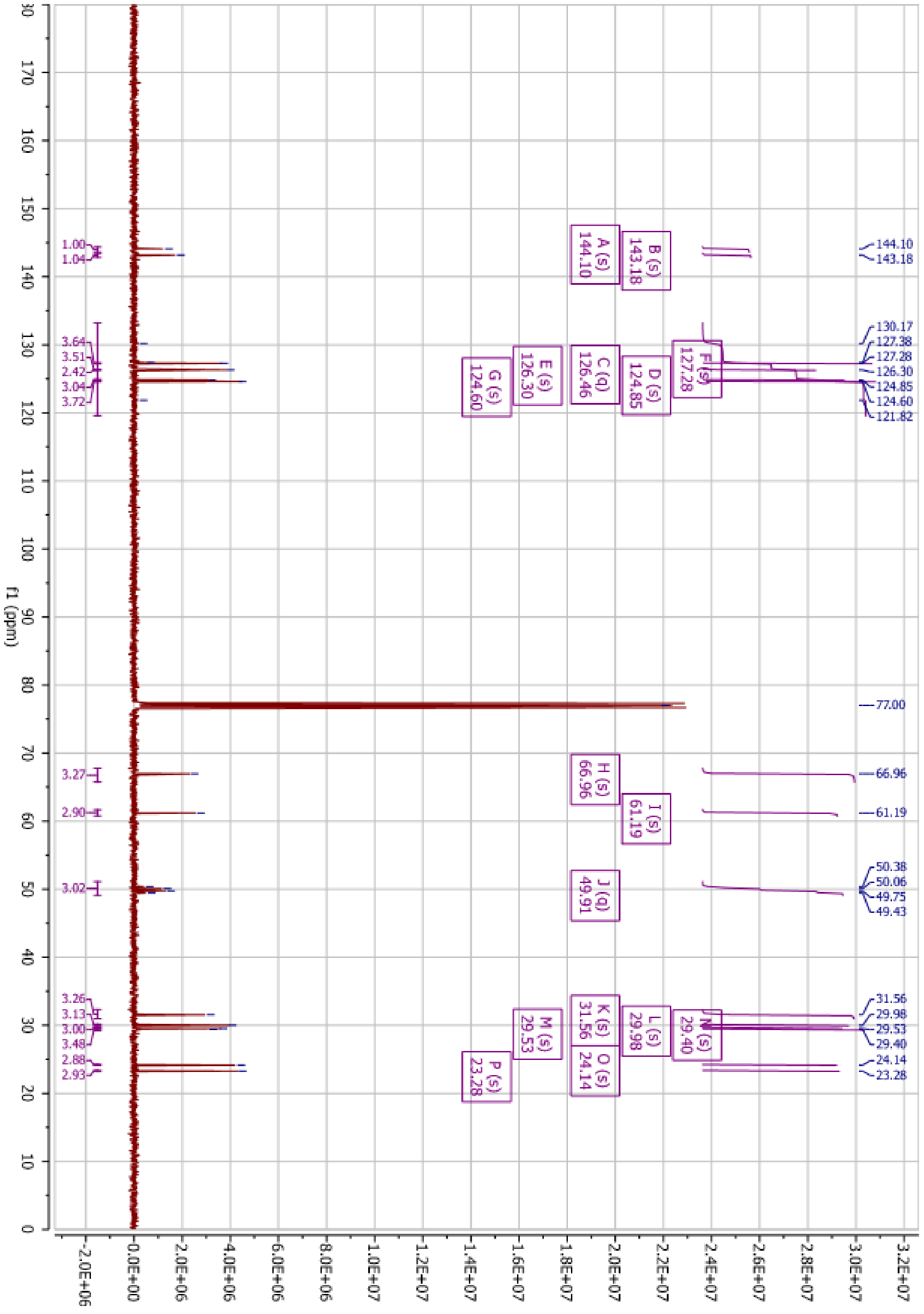

Supplementary Figure 121.
^{19}F-NMR (376 MHz, $CDCl_3$) *N*-(3-phenylpropyl)-*N*-(2,2,2-trifluoroethyl)-2,3-dihydro-1H-inden-1-amine (**37**)

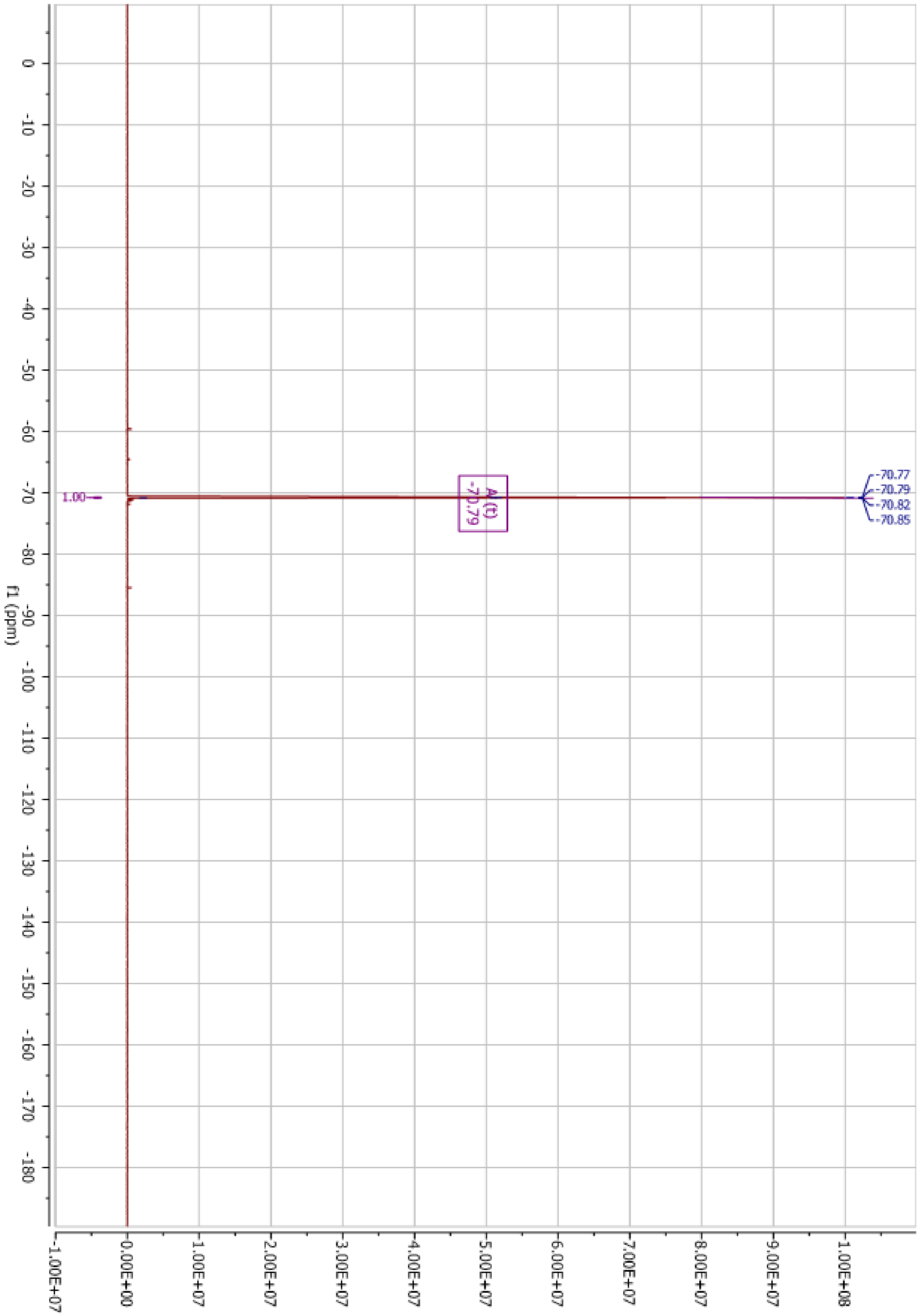

Supplementary Figure 122.
[1]**H-NMR (400 MHz, $CDCl_3$)** *N*-(3-phenylpropyl)-*N*-(2,2,2-trifluoroethyl)heptan-4-amine (**38**)

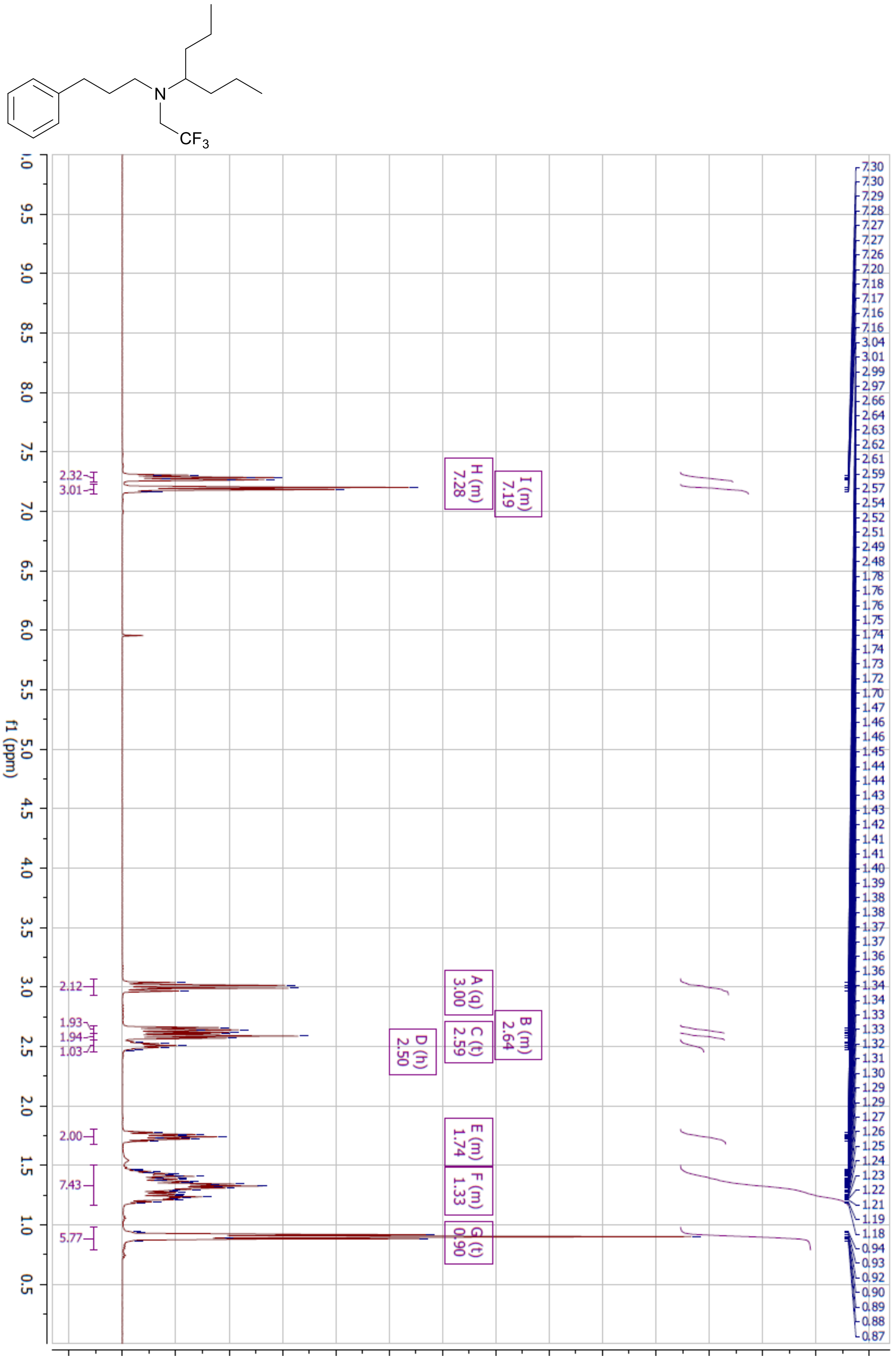

Supplementary Figure 123.

^{13}C-NMR (101 MHz, $CDCl_3$) *N*-(3-phenylpropyl)-*N*-(2,2,2-trifluoroethyl)heptan-4-amine (**38**)

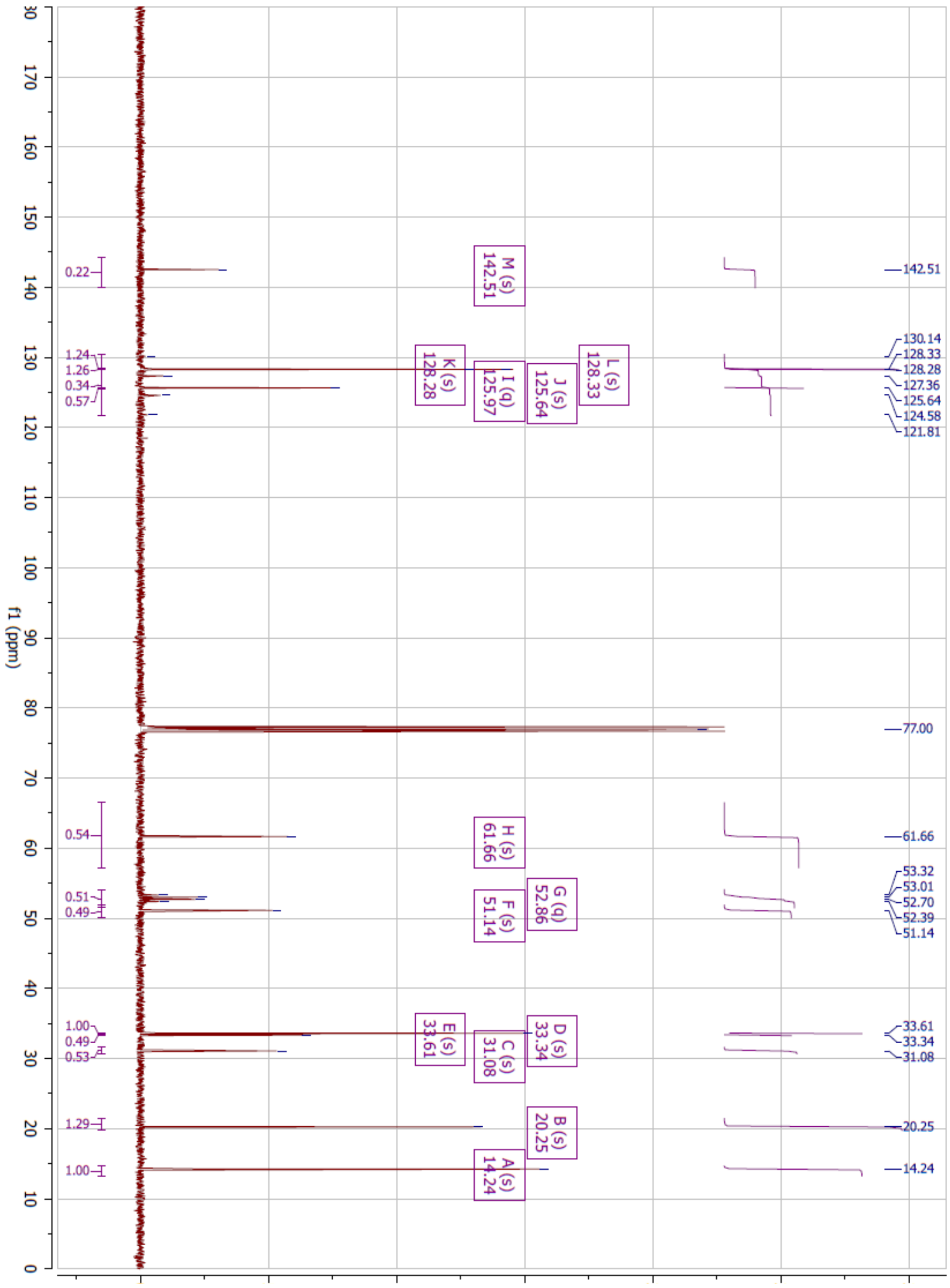

Supplementary Figure 124.
^{19}F-NMR (376 MHz, $CDCl_3$) *N*-(3-phenylpropyl)-*N*-(2,2,2-trifluoroethyl)heptan-4-amine (**38**)

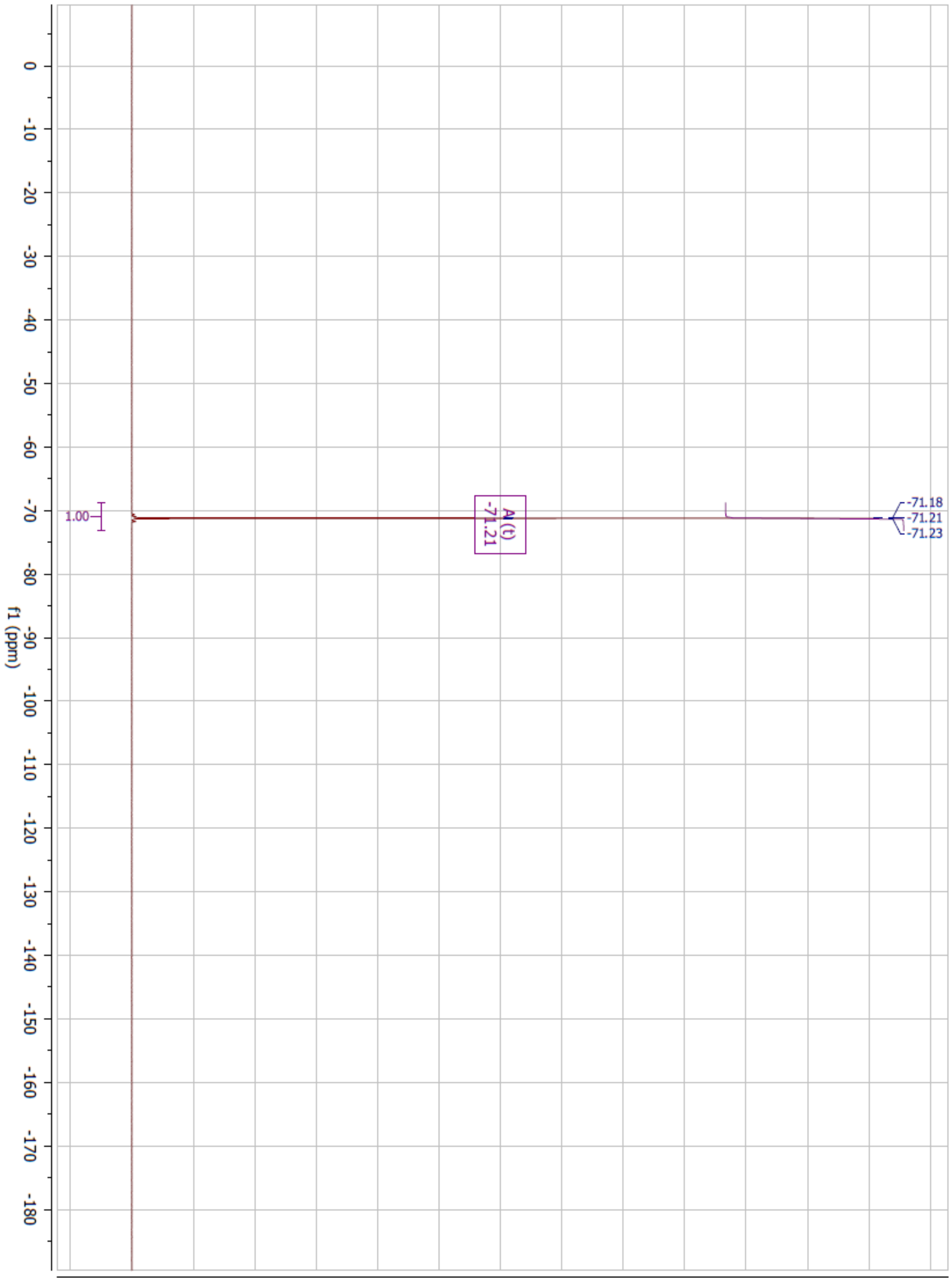

Supplementary Figure 125.

^{1}H-NMR (400 MHz, $CDCl_3$) ethyl *N*-(3-chloropropyl)-*N*-(2,2,2-trifluoroethyl)alaninate (**39**)

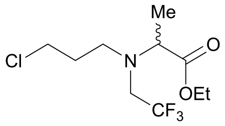

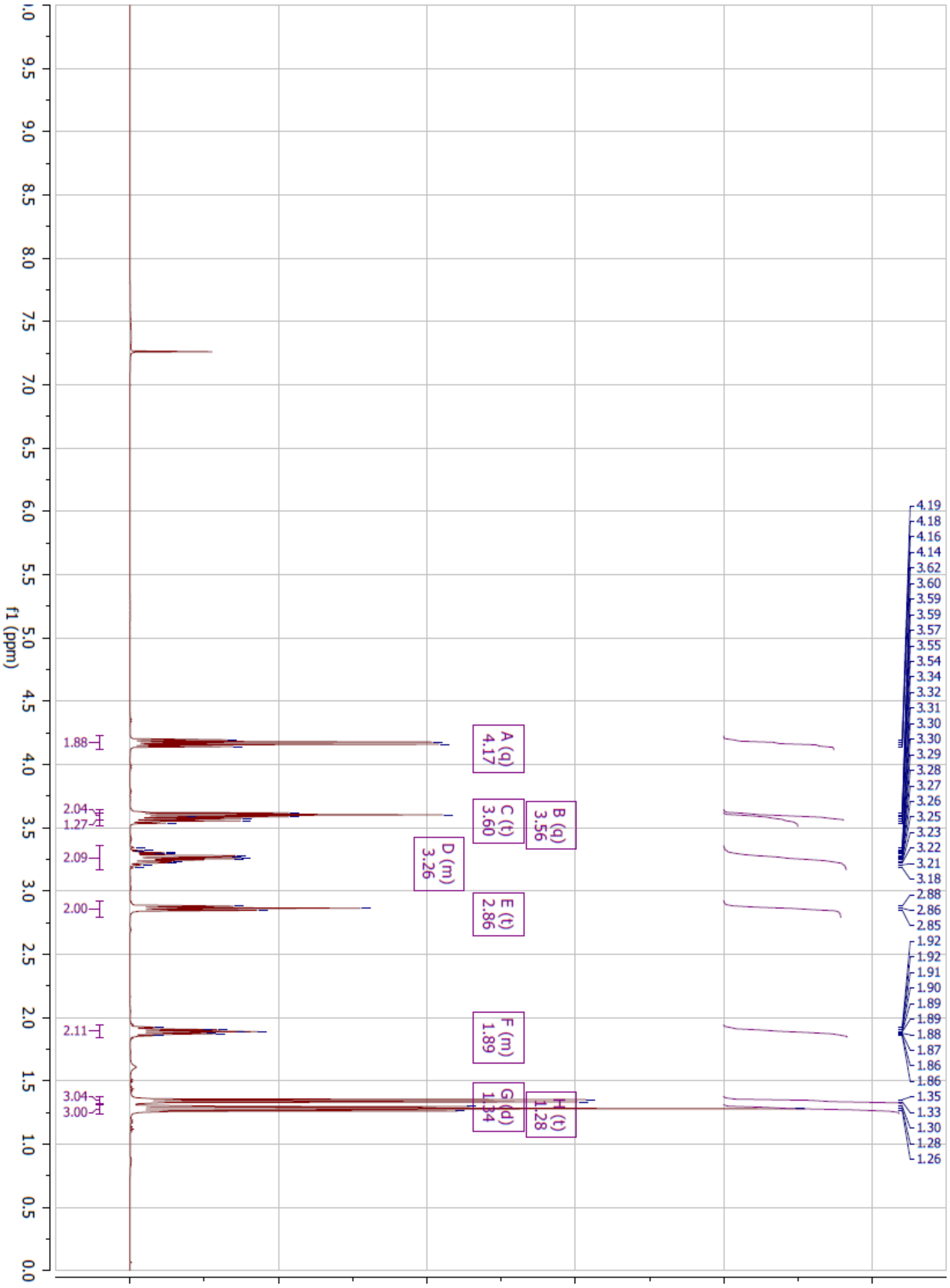

Supplementary Figure 126.

^{13}C-NMR (101 MHz, $CDCl_3$) ethyl *N*-(3-chloropropyl)-*N*-(2,2,2-trifluoroethyl)alaninate (**39**)

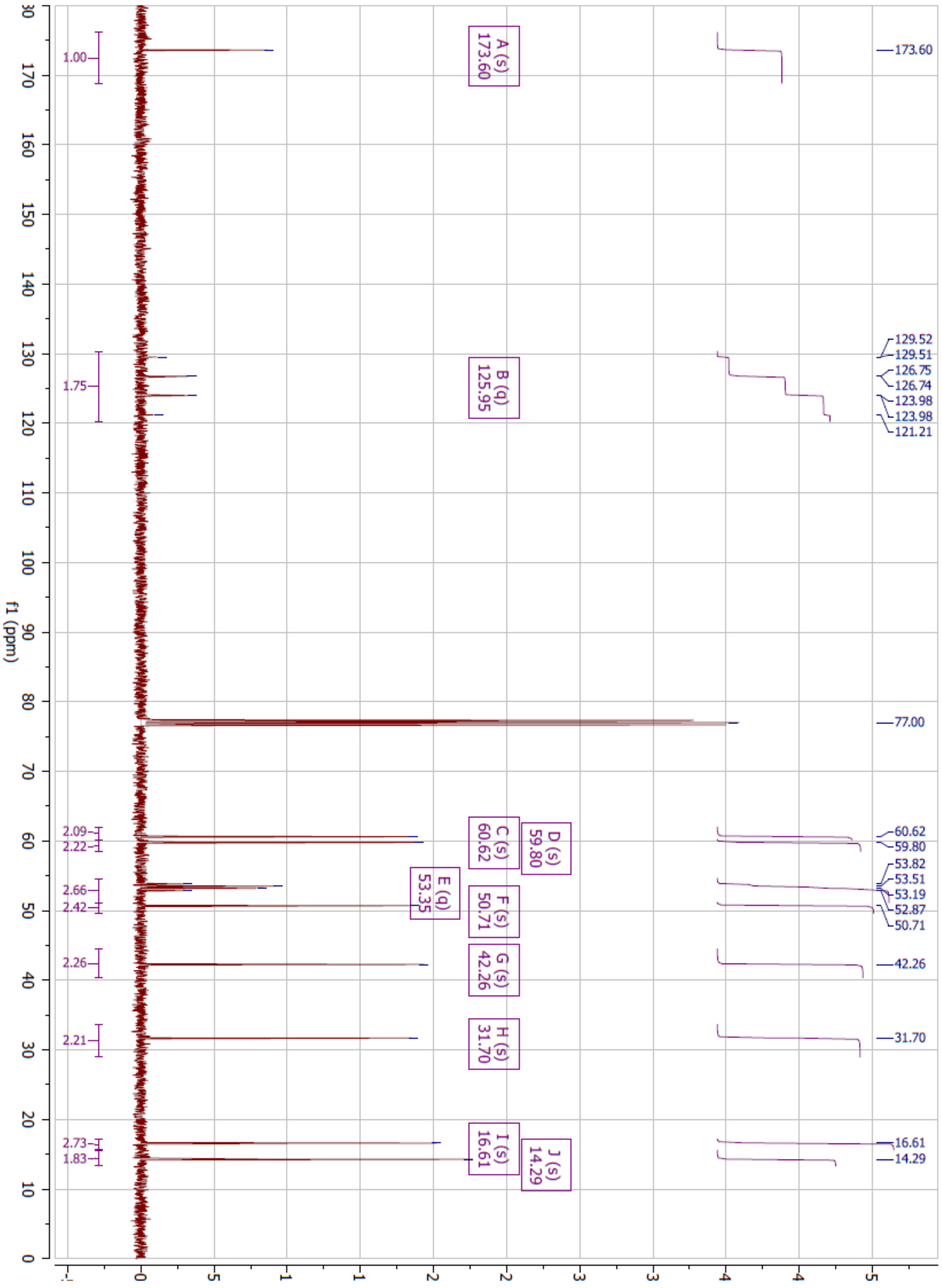

Supplementary Figure 127.

^{19}F-NMR (376 MHz, $CDCl_3$) ethyl *N*-(3-chloropropyl)-*N*-(2,2,2-trifluoroethyl)alaninate (**39**)

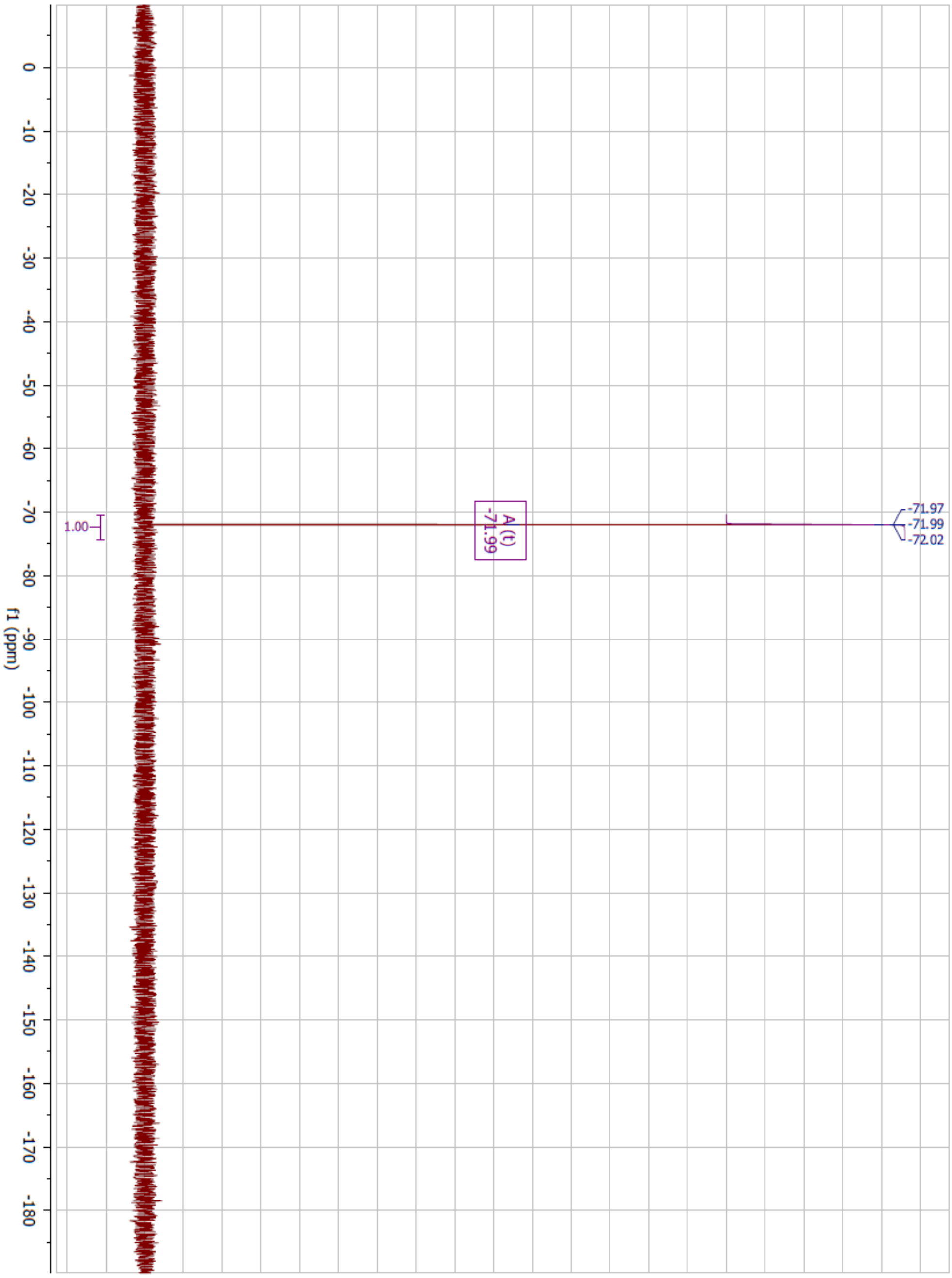

Supplementary Figure 128.

^{1}H-NMR (400 MHz, $CDCl_3$) *N*-benzyl-2,2,2-trichloro-*N*-methylethan-1-amine (**42**)

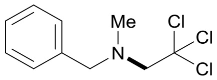

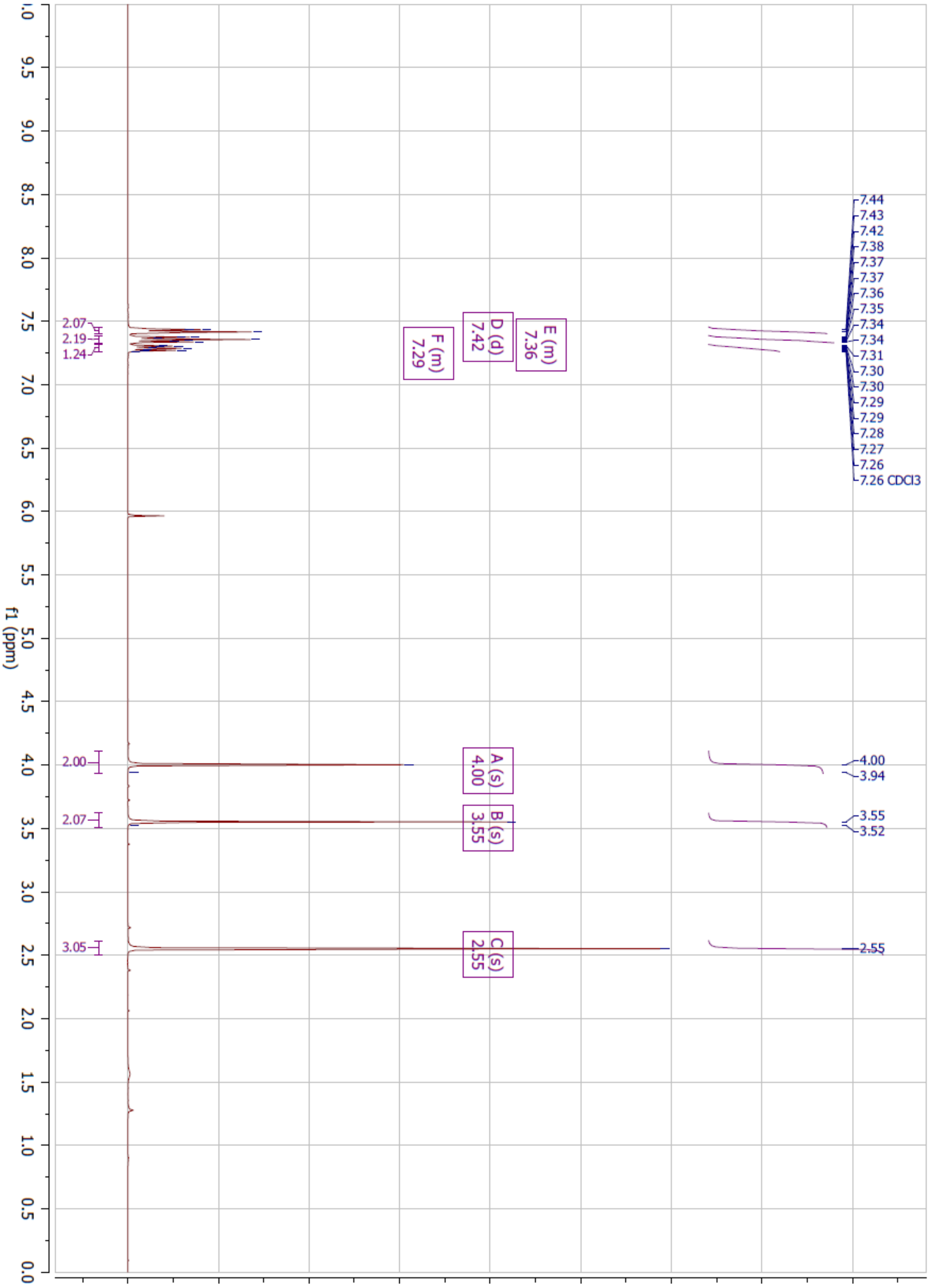

Supplementary Figure 129.

^{13}C-NMR (101 MHz, $CDCl_3$) *N*-benzyl-2,2,2-trichloro-*N*-methylethan-1-amine (**42**)

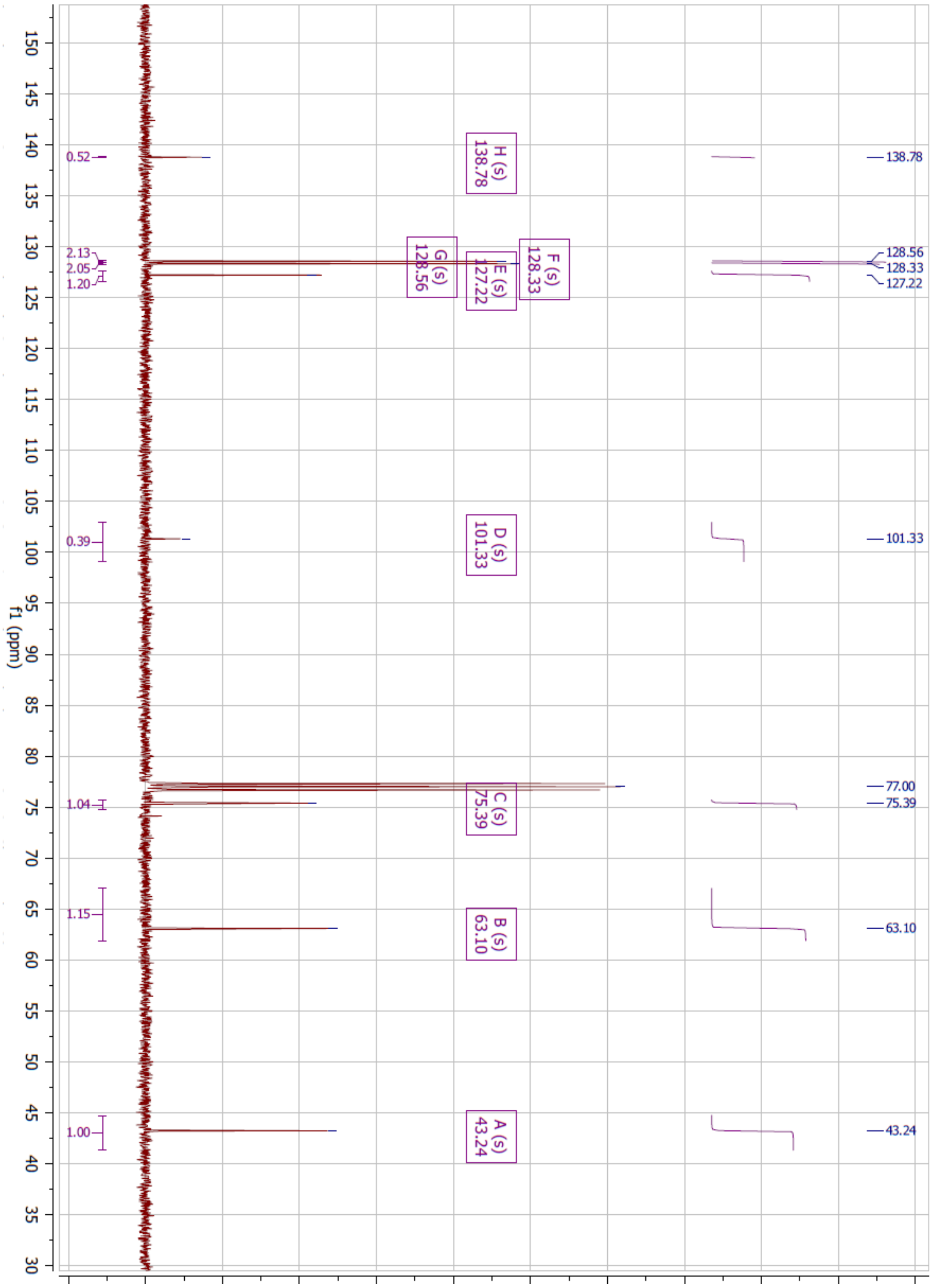

Supplementary Figure 130.

^{1}H-NMR (400 MHz, $CDCl_3$) *N*-benzyl-2,2-difluoro-*N*-methylethan-1-amine (**43**)

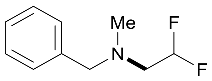

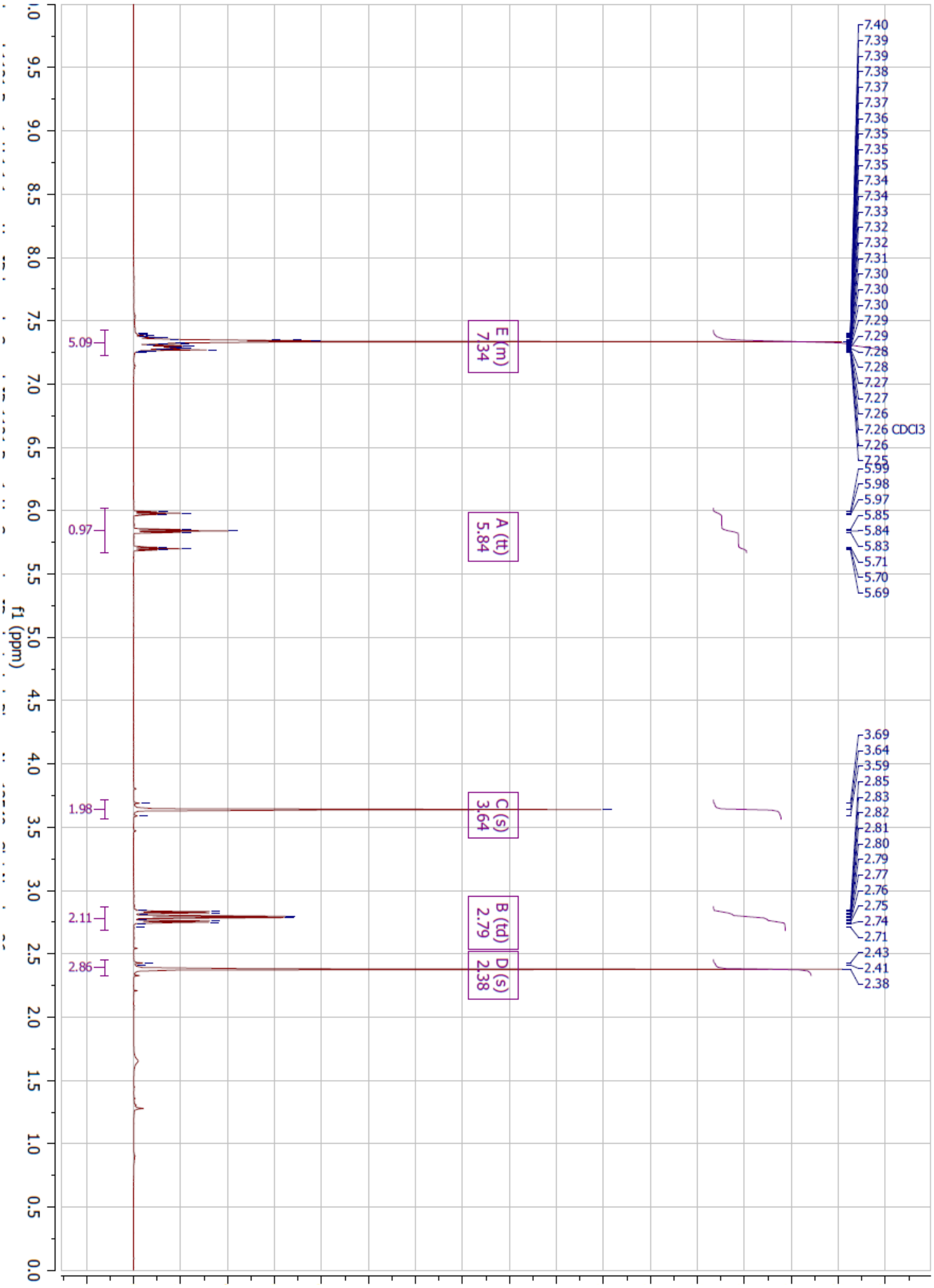

Supplementary Figure 131.

^{13}C-NMR (101 MHz, $CDCl_3$) *N*-benzyl-2,2-difluoro-*N*-methylethan-1-amine (**43**)

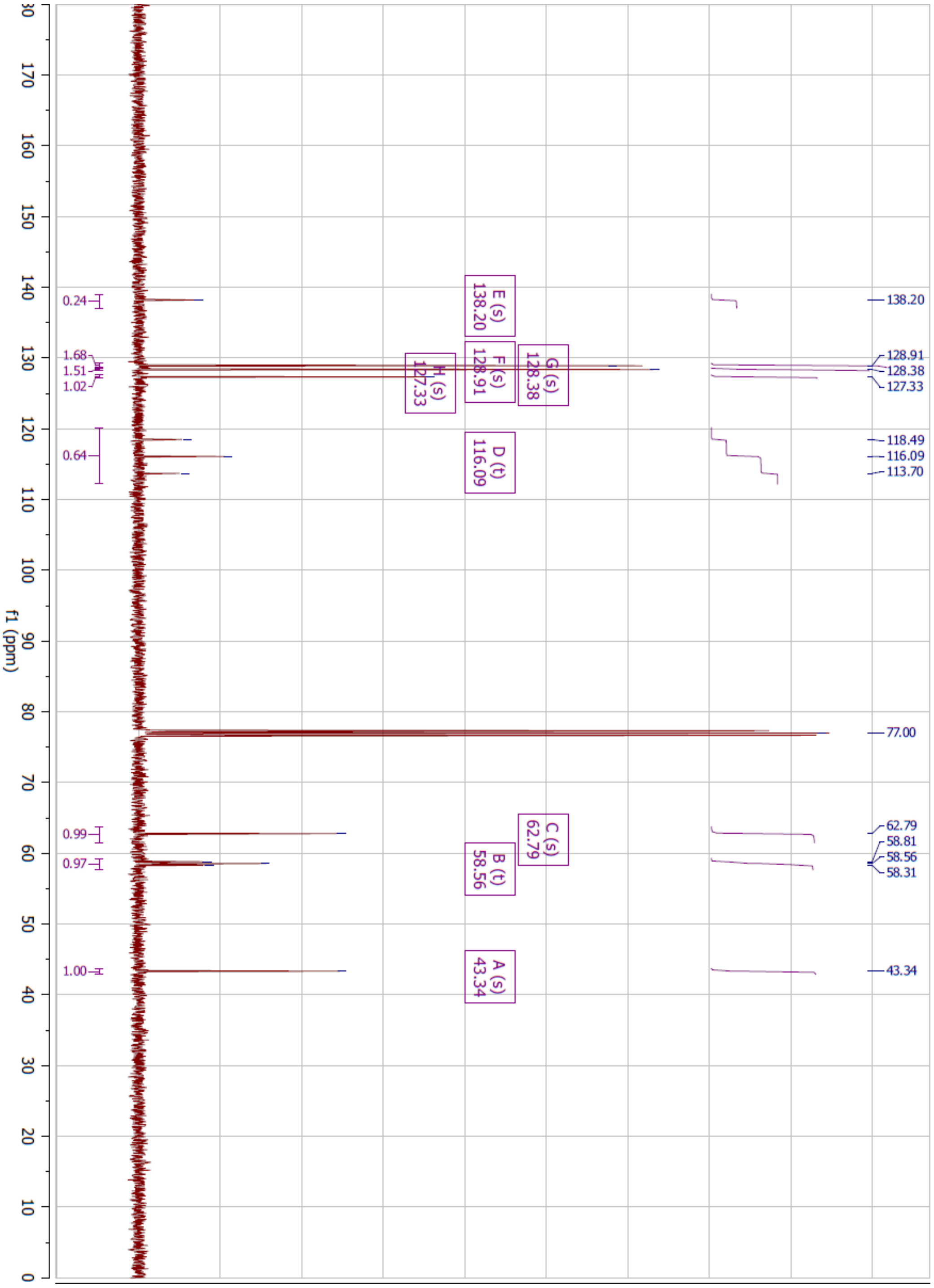

Supplementary Figure 132.

^{19}F-NMR (376 MHz, $CDCl_3$) *N*-benzyl-2,2-difluoro-*N*-methylethan-1-amine (**43**)

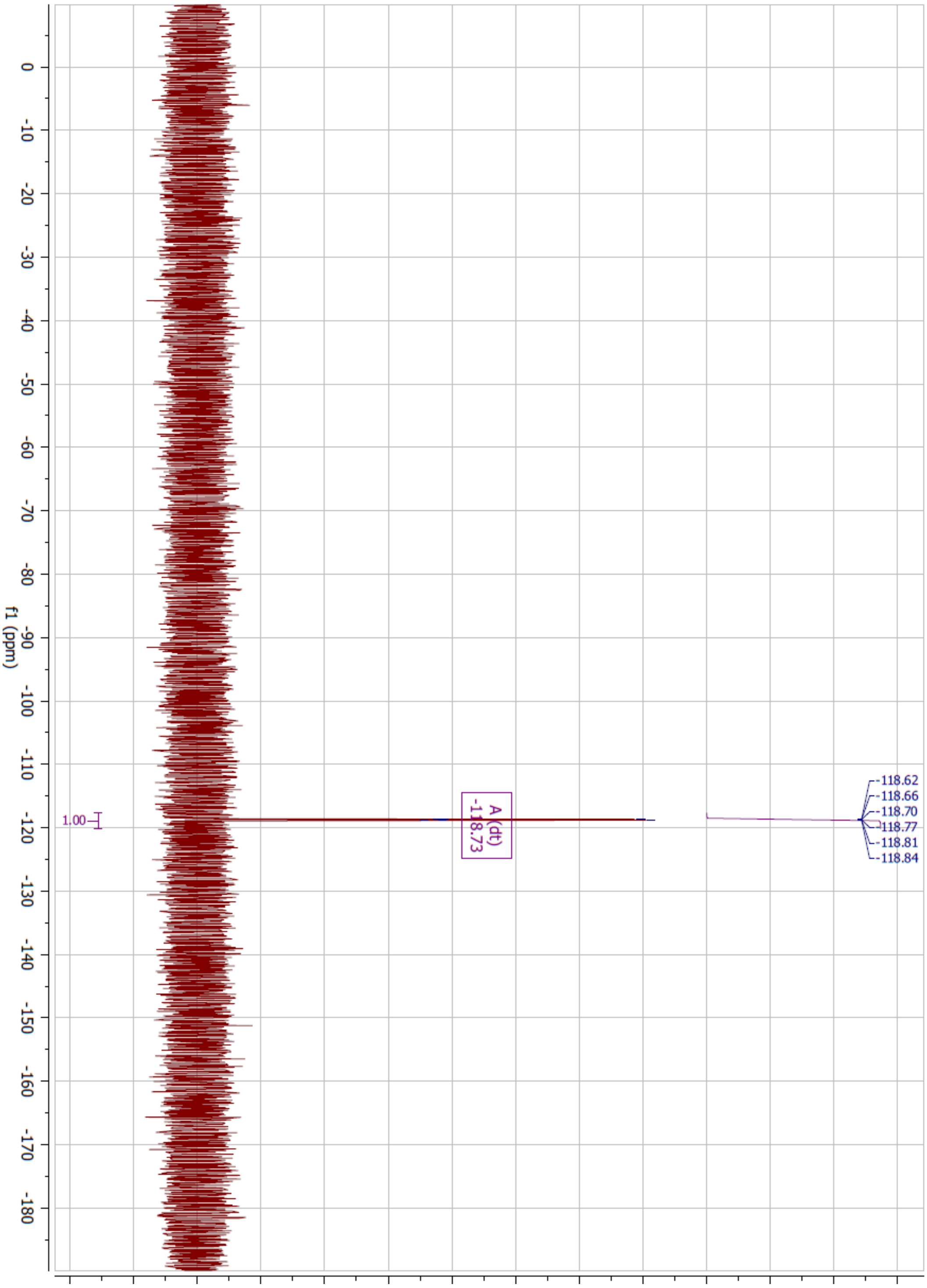

Supplementary Figure 133.

[1]**H-NMR (400 MHz, $CDCl_3$)** *N*-benzyl-2-chloro-*N*-methylethan-1-amine (**44**)

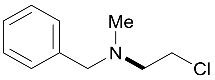

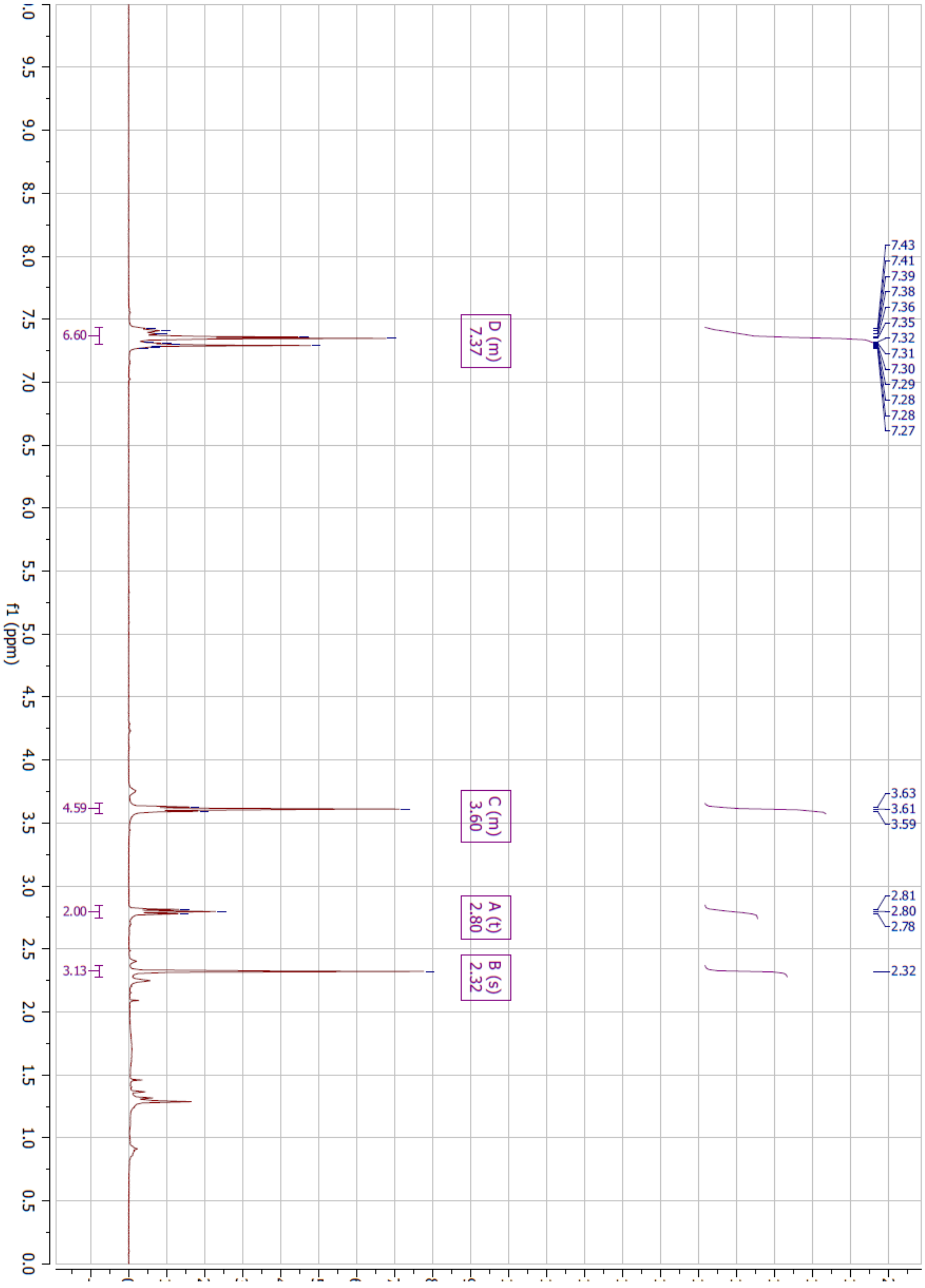

Supplementary Figure 134.

^{13}C-NMR (101 MHz, $CDCl_3$) *N*-benzyl-2-chloro-*N*-methylethan-1-amine (**44**)

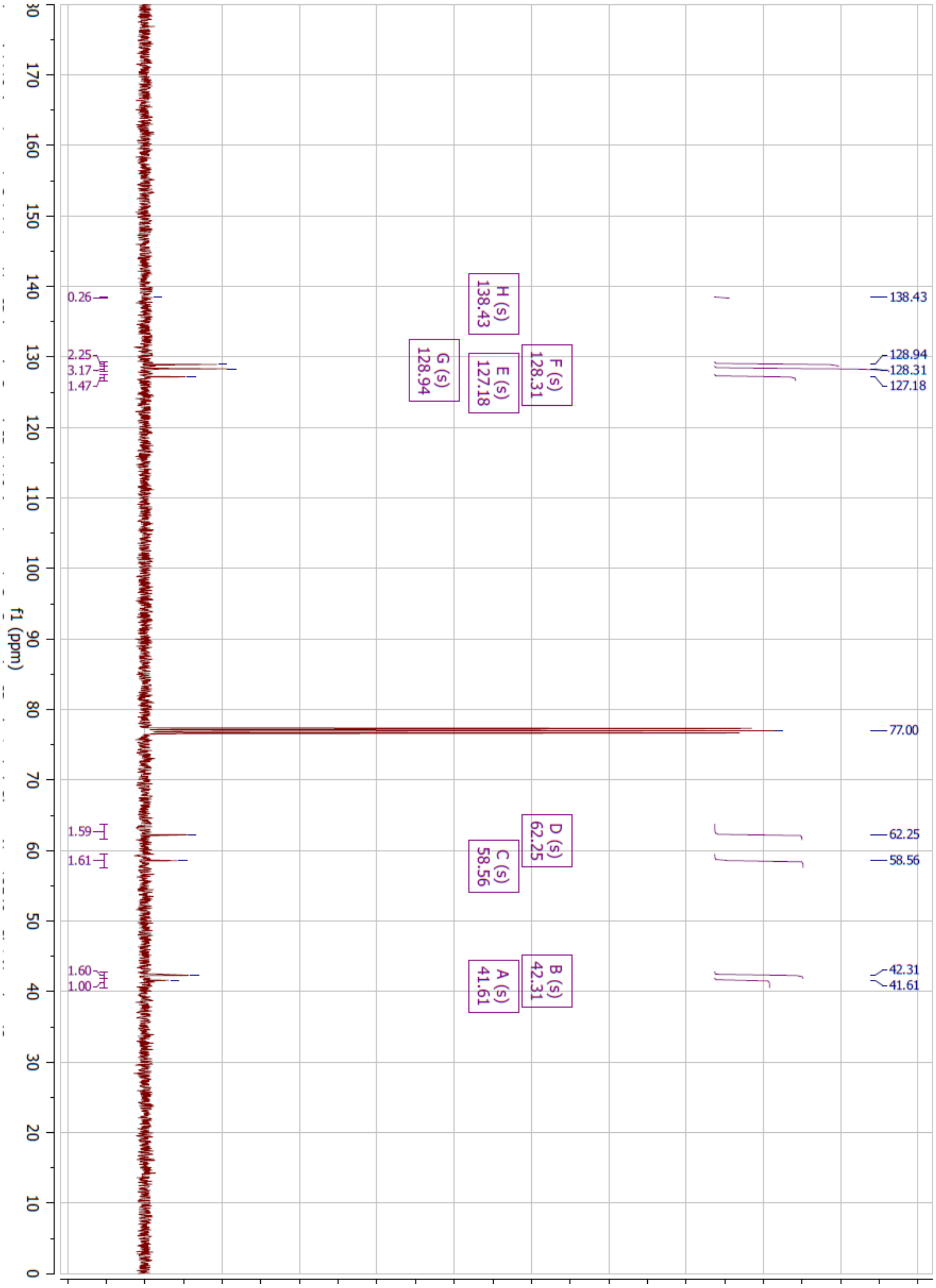

Supplementary Figure 135.

^{1}H-NMR (400 MHz, $CDCl_3$) *N*-benzyl-1-(3,5-dinitrophenyl)-*N*-methylmethanamine (**45**)

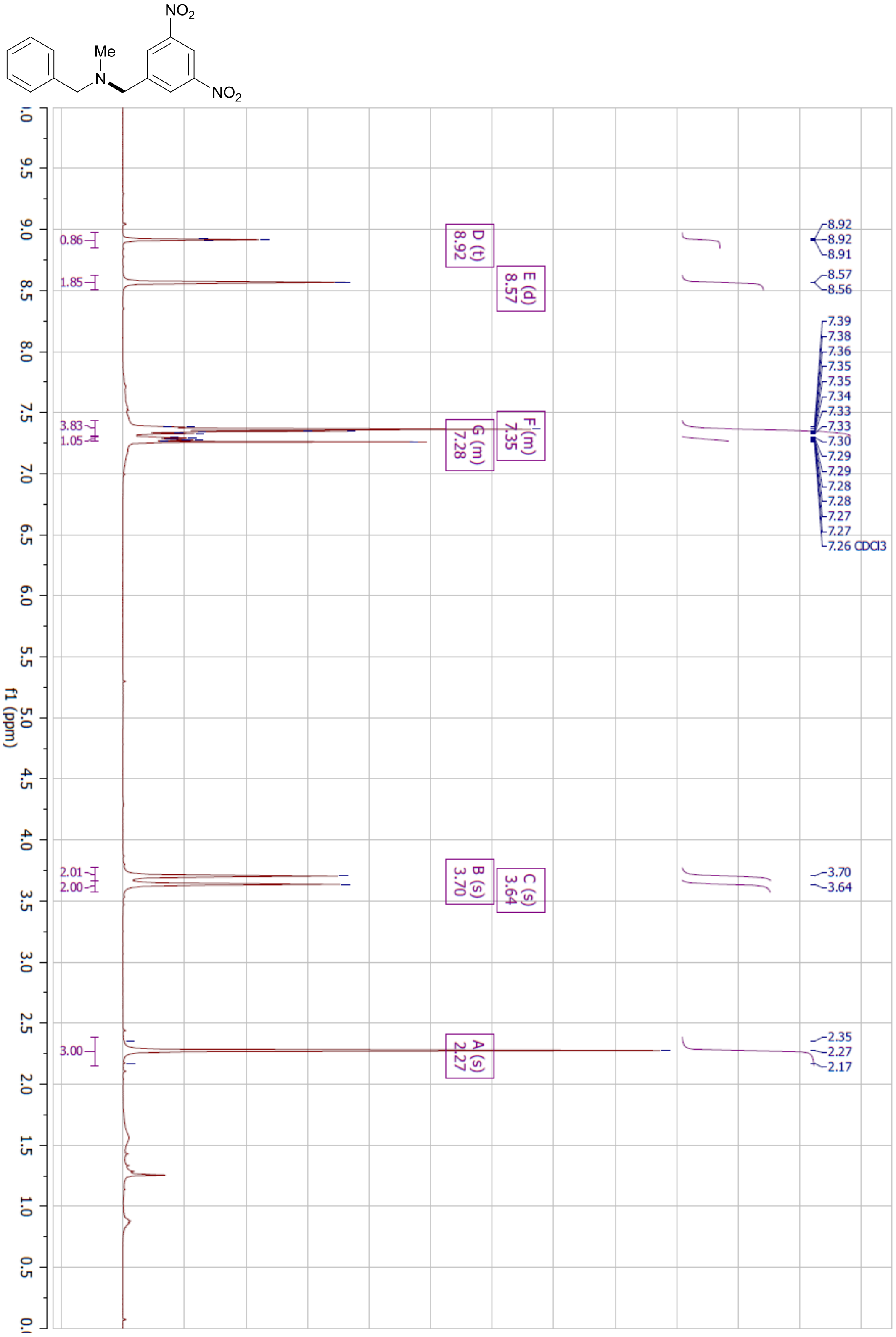

Supplementary Figure 136.

^{13}**C-NMR (101 MHz, CDCl$_3$)** *N*-benzyl-1-(3,5-dinitrophenyl)-*N*-methylmethanamine (**45**)

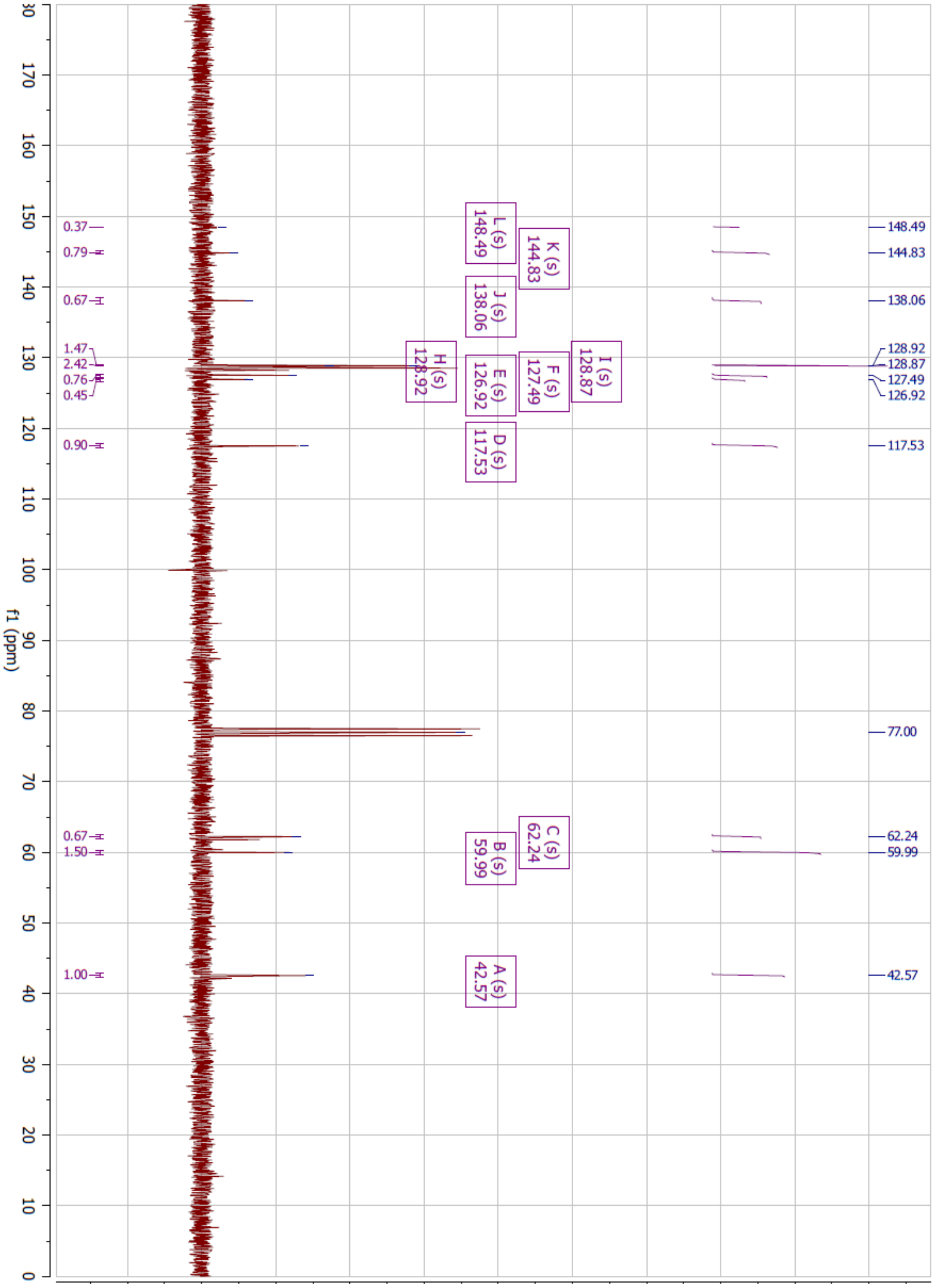

Supplementary References

1. Mimura, H., Kawada, K., Yamashita, T., Sakamoto, T. & Kikugawa, Y. Trifluoroacetaldehyde: A useful industrial bulk material for the synthesis of trifluoromethylated amino compounds. *J. Fluor. Chem.* **131,** 477–486 (2010).
2. Hansen, R. L. Perfluoroalkanesulfonate Esters as Alkylating Agents. *J. Org. Chem.* **30,** 4322–4324 (1965).
3. Ates, C., Janousek, Z. & Viehe, H. G. Trifluoroethylidenation of compounds with activated methylene groups. *Tetrahedron Lett.* **34,** 5711–5714 (1993).
4. Bailey, J. M., Booth, H., Al-Shirayda, H. A. R. Y. & Trimble, M. L. Ring inversion equilibria in 4-chloro-, 4-bromo-, and 4-methoxy-1-alkylpiperidines in a non-polar solvent. *J. Chem. Soc. Perkin Trans. 2* 737 (1984).
5. Sekiya, M., Matsuda, O. & Ito, K. Decarboxylation reactions. III. Reaction of N,N'- and N,O-linked methylene compounds with carboxylic acids. *Chem. Pharm. Bull. (Tokyo).* **23,** 1579–1585 (1975).
6. Yoshida, K. *et al.* MexAB-OprM specific efflux pump inhibitors in Pseudomonas aeruginosa. Part 6: Exploration of aromatic substituents. *Bioorg. Med. Chem.* **14,** 8506–8518 (2006).
7. Bihan, G. Le *et al.* Design and Synthesis of Imidazoline Derivatives Active on Glucose Homeostasis in a Rat Model of Type II Diabetes. 2. Syntheses and Biological Activities of 1,4-Dialkyl-, 1,4-Dibenzyl, and 1-Benzyl-4-alkyl-2-(4‘,5‘-dihydro-1‘H- imidazol-2‘-yl)piperazines a. *J. Med. Chem.* **42,** 1587–1603 (1999).
8. Riss, P. J. & Aigbirhio, F. I. A simple, rapid procedure for nucleophilic radiosynthesis of aliphatic [18F]trifluoromethyl groups. *Chem. Commun.* **47,** 11873–5 (2011).
9. Andrews, K. G., Summers, D. M., Donnelly, L. J. & Denton, R. M. Catalytic reductive N-alkylation of amines using carboxylic acids. *Chem. Commun.* **52,** 1855–8 (2016).
10. Funabiki, K. Maufacturing Method of α-Fluoroaldehyde Equivalent. (2016). JP2016084310 (A)
11. Landge, S. M., Borkin, D. A. & Török, B. Microwave-Assisted Preparation of Trifluoroacetaldehyde (Fluoral): Isolation and Applications. *Tetrahedron Lett.* **48,** 6372–6376 (2007).
